# *In silico* identification of drug targets and vaccine candidates against *Bartonella quintana*: a subtractive proteomics approach

**DOI:** 10.1590/0074-02760230040

**Published:** 2024-04-22

**Authors:** Shabir Ahmad, Hugo Verli

**Affiliations:** 1 Universidade Federal do Rio Grande do Sul, Centro de Biotecnologia, Porto Alegre, RS, Brasil

**Keywords:** Bartonella quintana, subtractive proteomics, drug targets, vaccine candidate

## Abstract

**BACKGROUND:**

The availability of genes and protein sequences for parasites has provided valuable information for drug target identification and vaccine development. One such parasite is *Bartonella quintana*, a Gram-negative, intracellular pathogen that causes bartonellosis in mammalian hosts.

**OBJECTIVE:**

Despite progress in understanding its pathogenesis, limited knowledge exists about the virulence factors and regulatory mechanisms specific to *B. quintana*.

**METHODS AND FINDINGS:**

To explore these aspects, we have adopted a subtractive proteomics approach to analyse the proteome of *B. quintana*. By subtractive proteins between the host and parasite proteome, a set of proteins that are likely unique to the parasite but absent in the host were identified. This analysis revealed that out of the 1197 protein sequences of the parasite, 660 proteins are non-homologous to the human host. Further analysis using the Database of Essential Genes predicted 159 essential proteins, with 28 of these being unique to the pathogen and predicted as potential putative targets. Subcellular localisation of the predicted targets revealed 13 cytoplasmic, eight membranes, one periplasmic, and multiple location proteins. The three-dimensional structure and B cell epitopes of the six membrane antigenic protein were predicted. Four B cell epitopes in KdtA and mraY proteins, three in lpxB and BQ09550, whereas the ftsl and yidC proteins were located with eleven and six B cell epitopes, respectively.

**MAINS CONCLUSIONS:**

This insight prioritises such proteins as novel putative targets for further investigations on their potential as drug and vaccine candidates.

*Bartonella quintana* is a bacterial pathogen that can infect humans and cause trench fever, first identified during World War I and spread by human body lice (*Pediculus humanus corporis*).^1^ Although other arthropods have been associated with transmitting *Bartonella* species, the human body louse is considered the primary vector for trench fever.^1^ ^,^ ^2^ ^,^ ^3^ This bacterial infection is associated with bartonellosis, a condition characterised by prolonged bacteraemia and found in a wide range of host reservoirs such as humans, cats, dogs, rabbits, rodents, horses, cattle, and other wild animals.^1^ ^,^ ^3^ ^,^ ^4^ ^,^ ^5^ ^,^ ^6^ While several *Bartonella* species can cause fever and culture-negative endocarditis in humans and animals, *B. quintana* and *B. henselae* (a causal agent of cat scratch disease) are most commonly associated with human endocarditis cases.^7^ ^,^ ^8^ ^,^ ^9^ The three species *B. quintana*, *Bartonella bacilliformis*, and *B. henselae* are the causative agents for human disease, producing a variety of signs from mild symptoms like fever, headache, and malaise to more serious symptoms such as hallucinations.^4^ ^,^ ^6^ ^,^ ^10^ ^,^ ^11^ ^,^ ^12^ The severity of clinical manifestations is often correlated with the immune status of the patient, and the large spectrum of animal reservoir hosts and arthropod vectors that can transmit these bacteria among animals and humans are major public health concerns.

Co-infection with *B. henselae* in people suffering from persistent symptoms after borreliosis treatment has been reported, and transmission with other pathogens, such as *Borrelia*, transmitted by ticks, can contribute to atypical disease progression.^9^ ^,^ ^13^ ^,^ ^14^ As reviewed by Lantos & Wormser, laboratory diagnostics were not performed in most reported cases of *Bartonella* and *Borrelia* co-infections.^15^ With different populations exposed to animals and arthropod vectors in various dimensions, veterinarians, veterinary technicians, and zookeepers are at increased risk of infection with *Bartonella* spp.^15^ ^,^ ^16^ ^,^ ^17^ *Bartonella* infections are suspected of having contributed to the deaths of two veterinarians in 2013.^15^

Recent progress in the field of bioinformatics has generated various *in silico* strategies and drug-designing approaches that reduce the time and cost associated with trial-and-error experiments for drug development.^18^ These methods serve to shortlist potential drug targets that may be used for experimental validation.^19^ ^,^ ^20^ ^,^ ^21^ ^,^ ^22^ ^,^ ^23^ One such approach is subtractive proteomics, an *in silico* method used to identify essential and non-host homologous proteins within a pathogen proteome.^18^ ^,^ ^24^ ^,^ ^25^ ^,^ ^26^ ^,^ ^27^ By selecting essential proteins unique to pathogen survival and propagation, the subtractive proteomics approach allows the identification of novel drug targets and vaccine candidates within a pathogen, as shown for life-threatening pathogens such as *Pseudomonas aeruginosa*,^28^ *Streptococcus pneumonia* ^29^ and *Mycobacterium tuberculosis*.^30^ ^,^ ^31^

Inceptive steps in discovering a novel drug target or vaccine candidate include identifying target proteins.^32^ However, to our knowledge, no studies have examined *B. quintana* utilising subtractive proteome analysis. Therefore, in this study, we employed an approach to identify potential drug targets and vaccine candidates in *B. quintana* by predicting the subcellular localisation of non-host homologous essential proteins, conducting druggability and antigenicity analyses, and predicting the three-dimensional (3D) structure of membrane antigenic proteins using AlphaFold. This study provides a novel insight into potential drug targets and vaccine candidates in *B. quintana*.

## MATERIALS AND METHODS

*Retrieval of proteomes of host and pathogen* - The selection of *B. quintana* str. Toulouse project accession PRJNA44’s proteome was based on the organism’s name and the availability of its pathways on the KEGG database. The proteome of *B. quintana* (accession no. GCF_000046685.1) and *Homo sapiens* (accession no. GCF_000001405.40) was obtained from the National Centre for Biotechnology Information (NCBI).^33^ ^,^ ^34^

*Screening of non-host homologous proteins* - To eliminate paralogs or duplicates from the *B. quintana* proteome, a 60% sequence identity threshold was applied using Cluster Database at High Identity with Tolerance (CD-HIT)^27^ ^,^ ^35^ before further analysis. The resulting protein set was then subjected to BLASTp against the *H. sapiens* proteome, using an E-value cut-off of 10^-5^, as reported by Altschul et al.^36^ Only the protein sequences of *B. quintana* that exhibited no similarity with *H. sapiens* proteins were considered for subsequent analysis.

*Screening of essential proteins in B. quintana* - To identify essential proteins in *B. quintana*, a BLASTp analysis was conducted on non-homologous protein sequences using a database of essential proteins downloaded from the Database of Essential Genes (DEG), as reported by Luo et al.^37^ The analysis utilised an E-value cut-off score of 10^-5^ and ≥ 30% identity, while a minimum bit-score cut-off of 100 was used to include proteins that could potentially represent essential genes. The resulting protein sequences represent the non-homologous essential proteins of *B. quintana*.

*Metabolic pathway analysis* - The essential proteins of *B. quintana* were subjected to metabolic pathway analysis using the Kyoto Encyclopedia of Genes and Genomes (KEGG) and the functional annotation of the genes was identified using the Automatic Annotation Server (KAAS). The KAAS analysis was performed using the BLAST option and the assignment method was used as a bi-directional best hit (BBH), which provided KEGG Orthology (KO) codes indicating the presence of particular proteins in the specific pathways of the pathogen. Comparative analysis of the metabolic pathways of the host and pathogen proteins was carried out using the KEGG pathway database, as reported by.^38^ ^,^ ^39^ The aim was to identify unique proteins involved in pathogen-specific metabolic pathways, which could serve as potential therapeutic targets.

*Subcellular localisation and sequence conservation analysis of unique essential proteins* - The analysis of unique essential proteins was performed using two online servers: CELLO v2.5 and QuickGo. CELLO v2.5, with a reported accuracy of up to 89% based on a training dataset of 1443 proteins,^40^ was used to identify the location of essential proteins in various cellular organelles such as the outer membrane, cytoplasm, and extracellular space. The analysis was performed using the parameters of the organism as gram-negative and the sequence parameters as proteins. Additionally, QuickGo, with an accuracy of up to 65% based on training datasets containing over 1000 proteins,^41^ was utilised for the same purpose. These tools provided valuable insights into the subcellular localisation of essential proteins in the study. These essential target proteins were used to map these proteins to the 25 genomes of *B. quintana* strains available on the NCBI website and were analysed using tBLASTn. The tBLASTn is a variant of the BLAST algorithm, which compares a query consisting of an amino acid sequence with the translated nucleotide (coding sequence) database. The unique essential target protein sequences were uploaded as a query using a database search as RefSeq Genome Database (refseq_genomes) against the organism *B. quintana* (taxid:803). Protein sequence searches are performed on all six translated frames in the database to enable protein-to-protein comparisons. To perform multiple sequence alignment (MSA), the amino acid sequences of the target protein were downloaded and then uploaded to CLUSTAL W.^42^ The sequences that had been aligned were subsequently imported into ESPript 3.0 (espript.ibcp.fr/ESPript/ESPript)^43^ in order to map sequence conservation.

*Evaluation of druggability potential of the unique essential proteins* - To identify potential drug targets in *B. quintana*, the essential proteins associated with unique pathways were subjected to BLASTp analysis with a bit score > 100 and E value < 10^-3^ against a customised database of Food and Drug Administration (FDA)-approved drug targets, as reported by.^44^ The protein targets that exhibited BLASTp hits against the FDA-approved drug target database were considered druggable targets.

Conversely, protein targets that did not exhibit any matches with the FDA-approved drug target database were considered novel targets for new drug identification. These novel targets offer the potential for the discovery of new drugs that can target unique metabolic pathways in *B. quintana*, thus enhancing the current arsenal of drugs available for the treatment of infections caused by this pathogen.

*Analysis of virulence factors (VFs)* - The Virulence Factor Database (VFDB) was employed to identify VFs in *B. quintana*. The VFDB is a comprehensive database comprising four categories of VFs, namely offensive, defensive, non-specific, and virulence-associated regulated proteins, from 25 pathogenic bacteria, as reported by.^45^

To identify potential VFs in *B. quintana*, the non-homologous essential proteins were subjected to a BLASTp search against the database of protein sequences from the VFDB core dataset (R1), with a cut-off E-value of 10^-5^. This approach allowed us to identify *B. quintana* proteins that exhibit significant sequence similarity to known VFs, which can aid in understanding the virulence mechanisms of this pathogen and identify potential targets for the development of new therapeutics.

*Antigenicity analysis and 3D structure prediction* - We employed the VaxiJen server developed for the antigenicity prediction of the membrane proteins. The VaxiJen server is a computational tool for predicting protein sequences’ antigenicity. It uses auto-cross covariance (ACC) transformation followed by a probabilistic neural network (PNN) algorithm to classify proteins as either antigenic or non-antigenic. This server has an accuracy range of 70% to 89% for antigenicity prediction. Proteins with scores > 0.4 were considered antigenic, and those with scores < 0.4 were labelled non-antigenic, leading to further investigation of antigenic proteins.^46^

Due to the absence of crystal structure data for the predicted antigenic membrane target proteins, we utilised AlphaFold^47^ and ColabFold^48^ to predict their 3D models using the parameters number of relax (num_relax = 1), template mode PDB100, number of cycles (num_recycles = 3), and number of seeds (num_seeds = 1). These tools employ a threading technique that considers protein folding rather than similarities to generate models of the proteins.

*B cell epitope prediction* - To predict B cell linear epitopes, we employed three algorithms: BCPred,^49^ which uses a default epitope length of 14 amino acids and reports only non-overlapping epitopes with over 75% specificity; ABCPred,^50^ which uses an epitope length of 14 amino acids and reports only non-overlapping epitopes above the 0.8 thresholds; and BepiPred,^51^ which requires a minimum threshold of 0.5 and at least six amino acids to identify an epitope.

Amino acid sequences identified as probable epitopes by at least two algorithms were then subjected to antigenicity analysis using VaxiJen v.2.0.^46^ VaxiJen classifies antigens based on their physicochemical features, and the default recommendation threshold for analysing the sequence of parasite proteins was 0.5.

The presence of discontinuous epitopes in the 3D models of the predicted membrane proteins was evaluated using two different prediction tools. ElliPro^52^ employs Thornton’s method along with residue clustering algorithms to predict epitopes with a minimum score of 0.5 and a maximum distance of six amino acids. The second tool used was DiscoTope,^53^ which calculates the surface accessibility of epitope fragments and uses the standard threshold of -3.7 in this analysis.

## RESULTS

**Fig. 1:**
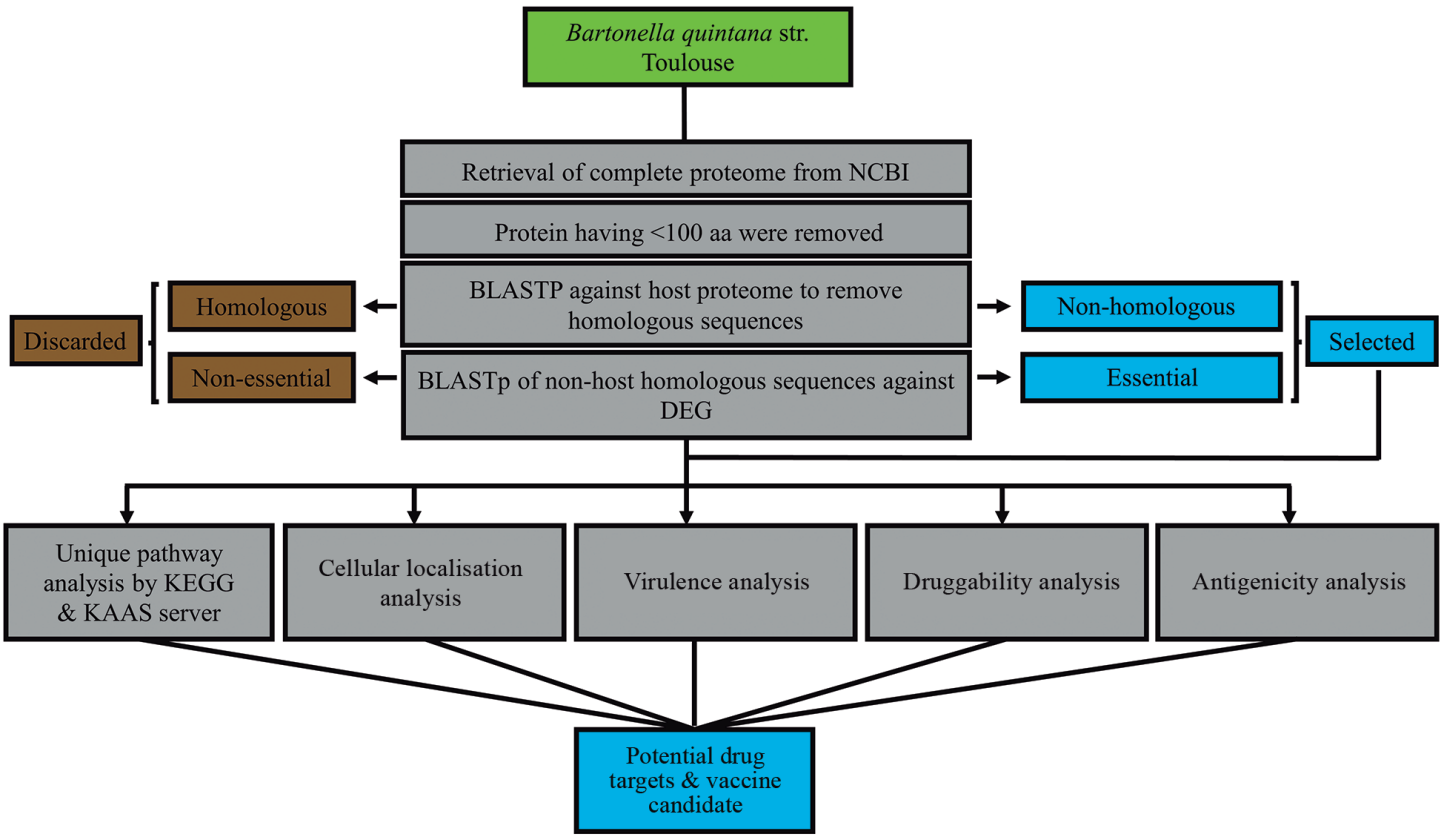
stepwise analysis flowchart for the subtractive proteomics analysis of *Bartonella quintana.* The green colour box consists of the pathogen name (*B. quintana* str. Toulouse), which is the starting step of the analysis. The grey colour box represents the methodological aspects at each analysis step. The gold colour box represents the homologous and non-essential data and is discarded. The light blue colour box represents the essential non-homologous and therapeutic targets.

*Identification of non-host homologous and essential proteins analysis* - The proteome of *B. quintana* was analysed in this study. Initially, 1197 proteins were retrieved from NCBI, and those with an amino acid sequence of fewer than 100 residues were removed, resulting in 1,026 non-redundant proteins according to the stepwise flowchart as shown in (Fig. 1). BLASTp analysis was conducted against the human proteome and found 660 proteins that were non-homologous to *H. sapiens*. The remaining 336 proteins were excluded. The 660 proteins were then subjected to DEG analysis to find the essential genes needed for the survival of the pathogen. This yielded 159 hits that were involved in structural organisation, nutrient uptake, pathogenesis, and other essential processes for *B. quintana* survival (Table I).

**TABLE I:** Classification of *Bartonella quintana* proteins using subtractive proteome analysis

SN	Step	No. of proteins in *B. quintana* str. Toulouse
1	Total proteome	1197
2	Non-paralogs (> 60% identical) in CD-HIT	1026
3	Non-homologs	660
4	Essential proteins in DEG	159
5	Unique metabolic pathway KEGG	25
6	Essential proteins involved in unique pathways	28
7	Druggable targets with a cut-off E-value of 10^-5^	7
8	Novel targets with a cut-off E-value of 10^-5^	13
9	Antigenic vaccine candidate	6

CD-HIT: Cluster Database at High Identity with Tolerance; DEG: Database of Essential Genes; KEGG: Kyoto Encyclopedia of Genes and Genomes; SN: serial number.

**Fig. 2:**
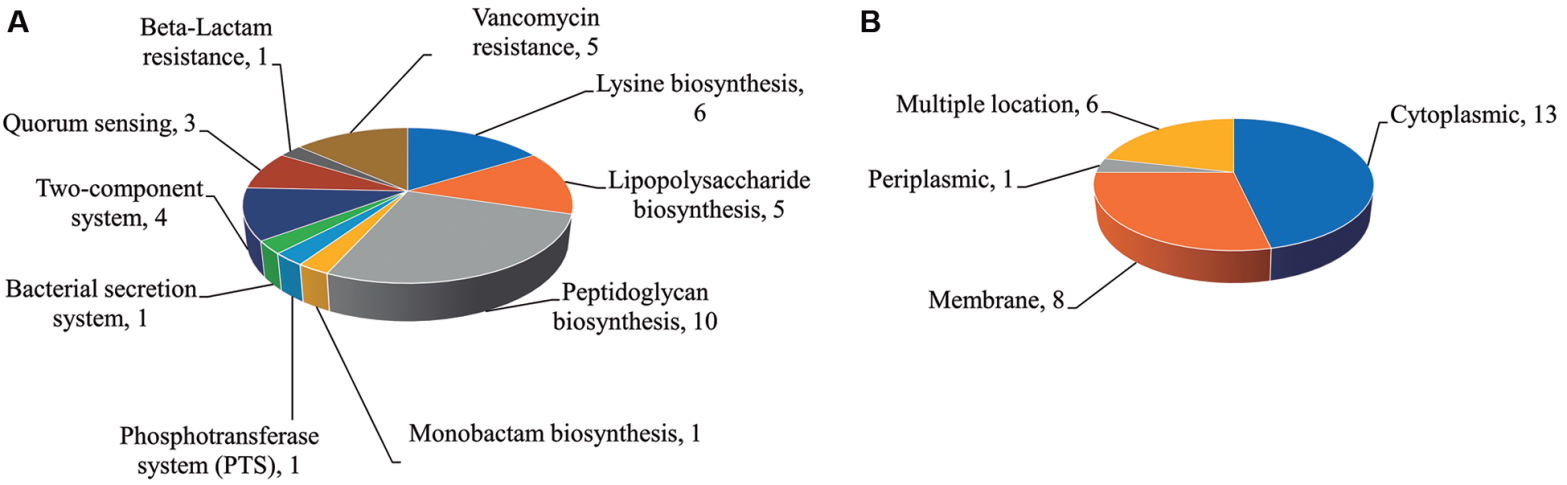
distribution of non-homologous essential therapeutic targets. (A) Unique essential proteins are present in each unique metabolic pathway; (B) Subcellular localisation of therapeutic target proteins show the majority are cytoplasmic.

*Metabolic pathway, subcellular localisation, and sequence conservation analysis* - 159 essential proteins were analysed to determine their involvement in metabolic pathways. Only 25 pathways specific to *B. quintana* and thus not present in humans were found, possessing 28 unique proteins non-homologous to humans. The pathways that are unique to *B. quintana* with the names of essential genes and their KO, have been presented in (Table II). Segregation of these 28 proteins into their metabolic pathways revealed that four proteins of them belong to the two-component system: 10 proteins in the peptidoglycan biosynthesis pathway, three proteins in the quorum-sensing pathway, five proteins in the lipopolysaccharide biosynthesis pathway, and six in the lysine biosynthesis pathway. Similarly, there are five proteins in the vancomycin pathway, one protein in the phosphotransferase system (PTS), one protein in the bacterial secretion system, one in beta-lactam resistance, and one in monobactam biosynthesis. Since these target proteins are essential and unique to *B. quintana*, their suppression will render bacteria more vulnerable to various medications, and proteins implicated in these pathways make better therapeutic targets (Table II, Fig. 2).

**TABLE II:** Non-homologous, essential, and unique proteins involved in twenty-five unique metabolic pathways and their druggability

SN	Accession no.	Gene name	KO list	Pathway’s name	DrugBank	Subcellular localisation	QuickGo	Virulence factor	Antigenicity
1	WP_011178881.1	*dapE*	K01439	Lysine biosynthesis	Druggable	Cytoplasmic	N/A	Non-virulent	-
2	WP_011178891.1	*BQ00560*	K07658	Two-component system	Novel	Cytoplasmic	N/A	Virulent	-
3	WP_011178913.1	*yfeA*	K11601	Two-component system	Novel	Multiple	N/A	Virulent	-
4	WP_011178939.1	*dnaA*	K02313	Two-component system	Novel	Cytoplasmic	Cytoplasm	Non-virulent	-
5	WP_011178959.1	*fruB*	K08483	Phosphotransferase system (PTS)	Novel	Multiple	Cytoplasm	Non-virulent	-
6	WP_011179066.1	*kdtA*	K02527	Lipopolysaccharide biosynthesis	-	Membrane	Membrane	Virulent	Antigenic
7	WP_011179067.1	*lpxK*	K00912	Lipopolysaccharide biosynthesis	Novel	Multiple	N/A	Virulent	Non-antigenic
8	WP_011179434.1	*lpxB*	K00748	Lipopolysaccharide biosynthesis	-	Membrane	N/A	Virulent	Antigenic
9	WP_011179438.1	*lpxD*	K02536	Lipopolysaccharide biosynthesis	Druggable	Cytoplasmic	N/A	Virulent	-
10	WP_011179451.1	*dacA2*	K07258	Peptidoglycan biosynthesis	Druggable	Periplasmic	N/A	Non-virulent	-
11	WP_011179558.1	*ybeJ*	K10001	Two-component system	Novel	Multiple	N/A	Virulent	-
12	WP_011179596.1	*lpxC*	K02535	Lipopolysaccharide biosynthesis	Novel	Cytoplasmic	N/A	Virulent	-
13	WP_011179600.1	*ddl*	K01921	Peptidoglycan biosynthesis, vancomycin resistance	Druggable	Cytoplasmic	Cytoplasm	Non-virulent	-
14	WP_011179602.1	*murC*	K01924	Peptidoglycan biosynthesis,	Novel	Cytoplasmic	Cytoplasm	Non-virulent	-
15	WP_011179603.1	*murG*	K02563	Peptidoglycan biosynthesis, vancomycin resistance	-	Membrane	Membrane	Non-virulent	Non-antigenic
16	WP_011179606.1	*mraY*	K01000	Peptidoglycan biosynthesis, vancomycin resistance	-	Membrane	Membrane	Virulent	Antigenic
17	WP_011179607.1	*murF*	K01929	Lysine biosynthesis, peptidoglycan biosynthesis, vancomycin resistance	Novel	Multiple	Cytoplasm	Non-virulent	-
18	WP_011179608.1	*murE*	K01928	Lysine biosynthesis, peptidoglycan biosynthesis	Novel	Cytoplasmic	Cytoplasm	Non-virulent	-
19	WP_011179609.1	*ftsl*	K03587	Peptidoglycan biosynthesis, beta-lactam resistance	-	Membrane	Membrane	Non-virulent	Antigenic
20	WP_011179659.1	*BQ09550*	K02034	Quorum sensing	-	Membrane	Membrane	Non-virulent	Antigenic
21	WP_011179660.1	*BQ09560*	K02033	Quorum sensing	-	Membrane	Membrane	Non-virulent	Non-antigenic
22	WP_011179677.1	*yidC*	K03217	Quorum sensing, bacterial secretion system	-	Membrane	Membrane	Non-virulent	Antigenic
23	WP_011179680.1	*dapB*	K00215	Lysine biosynthesis, monobactam biosynthesis	Novel	Cytoplasmic	Cytoplasm	Non-virulent	-
24	WP_011179695.1	*alr*	K01775	Vancomycin resistance	Druggable	Multiple	N/A	Non-virulent	-
25	WP_011179784.1	*murA*	K00790	Peptidoglycan biosynthesis	Druggable	Cytoplasmic	Cytoplasm	Non-virulent	-
26	WP_011179951.1	*BQ13390*	K00290	Lysine biosynthesis	Novel	Cytoplasmic	N/A	Non-virulent	-
27	WP_011179960.1	*dapF*	K01778	Lysine biosynthesis	Novel	Cytoplasmic	Cytoplasm	Non-virulent	-
28	WP_034449436.1	*murB*	K00075	Peptidoglycan biosynthesis	Druggable	Cytoplasmic	Cytoplasm	Virulent	-

KO list: Kyoto Encyclopedia of Genes and Genomes Orthology list; N/A: not available; SN: serial number.

The subcellular localisation analysis of the 28 therapeutic targets revealed that 13 proteins were cytoplasmic, eight were membrane, one was periplasmic, and six had multiple locations. Some of the many functions of bacterial secreted proteins include acting as immunogens, toxins, or virulence factors.^54^ Of the 28 final targets, not a single one was predicted as a secretory protein (Fig. 2). These protein targets were mapped across the 25 available strains of *B. quintana* (taxid:803) by analysing the 28 therapeutic targets of *B. quintana* str. Toulouse with NCBI tBLASTn. We analysed the tBLASTn strain data, and the amino acid sequences were downloaded and imported into CLUSTAL W to find sequence conversations across all the strains. To map the similarity information of the therapeutic targets across the *B. quintana* strains, the multiple sequence aligned file was subsequently uploaded to ESPript 3.0. The target proteins such as gene lpxD (accession no: WP_011179438.1), gene mraY (accession no: WP_011179606.1), and gene murB (accession no: WP_034449436.1) in all of the strains share the gene name and the accession number, showing 100% sequence conservation in the multiple sequence alignment. Residues that are conserved between the strain sequences and the template proteins (*B. quintana* str. Toulouse) are shaded red. While the unshaded residue is not conserved across the template and strain sequences [see Supplementary data (Fig. 1)].

*Druggability of unique essential proteins* - The cytoplasmic, periplasmic, and multiple localisation essential proteins involved in a distinctive metabolic pathway in *B. quintana* were subjected to further investigation to assess their druggability. The analysis showed that seven proteins of the shortlisted 20 unique, essential cytoplasmic, periplasmic, and multiple localisation proteins are druggable according to the FDA-approved DrugBank databases, while the remaining 13 proteins were predicted to be novel druggable targets (Table II).

*Virulence factor and antigenicity analysis of the vaccine candidate* - Further, the VFs for the 28 essential unique proteins of *B. quintana* were analysed, and 10 potential VFs were identified including the cytoplasmic and membrane proteins (Table II). Pathogen virulence-related membrane proteins have been shown to play an important role in adhesion and survival in host cells.^55^ Potential antigen candidates are membrane proteins, which are typically immunogenic and exposed on the cell surface. VaxiJen was used to assess the antigenicity of eight membrane proteins, and six proteins with an expected threshold value greater than 0.4 were considered antigenic. The remaining two proteins were deemed non-antigenic due to their values falling below the cut-off threshold.

**Fig. 3:**
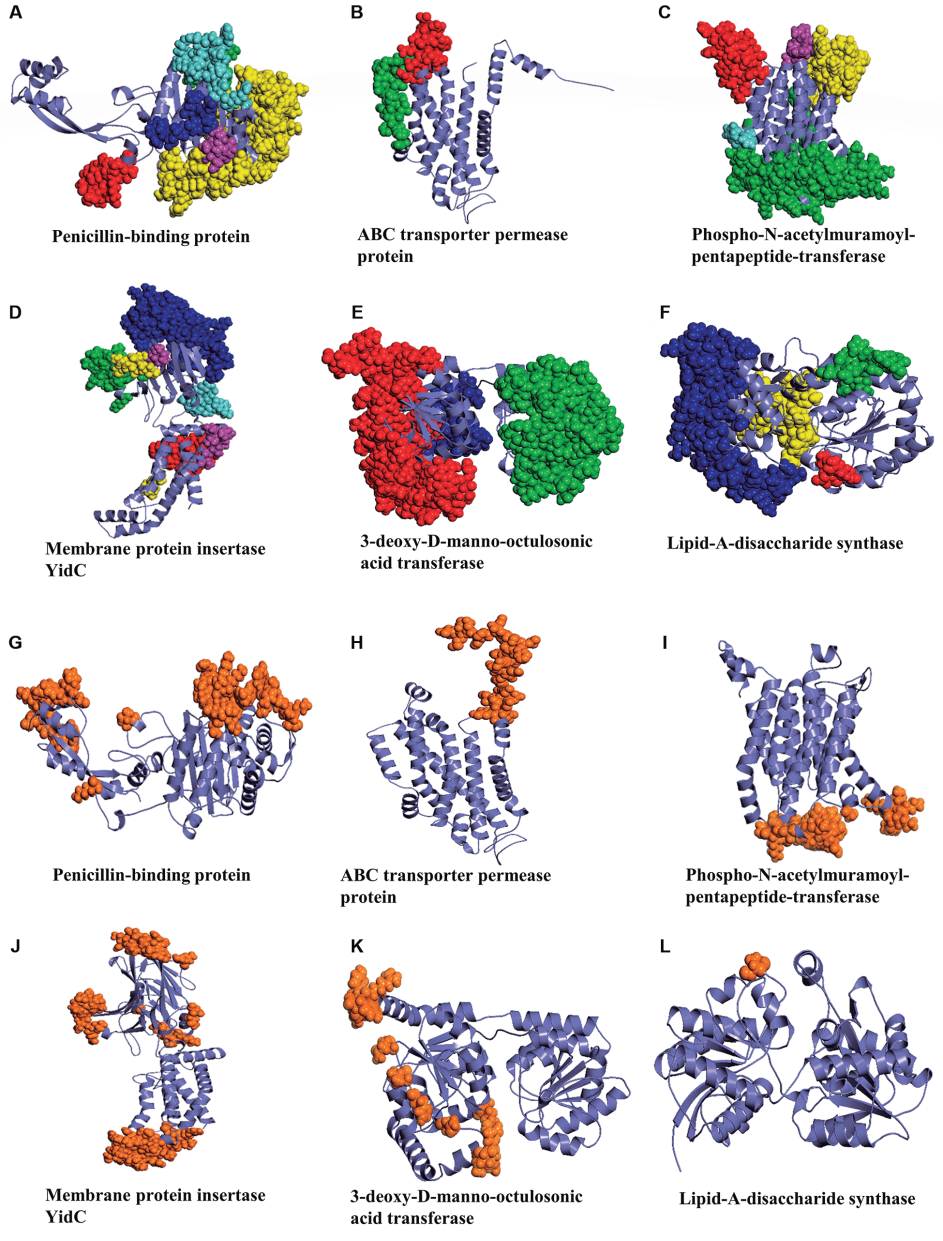
the discontinuous B cell epitope predicted using ElliPro. The coloured sphere shows the B cell epitope. The sphere is coloured according to the order of the PyMOL colour code Red > Green > Blue > Yellow > Magenta > Cyan. Red colours show the highest score of epitopes from A to F at the top of the figure. Similarity: discontinuous B cell epitopes predicted using DiscoTope are shown as brown from G to L at the bottom of the figure.

*B cell epitope and 3D structure prediction* - To predict B cell epitopes, *in silico* algorithms were used for the six membrane antigenic proteins. Linear epitope prediction algorithms (ABCPred, BCPred, and BepiPred) were employed to generate consensus sequences across all evaluated proteins. Four B cell epitopes were identified within the KdtA (IRGKEEWNRKKE, TIIVPRHPERSED, RRSNAIPARD, EALRQEMVDK) and mraY (ROGKGQPIRDGPQ, KVTKQTEKG, FVKDYFINLS, DYLQIHYVSG) proteins. LpxB (RPERAR, PIVPEYFNE, QKMKTEVPP) and BQ09550 (AHRLQPPSM, IFLTTI, LGAQLPSPE) were found to have three B cell epitopes each, whereas the ftsl (GQIEEAKGPGVL, GIPGIGF, QASSGYII, DFHGKNRPL, PLQTAVGAAA, RTKEQALKQAKQV, MRYLYKL, GRNAKVE, KVENGKYSKT, PKPEDGKYAA, IKPDFKKEYDSIL) and yidC (LKKYRLTVDKKS, EGNNTTLTPSTPVT, DSLKTEKYKTLA, EKYPDDRTKQQQA, MRMNPAPQDQT) proteins were mapped with eleven and six B cell epitopes, respectively [see Supplementary data (Fig. 2)]. The interaction of these epitopes with B cells is heavily influenced by their three-dimensional configuration. This interaction is heavily influenced by the spatial arrangement of amino acids in the epitope region. A linear or two-dimensional representation of the antigenic proteins cannot adequately capture the complexity of these interactions. A comprehensive 3D model is necessary to capture the accurate conformation of these epitopes. Given this requirement, to map B cell epitopes, the 3D structures of the final six selected antigenic proteins were predicted using AlphaFold. This approach was chosen due to the unavailability of crystal structures for these proteins. These models provided a clear understanding of the spatial orientation and configuration of potential B cell epitopes. Discontinuous epitope analyses (ElliPro and DiscoTope) revealed the presence of sequences of interest in all targets (Fig. 3). The results indicated that continuous epitopes are related to discontinuous epitopes and that multiple prediction tools should be utilised, as combining linear and conformational predictions improves the results.^56^

## DISCUSSION

In this study, the *B. quintana* proteome was analysed to identify its distinctive essential and virulent proteins. A comparison of *B. quintana’s* pathways with those of its host (human) revealed the presence of 28 unique metabolic pathways. These pathways included peptidoglycan biosynthesis, lipopolysaccharide biosynthesis, two-component system, lysine biosynthesis, Vancomycin resistance pathway, and the phosphotransferase system (PTS). Among these pathways, the peptidoglycan pathway is unique to bacteria, and inhibitors of this pathway are currently employed as antibiotics. Drugs target that peptidoglycan biosynthesis may reduce the pathogenicity of microorganisms.^57^ Additionally, the two-component system according to,^58^ plays a crucial role in the adaptation of bacteria to environmental and intracellular changes. Simultaneously, the lipopolysaccharide biosynthesis pathway is an essential metabolic pathway in *B. quintana*, providing stability to the outer membrane of Gram-negative bacteria by linking a conserved core oligosaccharide, lipid A, to a variable O-antigen.^59^ We found 13 non-homologous, unique essential proteins that can be used as drug targets and the virtual screening against these novel targets might be useful in the discovery of novel therapeutic compounds against *B. quintana*. Additionally, we found six non-homologous unique, and essential proteins that can be used as vaccine candidates. We located linear B cell epitope in the membrane antigenic proteins and revealed four B cell epitopes in KdtA and mraY proteins, three in lpxB and BQ09550, whereas the ftsl and yidC proteins were mapped with eleven and six B cell epitopes, respectively. A comparative analysis of the predicted antigenic epitopes region in the six vaccine candidate of *B. quintana* str. Toulouse with 24 genomes of *B quintana* str. shows similarity, except for one epitopes in the ftsl proteins (RTKEQALKQAKQV), where the underlined Q amino acid is replaced with K in only one of the *B. quintana* str. MF1.1 (RTKEQALKQAKKV). Formulations based on epitopes are currently considered one of the main strategies in vaccine development due to their cost and time benefit in process optimisation.^60^ One of the main advantages of this method is the ability to focus the immune response on crucial epitopes, avoiding the production of antibodies against regions of no interest.^61^ Additionally, discontinuous B cell epitopes were identified in the antigenic membrane proteins and mapped using the 3D structure of the antigenic proteins. We used AlphaFold to predict the 3D structure of these vaccine candidates, as there is no crystal structure available for these vaccine candidates. The predicted 3D structures provided a great aid in studying protein functions, dynamics, ligand interactions, and other components.^62^ By obtaining the structural information of these antigenic proteins, we have provided insights into their 3D organisation, the understanding of their functional properties, and the design of targeted interventions. Altogether, the present study has identified 28 essential non-homologous proteins in *B. quintana* that can be targeted for effective therapeutics*.* These proteins hold promise for further experimental validation to assess their suitability as therapeutic targets.

*In conclusion* - The subtractive proteomics approach employed in this study has provided a promising strategy for identifying potential drug targets and vaccine candidates against *B. quintana*. By scrutinising essential and unique proteins within the *B. quintana* proteome, we have identified 28 therapeutic targets. The prioritised list of targets generated by this method can serve as a valuable resource for the development of effective therapeutics against *B. quintana* and potentially other pathogens. Moreover, the targets identified in this study have not been previously characterised as drug targets against *B. quintana*, and therefore hold great potential for the discovery of new and innovative drug compounds. Overall, our findings highlight the utility of subtractive proteomics as a rapid and effective approach for identifying potential drug targets and vaccine candidates against pathogenic bacteria.
