# Supplementary material for: In silico identification of drug targets and vaccine candidates against Bartonella quintana: a subtractive proteomics approach
Source: Mem Inst Oswaldo Cruz. 2024 Apr 22;119:e230040. doi: 10.1590/0074-02760230040 (PMC11034861; doi:10.1590/0074-02760230040)
Supplement: Supplementary file 1 [file 1678-8060-mioc-119-e230040-s.pdf]

>WP_011178881.1 succinyl-diaminopimelate desuccinylase [Bartonella quintana str. Toulouse]

```
                1        10        20        30        40        50        60        70        80        90
str.Toulouse    MPVLTDPLQLLQALIRCPSVTPYEAGALSTLEQILTKMGFNVKRPVFTDKNTEDVENLYAKMGGEGRHLMFAGHTDVVPPGALEDWTYPPF
str.JK12        MPVLTDPLQLLQALIRCPSVTPYEAGALSTLEQILTKMGFNVKRPVFTDKNTEDVENLYAKMGGEGRHLMFAGHTDVVPPGALEDWTYPPF
str.CCUG45777   MPVLTDPLQLLQALIRCPSVTPYEAGALSTLEQILTKMGFNVKRPVFTDKNTEDVENLYAKMGGEGRHLMFAGHTDVVPPGALEDWTYPPF
str.JK56        MPVLTDPLQLLQALIRCPSVTPYEAGALSTLEQILTKMGFNVKRPVFTDKNTEDVENLYAKMGGEGRHLMFAGHTDVVPPGALEDWTYPPF
str.JK67        MPVLTDPLQLLQALIRCPSVTPYEAGALSTLEQILTKMGFNVKRPVFTDKNTEDVENLYAKMGGEGRHLMFAGHTDVVPPGALEDWTYPPF
str.JK19        MPVLTDPLQLLQALIRCPSVTPYEAGALSTLEQILTKMGFNVKRPVFTDKNTEDVENLYAKMGGEGRHLMFAGHTDVVPPGALEDWTYPPF
str.JK31        MPVLTDPLQLLQALIRCPSVTPYEAGALSTLEQILTKMGFNVKRPVFTDKNTEDVENLYAKMGGEGRHLMFAGHTDVVPPGALEDWTYPPF
str.JK68        MPVLTDPLQLLQALIRCPSVTPYEAGALSTLEQILTKMGFNVKRPVFTDKNTEDVENLYAKMGGEGRHLMFAGHTDVVPPGALEDWTYPPF
str.JK39        MPVLTDPLQLLQALIRCPSVTPYEAGALSTLEQILTKMGFNVKRPVFTDKNTEDVENLYAKMGGEGRHLMFAGHTDVVPPGALEDWTYPPF
str.JK63        MPVLTDPLQLLQALIRCPSVTPYEAGALSTLEQILTKMGFNVKRPVFTDKNTEDVENLYAKMGGEGRHLMFAGHTDVVPPGALEDWTYPPF
str.JK73        MPVLTDPLQLLQALIRCPSVTPYEAGALSTLEQILTKMGFNVKRPVFTDKNTEDVENLYAKMGGEGRHLMFAGHTDVVPPGALEDWTYPPF
str.G1712       MPVLTDPLQLLQALIRCPSVTPYEAGALSTLEQILTKMGFNVKRPVFTDKNTEDVENLYAKMGGEGRHLMFAGHTDVVPPGALEDWTYPPF
str.G1713       MPVLTDPLQLLQALIRCPSVTPYEAGALSTLEQILTKMGFNVKRPVFTDKNTEDVENLYAKMGGEGRHLMFAGHTDVVPPGALEDWTYPPF
str.CO20_0321   MPVLTDPLQLLQALIRCPSVTPYEAGALSTLEQILTKMGFNVKRPVFTDKNTEDVENLYAKMGGEGRHLMFAGHTDVVPPGALEDWTYPPF
str.BQ2-D70     MPVLTDPLQLLQALIRCPSVTPYEAGALSTLEQILTKMGFNVKRPVFTDKNTEDVENLYAKMGGEGRHLMFAGHTDVVPPGALEDWTYPPF
str.JK73rel     MPVLTDPLQLLQALIRCPSVTPYEAGALSTLEQILTKMGFNVKRPVFTDKNTEDVENLYAKMGGEGRHLMFAGHTDVVPPGALEDWTYPPF
str.JK7         MPVLTDPLQLLQALIRCPSVTPYEAGALSTLEQILTKMGFNVKRPVFTDKNTEDVENLYAKMGGEGRHLMFAGHTDVVPPGALEDWTYPPF
str.CO21_0024   MPVLTDPLQLLQALIRCPSVTPYEAGALSTLEQILTKMGFNVKRPVFTDKNTEDVENLYAKMGGEGRHLMFAGHTDVVPPGALEDWTYPPF
str.CO20_0297   MPVLTDPLQLLQALIRCPSVTPYEAGALSTLEQILTKMGFNVKRPVFTDKNTEDVENLYAKMGGEGRHLMFAGHTDVVPPGALEDWTYPPF
str.CO20_0256   MPVLTDPLQLLQALIRCPSVTPYEAGALSTLEQILTKMGFNVKRPVFTDKNTEDVENLYAKMGGEGRHLMFAGHTDVVPPGALEDWTYPPF
str.CO20_0257   MPVLTDPLQLLQALIRCPSVTPYEAGALSTLEQILTKMGFNVKRPVFTDKNTEDVENLYAKMGGEGRHLMFAGHTDVVPPGALEDWTYPPF
str.NCTC12899   MPVLTDPLQLLQALIRCPSVTPYEAGALSTLEQILTKMGFNVKRPVFTDKNTEDVENLYAKMGGEGRHLMFAGHTDVVPPGALEDWTYPPF
str.RM-11       MPVLTDPLQLLQALIRCPSVTPYEAGALSTLEQILTKMGFNVKRPVFTDKNTEDVENLYAKMGGEGRHLMFAGHTDVVPPGALEDWTYPPF
str.MF1-1       MPVLTDPLQLLQALIRCPSVTPYEAGALSTLEQILTKMGFNVKRPVFTDKNTEDVENLYAKMGGEGRHLMFAGHTDVVPPGALEDWTYPPF

                         100       110       120       130       140       150       160       170       180
str.Toulouse    EGVIDQGKLYGRGAVDMKGGIACFVAALARILEKRSIKGMVSLLITGDEEGPALNGTVKLLKWAEQKGEKWTAALVGEPTSVKTVGDVIKI
str.JK12        EGVIDQGKLYGRGAVDMKGGIACFVAALARILEKRSIKGMVSLLITGDEEGPALNGTVKLLKWAEQKGEKWTAALVGEPTSVKTVGDVIKI
str.CCUG45777   EGVIDQGKLYGRGAVDMKGGIACFVAALARILEKRSIKGMVSLLITGDEEGPALNGTVKLLKWAEQKGEKWTAALVGEPTSVKTVGDVIKI
str.JK56        EGVIDQGKLYGRGAVDMKGGIACFVAALARILEKRSIKGMVSLLITGDEEGPALNGTVKLLKWAEQKGEKWTAALVGEPTSVKTVGDVIKI
str.JK67        EGVIDQGKLYGRGAVDMKGGIACFVAALARILEKRSIKGMVSLLITGDEEGPALNGTVKLLKWAEQKGEKWTAALVGEPTSVKTVGDVIKI
str.JK19        EGVIDQGKLYGRGAVDMKGGIACFVAALARILEKRSIKGMVSLLITGDEEGPALNGTVKLLKWAEQKGEKWTAALVGEPTSVKTVGDVIKI
str.JK31        EGVIDQGKLYGRGAVDMKGGIACFVAALARILEKRSIKGMVSLLITGDEEGPALNGTVKLLKWAEQKGEKWTAALVGEPTSVKTVGDVIKI
str.JK68        EGVIDQGKLYGRGAVDMKGGIACFVAALARILEKRSIKGMVSLLITGDEEGPALNGTVKLLKWAEQKGEKWTAALVGEPTSVKTVGDVIKI
str.JK39        EGVIDQGKLYGRGAVDMKGGIACFVAALARILEKRSIKGMVSLLITGDEEGPALNGTVKLLKWAEQKGEKWTAALVGEPTSVKTVGDVIKI
str.JK63        EGVIDQGKLYGRGAVDMKGGIACFVAALARILEKRSIKGMVSLLITGDEEGPALNGTVKLLKWAEQKGEKWTAALVGEPTSVKTVGDVIKI
str.JK73        EGVIDQGKLYGRGAVDMKGGIACFVAALARILEKRSIKGMVSLLITGDEEGPALNGTVKLLKWAEQKGEKWTAALVGEPTSVKTVGDVIKI
str.G1712       EGVIDQGKLYGRGAVDMKGGIACFVAALARILEKRSIKGMVSLLITGDEEGPALNGTVKLLKWAEQKGEKWTAALVGEPTSVKTVGDVIKI
str.G1713       EGVIDQGKLYGRGAVDMKGGIACFVAALARILEKRSIKGMVSLLITGDEEGPALNGTVKLLKWAEQKGEKWTAALVGEPTSVKTVGDVIKI
str.CO20_0321   EGVIDQGKLYGRGAVDMKGGIACFVAALARILEKRSIKGMVSLLITGDEEGPALNGTVKLLKWAEQKGEKWTAALVGEPTSVKTVGDVIKI
str.BQ2-D70     EGVIDQGKLYGRGAVDMKGGIACFVAALARILEKRSIKGMVSLLITGDEEGPALNGTVKLLKWAEQKGEKWTAALVGEPTSVKTVGDVIKI
str.JK73rel     EGVIDQGKLYGRGAVDMKGGIACFVAALARILEKRSIKGMVSLLITGDEEGPALNGTVKLLKWAEQKGEKWTAALVGEPTSVKTVGDVIKI
str.JK7         EGVIDQGKLYGRGAVDMKGGIACFVAALARILEKRSIKGMVSLLITGDEEGPALNGTVKLLKWAEQKGEKWTAALVGEPTSVKTVGDVIKI
str.CO21_0024   EGVIDQGKLYGRGAVDMKGGIACFVAALARILEKRSIKGMVSLLITGDEEGPALNGTVKLLKWAEQKGEKWTAALVGEPTSVKTVGDVIKI
str.CO20_0297   EGVIDQGKLYGRGAVDMKGGIACFVAALARILEKRSIKGMVSLLITGDEEGPALNGTVKLLKWAEQKGEKWTAALVGEPTSVKTVGDVIKI
str.CO20_0256   EGVIDQGKLYGRGAVDMKGGIACFVAALARILEKRSIKGMVSLLITGDEEGPALNGTVKLLKWAEQKGEKWTAALVGEPTSVKTVGDVIKI
str.CO20_0257   EGVIDQGKLYGRGAVDMKGGIACFVAALARILEKRSIKGMVSLLITGDEEGPALNGTVKLLKWAEQKGEKWTAALVGEPTSVKTVGDVIKI
str.NCTC12899   EGVIDQGKLYGRGAVDMKGGIACFVAALARILEKRSIKGMVSLLITGDEEGPALNGTVKLLKWAEQKGEKWTAALVGEPTSVKTVGDVIKI
str.RM-11       AGVIDQGKLYGRGAVDMKGAIACFVAALARILEKRSIKGMVSLLITGDEEGPALNGTVKLLKWAEQKGEKWTAALVGEPTSVKTVGDVIKI
str.MF1-1       AGVIDQGKLYGRGAVDMKGAIACFVAALARILEKRSIKGMVSLLITGDEEGPALNGTVKLLKWAEQKGEKWTAALVGEPTSVKTVGDVIKI

                        190       200       210       220       230       240       250       260       270
str.Toulouse    GRRGSLSGVVTVKGRQGHVAFPERAANPLPLAGKLIQALTQTALDRGTENFQPSNLELTTIDTDNPAVNVIPAQTTIRFNIRYNDVWTKET
str.JK12        GRRGSLSGVVTVKGRQGHVAFPERAANPLPLAGKLIQALTQTALDRGTENFQPSNLELTTIDTDNPAVNVIPAQTTIRFNIRYNDVWTKET
str.CCUG45777   GRRGSLSGVVTVKGRQGHVAFPERAANPLPLAGKLIQALTQTALDRGTENFQPSNLELTTIDTDNPAVNVIPAQTTIRFNIRYNDVWTKET
str.JK56        GRRGSLSGVVTVKGRQGHVAFPERAANPLPLAGKLIQALTQTALDRGTENFQPSNLELTTIDTDNPAVNVIPAQTTIRFNIRYNDVWTKET
str.JK67        GRRGSLSGVVTVKGRQGHVAFPERAANPLPLAGKLIQALTQTALDRGTENFQPSNLELTTIDTDNPAVNVIPAQTTIRFNIRYNDVWTKET
str.JK19        GRRGSLSGVVTVKGRQGHVAFPERAANPLPLAGKLIQALTQTALDRGTENFQPSNLELTTIDTDNPAVNVIPAQTTIRFNIRYNDVWTKET
str.JK31        GRRGSLSGVVTVKGRQGHVAFPERAANPLPLAGKLIQALTQTALDRGTENFQPSNLELTTIDTDNPAVNVIPAQTTIRFNIRYNDVWTKET
str.JK68        GRRGSLSGVVTVKGRQGHVAFPERAANPLPLAGKLIQALTQTALDRGTENFQPSNLELTTIDTDNPAVNVIPAQTTIRFNIRYNDVWTKET
str.JK39        GRRGSLSGVVTVKGRQGHVAFPERAANPLPLAGKLIQALTQTALDRGTENFQPSNLELTTIDTDNPAVNVIPAQTTIRFNIRYNDVWTKET
str.JK63        GRRGSLSGVVTVKGRQGHVAFPERAANPLPLAGKLIQALTQTALDRGTENFQPSNLELTTIDTDNPAVNVIPAQTTIRFNIRYNDVWTKET
str.JK73        GRRGSLSGVVTVKGRQGHVAFPERAANPLPLAGKLIQALTQTALDRGTENFQPSNLELTTIDTDNPAVNVIPAQTTIRFNIRYNDVWTKET
str.G1712       GRRGSLSGVVTVKGRQGHVAFPERAANPLPLAGKLIQALTQTALDRGTENFQPSNLELTTIDTDNPAVNVIPAQTTIRFNIRYNDVWTKET
str.G1713       GRRGSLSGVVTVKGRQGHVAFPERAANPLPLAGKLIQALTQTALDRGTENFQPSNLELTTIDTDNPAVNVIPAQTTIRFNIRYNDVWTKET
str.CO20_0321   GRRGSLSGVVTVKGRQGHVAFPERAANPLPLAGKLIQALTQTALDRGTENFQPSNLELTTIDTDNPAVNVIPAQTTIRFNIRYNDVWTKET
str.BQ2-D70     GRRGSLSGVVTVKGRQGHVAFPERAANPLPLAGKLIQALTQTALDRGTENFQPSNLELTTIDTDNPAVNVIPAQTTIRFNIRYNDVWTKET
str.JK73rel     GRRGSLSGVVTVKGRQGHVAFPERAANPLPLAGKLIQALTQTALDRGTENFQPSNLELTTIDTDNPAVNVIPAQTTIRFNIRYNDVWTKET
str.JK7         GRRGSLSGVVTVKGRQGHVAFPERAANPLPLAGKLIQALTQTALDRGTENFQPSNLELTTIDTDNPAVNVIPAQTTIRFNIRYNDVWTKET
str.CO21_0024   GRRGSLSGVVTVKGRQGHVAFPERAANPLPLAGKLIQALTQTALDRGTENFQPSNLELTTIDTDNPAVNVIPAQTTIRFNIRYNDVWTKET
str.CO20_0297   GRRGSLSGVVTVKGRQGHVAFPERAANPLPLAGKLIQALTQTALDRGTENFQPSNLELTTIDTDNPAVNVIPAQTTIRFNIRYNDVWTKET
str.CO20_0256   GRRGSLSGVVTVKGRQGHVAFPERAANPLPLAGKLIQALTQTALDRGTENFQPSNLELTTIDTDNPAVNVIPAQTTIRFNIRYNDVWTKET
str.CO20_0257   GRRGSLSGVVTVKGRQGHVAFPERAANPLPLAGKLIQALTQTALDRGTENFQPSNLELTTIDTDNPAVNVIPAQTTIRFNIRYNDVWTKET
str.NCTC12899   GRRGSLSGVVTVKGRQGHVAFPERAANPLPLAGKLIQALTQTALDRGTENFQPSNLELTTIDTDNPAVNVIPAQTTIRFNIRYNDVWTKET
str.RM-11       GRRGSLSGVVTVKGRQGHVAFPERAANPLPLAGKLIQALTRTALDRGTENFQPSNLELTTIDTDNPAVNVIPAQTTIRFNIRYNDVWTKET
str.MF1-1       GRRGSLSGVVTVKGRQGHVAFPERAVNPLPLAGKLIQALTRTALDRGTENFQPSNLELTTIDTDNPVVNVIPAQTTIRFNIRYNDVWTKET

                   280       290       300       310       320       330       340       350       360
str.Toulouse    LMTEIEKRLASVQLKNNDYQYPYYQLEWIPSLGSVFITKNDKLIKTLSNAIESVTGNIPEYSTSGGTSDARFIKDYCPVVEFGLPGQTMHM
str.JK12        LMTEIEKRLASVQLKNNDYQYPYYQLEWIPSLGSVFITKNDKLIKTLSNAIESVTGNIPEYSTSGGTSDARFIKDYCPVVEFGLPGQTMHM
str.CCUG45777   LMTEIEKRLASVQLKNNDYQYPYYQLEWIPSLGSVFITKNDKLIKTLSNAIESVTGNIPEYSTSGGTSDARFIKDYCPVVEFGLPGQTMHM
str.JK56        LMTEIEKRLASVQLKNNDYQYPYYQLEWIPSLGSVFITKNDKLIKTLSNAIESVTGNIPEYSTSGGTSDARFIKDYCPVVEFGLPGQTMHM
str.JK67        LMTEIEKRLASVQLKNNDYQYPYYQLEWIPSLGSVFITKNDKLIKTLSNAIESVTGNIPEYSTSGGTSDARFIKDYCPVVEFGLPGQTMHM
str.JK19        LMTEIEKRLASVQLKNNDYQYPYYQLEWIPSLGSVFITKNDKLIKTLSNAIESVTGNIPEYSTSGGTSDARFIKDYCPVVEFGLPGQTMHM
str.JK31        LMTEIEKRLASVQLKNNDYQYPYYQLEWIPSLGSVFITKNDKLIKTLSNAIESVTGNIPEYSTSGGTSDARFIKDYCPVVEFGLPGQTMHM
str.JK68        LMTEIEKRLASVQLKNNDYQYPYYQLEWIPSLGSVFITKNDKLIKTLSNAIESVTGNIPEYSTSGGTSDARFIKDYCPVVEFGLPGQTMHM
str.JK39        LMTEIEKRLASVQLKNNDYQYPYYQLEWIPSLGSVFITKNDKLIKTLSNAIESVTGNIPEYSTSGGTSDARFIKDYCPVVEFGLPGQTMHM
str.JK63        LMTEIEKRLASVQLKNNDYQYPYYQLEWIPSLGSVFITKNDKLIKTLSNAIESVTGNIPEYSTSGGTSDARFIKDYCPVVEFGLPGQTMHM
str.JK73        LMTEIEKRLASVQLKNNDYQYPYYQLEWIPSLGSVFITKNDKLIKTLSNAIESVTGNIPEYSTSGGTSDARFIKDYCPVVEFGLPGQTMHM
str.G1712       LMTEIEKRLASVQLKNNDYQYPYYQLEWIPSLGSVFITKNDKLIKTLSNAIESVTGNIPEYSTSGGTSDARFIKDYCPVVEFGLPGQTMHM
str.G1713       LMTEIEKRLASVQLKNNDYQYPYYQLEWIPSLGSVFITKNDKLIKTLSNAIESVTGNIPEYSTSGGTSDARFIKDYCPVVEFGLPGQTMHM
str.CO20_0321   LMTEIEKRLASVQLKNNDYQYPYYQLEWIPSLGSVFITKNDKLIKTLSNAIESVTGNIPEYSTSGGTSDARFIKDYCPVVEFGLPGQTMHM
str.BQ2-D70     LMTEIEKRLASVQLKNNDYQYPYYQLEWIPSLGSVFITKNDKLIKTLSNAIESVTGNIPEYSTSGGTSDARFIKDYCPVVEFGLPGQTMHM
str.JK73rel     LMTEIEKRLASVQLKNNDYQYPYYQLEWIPSLGSVFITKNDKLIKTLSNAIESVTGNIPEYSTSGGTSDARFIKDYCPVVEFGLPGQTMHM
str.JK7         LMTEIEKRLASVQLKNNDYQYPYYQLEWIPSLGSVFITKNDKLIKTLSNAIESVTGNIPEYSTSGGTSDARFIKDYCPVVEFGLPGQTMHM
str.CO21_0024   LMTEIEKRLASVQLKNNDYQYPYYQLEWIPSLGSVFITKNDKLIKTLSNAIESVTGNIPEYSTSGGTSDARFIKDYCPVVEFGLPGQTMHM
str.CO20_0297   LMTEIEKRLASVQLKNNDYQYPYYQLEWIPSLGSVFITKNDKLIKTLSNAIESVTGNIPEYSTSGGTSDARFIKDYCPVVEFGLPGQTMHM
str.CO20_0256   LMTEIEKRLASVQLKNNDYQYPYYQLEWIPSLGSVFITKNDKLIKTLSNAIESVTGNIPEYSTSGGTSDARFIKDYCPVVEFGLPGQTMHM
str.CO20_0257   LMTEIEKRLASVQLKNNDYQYPYYQLEWIPSLGSVFITKNDKLIKTLSNAIESVTGNIPEYSTSGGTSDARFIKDYCPVVEFGLPGQTMHM
str.NCTC12899   LMTEIEKRLASVQLKNNDYQYPYYQLEWIPSLGSVFITKNDKLIKTLSNAIESVTGNIPEYSTSGGTSDARFIKDYCPVVEFGLPGQTMHM
str.RM-11       LMTEIEKRLASVQLKNNDDQYPYYQLEWIPSLGSVFITKNDKLIKILSNAIESVTGNIPEYSTSGGTSDARFIKDYCPVVEFGLPGQTMHM
str.MF1-1       LMAEIEKRLASVQLKNNDDQYPYYQLEWIPSLGSVFITKNDKLIKILSNAIESVTGNIPEYSTSGGTSDARFIKDYCPVVEFGLPGQTMHM
```

CC BY

	370 380 390
str.Toulouse	VDECVTLDAIETLTSVYERFIVDFFA
str.JK12	VDECVTLDAIETLTSVYERFIVDFFA
str.CCUG45777	VDECVTLDAIETLTSVYERFIVDFFA
str.JK56	VDECVTLDAIETLTSVYERFIVDFFA
str.JK67	VDECVTLDAIETLTSVYERFIVDFFA
str.JK19	VDECVTLDAIETLTSVYERFIVDFFA
str.JK31	VDECVTLDAIETLTSVYERFIVDFFA
str.JK68	VDECVTLDAIETLTSVYERFIVDFFA
str.JK39	VDECVTLDAIETLTSVYERFIVDFFA
str.JK63	VDECVTLDAIETLTSVYERFIVDFFA
str.JK73	VDECVTLDAIETLTSVYERFIVDFFA
str.G1712	VDECVTLDAIETLTSVYERFIVDFFA
str.G1713	VDECVTLDAIETLTSVYERFIVDFFA
str.CO20_0321	VDECVTLDAIETLTSVYERFIVDFFA
str.BQ2-D70	VDECVTLDAIETLTSVYERFIVDFFA
str.JK73rel	VDECVTLDAIETLTSVYERFIVDFFA
str.JK7	VDECVTLDAIETLTSVYERFIVDFFA
str.CO21_0024	VDECVTLDAIETLTSVYERFIVDFFA
str.CO20_0297	VDECVTLDAIETLTSVYERFIVDFFA
str.CO20_0256	VDECVTLDAIETLTSVYERFIVDFFA
str.CO20_0257	VDECVTLDAIETLTSVYERFIVDFFA
str.NCTC12899	VDECVTLDAIETLTSVYERFIVDFFA
str.RM-11	VDECVTLDAIETLTSVYERFIVDFFA
str.MF1-1	VDECVTLDAIETLTSVYERFIVDFFA

>WP_011178891.1 response regulator transcription factor [Bartonella quintana str. Toulouse]

```
               1         10        20        30        40        50        60        70        80        90
str.Toulouse   MKDTPTITQTIVLVDDDRNILTSLSFALETEGYRVESYTDGASALKNLTLHPPHLAIFDIKMPRMDGMELLRRLRQKSDIPVIFLTSKDDE
str.CCUG45777  MKDTPTITQTIVLVDDDRNILTSLSFALETEGYRVESYTDGASALKNLTLHPPHLAIFDIKMPRMDGMELLRRLRQKSDIPVIFLTSKDDE
str.JK12       MKDTPTITQTIVLVDDDRNILTSLSFALETEGYRVESYTDGASALKNLTLHPPHLAIFDIKMPRMDGMELLRRLRQKSDIPVIFLTSKDDE
str.JK67       MKDTPTITQTIVLVDDDRNILTSLSFALETEGYRVESYTDGASALKNLTLHPPHLAIFDIKMPRMDGMELLRRLRQKSDIPVIFLTSKDDE
str.JK56       MKDTPTITQTIVLVDDDRNILTSLSFALETEGYRVESYTDGASALKNLTLHPPHLAIFDIKMPRMDGMELLRRLRQKSDIPVIFLTSKDDE
str.BQ2-D70    MKDTPTITQTIVLVDDDRNILTSLSFALETEGYRVESYTDGASALKNLTLHPPHLAIFDIKMPRMDGMELLRRLRQKSDIPVIFLTSKDDE
str.JK19       MKDTPTITQTIVLVDDDRNILTSLSFALETEGYRVESYTDGASALKNLTLHPPHLAIFDIKMPRMDGMELLRRLRQKSDIPVIFLTSKDDE
str.JK7        MKDTPTITQTIVLVDDDRNILTSLSFALETEGYRVESYTDGASALKNLTLHPPHLAIFDIKMPRMDGMELLRRLRQKSDIPVIFLTSKDDE
str.JK73rel    MKDTPTITQTIVLVDDDRNILTSLSFALETEGYRVESYTDGASALKNLTLHPPHLAIFDIKMPRMDGMELLRRLRQKSDIPVIFLTSKDDE
str.JK63       MKDTPTITQTIVLVDDDRNILTSLSFALETEGYRVESYTDGASALKNLTLHPPHLAIFDIKMPRMDGMELLRRLRQKSDIPVIFLTSKDDE
str.JK73       MKDTPTITQTIVLVDDDRNILTSLSFALETEGYRVESYTDGASALKNLTLHPPHLAIFDIKMPRMDGMELLRRLRQKSDIPVIFLTSKDDE
str.JK68       MKDTPTITQTIVLVDDDRNILTSLSFALETEGYRVESYTDGASALKNLTLHPPHLAIFDIKMPRMDGMELLRRLRQKSDIPVIFLTSKDDE
str.JK39       MKDTPTITQTIVLVDDDRNILTSLSFALETEGYRVESYTDGASALKNLTLHPPHLAIFDIKMPRMDGMELLRRLRQKSDIPVIFLTSKDDE
str.NCTC12899  MKDTPTITQTIVLVDDDRNILTSLSFALETEGYRVESYTDGASALKNLTLHPPHLAIFDIKMPRMDGMELLRRLRQKSDIPVIFLTSKDDE
str.JK31       MKDTPTITQTIVLVDDDRNILTSLSFALETEGYRVESYTDGASALKNLTLHPPHLAIFDIKMPRMDGMELLRRLRQKSDIPVIFLTSKDDE
str.CO20_0256  MKDTPTITQTIVLVDDDRNILTSLSFALETEGYRVESYTDGASALKNLTLHPPHLAIFDIKMPRMDGMELLRRLRQKSDIPVIFLTSKDDE
str.CO20_0321  MKDTPTITQTIVLVDDDRNILTSLSFALETEGYRVESYTDGASALKNLTLHPPHLAIFDIKMPRMDGMELLRRLRQKSDIPVIFLTSKDDE
str.CO20_0257  MKDTPTITQTIVLVDDDRNILTSLSFALETEGYRVESYTDGASALKNLTLHPPHLAIFDIKMPRMDGMELLRRLRQKSDIPVIFLTSKDDE
str.CO21_0024  MKDTPTITQTIVLVDDDRNILTSLSFALETEGYRVESYTDGASALKNLTLHPPHLAIFDIKMPRMDGMELLRRLRQKSDIPVIFLTSKDDE
str.CO20_0297  MKDTPTITQTIVLVDDDRNILTSLSFALETEGYRVESYTDGASALKNLTLHPPHLAIFDIKMPRMDGMELLRRLRQKSDIPVIFLTSKDDE
str.RM-11      MKDTPTITQTIVLVDDDRNILTSLSFALETEGYRVESYTDGASALKNLTLHPPHLAIFDIKMPRMDGMELLRRLRQKSDIPVIFLTSKDDE
str.MF1-1      MKDTPTITQTIVLVDDDRNILTSLSFALETEGYRVESYTDGASALKNLTLHPPHLAIFDIKMPRMDGMELLRRLRQKSDIPVIFLTSKDDE
str.G1712      ...........................................................................................
str.G1713      MKDTPTITQTIVLVDDDRNILTSLSFALETEGYRVESYTDGASALKNLTLHPPHLAIFDIKMPRMDGMELLRRLRQKSDIPVIFLTSKDDE

                      100       110       120       130       140       150       160       170       180
str.Toulouse   IDELFGLKMGADDFITKPFSQRLLIERVKAILRRANARNQPLATGASSTSSLKRGDLVMDQERHTCTWKDKPVILTVTEFLILQTLAQRPG
str.CCUG45777  IDELFGLKMGADDFITKPFSQRLLIERVKAILRRANARNQPLATGASSTSSLKRGDLVMDQERHTCTWKDKPVILTVTEFLILQTLAQRPG
str.JK12       IDELFGLKMGADDFITKPFSQRLLIERVKAILRRANARNQPLATGASSTSSLKRGDLVMDQERHTCTWKDKPVILTVTEFLILQTLAQRPG
str.JK67       IDELFGLKMGADDFITKPFSQRLLIERVKAILRRANARNQPLATGASSTSSLKRGDLVMDQERHTCTWKDKPVILTVTEFLILQTLAQRPG
str.JK56       IDELFGLKMGADDFITKPFSQRLLIERVKAILRRANARNQPLATGASSTSSLKRGDLVMDQERHTCTWKDKPVILTVTEFLILQTLAQRPG
str.BQ2-D70    IDELFGLKMGADDFITKPFSQRLLIERVKAILRRANARNQPLATGASSTSSLKRGDLVMDQERHTCTWKDKPVILTVTEFLILQTLAQRPG
str.JK19       IDELFGLKMGADDFITKPFSQRLLIERVKAILRRANARNQPLATGASSTSSLKRGDLVMDQERHTCTWKDKPVILTVTEFLILQTLAQRPG
str.JK7        IDELFGLKMGADDFITKPFSQRLLIERVKAILRRANARNQPLATGASSTSSLKRGDLVMDQERHTCTWKDKPVILTVTEFLILQTLAQRPG
str.JK73rel    IDELFGLKMGADDFITKPFSQRLLIERVKAILRRANARNQPLATGASSTSSLKRGDLVMDQERHTCTWKDKPVILTVTEFLILQTLAQRPG
str.JK63       IDELFGLKMGADDFITKPFSQRLLIERVKAILRRANARNQPLATGASSTSSLKRGDLVMDQERHTCTWKDKPVILTVTEFLILQTLAQRPG
str.JK73       IDELFGLKMGADDFITKPFSQRLLIERVKAILRRANARNQPLATGASSTSSLKRGDLVMDQERHTCTWKDKPVILTVTEFLILQTLAQRPG
str.JK68       IDELFGLKMGADDFITKPFSQRLLIERVKAILRRANARNQPLATGASSTSSLKRGDLVMDQERHTCTWKDKPVILTVTEFLILQTLAQRPG
str.JK39       IDELFGLKMGADDFITKPFSQRLLIERVKAILRRANARNQPLATGASSTSSLKRGDLVMDQERHTCTWKDKPVILTVTEFLILQTLAQRPG
str.NCTC12899  IDELFGLKMGADDFITKPFSQRLLIERVKAILRRANARNQPLATGASSTSSLKRGDLVMDQERHTCTWKDKPVILTVTEFLILQTLAQRPG
str.JK31       IDELFGLKMGADDFITKPFSQRLLIERVKAILRRANARNQPLATGASSTSSLKRGDLVMDQERHTCTWKDKPVILTVTEFLILQTLAQRPG
str.CO20_0256  IDELFGLKMGADDFITKPFSQRLLIERVKAILRRANARNQPLATGASSTSSLKRGDLVMDQERHTCTWKDKPVILTVTEFLILQTLAQRPG
str.CO20_0321  IDELFGLKMGADDFITKPFSQRLLIERVKAILRRANARNQPLATGASSTSSLKRGDLVMDQERHTCTWKDKPVILTVTEFLILQTLAQRPG
str.CO20_0257  IDELFGLKMGADDFITKPFSQRLLIERVKAILRRANARNQPLATGASSTSSLKRGDLVMDQERHTCTWKDKPVILTVTEFLILQTLAQRPG
str.CO21_0024  IDELFGLKMGADDFITKPFSQRLLIERVKAILRRANARNQPLATGASSTSSLKRGDLVMDQERHTCTWKDKPVILTVTEFLILQTLAQRPG
str.CO20_0297  IDELFGLKMGADDFITKPFSQRLLIERVKAILRRANARNQPLATGASSTSSLKRGDLVMDQERHTCTWKDKPVILTVTEFLILQTLAQRPG
str.RM-11      IDELFGLKMGADDFITKPFSQRLLIERVKAILRRANARNQPLATGASSTSSLKRGDLVMDQERHTCTWKDKPVILTVTEFLILQTLAQRPG
str.MF1-1      IDELFGLKMGADDFITKPFSQRLLIERVKAILRRANARNQPLATGASSTSSLKRGDLVMDQERHTCTWKDKPVILTVTEFLILQTLAQRPG
str.G1712      ...............................LRRANARNQPLATGASSTSSLKRGDLVMDQERHTCTWKDKPVILTVTEFLILQTLAQRPG
str.G1713      IDELFGLKMGADDFITKPFSQRLLIERVKAILRRANARNQPLATGASSTSSLKRGDLVMDQERHTCTWKDKPVILTVTEFLILQTLAQRPG

                    190       200       210       220       230       240
str.Toulouse   VVKSRDALMDAAYSDQVYVDDRTIDSHIKRLRKKFKQVDDDFAMIETLYGVGYRFHEV
str.CCUG45777  VVKSRDALMDAAYSDQVYVDDRTIDSHIKRLRKKFKQVDDDFAMIETLYGVGYRFHEV
str.JK12       VVKSRDALMDAAYSDQVYVDDRTIDSHIKRLRKKFKQVDDDFAMIETLYGVGYRFHEV
str.JK67       VVKSRDALMDAAYSDQVYVDDRTIDSHIKRLRKKFKQVDDDFAMIETLYGVGYRFHEV
str.JK56       VVKSRDALMDAAYSDQVYVDDRTIDSHIKRLRKKFKQVDDDFAMIETLYGVGYRFHEV
str.BQ2-D70    VVKSRDALMDAAYSDQVYVDDRTIDSHIKRLRKKFKQVDDDFAMIETLYGVGYRFHEV
str.JK19       VVKSRDALMDAAYSDQVYVDDRTIDSHIKRLRKKFKQVDDDFAMIETLYGVGYRFHEV
str.JK7        VVKSRDALMDAAYSDQVYVDDRTIDSHIKRLRKKFKQVDDDFAMIETLYGVGYRFHEV
str.JK73rel    VVKSRDALMDAAYSDQVYVDDRTIDSHIKRLRKKFKQVDDDFAMIETLYGVGYRFHEV
str.JK63       VVKSRDALMDAAYSDQVYVDDRTIDSHIKRLRKKFKQVDDDFAMIETLYGVGYRFHEV
str.JK73       VVKSRDALMDAAYSDQVYVDDRTIDSHIKRLRKKFKQVDDDFAMIETLYGVGYRFHEV
str.JK68       VVKSRDALMDAAYSDQVYVDDRTIDSHIKRLRKKFKQVDDDFAMIETLYGVGYRFHEV
str.JK39       VVKSRDALMDAAYSDQVYVDDRTIDSHIKRLRKKFKQVDDDFAMIETLYGVGYRFHEV
str.NCTC12899  VVKSRDALMDAAYSDQVYVDDRTIDSHIKRLRKKFKQVDDDFAMIETLYGVGYRFHEV
str.JK31       VVKSRDALMDAAYSDQVYVDDRTIDSHIKRLRKKFKQVDDDFAMIETLYGVGYRFHEV
str.CO20_0256  VVKSRDALMDAAYSDQVYVDDRTIDSHIKRLRKKFKQVDDDFAMIETLYGVGYRFHEV
str.CO20_0321  VVKSRDALMDAAYSDQVYVDDRTIDSHIKRLRKKFKQVDDDFAMIETLYGVGYRFHEV
str.CO20_0257  VVKSRDALMDAAYSDQVYVDDRTIDSHIKRLRKKFKQVDDDFAMIETLYGVGYRFHEV
str.CO21_0024  VVKSRDALMDAAYSDQVYVDDRTIDSHIKRLRKKFKQVDDDFAMIETLYGVGYRFHEV
str.CO20_0297  VVKSRDALMDAAYSDQVYVDDRTIDSHIKRLRKKFKQVDDDFAMIETLYGVGYRFHEV
str.RM-11      VVKSRDALMDAAYSDQVYVDDRTIDSHIKRLRKKFKQVDDDFAMIETLYGVGYRFHEV
str.MF1-1      VVKSRDALMDAAYSDQVYVDDRTIDSHIKRLRKKFKQVDDDFAMIETLYGVGYRFHEV
str.G1712      VVKSRDALMDAAYSDQVYVDDRTIDSHIKRLRKKFKQVDDDFAMIETLYGVGYRFHEV
str.G1713      VVKSRDALMDAAYSDQVYVDDRTIDSHIKRLRKKFKQVDDDFAMIETLYGVGYRFHEV
```

>WP_011178913.1 metal ABC transporter substrate-binding protein [Bartonella quintana str. Toulouse]

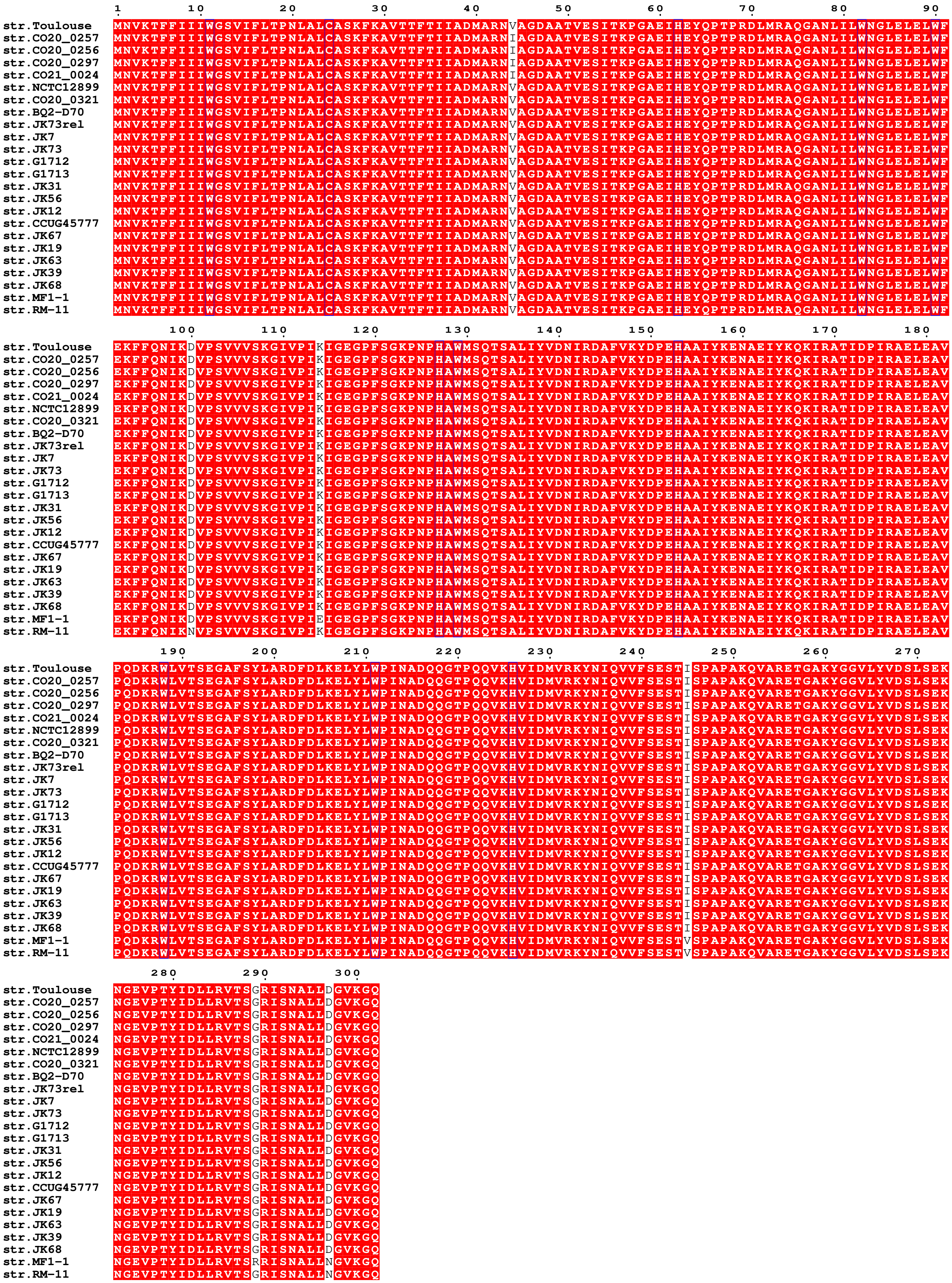

>WP_011178939.1 chromosomal replication initiator protein DnaA [Bartonella quintana str. Toulouse]

```
                 1         10        20        30        40        50        60        70        80        90
str.Toulouse     MVDKLNSKASSFKRVSVDILSAEKIIKSKMTNIGGPVVENASIEHPVISEEEGAAAFARVMAQLKAQVGIEAYTSWFGRLKLAEYSRNLVK
str.RM-11        MVDKLNSKASSFKRVSVDILSAEKIIKSKMTNIGGPVVENASIEHPVISEEEGAAAFARVMAQLKAQVGIEAYTSWFGRLKLAEYSRNLVK
str.MF1-1        MVDKLNSKASSFKRVSVDILSAEKIIKSKMTNIGGPVVENASIEHPVISEEEGAATFARVMAQLKAQVGIEAYTSWFGRLKLAEYSRNLVK
str.NCTC12899    MVDKLNSKASSFKRVSVDILSAEKIIKSKMTNIGGPVVENASIEHPVISEEEGAAAFARVMAQLKAQVGIEAYTSWFGRLKLAEYSRNLVK
str.CCUG45777    MVDKLNSKASSFKRVSVDILSAEKIIKSKMTNIGGPVVENASIEHPVISEEEGAAAFARVMAQLKAQVGIEAYTSWFGRLKLAEYSRNLVK
str.JK12         MVDKLNSKASSFKRVSVDILSAEKIIKSKMTNIGGPVVENASIEHPVISEEEGAAAFARVMAQLKAQVGIEAYTSWFGRLKLAEYSRNLVK
str.JK56         MVDKLNSKASSFKRVSVDILSAEKIIKSKMTNIGGPVVENASIEHPVISEEEGAAAFARVMAQLKAQVGIEAYTSWFGRLKLAEYSRNLVK
str.JK67         MVDKLNSKASSFKRVSVDILSAEKIIKSKMTNIGGPVVENASIEHPVISEEEGAAAFARVMAQLKAQVGIEAYTSWFGRLKLAEYSRNLVK
str.JK19         MVDKLNSKASSFKRVSVDILSAEKIIKSKMTNIGGPVVENASIEHPVISEEEGAAAFARVMAQLKAQVGIEAYTSWFGRLKLAEYSRNLVK
str.JK63         MVDKLNSKASSFKRVSVDILSAEKIIKSKMTNIGGPVVENASIEHPVISEEEGAAAFARVMAQLKAQVGIEAYTSWFGRLKLAEYSRNLVK
str.JK39         MVDKLNSKASSFKRVSVDILSAEKIIKSKMTNIGGPVVENASIEHPVISEEEGAAAFARVMAQLKAQVGIEAYTSWFGRLKLAEYSRNLVK
str.JK68         MVDKLNSKASSFKRVSVDILSAEKIIKSKMTNIGGPVVENASIEHPVISEEEGAAAFARVMAQLKAQVGIEAYTSWFGRLKLAEYSRNLVK
str.JK31         MVDKLNSKASSFKRVSVDILSAEKIIKSKMTNIGGPVVENASIEHPVISEEEGAAAFARVMAQLKAQVGIEAYTSWFGRLKLAEYSRNLVK
str.JK73         MVDKLNSKASSFKRVSVDILSAEKIIKSKMTNIGGPVVENASIEHPVISEEEGAAAFARVMAQLKAQVGIEAYTSWFGRLKLAEYSRNLVK
str.JK7          MVDKLNSKASSFKRVSVDILSAEKIIKSKMTNIGGPVVENASIEHPVISEEEGAAAFARVMAQLKAQVGIEAYTSWFGRLKLAEYSRNLVK
str.JK73rel      MVDKLNSKASSFKRVSVDILSAEKIIKSKMTNIGGPVVENASIEHPVISEEEGAAAFARVMAQLKAQVGIEAYTSWFGRLKLAEYSRNLVK
str.BQ2-D70      MVDKLNSKASSFKRVSVDILSAEKIIKSKMTNIGGPVVENASIEHPVISEEEGAAAFARVMAQLKAQVGIEAYTSWFGRLKLAEYSRNLVK
str.CO20_0321    MVDKLNSKASSFKRVSVDILSAEKIIKSKMTNIGGPVVENASIEHPVISEEEGAAAFARVMAQLKAQVGIEAYTSWFGRLKLAEYSRNLVK
str.CO21_0024    MVDKLNSKASSFKRVSVDILSAEKIIKSKMTNIGGPVVENASIEHPVISEEEGAAAFARVMAQLKAQVGIEAYTSWFGRLKLAEYSRNLVK
str.CO20_0297    MVDKLNSKASSFKRVSVDILSAEKIIKSKMTNIGGPVVENASIEHPVISEEEGAAAFARVMAQLKAQVGIEAYTSWFGRLKLAEYSRNLVK
str.CO20_0256    MVDKLNSKASSFKRVSVDILSAEKIIKSKMTNIGGPVVENASIEHPVISEEEGAAAFARVMAQLKAQVGIEAYTSWFGRLKLAEYSRNLVK
str.CO20_0257    MVDKLNSKASSFKRVSVDILSAEKIIKSKMTNIGGPVVENASIEHPVISEEEGAAAFARVMAQLKAQVGIEAYTSWFGRLKLAEYSRNLVK
str.G1712        .............................MTNIGGPVVENASIEHPVISEEEGAAAFARVMAQLKAQVGIEAYTSWFGRLKLAEYSRNLVK
str.G1713        .............................MTNIGGPVVENASIEHPVISEEEGAAAFARVMAQLKAQVGIEAYTSWFGRLKLAEYSRNLVK

                     100       110       120       130       140       150       160       170       180
str.Toulouse     LSVPTAFLRSWINNHYGSLLTNLWKQENSAILRVEVIVRGMKRVSKGVVCRTSAAPVVLEGQTASSFVESYTEPSVKDIEAGVFGSPLDSR
str.RM-11        LSVPTAFLRSWINNHYGSLLTNLWKQENSAILRVEVIVRGMKRVSKGVVCRTSAAPVVLEGQTASSFVESYTEPSVKDIEAGVFGSPLDSR
str.MF1-1        LSVPTAFLRSWINNHYGSLLTNLWKQENSAILRVEVIVRGMKRVSKGVVCRTSAAPVVLEGQTASSFVESYTEPSVKDIEAGVFGSPLDSR
str.NCTC12899    LSVPTAFLRSWINNHYGSLLTNLWKQENSAILRVEVIVRGMKRVSKGVVCRTSAAPVVLEGQTASSFVESYTEPSVKDIEAGVFGSPLDSR
str.CCUG45777    LSVPTAFLRSWINNHYGSLLTNLWKQENSAILRVEVIVRGMKRVSKGVVCRTSAAPVVLEGQTASSFVESYTEPSVKDIEAGVFGSPLDSR
str.JK12         LSVPTAFLRSWINNHYGSLLTNLWKQENSAILRVEVIVRGMKRVSKGVVCRTSAAPVVLEGQTASSFVESYTEPSVKDIEAGVFGSPLDSR
str.JK56         LSVPTAFLRSWINNHYGSLLTNLWKQENSAILRVEVIVRGMKRVSKGVVCRTSAAPVVLEGQTASSFVESYTEPSVKDIEAGVFGSPLDSR
str.JK67         LSVPTAFLRSWINNHYGSLLTNLWKQENSAILRVEVIVRGMKRVSKGVVCRTSAAPVVLEGQTASSFVESYTEPSVKDIEAGVFGSPLDSR
str.JK19         LSVPTAFLRSWINNHYGSLLTNLWKQENSAILRVEVIVRGMKRVSKGVVCRTSAAPVVLEGQTASSFVESYTEPSVKDIEAGVFGSPLDSR
str.JK63         LSVPTAFLRSWINNHYGSLLTNLWKQENSAILRVEVIVRGMKRVSKGVVCRTSAAPVVLEGQTASSFVESYTEPSVKDIEAGVFGSPLDSR
str.JK39         LSVPTAFLRSWINNHYGSLLTNLWKQENSAILRVEVIVRGMKRVSKGVVCRTSAAPVVLEGQTASSFVESYTEPSVKDIEAGVFGSPLDSR
str.JK68         LSVPTAFLRSWINNHYGSLLTNLWKQENSAILRVEVIVRGMKRVSKGVVCRTSAAPVVLEGQTASSFVESYTEPSVKDIEAGVFGSPLDSR
str.JK31         LSVPTAFLRSWINNHYGSLLTNLWKQENSAILRVEVIVRGMKRVSKGVVCRTSAAPVVLEGQTASSFVESYTEPSVKDIEAGVFGSPLDSR
str.JK73         LSVPTAFLRSWINNHYGSLLTNLWKQENSAILRVEVIVRGMKRVSKGVVCRTSAAPVVLEGQTASSFVESYTEPSVKDIEAGVFGSPLDSR
str.JK7          LSVPTAFLRSWINNHYGSLLTNLWKQENSAILRVEVIVRGMKRVSKGVVCRTSAAPVVLEGQTASSFVESYTEPSVKDIEAGVFGSPLDSR
str.JK73rel      LSVPTAFLRSWINNHYGSLLTNLWKQENSAILRVEVIVRGMKRVSKGVVCRTSAAPVVLEGQTASSFVESYTEPSVKDIEAGVFGSPLDSR
str.BQ2-D70      LSVPTAFLRSWINNHYGSLLTNLWKQENSAILRVEVIVRGMKRVSKGVVCRTSAAPVVLEGQTASSFVESYTEPSVKDIEAGVFGSPLDSR
str.CO20_0321    LSVPTAFLRSWINNHYGSLLTNLWKQENSAILRVEVIVRGMKRVSKGVVCRTSAAPVVLEGQTASSFVESYTEPSVKDIEAGVFGSPLDSR
str.CO21_0024    LSVPTAFLRSWINNHYGSLLTNLWKQENSAILRVEVIVRGMKRVSKGVVCRTSAAPVVLEGQTASSFVESYTEPSVKDIEAGVFGSPLDSR
str.CO20_0297    LSVPTAFLRSWINNHYGSLLTNLWKQENSAILRVEVIVRGMKRVSKGVVCRTSAAPVVLEGQTASSFVESYTEPSVKDIEAGVFGSPLDSR
str.CO20_0256    LSVPTAFLRSWINNHYGSLLTNLWKQENSAILRVEVIVRGMKRVSKGVVCRTSAAPVVLEGQTASSFVESYTEPSVKDIEAGVFGSPLDSR
str.CO20_0257    LSVPTAFLRSWINNHYGSLLTNLWKQENSAILRVEVIVRGMKRVSKGVVCRTSAAPVVLEGQTASSFVESYTEPSVKDIEAGVFGSPLDSR
str.G1712        LSVPTAFLRSWINNHYGSLLTNLWKQENSAILRVEVIVRGMKRVSKGVVCRTSAAPVVLEGQTASSFVESYTEPSVKDIEAGVFGSPLDSR
str.G1713        LSVPTAFLRSWINNHYGSLLTNLWKQENSAILRVEVIVRGMKRVSKGVVCRTSAAPVVLEGQTASSFVESYTEPSVKDIEAGVFGSPLDSR

                   190       200       210       220       230       240       250       260       270
str.Toulouse     YTFESFVEGSSNRVALAAARSIAEGHKSALRFNPLFIHASVGLGKTHLLQAVAAAALKRLMPARVIYLTAEYFMWRFATAIRDNAALSFKE
str.RM-11        YTFESFVEGSSNRVALAAARSIAEGHKSALRFNPLFIHASVGLGKTHLLQAVAAAALKRLMPARVIYLTAEYFMWRFATAIRDNAALSFKE
str.MF1-1        YTFESFVEGSSNRVALAAARSIAEGHKSALRFNPLFIHASVGLGKTHLLQAVAAAALKRLMPARVIYLTAEYFMWRFATAIRDNAALSFKE
str.NCTC12899    YTFESFVEGSSNRVALAAARSIAEGHKSALRFNPLFIHASVGLGKTHLLQAVAAAALKRLMPARVIYLTAEYFMWRFATAIRDNAALSFKE
str.CCUG45777    YTFESFVEGSSNRVALAAARSIAEGHKSALRFNPLFIHASVGLGKTHLLQAVAAAALKRLMPARVIYLTAEYFMWRFATAIRDNAALSFKE
str.JK12         YTFESFVEGSSNRVALAAARSIAEGHKSALRFNPLFIHASVGLGKTHLLQAVAAAALKRLMPARVIYLTAEYFMWRFATAIRDNAALSFKE
str.JK56         YTFESFVEGSSNRVALAAARSIAEGHKSALRFNPLFIHASVGLGKTHLLQAVAAAALKRLMPARVIYLTAEYFMWRFATAIRDNAALSFKE
str.JK67         YTFESFVEGSSNRVALAAARSIAEGHKSALRFNPLFIHASVGLGKTHLLQAVAAAALKRLMPARVIYLTAEYFMWRFATAIRDNAALSFKE
str.JK19         YTFESFVEGSSNRVALAAARSIAEGHKSALRFNPLFIHASVGLGKTHLLQAVAAAALKRLMPARVIYLTAEYFMWRFATAIRDNAALSFKE
str.JK63         YTFESFVEGSSNRVALAAARSIAEGHKSALRFNPLFIHASVGLGKTHLLQAVAAAALKRLMPARVIYLTAEYFMWRFATAIRDNAALSFKE
str.JK39         YTFESFVEGSSNRVALAAARSIAEGHKSALRFNPLFIHASVGLGKTHLLQAVAAAALKRLMPARVIYLTAEYFMWRFATAIRDNAALSFKE
str.JK68         YTFESFVEGSSNRVALAAARSIAEGHKSALRFNPLFIHASVGLGKTHLLQAVAAAALKRLMPARVIYLTAEYFMWRFATAIRDNAALSFKE
str.JK31         YTFESFVEGSSNRVALAAARSIAEGHKSALRFNPLFIHASVGLGKTHLLQAVAAAALKRLMPARVIYLTAEYFMWRFATAIRDNAALSFKE
str.JK73         YTFESFVEGSSNRVALAAARSIAEGHKSALRFNPLFIHASVGLGKTHLLQAVAAAALKRLMPARVIYLTAEYFMWRFATAIRDNAALSFKE
str.JK7          YTFESFVEGSSNRVALAAARSIAEGHKSALRFNPLFIHASVGLGKTHLLQAVAAAALKRLMPARVIYLTAEYFMWRFATAIRDNAALSFKE
str.JK73rel      YTFESFVEGSSNRVALAAARSIAEGHKSALRFNPLFIHASVGLGKTHLLQAVAAAALKRLMPARVIYLTAEYFMWRFATAIRDNAALSFKE
str.BQ2-D70      YTFESFVEGSSNRVALAAARSIAEGHKSALRFNPLFIHASVGLGKTHLLQAVAAAALKRLMPARVIYLTAEYFMWRFATAIRDNAALSFKE
str.CO20_0321    YTFESFVEGSSNRVALAAARSIAEGHKSALRFNPLFIHASVGLGKTHLLQAVAAAALKRLMPARVIYLTAEYFMWRFATAIRDNAALSFKE
str.CO21_0024    YTFESFVEGSSNRVALAAARSIAEGHKSALRFNPLFIHASVGLGKTHLLQAVAAAALKRLMPARVIYLTAEYFMWRFATAIRDNAALSFKE
str.CO20_0297    YTFESFVEGSSNRVALAAARSIAEGHKSALRFNPLFIHASVGLGKTHLLQAVAAAALKRLMPARVIYLTAEYFMWRFATAIRDNAALSFKE
str.CO20_0256    YTFESFVEGSSNRVALAAARSIAEGHKSALRFNPLFIHASVGLGKTHLLQAVAAAALKRLMPARVIYLTAEYFMWRFATAIRDNAALSFKE
str.CO20_0257    YTFESFVEGSSNRVALAAARSIAEGHKSALRFNPLFIHASVGLGKTHLLQAVAAAALKRLMPARVIYLTAEYFMWRFATAIRDNAALSFKE
str.G1712        YTFESFVEGSSNRVALAAARSIAEGHKSALRFNPLFIHASVGLGKTHLLQAVAAAALKRLMPARVIYLTAEYFMWRFATAIRDNAALSFKE
str.G1713        YTFESFVEGSSNRVALAAARSIAEGHKSALRFNPLFIHASVGLGKTHLLQAVAAAALKRLMPARVIYLTAEYFMWRFATAIRDNAALSFKE

                     280       290       300       310       320       330       340       350       360
str.Toulouse     QLRDIDLLIIDDMQFLQGKSIQNEFCHLLNMLLDSAKQVVVAADRPPAELESLDLRVRSRLQGGVALEIEVPDYEMRLKMLRQRLKVVQQD
str.RM-11        QLRDIDLLIIDDMQFLQGKSIQNEFCHLLNMLLDSAKQVVVAADRPPAELESLDLRVRSRLQGGVALEIEVPDYEMRLKMLRQRLKVVQQD
str.MF1-1        QLRDIDLLIIDDMQFLQGKSIQNEFCHLLNMLLDSAKQVVVAADRPPAELESLDLRVRSRLQGGVALEIEVPDYEMRLKMLRQRLKVAQQD
str.NCTC12899    QLRDIDLLIIDDMQFLQGKSIQNEFCHLLNMLLDSAKQVVVAADRPPAELESLDLRVRSRLQGGVALEIEVPDYEMRLKMLRQRLKVVQQD
str.CCUG45777    QLRDIDLLIIDDMQFLQGKSIQNEFCHLLNMLLDSAKQVVVAADRPPAELESLDLRVRSRLQGGVALEIEVPDYEMRLKMLRQRLKVVQQD
str.JK12         QLRDIDLLIIDDMQFLQGKSIQNEFCHLLNMLLDSAKQVVVAADRPPAELESLDLRVRSRLQGGVALEIEVPDYEMRLKMLRQRLKVVQQD
str.JK56         QLRDIDLLIIDDMQFLQGKSIQNEFCHLLNMLLDSAKQVVVAADRPPAELESLDLRVRSRLQGGVALEIEVPDYEMRLKMLRQRLKVVQQD
str.JK67         QLRDIDLLIIDDMQFLQGKSIQNEFCHLLNMLLDSAKQVVVAADRPPAELESLDLRVRSRLQGGVALEIEVPDYEMRLKMLRQRLKVVQQD
str.JK19         QLRDIDLLIIDDMQFLQGKSIQNEFCHLLNMLLDSAKQVVVAADRPPAELESLDLRVRSRLQGGVALEIEVPDYEMRLKMLRQRLKVVQQD
str.JK63         QLRDIDLLIIDDMQFLQGKSIQNEFCHLLNMLLDSAKQVVVAADRPPAELESLDLRVRSRLQGGVALEIEVPDYEMRLKMLRQRLKVVQQD
str.JK39         QLRDIDLLIIDDMQFLQGKSIQNEFCHLLNMLLDSAKQVVVAADRPPAELESLDLRVRSRLQGGVALEIEVPDYEMRLKMLRQRLKVVQQD
str.JK68         QLRDIDLLIIDDMQFLQGKSIQNEFCHLLNMLLDSAKQVVVAADRPPAELESLDLRVRSRLQGGVALEIEVPDYEMRLKMLRQRLKVVQQD
str.JK31         QLRDIDLLIIDDMQFLQGKSIQNEFCHLLNMLLDSAKQVVVAADRPPAELESLDLRVRSRLQGGVALEIEVPDYEMRLKMLRQRLKVVQQD
str.JK73         QLRDIDLLIIDDMQFLQGKSIQNEFCHLLNMLLDSAKQVVVAADRPPAELESLDLRVRSRLQGGVALEIEVPDYEMRLKMLRQRLKVVQQD
str.JK7          QLRDIDLLIIDDMQFLQGKSIQNEFCHLLNMLLDSAKQVVVAADRPPAELESLDLRVRSRLQGGVALEIEVPDYEMRLKMLRQRLKVVQQD
str.JK73rel      QLRDIDLLIIDDMQFLQGKSIQNEFCHLLNMLLDSAKQVVVAADRPPAELESLDLRVRSRLQGGVALEIEVPDYEMRLKMLRQRLKVVQQD
str.BQ2-D70      QLRDIDLLIIDDMQFLQGKSIQNEFCHLLNMLLDSAKQVVVAADRPPAELESLDLRVRSRLQGGVALEIEVPDYEMRLKMLRQRLKVVQQD
str.CO20_0321    QLRDIDLLIIDDMQFLQGKSIQNEFCHLLNMLLDSAKQVVVAADRPPAELESLDLRVRSRLQGGVALEIEVPDYEMRLKMLRQRLKVVQQD
str.CO21_0024    QLRDIDLLIIDDMQFLQGKSIQNEFCHLLNMLLDSAKQVVVAADRPPAELESLDLRVRSRLQGGVALEIEVPDYEMRLKMLRQRLKVVQQD
str.CO20_0297    QLRDIDLLIIDDMQFLQGKSIQNEFCHLLNMLLDSAKQVVVAADRPPAELESLDLRVRSRLQGGVALEIEVPDYEMRLKMLRQRLKVVQQD
str.CO20_0256    QLRDIDLLIIDDMQFLQGKSIQNEFCHLLNMLLDSAKQVVVAADRPPAELESLDLRVRSRLQGGVALEIEVPDYEMRLKMLRQRLKVVQQD
str.CO20_0257    QLRDIDLLIIDDMQFLQGKSIQNEFCHLLNMLLDSAKQVVVAADRPPAELESLDLRVRSRLQGGVALEIEVPDYEMRLKMLRQRLKVVQQD
str.G1712        QLRDIDLLIIDDMQFLQGKSIQNEFCHLLNMLLDSAKQVVVAADRPPAELESLDLRVRSRLQGGVALEIEVPDYEMRLKMLRQRLKVVQQD
str.G1713        QLRDIDLLIIDDMQFLQGKSIQNEFCHLLNMLLDSAKQVVVAADRPPAELESLDLRVRSRLQGGVALEIEVPDYEMRLKMLRQRLKVVQQD
```

```
                    370       380       390       400       410       420       430       440       450
str.Toulouse    DNMVVISDEVLQYIAKTVLGSGRDIEGAFNQLLFRQSFESDLSLERIDELLGHLTRSGESKRIRIEEIQRAVARHYNVSKQDLLSNRRTRT
str.RM-11       DNMVVISDEVLQYIAKTVLGSGRDIEGAFNQLLFRQSFESDLSLERIDELLGHLTRSGESKRIRIEEIQRAVARHYNVSKQDLLSNRRTRT
str.MF1-1       DNMVVISDEVLQYIAKTILGSGRDIEGAFNQLLFRQSFESDLSLERIDELLGHLTRSGESKRIRIEEIQRAVARHYNVSKQDLLSNRRTRT
str.NCTC12899   DNMVVISDEVLQYIAKTVLGSGRDIEGAFNQLLFRQSFESDLSLERIDELLGHLTRSGESKRIRIEEIQRAVARHYNVSKQDLLSNRRTRT
str.CCUG45777   DNMVVISDEVLQYIAKTVLGSGRDIEGAFNQLLFRQSFESDLSLERIDELLGHLTRSGESKRIRIEEIQRAVARHYNVSKQDLLSNRRTRT
str.JK12        DNMVVISDEVLQYIAKTVLGSGRDIEGAFNQLLFRQSFESDLSLERIDELLGHLTRSGESKRIRIEEIQRAVARHYNVSKQDLLSNRRTRT
str.JK56        DNMVVISDEVLQYIAKTVLGSGRDIEGAFNQLLFRQSFESDLSLERIDELLGHLTRSGESKRIRIEEIQRAVARHYNVSKQDLLSNRRTRT
str.JK67        DNMVVISDEVLQYIAKTVLGSGRDIEGAFNQLLFRQSFESDLSLERIDELLGHLTRSGESKRIRIEEIQRAVARHYNVSKQDLLSNRRTRT
str.JK19        DNMVVISDEVLQYIAKTVLGSGRDIEGAFNQLLFRQSFESDLSLERIDELLGHLTRSGESKRIRIEEIQRAVARHYNVSKQDLLSNRRTRT
str.JK63        DNMVVISDEVLQYIAKTVLGSGRDIEGAFNQLLFRQSFESDLSLERIDELLGHLTRSGESKRIRIEEIQRAVARHYNVSKQDLLSNRRTRT
str.JK39        DNMVVISDEVLQYIAKTVLGSGRDIEGAFNQLLFRQSFESDLSLERIDELLGHLTRSGESKRIRIEEIQRAVARHYNVSKQDLLSNRRTRT
str.JK68        DNMVVISDEVLQYIAKTVLGSGRDIEGAFNQLLFRQSFESDLSLERIDELLGHLTRSGESKRIRIEEIQRAVARHYNVSKQDLLSNRRTRT
str.JK31        DNMVVISDEVLQYIAKTVLGSGRDIEGAFNQLLFRQSFESDLSLERIDELLGHLTRSGESKRIRIEEIQRAVARHYNVSKQDLLSNRRTRT
str.JK73        DNMVVISDEVLQYIAKTVLGSGRDIEGAFNQLLFRQSFESDLSLERIDELLGHLTRSGESKRIRIEEIQRAVARHYNVSKQDLLSNRRTRT
str.JK7         DNMVVISDEVLQYIAKTVLGSGRDIEGAFNQLLFRQSFESDLSLERIDELLGHLTRSGESKRIRIEEIQRAVARHYNVSKQDLLSNRRTRT
str.JK73rel     DNMVVISDEVLQYIAKTVLGSGRDIEGAFNQLLFRQSFESDLSLERIDELLGHLTRSGESKRIRIEEIQRAVARHYNVSKQDLLSNRRTRT
str.BQ2-D70     DNMVVISDEVLQYIAKTVLGSGRDIEGAFNQLLFRQSFESDLSLERIDELLGHLTRSGESKRIRIEEIQRAVARHYNVSKQDLLSNRRTRT
str.CO20_0321   DNMVVISDEVLQYIAKTVLGSGRDIEGAFNQLLFRQSFESDLSLERIDELLGHLTRSGESKRIRIEEIQRAVARHYNVSKQDLLSNRRTRT
str.CO21_0024   DNMVVISDEVLQYIAKTVLGSGRDIEGAFNQLLFRQSFESDLSLERIDELLGHLTRSGESKRIRIEEIQRAVARHYNVSKQDLLSNRRTRT
str.CO20_0297   DNMVVISDEVLQYIAKTVLGSGRDIEGAFNQLLFRQSFESDLSLERIDELLGHLTRSGESKRIRIEEIQRAVARHYNVSKQDLLSNRRTRT
str.CO20_0256   DNMVVISDEVLQYIAKTVLGSGRDIEGAFNQLLFRQSFESDLSLERIDELLGHLTRSGESKRIRIEEIQRAVARHYNVSKQDLLSNRRTRT
str.CO20_0257   DNMVVISDEVLQYIAKTVLGSGRDIEGAFNQLLFRQSFESDLSLERIDELLGHLTRSGESKRIRIEEIQRAVARHYNVSKQDLLSNRRTRT
str.G1712       DNMVVISDEVLQYIAKTVLGSGRDIEGAFNQLLFRQSFESDLSLERIDELLGHLTRSGESKRIRIEEIQRAVARHYNVSKQDLLSNRRTRT
str.G1713       DNMVVISDEVLQYIAKTVLGSGRDIEGAFNQLLFRQSFESDLSLERIDELLGHLTRSGESKRIRIEEIQRAVARHYNVSKQDLLSNRRTRT

                    460       470       480       490       500       510       520
str.Toulouse    VVKPRQVAMYLAKMLTPRSLPEIGRRFGGRDHTTVLHAVRKIEDLVCDDQTLAKELELLKRLIGEQAA
str.RM-11       VVKPRQVAMYLAKMLTPRSLPEIGRRFGGRDHTTVLHAVRKIEDLVCDDQTLAKELELLKRLIGEQAA
str.MF1-1       VVKPRQVAMYLAKMLTPRSLPEIGRRFGGRDHTTVLHAVRKIEDLVCDDQTLAKELELLKRLIGEQAA
str.NCTC12899   VVKPRQVAMYLAKMLTPRSLPEIGRRFGGRDHTTVLHAVRKIEDLVCDDQTLAKELELLKRLIGEQAA
str.CCUG45777   VVKPRQVAMYLAKMLTPRSLPEIGRRFGGRDHTTVLHAVRKIEDLVCDDQTLAKELELLKRLIGEQAA
str.JK12        VVKPRQVAMYLAKMLTPRSLPEIGRRFGGRDHTTVLHAVRKIEDLVCDDQTLAKELELLKRLIGEQAA
str.JK56        VVKPRQVAMYLAKMLTPRSLPEIGRRFGGRDHTTVLHAVRKIEDLVCDDQTLAKELELLKRLIGEQAA
str.JK67        VVKPRQVAMYLAKMLTPRSLPEIGRRFGGRDHTTVLHAVRKIEDLVCDDQTLAKELELLKRLIGEQAA
str.JK19        VVKPRQVAMYLAKMLTPRSLPEIGRRFGGRDHTTVLHAVRKIEDLVCDDQTLAKELELLKRLIGEQAA
str.JK63        VVKPRQVAMYLAKMLTPRSLPEIGRRFGGRDHTTVLHAVRKIEDLVCDDQTLAKELELLKRLIGEQAA
str.JK39        VVKPRQVAMYLAKMLTPRSLPEIGRRFGGRDHTTVLHAVRKIEDLVCDDQTLAKELELLKRLIGEQAA
str.JK68        VVKPRQVAMYLAKMLTPRSLPEIGRRFGGRDHTTVLHAVRKIEDLVCDDQTLAKELELLKRLIGEQAA
str.JK31        VVKPRQVAMYLAKMLTPRSLPEIGRRFGGRDHTTVLHAVRKIEDLVCDDQTLAKELELLKRLIGEQAA
str.JK73        VVKPRQVAMYLAKMLTPRSLPEIGRRFGGRDHTTVLHAVRKIEDLVCDDQTLAKELELLKRLIGEQAA
str.JK7         VVKPRQVAMYLAKMLTPRSLPEIGRRFGGRDHTTVLHAVRKIEDLVCDDQTLAKELELLKRLIGEQAA
str.JK73rel     VVKPRQVAMYLAKMLTPRSLPEIGRRFGGRDHTTVLHAVRKIEDLVCDDQTLAKELELLKRLIGEQAA
str.BQ2-D70     VVKPRQVAMYLAKMLTPRSLPEIGRRFGGRDHTTVLHAVRKIEDLVCDDQTLAKELELLKRLIGEQAA
str.CO20_0321   VVKPRQVAMYLAKMLTPRSLPEIGRRFGGRDHTTVLHAVRKIEDLVCDDQTLAKELELLKRLIGEQAA
str.CO21_0024   VVKPRQVAMYLAKMLTPRSLPEIGRRFGGRDHTTVLHAVRKIEDLVCDDQTLAKELELLKRLIGEQAA
str.CO20_0297   VVKPRQVAMYLAKMLTPRSLPEIGRRFGGRDHTTVLHAVRKIEDLVCDDQTLAKELELLKRLIGEQAA
str.CO20_0256   VVKPRQVAMYLAKMLTPRSLPEIGRRFGGRDHTTVLHAVRKIEDLVCDDQTLAKELELLKRLIGEQAA
str.CO20_0257   VVKPRQVAMYLAKMLTPRSLPEIGRRFGGRDHTTVLHAVRKIEDLVCDDQTLAKELELLKRLIGEQAA
str.G1712       VVKPRQVAMYLAKMLTPRSLPEIGRRFGGRDHTTVLHAVRKIEDLVCDDQTLAKELELLKRLIGEQAA
str.G1713       VVKPRQVAMYLAKMLTPRSLPEIGRRFGGRDHTTVLHAVRKIEDLVCDDQTLAKELELLKRLIGEQAA
```

>WP_011178959.1 phosphoenolpyruvate - protein phosphotransferase [Bartonella quintana str. Toulouse]

```
                1        10        20        30        40        50        60        70        80        90
str.Toulouse    MNTGFNEIEILSIRSVKLAAAFDHKNDAIHAAAELLVQIGAVDKCYLASMLKREAVTNTWLGNGIAIPHGMVENRDLIIRDAVAVVQIPAG
str.G1712       MNTGFNEIEILSIRSVKLAAAFDHKNDAIHAAAELLVQIGAVDKCYLASMLKREAVTNTWLGNGIAIPHGMVENRDLIIRDAVAVVQIPAG
str.G1713       MNTGFNEIEILSIRSVKLAAAFDHKNDAIHAAAELLVQIGAVDKCYLASMLKREAVTNTWLGNGIAIPHGMVENRDLIIRDAVAVVQIPAG
str.CCUG45777   MNTGFNEIEILSIRSVKLAAAFDHKNDAIHAAAELLVQIGAVDKCYLASMLKREAVTNTWLGNGIAIPHGMVENRDLIIRDAVAVVQIPAG
str.NCTC12899   MNTGFNEIEILSIRSVKLAAAFDHKNDAIHAAAELLVQIGAVDKCYLASMLKREAVTNTWLGNGIAIPHGMVENRDLIIRDAVAVVQIPAG
str.CO20_0321   MNTGFNEIEILSIRSVKLAAAFDHKNDAIHAAAELLVQIGAVDKCYLASMLKREAVTNTWLGNGIAIPHGMVENRDLIIRDAVAVVQIPAG
str.BQ2-D70     MNTGFNEIEILSIRSVKLAAAFDHKNDAIHAAAELLVQIGAVDKCYLASMLKREAVTNTWLGNGIAIPHGMVENRDLIIRDAVAVVQIPAG
str.JK73rel     MNTGFNEIEILSIRSVKLAAAFDHKNDAIHAAAELLVQIGAVDKCYLASMLKREAVTNTWLGNGIAIPHGMVENRDLIIRDAVAVVQIPAG
str.JK7         MNTGFNEIEILSIRSVKLAAAFDHKNDAIHAAAELLVQIGAVDKCYLASMLKREAVTNTWLGNGIAIPHGMVENRDLIIRDAVAVVQIPAG
str.JK73        MNTGFNEIEILSIRSVKLAAAFDHKNDAIHAAAELLVQIGAVDKCYLASMLKREAVTNTWLGNGIAIPHGMVENRDLIIRDAVAVVQIPAG
str.JK31        MNTGFNEIEILSIRSVKLAAAFDHKNDAIHAAAELLVQIGAVDKCYLASMLKREAVTNTWLGNGIAIPHGMVENRDLIIRDAVAVVQIPAG
str.JK68        MNTGFNEIEILSIRSVKLAAAFDHKNDAIHAAAELLVQIGAVDKCYLASMLKREAVTNTWLGNGIAIPHGMVENRDLIIRDAVAVVQIPAG
str.JK39        MNTGFNEIEILSIRSVKLAAAFDHKNDAIHAAAELLVQIGAVDKCYLASMLKREAVTNTWLGNGIAIPHGMVENRDLIIRDAVAVVQIPAG
str.JK63        MNTGFNEIEILSIRSVKLAAAFDHKNDAIHAAAELLVQIGAVDKCYLASMLKREAVTNTWLGNGIAIPHGMVENRDLIIRDAVAVVQIPAG
str.JK19        MNTGFNEIEILSIRSVKLAAAFDHKNDAIHAAAELLVQIGAVDKCYLASMLKREAVTNTWLGNGIAIPHGMVENRDLIIRDAVAVVQIPAG
str.JK67        MNTGFNEIEILSIRSVKLAAAFDHKNDAIHAAAELLVQIGAVDKCYLASMLKREAVTNTWLGNGIAIPHGMVENRDLIIRDAVAVVQIPAG
str.JK12        MNTGFNEIEILSIRSVKLAAAFDHKNDAIHAAAELLVQIGAVDKCYLASMLKREAVTNTWLGNGIAIPHGMVENRDLIIRDAVAVVQIPAG
str.CO20_0257   MNTGFNEIEILSIRSVKLAAAFDHKNDAIHAATELLVQIGAVDKCYLASMLKREAVTNTWLGNGIAIPHGMVENRDLIIRDAVAVVQIPAG
str.CO20_0256   MNTGFNEIEILSIRSVKLAAAFDHKNDAIHAATELLVQIGAVDKCYLASMLKREAVTNTWLGNGIAIPHGMVENRDLIIRDAVAVVQIPAG
str.CO20_0297   MNTGFNEIEILSIRSVKLAAAFDHKNDAIHAATELLVQIGAVDKCYLASMLKREAVTNTWLGNGIAIPHGMVENRDLIIRDAVAVVQIPAG
str.CO21_0024   MNTGFNEIEILSIRSVKLAAAFDHKNDAIHAATELLVQIGAVDKCYLASMLKREAVTNTWLGNGIAIPHGMVENRDLIIRDAVAVVQIPAG
str.JK56        MNTGFNEIEILSIRSVKLAAAFDHKNDAIHAAAELLVQIGAVDKCYLASMLKREAVTNTWLGNGIAIPHGMVENRDLIIRDAVAVVQIPAG
str.RM-11       MNTGFNEIEILSIRSVKLAAAFDHKNDAIHAAAELLVQIGAVDKCYLASMLKREAVTNTWLGNGIAIPHGMVENRDLIIRDAVAVVQIPAG
str.MF1-1       MNTGFNEIEILSIRSVKLAAAFDHKNDAIHAAAELLVQIGAVDKCYLASMLKREAVTNTWLGNGIAIPHGMVENRDLIIRDAVAVVQIPAG

                        100       110       120       130       140       150       160       170       180
str.Toulouse    VEWRDGNKARLVIAIAACPDRYIEICKKLMRLLFDKEQLEALSTTADRQQIVTTLFGQDQDTESGRTFIGDLSVCQEWTLDYPSGLHARPA
str.G1712       VEWRDGNKARLVIAIAACPDRYIEICKKLMRLLFDKEQLEALSTTADRQQIVTTLFGQDQDTESGRTFIGDLSVCQEWTLDYPSGLHARPA
str.G1713       VEWRDGNKARLVIAIAACPDRYIEICKKLMRLLFDKEQLEALSTTADRQQIVTTLFGQDQDTESGRTFIGDLSVCQEWTLDYPSGLHARPA
str.CCUG45777   VEWRDGNKARLVIAIAACPDRYIEICKKLMRLLFDKEQLEALSTTADRQQIVTTLFGQDQDTESGRTFIGDLSVCQEWTLDYPSGLHARPA
str.NCTC12899   VEWRDGNKARLVIAIAACPDRYIEICKKLMRLLFDKEQLEALSTTADRQQIVTTLFGQDQDTESGRTFIGDLSVCQEWTLDYPSGLHARPA
str.CO20_0321   VEWRDGNKARLVIAIAACPDRYIEICKKLMRLLFDKEQLEALSTTADRQQIVTTLFGQDQDTESGRTFIGDLSVCQEWTLDYPSGLHARPA
str.BQ2-D70     VEWRDGNKARLVIAIAACPDRYIEICKKLMRLLFDKEQLEALSTTADRQQIVTTLFGQDQDTESGRTFIGDLSVCQEWTLDYPSGLHARPA
str.JK73rel     VEWRDGNKARLVIAIAACPDRYIEICKKLMRLLFDKEQLEALSTTADRQQIVTTLFGQDQDTESGRTFIGDLSVCQEWTLDYPSGLHARPA
str.JK7         VEWRDGNKARLVIAIAACPDRYIEICKKLMRLLFDKEQLEALSTTADRQQIVTTLFGQDQDTESGRTFIGDLSVCQEWTLDYPSGLHARPA
str.JK73        VEWRDGNKARLVIAIAACPDRYIEICKKLMRLLFDKEQLEALSTTADRQQIVTTLFGQDQDTESGRTFIGDLSVCQEWTLDYPSGLHARPA
str.JK31        VEWRDGNKARLVIAIAACPDRYIEICKKLMRLLFDKEQLEALSTTADRQQIVTTLFGQDQDTESGRTFIGDLSVCQEWTLDYPSGLHARPA
str.JK68        VEWRDGNKARLVIAIAACPDRYIEICKKLMRLLFDKEQLEALSTTADRQQIVTTLFGQDQDTESGRTFIGDLSVCQEWTLDYPSGLHARPA
str.JK39        VEWRDGNKARLVIAIAACPDRYIEICKKLMRLLFDKEQLEALSTTADRQQIVTTLFGQDQDTESGRTFIGDLSVCQEWTLDYPSGLHARPA
str.JK63        VEWRDGNKARLVIAIAACPDRYIEICKKLMRLLFDKEQLEALSTTADRQQIVTTLFGQDQDTESGRTFIGDLSVCQEWTLDYPSGLHARPA
str.JK19        VEWRDGNKARLVIAIAACPDRYIEICKKLMRLLFDKEQLEALSTTADRQQIVTTLFGQDQDTESGRTFIGDLSVCQEWTLDYPSGLHARPA
str.JK67        VEWRDGNKARLVIAIAACPDRYIEICKKLMRLLFDKEQLEALSTTADRQQIVTTLFGQDQDTESGRTFIGDLSVCQEWTLDYPSGLHARPA
str.JK12        VEWRDGNKARLVIAIAACPDRYIEICKKLMRLLFDKEQLEALSTTADRQQIVTTLFGQDQDTESGRTFIGDLSVCQEWTLDYPSGLHARPA
str.CO20_0257   VEWRDGNKARLVIAIAACPDRYIEICKKLMRLLFDKEQLEALSTTADRQQIVTTLFGQDQDTESGRTFIGDLSVCQEWTLDYPSGLHARPA
str.CO20_0256   VEWRDGNKARLVIAIAACPDRYIEICKKLMRLLFDKEQLEALSTTADRQQIVTTLFGQDQDTESGRTFIGDLSVCQEWTLDYPSGLHARPA
str.CO20_0297   VEWRDGNKARLVIAIAACPDRYIEICKKLMRLLFDKEQLEALSTTADRQQIVTTLFGQDQDTESGRTFIGDLSVCQEWTLDYPSGLHARPA
str.CO21_0024   VEWRDGNKARLVIAIAACPDRYIEICKKLMRLLFDKEQLEALSTTADRQQIVTTLFGQDQDTESGRTFIGDLSVCQEWTLDYPSGLHARPA
str.JK56        VEWRDGNKARLVIAIAACPDRYIEICKKLMRLLFDKEQLEALSTTADRQQIVTTLFGQDQDTESGRTFIGDLSVCQEWTLDYPSGLHARPA
str.RM-11       VEWRDGNKARLVIAIAACPDRYIEICKKLMRLLFNKEQLEALSTTADRQQIVTTLFGQDQDTESGRTFIGDLSVCQEWTLDYPSGLHARPA
str.MF1-1       VEWRDGNKARLVIAIAACPDRYIEICKKLMRLLFNKEQLEALSTTADRQQIVTTLFGQDQDTESGRTFIGDLSVCQEWTLDYPSGLHARPA

                      190       200       210       220       230       240       250       260       270
str.Toulouse    SLWVDFAKKAQSSIRVRRGQYAVEMKNLVGLLQLGAKNGDVLIFSTDALEGAQLLEEAISVVKKVSISEKCVIREMEAQTKTFHSWYPRSG
str.G1712       SLWVDFAKKAQSSIRVRRGQYAVEMKNLVGLLQLGAKNGDVLIFSTDALEGAQLLEEAISVVKKVSISEKCVIREMEAQTKTFHSWYPRSG
str.G1713       SLWVDFAKKAQSSIRVRRGQYAVEMKNLVGLLQLGAKNGDVLIFSTDALEGAQLLEEAISVVKKVSISEKCVIREMEAQTKTFHSWYPRSG
str.CCUG45777   SLWVDFAKKAQSSIRVRRGQYAVEMKNLVGLLQLGAKNGDVLIFSTDALEGAQLLEEAISVVKKVSISEKCVIREMEAQTKTFHSWYPRSG
str.NCTC12899   SLWVDFAKKAQSSIRVRRGQYAVEMKNLVGLLQLGAKNGDVLIFSTDALEGAQLLEEAISVVKKVSISEKCVIREMEAQTKTFHSWYPRSG
str.CO20_0321   SLWVDFAKKAQSSIRVRRGQYAVEMKNLVGLLQLGAKNGDVLIFSTDALEGAQLLEEAISVVKKVSISEKCVIREMEAQTKTFHSWYPRSG
str.BQ2-D70     SLWVDFAKKAQSSIRVRRGQYAVEMKNLVGLLQLGAKNGDVLIFSTDALEGAQLLEEAISVVKKVSISEKCVIREMEAQTKTFHSWYPRSG
str.JK73rel     SLWVDFAKKAQSSIRVRRGQYAVEMKNLVGLLQLGAKNGDVLIFSTDALEGAQLLEEAISVVKKVSISEKCVIREMEAQTKTFHSWYPRSG
str.JK7         SLWVDFAKKAQSSIRVRRGQYAVEMKNLVGLLQLGAKNGDVLIFSTDALEGAQLLEEAISVVKKVSISEKCVIREMEAQTKTFHSWYPRSG
str.JK73        SLWVDFAKKAQSSIRVRRGQYAVEMKNLVGLLQLGAKNGDVLIFSTDALEGAQLLEEAISVVKKVSISEKCVIREMEAQTKTFHSWYPRSG
str.JK31        SLWVDFAKKAQSSIRVRRGQYAVEMKNLVGLLQLGAKNGDVLIFSTDALEGAQLLEEAISVVKKVSISEKCVIREMEAQTKTFHSWYPRSG
str.JK68        SLWVDFAKKAQSSIRVRRGQYAVEMKNLVGLLQLGAKNGDVLIFSTDALEGAQLLEEAISVVKKVSISEKCVIREMEAQTKTFHSWYPRSG
str.JK39        SLWVDFAKKAQSSIRVRRGQYAVEMKNLVGLLQLGAKNGDVLIFSTDALEGAQLLEEAISVVKKVSISEKCVIREMEAQTKTFHSWYPRSG
str.JK63        SLWVDFAKKAQSSIRVRRGQYAVEMKNLVGLLQLGAKNGDVLIFSTDALEGAQLLEEAISVVKKVSISEKCVIREMEAQTKTFHSWYPRSG
str.JK19        SLWVDFAKKAQSSIRVRRGQYAVEMKNLVGLLQLGAKNGDVLIFSTDALEGAQLLEEAISVVKKVSISEKCVIREMEAQTKTFHSWYPRSG
str.JK67        SLWVDFAKKAQSSIRVRRGQYAVEMKNLVGLLQLGAKNGDVLIFSTDALEGAQLLEEAISVVKKVSISEKCVIREMEAQTKTFHSWYPRSG
str.JK12        SLWVDFAKKAQSSIRVRRGQYAVEMKNLVGLLQLGAKNGDVLIFSTDALEGAQLLEEAISVVKKVSISEKCVIREMEAQTKTFHSWYPRSG
str.CO20_0257   SLWVDFAKKAQSSIRVRRGQYAVEMKNLVGLLQLGAKNGDVLIFSTDALEGAQLLEEAISVVKKVSISEKCVIREMEAQTKTFHSWYPRSG
str.CO20_0256   SLWVDFAKKAQSSIRVRRGQYAVEMKNLVGLLQLGAKNGDVLIFSTDALEGAQLLEEAISVVKKVSISEKCVIREMEAQTKTFHSWYPRSG
str.CO20_0297   SLWVDFAKKAQSSIRVRRGQYAVEMKNLVGLLQLGAKNGDVLIFSTDALEGAQLLEEAISVVKKVSISEKCVIREMEAQTKTFHSWYPRSG
str.CO21_0024   SLWVDFAKKAQSSIRVRRGQYAVEMKNLVGLLQLGAKNGDVLIFSTDALEGAQLLEEAISVVKKVSISEKCVIREMEAQTKTFHSWYPRSG
str.JK56        SLWVDFAKKAQSSIRVRRGQYAVEMKNLVGLLQLGAKNGDVLIFSTDALEGAQLLEEAISVVKKVSISEKCVIREMEAQTKTFHSWYPRSG
str.RM-11       SLWVDFAKKAQSSIRVRRGQYAVEMKNLVGLLQLGAKNGDVLIFSTDALEGAQLLEEAISVVKKVSISEKCVIREMEAQTKTFHSWYPRSG
str.MF1-1       SLWVDFAKKAQSSIRVRRGQYAVEMKNLVGLLQLGAKNGDVLIFSTDALEGAQLLEEAISVVKKVSISEKCVIREMEAQTKTFHSWYPRSG

                      280       290       300       310       320       330       340       350       360
str.Toulouse    QRGLSGIGVSPGLALGKIFVLRQNDISITDQPTGFVAGTVCLENALTKTKHKMASIIDDITARMGADSAAIFSAQMVLLEDENLIAQACRF
str.G1712       QRGLSGIGVSPGLALGKIFVLRQNDISITDQPTGFVAGTVCLENALTKTKHKMASIIDDITARMGADSAAIFSAQMVLLEDENLIAQACRF
str.G1713       QRGLSGIGVSPGLALGKIFVLRQNDISITDQPTGFVAGTVCLENALTKTKHKMASIIDDITARMGADSAAIFSAQMVLLEDENLIAQACRF
str.CCUG45777   QRGLSGIGVSPGLALGKIFVLRQNDISITDQPTGFVAGTVCLENALTKTKHKMASIIDDITARMGADSAAIFSAQMVLLEDENLIAQACRF
str.NCTC12899   QRGLSGIGVSPGLALGKIFVLRQNDISITDQPTGFVAGTVCLENALTKTKHKMASIIDDITARMGADSAAIFSAQMVLLEDENLIAQACRF
str.CO20_0321   QRGLSGIGVSPGLALGKIFVLRQNDISITDQPTGFVAGTVCLENALTKTKHKMASIIDDITARMGADSAAIFSAQMVLLEDENLIAQACRF
str.BQ2-D70     QRGLSGIGVSPGLALGKIFVLRQNDISITDQPTGFVAGTVCLENALTKTKHKMASIIDDITARMGADSAAIFSAQMVLLEDENLIAQACRF
str.JK73rel     QRGLSGIGVSPGLALGKIFVLRQNDISITDQPTGFVAGTVCLENALTKTKHKMASIIDDITARMGADSAAIFSAQMVLLEDENLIAQACRF
str.JK7         QRGLSGIGVSPGLALGKIFVLRQNDISITDQPTGFVAGTVCLENALTKTKHKMASIIDDITARMGADSAAIFSAQMVLLEDENLIAQACRF
str.JK73        QRGLSGIGVSPGLALGKIFVLRQNDISITDQPTGFVAGTVCLENALTKTKHKMASIIDDITARMGADSAAIFSAQMVLLEDENLIAQACRF
str.JK31        QRGLSGIGVSPGLALGKIFVLRQNDISITDQPTGFVAGTVCLENALTKTKHKMASIIDDITARMGADSAAIFSAQMVLLEDENLIAQACRF
str.JK68        QRGLSGIGVSPGLALGKIFVLRQNDISITDQPTGFVAGTVCLENALTKTKHKMASIIDDITARMGADSAAIFSAQMVLLEDENLIAQACRF
str.JK39        QRGLSGIGVSPGLALGKIFVLRQNDISITDQPTGFVAGTVCLENALTKTKHKMASIIDDITARMGADSAAIFSAQMVLLEDENLIAQACRF
str.JK63        QRGLSGIGVSPGLALGKIFVLRQNDISITDQPTGFVAGTVCLENALTKTKHKMASIIDDITARMGADSAAIFSAQMVLLEDENLIAQACRF
str.JK19        QRGLSGIGVSPGLALGKIFVLRQNDISITDQPTGFVAGTVCLENALTKTKHKMASIIDDITARMGADSAAIFSAQMVLLEDENLIAQACRF
str.JK67        QRGLSGIGVSPGLALGKIFVLRQNDISITDQPTGFVAGTVCLENALTKTKHKMASIIDDITARMGADSAAIFSAQMVLLEDENLIAQACRF
str.JK12        QRGLSGIGVSPGLALGKIFVLRQNDISITDQPTGFVAGTVCLENALTKTKHKMASIIDDITARMGADSAAIFSAQMVLLEDENLIAQACRF
str.CO20_0257   QRGLSGIGVSPGLALGKIFVLRQNDISITDQPTGFVAGTVCLENALTKTKHKMASIIDDITARMGADSAAIFSAQMVLLEDENLIAQACRF
str.CO20_0256   QRGLSGIGVSPGLALGKIFVLRQNDISITDQPTGFVAGTVCLENALTKTKHKMASIIDDITARMGADSAAIFSAQMVLLEDENLIAQACRF
str.CO20_0297   QRGLSGIGVSPGLALGKIFVLRQNDISITDQPTGFVAGTVCLENALTKTKHKMASIIDDITARMGADSAAIFSAQMVLLEDENLIAQACRF
str.CO21_0024   QRGLSGIGVSPGLALGKIFVLRQNDISITDQPTGFVAGTVCLENALTKTKHKMASIIDDITARMGADSAAIFSAQMVLLEDENLIAQACRF
str.JK56        QRGLSGIGVSPGLALGKIFVLRQNDISITDQPTGFVAGTVCLENALTKTKHKMASIIDDITARMGADSAAIFSAQMVLLEDENLIAQACRF
str.RM-11       QRGLSGIGVSPGLALGKIFVLRQNDISITDQPTGFVAGTVCLENALTKTKHKMASIIDDITARMGADSAAIFSAQMVLLEDENLIAQACRF
str.MF1-1       QRGLSGIGVSPGLALGKIFVLRQNDISITDQPTGFVAGTVCLENALTKTKHKMASIIDDITARMGADSAAIFSAQMVLLEDENLIAQACRF
```

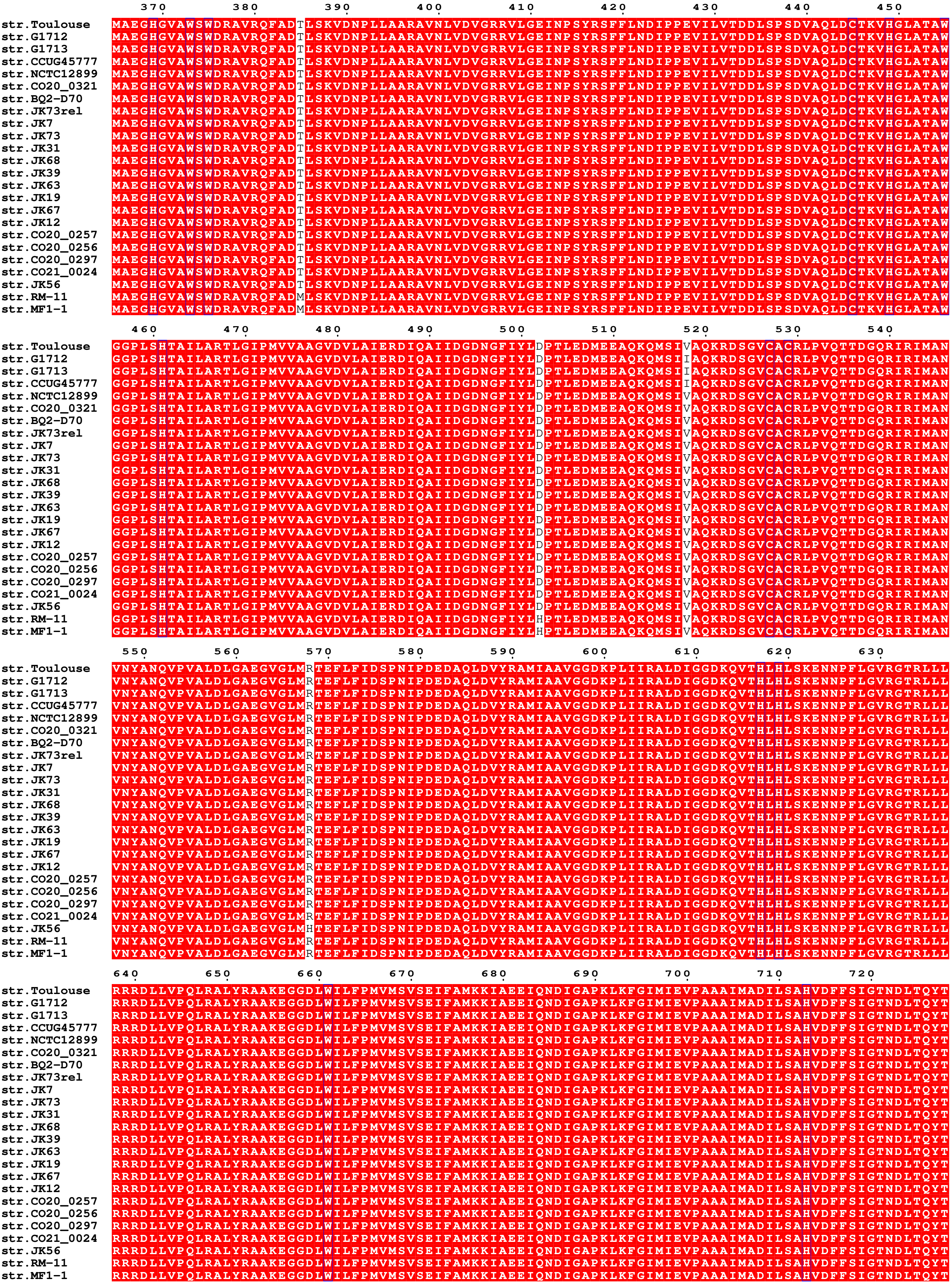
370 380 390 400 410 420 430 440 450
str.Toulouse MAEGHGVAWSWDRAVRQFADTLSKVDNPLLAARAVNLVDVGRRVLGEINPSYRSFFLNDIPPEVILVTDDLSPSDVAQLDCTKVHGLATAW
str.G1712 MAEGHGVAWSWDRAVRQFADTLSKVDNPLLAARAVNLVDVGRRVLGEINPSYRSFFLNDIPPEVILVTDDLSPSDVAQLDCTKVHGLATAW
str.G1713 MAEGHGVAWSWDRAVRQFADTLSKVDNPLLAARAVNLVDVGRRVLGEINPSYRSFFLNDIPPEVILVTDDLSPSDVAQLDCTKVHGLATAW
str.CCUG45777 MAEGHGVAWSWDRAVRQFADTLSKVDNPLLAARAVNLVDVGRRVLGEINPSYRSFFLNDIPPEVILVTDDLSPSDVAQLDCTKVHGLATAW
str.NCTC12899 MAEGHGVAWSWDRAVRQFADTLSKVDNPLLAARAVNLVDVGRRVLGEINPSYRSFFLNDIPPEVILVTDDLSPSDVAQLDCTKVHGLATAW
str.CO20_0321 MAEGHGVAWSWDRAVRQFADTLSKVDNPLLAARAVNLVDVGRRVLGEINPSYRSFFLNDIPPEVILVTDDLSPSDVAQLDCTKVHGLATAW
str.BQ2-D70 MAEGHGVAWSWDRAVRQFADTLSKVDNPLLAARAVNLVDVGRRVLGEINPSYRSFFLNDIPPEVILVTDDLSPSDVAQLDCTKVHGLATAW
str.JK73rel MAEGHGVAWSWDRAVRQFADTLSKVDNPLLAARAVNLVDVGRRVLGEINPSYRSFFLNDIPPEVILVTDDLSPSDVAQLDCTKVHGLATAW
str.JK7 MAEGHGVAWSWDRAVRQFADTLSKVDNPLLAARAVNLVDVGRRVLGEINPSYRSFFLNDIPPEVILVTDDLSPSDVAQLDCTKVHGLATAW
str.JK73 MAEGHGVAWSWDRAVRQFADTLSKVDNPLLAARAVNLVDVGRRVLGEINPSYRSFFLNDIPPEVILVTDDLSPSDVAQLDCTKVHGLATAW
str.JK31 MAEGHGVAWSWDRAVRQFADTLSKVDNPLLAARAVNLVDVGRRVLGEINPSYRSFFLNDIPPEVILVTDDLSPSDVAQLDCTKVHGLATAW
str.JK68 MAEGHGVAWSWDRAVRQFADTLSKVDNPLLAARAVNLVDVGRRVLGEINPSYRSFFLNDIPPEVILVTDDLSPSDVAQLDCTKVHGLATAW
str.JK39 MAEGHGVAWSWDRAVRQFADTLSKVDNPLLAARAVNLVDVGRRVLGEINPSYRSFFLNDIPPEVILVTDDLSPSDVAQLDCTKVHGLATAW
str.JK63 MAEGHGVAWSWDRAVRQFADTLSKVDNPLLAARAVNLVDVGRRVLGEINPSYRSFFLNDIPPEVILVTDDLSPSDVAQLDCTKVHGLATAW
str.JK19 MAEGHGVAWSWDRAVRQFADTLSKVDNPLLAARAVNLVDVGRRVLGEINPSYRSFFLNDIPPEVILVTDDLSPSDVAQLDCTKVHGLATAW
str.JK67 MAEGHGVAWSWDRAVRQFADTLSKVDNPLLAARAVNLVDVGRRVLGEINPSYRSFFLNDIPPEVILVTDDLSPSDVAQLDCTKVHGLATAW
str.JK12 MAEGHGVAWSWDRAVRQFADTLSKVDNPLLAARAVNLVDVGRRVLGEINPSYRSFFLNDIPPEVILVTDDLSPSDVAQLDCTKVHGLATAW
str.CO20_0257 MAEGHGVAWSWDRAVRQFADTLSKVDNPLLAARAVNLVDVGRRVLGEINPSYRSFFLNDIPPEVILVTDDLSPSDVAQLDCTKVHGLATAW
str.CO20_0256 MAEGHGVAWSWDRAVRQFADTLSKVDNPLLAARAVNLVDVGRRVLGEINPSYRSFFLNDIPPEVILVTDDLSPSDVAQLDCTKVHGLATAW
str.CO20_0297 MAEGHGVAWSWDRAVRQFADTLSKVDNPLLAARAVNLVDVGRRVLGEINPSYRSFFLNDIPPEVILVTDDLSPSDVAQLDCTKVHGLATAW
str.CO21_0024 MAEGHGVAWSWDRAVRQFADTLSKVDNPLLAARAVNLVDVGRRVLGEINPSYRSFFLNDIPPEVILVTDDLSPSDVAQLDCTKVHGLATAW
str.JK56 MAEGHGVAWSWDRAVRQFADTLSKVDNPLLAARAVNLVDVGRRVLGEINPSYRSFFLNDIPPEVILVTDDLSPSDVAQLDCTKVHGLATAW
str.RM-11 MAEGHGVAWSWDRAVRQFADMLSKVDNPLLAARAVNLVDVGRRVLGEINPSYRSFFLNDIPPEVILVTDDLSPSDVAQLDCTKVHGLATAW
str.MF1-1 MAEGHGVAWSWDRAVRQFADMLSKVDNPLLAARAVNLVDVGRRVLGEINPSYRSFFLNDIPPEVILVTDDLSPSDVAQLDCTKVHGLATAW
460 470 480 490 500 510 520 530 540
str.Toulouse GGPLSHTAILARTLGIPMVVAAGVDVLAIERDIQAIIDGDNGFIYLDPTLEDMEEAQKQMSIVAQKRDSGVCACRLPVQTTDGQRIRIMAN
str.G1712 GGPLSHTAILARTLGIPMVVAAGVDVLAIERDIQAIIDGDNGFIYLDPTLEDMEEAQKQMSIIAQKRDSGVCACRLPVQTTDGQRIRIMAN
str.G1713 GGPLSHTAILARTLGIPMVVAAGVDVLAIERDIQAIIDGDNGFIYLDPTLEDMEEAQKQMSIIAQKRDSGVCACRLPVQTTDGQRIRIMAN
str.CCUG45777 GGPLSHTAILARTLGIPMVVAAGVDVLAIERDIQAIIDGDNGFIYLDPTLEDMEEAQKQMSIIAQKRDSGVCACRLPVQTTDGQRIRIMAN
str.NCTC12899 GGPLSHTAILARTLGIPMVVAAGVDVLAIERDIQAIIDGDNGFIYLDPTLEDMEEAQKQMSIVAQKRDSGVCACRLPVQTTDGQRIRIMAN
str.CO20_0321 GGPLSHTAILARTLGIPMVVAAGVDVLAIERDIQAIIDGDNGFIYLDPTLEDMEEAQKQMSIVAQKRDSGVCACRLPVQTTDGQRIRIMAN
str.BQ2-D70 GGPLSHTAILARTLGIPMVVAAGVDVLAIERDIQAIIDGDNGFIYLDPTLEDMEEAQKQMSIVAQKRDSGVCACRLPVQTTDGQRIRIMAN
str.JK73rel GGPLSHTAILARTLGIPMVVAAGVDVLAIERDIQAIIDGDNGFIYLDPTLEDMEEAQKQMSIVAQKRDSGVCACRLPVQTTDGQRIRIMAN
str.JK7 GGPLSHTAILARTLGIPMVVAAGVDVLAIERDIQAIIDGDNGFIYLDPTLEDMEEAQKQMSIVAQKRDSGVCACRLPVQTTDGQRIRIMAN
str.JK73 GGPLSHTAILARTLGIPMVVAAGVDVLAIERDIQAIIDGDNGFIYLDPTLEDMEEAQKQMSIVAQKRDSGVCACRLPVQTTDGQRIRIMAN
str.JK31 GGPLSHTAILARTLGIPMVVAAGVDVLAIERDIQAIIDGDNGFIYLDPTLEDMEEAQKQMSIVAQKRDSGVCACRLPVQTTDGQRIRIMAN
str.JK68 GGPLSHTAILARTLGIPMVVAAGVDVLAIERDIQAIIDGDNGFIYLDPTLEDMEEAQKQMSIVAQKRDSGVCACRLPVQTTDGQRIRIMAN
str.JK39 GGPLSHTAILARTLGIPMVVAAGVDVLAIERDIQAIIDGDNGFIYLDPTLEDMEEAQKQMSIVAQKRDSGVCACRLPVQTTDGQRIRIMAN
str.JK63 GGPLSHTAILARTLGIPMVVAAGVDVLAIERDIQAIIDGDNGFIYLDPTLEDMEEAQKQMSIVAQKRDSGVCACRLPVQTTDGQRIRIMAN
str.JK19 GGPLSHTAILARTLGIPMVVAAGVDVLAIERDIQAIIDGDNGFIYLDPTLEDMEEAQKQMSIVAQKRDSGVCACRLPVQTTDGQRIRIMAN
str.JK67 GGPLSHTAILARTLGIPMVVAAGVDVLAIERDIQAIIDGDNGFIYLDPTLEDMEEAQKQMSIVAQKRDSGVCACRLPVQTTDGQRIRIMAN
str.JK12 GGPLSHTAILARTLGIPMVVAAGVDVLAIERDIQAIIDGDNGFIYLDPTLEDMEEAQKQMSIVAQKRDSGVCACRLPVQTTDGQRIRIMAN
str.CO20_0257 GGPLSHTAILARTLGIPMVVAAGVDVLAIERDIQAIIDGDNGFIYLDPTLEDMEEAQKQMSIVAQKRDSGVCACRLPVQTTDGQRIRIMAN
str.CO20_0256 GGPLSHTAILARTLGIPMVVAAGVDVLAIERDIQAIIDGDNGFIYLDPTLEDMEEAQKQMSIVAQKRDSGVCACRLPVQTTDGQRIRIMAN
str.CO20_0297 GGPLSHTAILARTLGIPMVVAAGVDVLAIERDIQAIIDGDNGFIYLDPTLEDMEEAQKQMSIVAQKRDSGVCACRLPVQTTDGQRIRIMAN
str.CO21_0024 GGPLSHTAILARTLGIPMVVAAGVDVLAIERDIQAIIDGDNGFIYLDPTLEDMEEAQKQMSIVAQKRDSGVCACRLPVQTTDGQRIRIMAN
str.JK56 GGPLSHTAILARTLGIPMVVAAGVDVLAIERDIQAIIDGDNGFIYLDPTLEDMEEAQKQMSIVAQKRDSGVCACRLPVQTTDGQRIRIMAN
str.RM-11 GGPLSHTAILARTLGIPMVVAAGVDVLAIERDIQAIIDGDNGFIYLHPTLEDMEEAQKQMSIVAQKRDSGVCACRLPVQTTDGQRIRIMAN
str.MF1-1 GGPLSHTAILARTLGIPMVVAAGVDVLAIERDIQAIIDGDNGFIYLHPTLEDMEEAQKQMSIVAQKRDSGVCACRLPVQTTDGQRIRIMAN
550 560 570 580 590 600 610 620 630
str.Toulouse VNYANQVPVALDLGAEGVGLMRTEFLFIDSPNIPDEDAQLDVYRAMIAAVGGDKPLIIRALDIGGDKQVTHLHLSKENNPFLGVRGTRLLL
str.G1712 VNYANQVPVALDLGAEGVGLMRTEFLFIDSPNIPDEDAQLDVYRAMIAAVGGDKPLIIRALDIGGDKQVTHLHLSKENNPFLGVRGTRLLL
str.G1713 VNYANQVPVALDLGAEGVGLMRTEFLFIDSPNIPDEDAQLDVYRAMIAAVGGDKPLIIRALDIGGDKQVTHLHLSKENNPFLGVRGTRLLL
str.CCUG45777 VNYANQVPVALDLGAEGVGLMRTEFLFIDSPNIPDEDAQLDVYRAMIAAVGGDKPLIIRALDIGGDKQVTHLHLSKENNPFLGVRGTRLLL
str.NCTC12899 VNYANQVPVALDLGAEGVGLMRTEFLFIDSPNIPDEDAQLDVYRAMIAAVGGDKPLIIRALDIGGDKQVTHLHLSKENNPFLGVRGTRLLL
str.CO20_0321 VNYANQVPVALDLGAEGVGLMRTEFLFIDSPNIPDEDAQLDVYRAMIAAVGGDKPLIIRALDIGGDKQVTHLHLSKENNPFLGVRGTRLLL
str.BQ2-D70 VNYANQVPVALDLGAEGVGLMRTEFLFIDSPNIPDEDAQLDVYRAMIAAVGGDKPLIIRALDIGGDKQVTHLHLSKENNPFLGVRGTRLLL
str.JK73rel VNYANQVPVALDLGAEGVGLMRTEFLFIDSPNIPDEDAQLDVYRAMIAAVGGDKPLIIRALDIGGDKQVTHLHLSKENNPFLGVRGTRLLL
str.JK7 VNYANQVPVALDLGAEGVGLMRTEFLFIDSPNIPDEDAQLDVYRAMIAAVGGDKPLIIRALDIGGDKQVTHLHLSKENNPFLGVRGTRLLL
str.JK73 VNYANQVPVALDLGAEGVGLMRTEFLFIDSPNIPDEDAQLDVYRAMIAAVGGDKPLIIRALDIGGDKQVTHLHLSKENNPFLGVRGTRLLL
str.JK31 VNYANQVPVALDLGAEGVGLMRTEFLFIDSPNIPDEDAQLDVYRAMIAAVGGDKPLIIRALDIGGDKQVTHLHLSKENNPFLGVRGTRLLL
str.JK68 VNYANQVPVALDLGAEGVGLMRTEFLFIDSPNIPDEDAQLDVYRAMIAAVGGDKPLIIRALDIGGDKQVTHLHLSKENNPFLGVRGTRLLL
str.JK39 VNYANQVPVALDLGAEGVGLMRTEFLFIDSPNIPDEDAQLDVYRAMIAAVGGDKPLIIRALDIGGDKQVTHLHLSKENNPFLGVRGTRLLL
str.JK63 VNYANQVPVALDLGAEGVGLMRTEFLFIDSPNIPDEDAQLDVYRAMIAAVGGDKPLIIRALDIGGDKQVTHLHLSKENNPFLGVRGTRLLL
str.JK19 VNYANQVPVALDLGAEGVGLMRTEFLFIDSPNIPDEDAQLDVYRAMIAAVGGDKPLIIRALDIGGDKQVTHLHLSKENNPFLGVRGTRLLL
str.JK67 VNYANQVPVALDLGAEGVGLMRTEFLFIDSPNIPDEDAQLDVYRAMIAAVGGDKPLIIRALDIGGDKQVTHLHLSKENNPFLGVRGTRLLL
str.JK12 VNYANQVPVALDLGAEGVGLMRTEFLFIDSPNIPDEDAQLDVYRAMIAAVGGDKPLIIRALDIGGDKQVTHLHLSKENNPFLGVRGTRLLL
str.CO20_0257 VNYANQVPVALDLGAEGVGLMRTEFLFIDSPNIPDEDAQLDVYRAMIAAVGGDKPLIIRALDIGGDKQVTHLHLSKENNPFLGVRGTRLLL
str.CO20_0256 VNYANQVPVALDLGAEGVGLMRTEFLFIDSPNIPDEDAQLDVYRAMIAAVGGDKPLIIRALDIGGDKQVTHLHLSKENNPFLGVRGTRLLL
str.CO20_0297 VNYANQVPVALDLGAEGVGLMRTEFLFIDSPNIPDEDAQLDVYRAMIAAVGGDKPLIIRALDIGGDKQVTHLHLSKENNPFLGVRGTRLLL
str.CO21_0024 VNYANQVPVALDLGAEGVGLMRTEFLFIDSPNIPDEDAQLDVYRAMIAAVGGDKPLIIRALDIGGDKQVTHLHLSKENNPFLGVRGTRLLL
str.JK56 VNYANQVPVALDLGAEGVGLMHTEFLFIDSPNIPDEDAQLDVYRAMIAAVGGDKPLIIRALDIGGDKQVTHLHLSKENNPFLGVRGTRLLL
str.RM-11 VNYANQVPVALDLGAEGVGLMHTEFLFIDSPNIPDEDAQLDVYRAMIAAVGGDKPLIIRALDIGGDKQVTHLHLSKENNPFLGVRGTRLLL
str.MF1-1 VNYANQVPVALDLGAEGVGLMHTEFLFIDSPNIPDEDAQLDVYRAMIAAVGGDKPLIIRALDIGGDKQVTHLHLSKENNPFLGVRGTRLLL
640 650 660 670 680 690 700 710 720
str.Toulouse RRRDLLVPQLRALYRAAKEGGDLWILFPMVMSVSEIFAMKKIAEEIQNDIGAPKLKFGIMIEVPAAAIMADILSAHVDFFSIGTNDLTQYT
str.G1712 RRRDLLVPQLRALYRAAKEGGDLWILFPMVMSVSEIFAMKKIAEEIQNDIGAPKLKFGIMIEVPAAAIMADILSAHVDFFSIGTNDLTQYT
str.G1713 RRRDLLVPQLRALYRAAKEGGDLWILFPMVMSVSEIFAMKKIAEEIQNDIGAPKLKFGIMIEVPAAAIMADILSAHVDFFSIGTNDLTQYT
str.CCUG45777 RRRDLLVPQLRALYRAAKEGGDLWILFPMVMSVSEIFAMKKIAEEIQNDIGAPKLKFGIMIEVPAAAIMADILSAHVDFFSIGTNDLTQYT
str.NCTC12899 RRRDLLVPQLRALYRAAKEGGDLWILFPMVMSVSEIFAMKKIAEEIQNDIGAPKLKFGIMIEVPAAAIMADILSAHVDFFSIGTNDLTQYT
str.CO20_0321 RRRDLLVPQLRALYRAAKEGGDLWILFPMVMSVSEIFAMKKIAEEIQNDIGAPKLKFGIMIEVPAAAIMADILSAHVDFFSIGTNDLTQYT
str.BQ2-D70 RRRDLLVPQLRALYRAAKEGGDLWILFPMVMSVSEIFAMKKIAEEIQNDIGAPKLKFGIMIEVPAAAIMADILSAHVDFFSIGTNDLTQYT
str.JK73rel RRRDLLVPQLRALYRAAKEGGDLWILFPMVMSVSEIFAMKKIAEEIQNDIGAPKLKFGIMIEVPAAAIMADILSAHVDFFSIGTNDLTQYT
str.JK7 RRRDLLVPQLRALYRAAKEGGDLWILFPMVMSVSEIFAMKKIAEEIQNDIGAPKLKFGIMIEVPAAAIMADILSAHVDFFSIGTNDLTQYT
str.JK73 RRRDLLVPQLRALYRAAKEGGDLWILFPMVMSVSEIFAMKKIAEEIQNDIGAPKLKFGIMIEVPAAAIMADILSAHVDFFSIGTNDLTQYT
str.JK31 RRRDLLVPQLRALYRAAKEGGDLWILFPMVMSVSEIFAMKKIAEEIQNDIGAPKLKFGIMIEVPAAAIMADILSAHVDFFSIGTNDLTQYT
str.JK68 RRRDLLVPQLRALYRAAKEGGDLWILFPMVMSVSEIFAMKKIAEEIQNDIGAPKLKFGIMIEVPAAAIMADILSAHVDFFSIGTNDLTQYT
str.JK39 RRRDLLVPQLRALYRAAKEGGDLWILFPMVMSVSEIFAMKKIAEEIQNDIGAPKLKFGIMIEVPAAAIMADILSAHVDFFSIGTNDLTQYT
str.JK63 RRRDLLVPQLRALYRAAKEGGDLWILFPMVMSVSEIFAMKKIAEEIQNDIGAPKLKFGIMIEVPAAAIMADILSAHVDFFSIGTNDLTQYT
str.JK19 RRRDLLVPQLRALYRAAKEGGDLWILFPMVMSVSEIFAMKKIAEEIQNDIGAPKLKFGIMIEVPAAAIMADILSAHVDFFSIGTNDLTQYT
str.JK67 RRRDLLVPQLRALYRAAKEGGDLWILFPMVMSVSEIFAMKKIAEEIQNDIGAPKLKFGIMIEVPAAAIMADILSAHVDFFSIGTNDLTQYT
str.JK12 RRRDLLVPQLRALYRAAKEGGDLWILFPMVMSVSEIFAMKKIAEEIQNDIGAPKLKFGIMIEVPAAAIMADILSAHVDFFSIGTNDLTQYT
str.CO20_0257 RRRDLLVPQLRALYRAAKEGGDLWILFPMVMSVSEIFAMKKIAEEIQNDIGAPKLKFGIMIEVPAAAIMADILSAHVDFFSIGTNDLTQYT
str.CO20_0256 RRRDLLVPQLRALYRAAKEGGDLWILFPMVMSVSEIFAMKKIAEEIQNDIGAPKLKFGIMIEVPAAAIMADILSAHVDFFSIGTNDLTQYT
str.CO20_0297 RRRDLLVPQLRALYRAAKEGGDLWILFPMVMSVSEIFAMKKIAEEIQNDIGAPKLKFGIMIEVPAAAIMADILSAHVDFFSIGTNDLTQYT
str.CO21_0024 RRRDLLVPQLRALYRAAKEGGDLWILFPMVMSVSEIFAMKKIAEEIQNDIGAPKLKFGIMIEVPAAAIMADILSAHVDFFSIGTNDLTQYT
str.JK56 RRRDLLVPQLRALYRAAKEGGDLWILFPMVMSVSEIFAMKKIAEEIQNDIGAPKLKFGIMIEVPAAAIMADILSAHVDFFSIGTNDLTQYT
str.RM-11 RRRDLLVPQLRALYRAAKEGGDLWILFPMVMSVSEIFAMKKIAEEIQNDIGAPKLKFGIMIEVPAAAIMADILSAHVDFFSIGTNDLTQYT
str.MF1-1 RRRDLLVPQLRALYRAAKEGGDLWILFPMVMSVSEIFAMKKIAEEIQNDIGAPKLKFGIMIEVPAAAIMADILSAHVDFFSIGTNDLTQYT

```
                  730       740       750       760       770       780       790       800       810
str.Toulouse      MAVDRQNPHLVSEADSLDPAVLRMINYTIQGAAKHKCWVSVCGGMAGDPFAAMILTGLGIHELSMISCDISSVKACLQAHSFEDMKILAKK
str.G1712         MAVDRQNPHLVSEADSLDPAVLRMINYTIQGAAKHKCWVSVCGGMAGDPFAAMILTGLGIHELSMISCDISSVKACLQAHSFEDMKILAKK
str.G1713         MAVDRQNPHLVSEADSLDPAVLRMINYTIQGAAKHKCWVSVCGGMAGDPFAAMILTGLGIHELSMISCDISSVKACLQAHSFEDMKILAKK
str.CCUG45777     MAVDRQNPHLVSEADSLDPAVLRMINYTIQGAAKHKCWVSVCGGMAGDPFAAMILTGLGIHELSMISCDISSVKACLQAHSFEDMKILAKK
str.NCTC12899     MAVDRQNPHLVSEADSLDPAVLRMINYTIQGAAKHKCWVSVCGGMAGDPFAAMILTGLGIHELSMISCDISSVKACLQAHSFEDMKILAKK
str.CO20_0321     MAVDRQNPHLVSEADSLDPAVLRMINYTIQGAAKHKCWVSVCGGMAGDPFAAMILTGLGIHELSMISCDISSVKACLQAHSFEDMKILAKK
str.BQ2-D70       MAVDRQNPHLVSEADSLDPAVLRMINYTIQGAAKHKCWVSVCGGMAGDPFAAMILTGLGIHELSMISCDISSVKACLQAHSFEDMKILAKK
str.JK73rel       MAVDRQNPHLVSEADSLDPAVLRMINYTIQGAAKHKCWVSVCGGMAGDPFAAMILTGLGIHELSMISCDISSVKACLQAHSFEDMKILAKK
str.JK7           MAVDRQNPHLVSEADSLDPAVLRMINYTIQGAAKHKCWVSVCGGMAGDPFAAMILTGLGIHELSMISCDISSVKACLQAHSFEDMKILAKK
str.JK73          MAVDRQNPHLVSEADSLDPAVLRMINYTIQGAAKHKCWVSVCGGMAGDPFAAMILTGLGIHELSMISCDISSVKACLQAHSFEDMKILAKK
str.JK31          MAVDRQNPHLVSEADSLDPAVLRMINYTIQGAAKHKCWVSVCGGMAGDPFAAMILTGLGIHELSMISCDISSVKACLQAHSFEDMKILAKK
str.JK68          MAVDRQNPHLVSEADSLDPAVLRMINYTIQGAAKHKCWVSVCGGMAGDPFAAMILTGLGIHELSMISCDISSVKACLQAHSFEDMKILAKK
str.JK39          MAVDRQNPHLVSEADSLDPAVLRMINYTIQGAAKHKCWVSVCGGMAGDPFAAMILTGLGIHELSMISCDISSVKACLQAHSFEDMKILAKK
str.JK63          MAVDRQNPHLVSEADSLDPAVLRMINYTIQGAAKHKCWVSVCGGMAGDPFAAMILTGLGIHELSMISCDISSVKACLQAHSFEDMKILAKK
str.JK19          MAVDRQNPHLVSEADSLDPAVLRMINYTIQGAAKHKCWVSVCGGMAGDPFAAMILTGLGIHELSMISCDISSVKACLQAHSFEDMKILAKK
str.JK67          MAVDRQNPHLVSEADSLDPAVLRMINYTIQGAAKHKCWVSVCGGMAGDPFAAMILTGLGIHELSMISCDISSVKACLQAHSFEDMKILAKK
str.JK12          MAVDRQNPHLVSEADSLDPAVLRMINYTIQGAAKHKCWVSVCGGMAGDPFAAMILTGLGIHELSMISCDISSVKACLQAHSFEDMKILAKK
str.CO20_0257     MAVDRQNPHLVSEADSLDPAVLRMINYTIQGAAKHKCWVSVCGGMAGDPFAAMILTGLGIHELSMISCDISSVKACLQAHSFEDMKILAKK
str.CO20_0256     MAVDRQNPHLVSEADSLDPAVLRMINYTIQGAAKHKCWVSVCGGMAGDPFAAMILTGLGIHELSMISCDISSVKACLQAHSFEDMKILAKK
str.CO20_0297     MAVDRQNPHLVSEADSLDPAVLRMINYTIQGAAKHKCWVSVCGGMAGDPFAAMILTGLGIHELSMISCDISSVKACLQAHSFEDMKILAKK
str.CO21_0024     MAVDRQNPHLVSEADSLDPAVLRMINYTIQGAAKHKCWVSVCGGMAGDPFAAMILTGLGIHELSMISCDISSVKACLQAHSFEDMKILAKK
str.JK56          MAVDRQNPHLVSEADSLDPAVLRMINYTIQGAAKHKCWVSVCGGMAGDPFAAMILTGLGIHELSMISCDISSVKACLQAHSFEDMKILAKK
str.RM-11         MAVDRQNPHLVSEADSLDPAVLRMINYTIQGAAKHKCWVSVCGGMAGDPFAAMILTGLGIHELSMISCDISSVKACLQAHSFEDMKILAKK
str.MF1-1         MAVDRQNPHLVSEADSLDPAVLRMINYTIQGAAKHKCWVSVCGGMAGDPFAAMILTGLGIHELSMISCDISSVKACLQAHSFEDMKILAKK

                820       830
str.Toulouse      ALQCETAGAVRALRNDIR
str.G1712         ALQCETAGAVRALRND..
str.G1713         ALQCETAGAVRALRND..
str.CCUG45777     ALQCETAGAVRALRNDIR
str.NCTC12899     ALQCETAGAVRALRNDIR
str.CO20_0321     ALQCETAGAVRALRNDIR
str.BQ2-D70       ALQCETAGAVRALRNDIR
str.JK73rel       ALQCETAGAVRALRNDIR
str.JK7           ALQCETAGAVRALRNDIR
str.JK73          ALQCETAGAVRALRNDIR
str.JK31          ALQCETAGAVRALRNDIR
str.JK68          ALQCETAGAVRALRNDIR
str.JK39          ALQCETAGAVRALRNDIR
str.JK63          ALQCETAGAVRALRNDIR
str.JK19          ALQCETAGAVRALRNDIR
str.JK67          ALQCETAGAVRALRNDIR
str.JK12          ALQCETAGAVRALRNDIR
str.CO20_0257     ALQCETAGAVRALRNDIR
str.CO20_0256     ALQCETAGAVRALRNDIR
str.CO20_0297     ALQCETAGAVRALRNDIR
str.CO21_0024     ALQCETAGAVRALRNDIR
str.JK56          ALQCETAGAVRALRNDIR
str.RM-11         ALQCETAGAVRALRNDIR
str.MF1-1         ALQCETAGAVRALRNDIR
```

>WP_011179066.1 lipid IV(A) 3-deoxy-D-manno-octulosonic acid transferase [Bartonella quintana str. Toulouse]

```
              1        10        20        30        40        50        60        70        80        90
str.Toulouse  MVELKAHAALLIYRMIGFCLRPLVPFYLFFRAIRGKEEWNRKKERLGKSHQVRPQSPLIWLHAASVGETLALFPLINYILSLKINVLLTTG
str.JK12      MVELKAHAALLIYRMIGFCLRPLVPFYLFFRAIRGKEEWNRKKERLGKSHQVRPQSPLIWLHAASVGETLALFPLINYILSLKINVLLTTG
str.CCUG45777 MVELKAHAALLIYRMIGFCLRPLVPFYLFFRAIRGKEEWNRKKERLGKSHQVRPQSPLIWLHAASVGETLALFPLINYILSLKINVLLTTG
str.JK56      MVELKAHAALLIYRMIGFCLRPLVPFYLFFRAIRGKEEWNRKKERLGKSHQVRPQSPLIWLHAASVGETLALFPLINYILSLKINVLLTTG
str.JK67      MVELKAHAALLIYRMIGFCLRPLVPFYLFFRAIRGKEEWNRKKERLGKSHQVRPQSPLIWLHAASVGETLALFPLINYILSLKINVLLTTG
str.JK63      MVELKAHAALLIYRMIGFCLRPLVPFYLFFRAIRGKEEWNRKKERLGKSHQVRPQSPLIWLHAASVGETLALFPLINYILSLKINVLLTTG
str.JK19      MVELKAHAALLIYRMIGFCLRPLVPFYLFFRAIRGKEEWNRKKERLGKSHQVRPQSPLIWLHAASVGETLALFPLINYILSLKINVLLTTG
str.JK39      MVELKAHAALLIYRMIGFCLRPLVPFYLFFRAIRGKEEWNRKKERLGKSHQVRPQSPLIWLHAASVGETLALFPLINYILSLKINVLLTTG
str.JK68      MVELKAHAALLIYRMIGFCLRPLVPFYLFFRAIRGKEEWNRKKERLGKSHQVRPQSPLIWLHAASVGETLALFPLINYILSLKINVLLTTG
str.JK31      MVELKAHAALLIYRMIGFCLRPLVPFYLFFRAIRGKEEWNRKKERLGKSHQVRPQSPLIWLHAASVGETLALFPLINYILSLKINVLLTTG
str.G1713     MVELKAHAALLIYRMIGFCLRPLVPFYLFFRAIRGKEEWNRKKERLGKSHQVRPQSPLIWLHAASVGETLALFPLINYILSLKINVLLTTG
str.G1712     MVELKAHAALLIYRMIGFCLRPLVPFYLFFRAIRGKEEWNRKKERLGKSHQVRPQSPLIWLHAASVGETLALFPLINYILSLKINVLLTTG
str.JK73      MVELKAHAALLIYRMIGFCLRPLVPFYLFFRAIRGKEEWNRKKERLGKSHQVRPQSPLIWLHAASVGETLALFPLINYILSLKINVLLTTG
str.JK7       MVELKAHAALLIYRMIGFCLRPLVPFYLFFRAIRGKEEWNRKKERLGKSHQVRPQSPLIWLHAASVGETLALFPLINYILSLKINVLLTTG
str.JK73rel   MVELKAHAALLIYRMIGFCLRPLVPFYLFFRAIRGKEEWNRKKERLGKSHQVRPQSPLIWLHAASVGETLALFPLINYILSLKINVLLTTG
str.BQ2-D70   MVELKAHAALLIYRMIGFCLRPLVPFYLFFRAIRGKEEWNRKKERLGKSHQVRPQSPLIWLHAASVGETLALFPLINYILSLKINVLLTTG
str.CO20_0321 MVELKAHAALLIYRMIGFCLRPLVPFYLFFRAIRGKEEWNRKKERLGKSHQVRPQSPLIWLHAASVGETLALFPLINYILSLKINVLLTTG
str.CO21_0024 MVELKAHAALLIYRMIGFCLRPLVPFYLFFRAIRGKEEWNRKKERLGKSHQVRPQSPLIWLHAASVGETLALFPLINYILSLKINVLLTTG
str.CO20_0297 MVELKAHAALLIYRMIGFCLRPLVPFYLFFRAIRGKEEWNRKKERLGKSHQVRPQSPLIWLHAASVGETLALFPLINYILSLKINVLLTTG
str.CO20_0256 MVELKAHAALLIYRMIGFCLRPLVPFYLFFRAIRGKEEWNRKKERLGKSHQVRPQSPLIWLHAASVGETLALFPLINYILSLKINVLLTTG
str.CO20_0257 MVELKAHAALLIYRMIGFCLRPLVPFYLFFRAIRGKEEWNRKKERLGKSHQVRPQSPLIWLHAASVGETLALFPLINYILSLKINVLLTTG
str.NCTC12899 MVELKAHAALLIYRMIGFCLRPLVPFYLFFRAIRGKEEWNRKKERLGKSHQVRPQSPLIWLHAASVGETLALFPLINYILSLKINVLLTTG
str.RM-11     MVELKAHAALLIYRMIGFCLRPLVPFYLFFRAIRGKEEWDRKKERLGKSHQVRPQSPLIWLHAASVGETLALFPLINYILSLKINVLLTTG
str.MF1-1     MVELKAHAALLIYRMIGFCLRPLVPFYLFFRAIRGKEEWDRKKERLGKSHQVRPQSPLIWLHAASVGETLALFPLINYILSLKINVLLTTG
```

```
                      100       110       120       130       140       150       160       170       180
str.Toulouse  TVTSSYLVRKHFDDRLIHQYAPLDLDLAVRRFISHWKPDLALTCESEIWPLRIKELAKMRIPQILVNAHMSERSFKAWQKRRILARHIFKH
str.JK12      TVTSSYLVRKHFDDRLIHQYAPLDLDLAVRRFISHWKPDLALTCESEIWPLRIKELAKMRIPQILVNAHMSERSFKAWQKRRILARHIFKH
str.CCUG45777 TVTSSYLVRKHFDDRLIHQYAPLDLDLAVRRFISHWKPDLALTCESEIWPLRIKELAKMRIPQILVNAHMSERSFKAWQKRRILARHIFKH
str.JK56      TVTSSYLVRKHFDDRLIHQYAPLDLDLAVRRFISHWKPDLALTCESEIWPLRIKELAKMRIPQILVNAHMSERSFKAWQKRRILARHIFKH
str.JK67      TVTSSYLVRKHFDDRLIHQYAPLDLDLAVRRFISHWKPDLALTCESEIWPLRIKELAKMRIPQILVNAHMSERSFKAWQKRRILARHIFKH
str.JK63      TVTSSYLVRKHFDDRLIHQYAPLDLDLAVRRFISHWKPDLALTCESEIWPLRIKELAKMRIPQILVNAHMSERSFKAWQKRRILARHIFKH
str.JK19      TVTSSYLVRKHFDDRLIHQYAPLDLDLAVRRFISHWKPDLALTCESEIWPLRIKELAKMRIPQILVNAHMSERSFKAWQKRRILARHIFKH
str.JK39      TVTSSYLVRKHFDDRLIHQYAPLDLDLAVRRFISHWKPDLALTCESEIWPLRIKELAKMRIPQILVNAHMSERSFKAWQKRRILARHIFKH
str.JK68      TVTSSYLVRKHFDDRLIHQYAPLDLDLAVRRFISHWKPDLALTCESEIWPLRIKELAKMRIPQILVNAHMSERSFKAWQKRRILARHIFKH
str.JK31      TVTSSYLVRKHFDDRLIHQYAPLDLDLAVRRFISHWKPDLALTCESEIWPLRIKELAKMRIPQILVNAHMSERSFKAWQKRRILARHIFKH
str.G1713     TVTSSYLVRKHFDDRLIHQYAPLDLDLAVRRFISHWKPDLALTCESEIWPLRIKELAKMRIPQILVNAHMSERSFKAWQKRRILARHIFKH
str.G1712     TVTSSYLVRKHFDDRLIHQYAPLDLDLAVRRFISHWKPDLALTCESEIWPLRIKELAKMRIPQILVNAHMSERSFKAWQKRRILARHIFKH
str.JK73      TVTSSYLVRKHFDDRLIHQYAPLDLDLAVRRFISHWKPDLALTCESEIWPLRIKELAKMRIPQILVNAHMSERSFKAWQKRRILARHIFKH
str.JK7       TVTSSYLVRKHFDDRLIHQYAPLDLDLAVRRFISHWKPDLALTCESEIWPLRIKELAKMRIPQILVNAHMSERSFKAWQKRRILARHIFKH
str.JK73rel   TVTSSYLVRKHFDDRLIHQYAPLDLDLAVRRFISHWKPDLALTCESEIWPLRIKELAKMRIPQILVNAHMSERSFKAWQKRRILARHIFKH
str.BQ2-D70   TVTSSYLVRKHFDDRLIHQYAPLDLDLAVRRFISHWKPDLALTCESEIWPLRIKELAKMRIPQILVNAHMSERSFKAWQKRRILARHIFKH
str.CO20_0321 TVTSSYLVRKHFDDRLIHQYAPLDLDLAVRRFISHWKPDLALTCESEIWPLRIKELAKMRIPQILVNAHMSERSFKAWQKRRILARHIFKH
str.CO21_0024 TVTSSYLVRKHFDDRLIHQYAPLDLDLAVRRFISHWKPDLALTCESEIWPLRIKELAKMRIPQILVNAHMSERSFKAWQKRRILARHIFKH
str.CO20_0297 TVTSSYLVRKHFDDRLIHQYAPLDLDLAVRRFISHWKPDLALTCESEIWPLRIKELAKMRIPQILVNAHMSERSFKAWQKRRILARHIFKH
str.CO20_0256 TVTSSYLVRKHFDDRLIHQYAPLDLDLAVRRFISHWKPDLALTCESEIWPLRIKELAKMRIPQILVNAHMSERSFKAWQKRRILARHIFKH
str.CO20_0257 TVTSSYLVRKHFDDRLIHQYAPLDLDLAVRRFISHWKPDLALTCESEIWPLRIKELAKMRIPQILVNAHMSERSFKAWQKRRILARHIFKH
str.NCTC12899 TVTSSYLVRKHFDDRLIHQYAPLDLDLAVRRFISHWKPDLALTCESEIWPLRIKELAKMRIPQILVNAHMSERSFKAWQKRRILARHIFKH
str.RM-11     TVTSSYLVRKHFDDRLIHQYAPLDLDLAVRRFISHWKPDLALTCESEIWPLRIKELAKMRIPQILVNAHMSERSFKAWQKRRILARHIFKH
str.MF1-1     TVTSSYLVRKHFDDRLIHQYAPLDLDLAVRRFISHWKPDLALTCESEIWPLRIKELAKMRIPQILVNAHMSERSFKAWQKRRILARHIFKH
```

```
                     190       200       210       220       230       240       250       260       270
str.Toulouse  IDLAIAQNERDVAYYRALGIKSVALSGNLKADVFWAEDQALLAYYRAAIGNRPVWAAVSTHEGEEEIAFEVHKILKNYFPDLLTIIVPRHP
str.JK12      IDLAIAQNERDVAYYRALGIKSVALSGNLKADVFWAEDQALLAYYRAAIGNRPVWAAVSTHEGEEEIAFEVHKILKNYFPDLLTIIVPRHP
str.CCUG45777 IDLAIAQNERDVAYYRALGIKSVALSGNLKADVFWAEDQALLAYYRAAIGNRPVWAAVSTHEGEEEIAFEVHKILKNYFPDLLTIIVPRHP
str.JK56      IDLAIAQNERDVAYYRALGIKSVALSGNLKADVFWAEDQALLAYYRAAIGNRPVWAAVSTHEGEEEIAFEVHKILKNYFPDLLTIIVPRHP
str.JK67      IDLAIAQNERDVAYYRALGIKSVALSGNLKADVFWAEDQALLAYYRAAIGNRPVWAAVSTHEGEEEIAFEVHKILKNYFPDLLTIIVPRHP
str.JK63      IDLAIAQNERDVAYYRALGIKSVALSGNLKADVFWAEDQALLAYYRAAIGNRPVWAAVSTHEGEEEIAFEVHKILKNYFPDLLTIIVPRHP
str.JK19      IDLAIAQNERDVAYYRALGIKSVALSGNLKADVFWAEDQALLAYYRAAIGNRPVWAAVSTHEGEEEIAFEVHKILKNYFPDLLTIIVPRHP
str.JK39      IDLAIAQNERDVAYYRALGIKSVALSGNLKADVFWAEDQALLAYYRAAIGNRPVWAAVSTHEGEEEIAFEVHKILKNYFPDLLTIIVPRHP
str.JK68      IDLAIAQNERDVAYYRALGIKSVALSGNLKADVFWAEDQALLAYYRAAIGNRPVWAAVSTHEGEEEIAFEVHKILKNYFPDLLTIIVPRHP
str.JK31      IDLAIAQNERDVAYYRALGIKSVALSGNLKADVFWAEDQALLAYYRAAIGNRPVWAAVSTHEGEEEIAFEVHKILKNYFPDLLTIIVPRHP
str.G1713     IDLAIAQNERDVAYYRALGIKSVALSGNLKADVFWAEDQALLAYYRAAIGNRPVWAAVSTHEGEEEIAFEVHKILKNYFPDLLTIIVPRHP
str.G1712     IDLAIAQNERDVAYYRALGIKSVALSGNLKADVFWAEDQALLAYYRAAIGNRPVWAAVSTHEGEEEIAFEVHKILKNYFPDLLTIIVPRHP
str.JK73      IDLAIAQNERDVAYYRALGIKSVALSGNLKADVFWAEDQALLAYYRAAIGNRPVWAAVSTHEGEEEIAFEVHKILKNYFPDLLTIIVPRHP
str.JK7       IDLAIAQNERDVAYYRALGIKSVALSGNLKADVFWAEDQALLAYYRAAIGNRPVWAAVSTHEGEEEIAFEVHKILKNYFPDLLTIIVPRHP
str.JK73rel   IDLAIAQNERDVAYYRALGIKSVALSGNLKADVFWAEDQALLAYYRAAIGNRPVWAAVSTHEGEEEIAFEVHKILKNYFPDLLTIIVPRHP
str.BQ2-D70   IDLAIAQNERDVAYYRALGIKSVALSGNLKADVFWAEDQALLAYYRAAIGNRPVWAAVSTHEGEEEIAFEVHKILKNYFPDLLTIIVPRHP
str.CO20_0321 IDLAIAQNERDVAYYRALGIKSVALSGNLKADVFWAEDQALLAYYRAAIGNRPVWAAVSTHEGEEEIAFEVHKILKNYFPDLLTIIVPRHP
str.CO21_0024 IDLAIAQNERDVAYYRALGIKSVALSGNLKADVFWAEDQALLAYYRAAIGNRPVWAAVSTHEGEEEIAFEVHKILKNYFPDLLTIIVPRHP
str.CO20_0297 IDLAIAQNERDVAYYRALGIKSVALSGNLKADVFWAEDQALLAYYRAAIGNRPVWAAVSTHEGEEEIAFEVHKILKNYFPDLLTIIVPRHP
str.CO20_0256 IDLAIAQNERDVAYYRALGIKSVALSGNLKADVFWAEDQALLAYYRAAIGNRPVWAAVSTHEGEEEIAFEVHKILKNYFPDLLTIIVPRHP
str.CO20_0257 IDLAIAQNERDVAYYRALGIKSVALSGNLKADVFWAEDQALLAYYRAAIGNRPVWAAVSTHEGEEEIAFEVHKILKNYFPDLLTIIVPRHP
str.NCTC12899 IDLAIAQNERDVAYYRALGIKSVALSGNLKADVFWAEDQALLAYYRAAIGNRPVWAAVSTHEGEEEIAFEVHKILKNYFPDLLTIIVPRHP
str.RM-11     IDLAIAQNERDVAYYRALGIKSVALSGNLKADVFWAEDQALLAYYRAAIGNRPVWAAVSTHEGEEEIAFEVHKILKNYFPDLLTIIVPRHP
str.MF1-1     IDLAIAQNERDVAYYRALGIKSVTLSGNLKADVFWAEDQALLAYYRAAIGNRPVWAAVSTHEGEEEIAFEVHKILKNYFPDLLTIIVPRHP
```

```
                    280       290       300       310       320       330       340       350       360
str.Toulouse  ERSEDLIKKCDNKSLRFIRRSNNAIPARDTDVLLGDTIGEMGLFLRLSKVSFIGKSLCGDGGHNPLELALLGSAILTGPHISNFQEMFEQF
str.JK12      ERSEDLIKKCDNKSLRFIRRSNNAIPARDTDVLLGDTIGEMGLFLRLSKVSFIGKSLCGDGGHNPLELALLGSAILTGPHISNFQEMFEQF
str.CCUG45777 ERSEDLIKKCDNKSLRFIRRSNNAIPARDTDVLLGDTIGEMGLFLRLSKVSFIGKSLCGDGGHNPLELALLGSAILTGPHISNFQEMFEQF
str.JK56      ERSEDLIKKCDNKSLRFIRRSNNAIPARDTDVLLGDTIGEMGLFLRLSKVSFIGKSLCGDGGHNPLELALLGSAILTGPHISNFQEMFEQF
str.JK67      ERSEDLIKKCDNKSLRFIRRSNNAIPARDTDVLLGDTIGEMGLFLRLSKVSFIGKSLCGDGGHNPLELALLGSAILTGPHISNFQEMFEQF
str.JK63      ERSEDLIKKCDNKSLRFIRRSNNAIPARDTDVLLGDTIGEMGLFLRLSKVSFIGKSLCGDGGHNPLELALLGSAILTGPHISNFQEMFEQF
str.JK19      ERSEDLIKKCDNKSLRFIRRSNNAIPARDTDVLLGDTIGEMGLFLRLSKVSFIGKSLCGDGGHNPLELALLGSAILTGPHISNFQEMFEQF
str.JK39      ERSEDLIKKCDNKSLRFIRRSNNAIPARDTDVLLGDTIGEMGLFLRLSKVSFIGKSLCGDGGHNPLELALLGSAILTGPHISNFQEMFEQF
str.JK68      ERSEDLIKKCDNKSLRFIRRSNNAIPARDTDVLLGDTIGEMGLFLRLSKVSFIGKSLCGDGGHNPLELALLGSAILTGPHISNFQEMFEQF
str.JK31      ERSEDLIKKCDNKSLRFIRRSNNAIPARDTDVLLGDTIGEMGLFLRLSKVSFIGKSLCGDGGHNPLELALLGSAILTGPHISNFQEMFEQF
str.G1713     ERSEDLIKKCDNKSLRFIRRSNNAIPARDTDVLLGDTIGEMGLFLRLSKVSFIGKSLCGDGGHNPLELALLGSAILTGPHISNFQEMFEQF
str.G1712     ERSEDLIKKCDNKSLRFIRRSNNAIPARDTDVLLGDTIGEMGLFLRLSKVSFIGKSLCGDGGHNPLELALLGSAILTGPHISNFQEMFEQF
str.JK73      ERSEDLIKKCDNKSLRFIRRSNNAIPARDTDVLLGDTIGEMGLFLRLSKVSFIGKSLCGDGGHNPLELALLGSAILTGPHISNFQEMFEQF
str.JK7       ERSEDLIKKCDNKSLRFIRRSNNAIPARDTDVLLGDTIGEMGLFLRLSKVSFIGKSLCGDGGHNPLELALLGSAILTGPHISNFQEMFEQF
str.JK73rel   ERSEDLIKKCDNKSLRFIRRSNNAIPARDTDVLLGDTIGEMGLFLRLSKVSFIGKSLCGDGGHNPLELALLGSAILTGPHISNFQEMFEQF
str.BQ2-D70   ERSEDLIKKCDNKSLRFIRRSNNAIPARDTDVLLGDTIGEMGLFLRLSKVSFIGKSLCGDGGHNPLELALLGSAILTGPHISNFQEMFEQF
str.CO20_0321 ERSEDLIKKCDNKSLRFIRRSNNAIPARDTDVLLGDTIGEMGLFLRLSKVSFIGKSLCGDGGHNPLELALLGSAILTGPHISNFQEMFEQF
str.CO21_0024 ERSEDLIKKCDNKSLRFIRRSNNAIPARDTDVLLGDTIGEMGLFLRLSKVSFIGKSLCGDGGHNPLELALLGSAILTGPHISNFQEMFEQF
str.CO20_0297 ERSEDLIKKCDNKSLRFIRRSNNAIPARDTDVLLGDTIGEMGLFLRLSKVSFIGKSLCGDGGHNPLELALLGSAILTGPHISNFQEMFEQF
str.CO20_0256 ERSEDLIKKCDNKSLRFIRRSNNAIPARDTDVLLGDTIGEMGLFLRLSKVSFIGKSLCGDGGHNPLELALLGSAILTGPHISNFQEMFEQF
str.CO20_0257 ERSEDLIKKCDNKSLRFIRRSNNAIPARDTDVLLGDTIGEMGLFLRLSKVSFIGKSLCGDGGHNPLELALLGSAILTGPHISNFQEMFEQF
str.NCTC12899 ERSEDLIKKCDNKSLRFIRRSNNAIPARDTDVLLGDTIGEMGLFLRLSKVSFIGKSLCGDGGHNPLELALLGSAILTGPHISNFQEMFEQF
str.RM-11     ERSEDLIKKCDNKSLRFIRRSNNAIPARDTDVLLGDTIGEMGLFLRLSKVSFIGKSLCGDGGHNPLELALLGSAILTGPHISNFQEMFEQF
str.MF1-1     ERSEDLIKKCDNKSLRFIRRSNNAIPARDTDVLLGDTIGEMGLFLRLSKVSFIGKSLCGDGGHNPLELALLGSAILTGPHISNFQEMFEQF
```

```
                   370       380       390       400       410       420       430       440
str.Toulouse    LTCDAACMVQDTKQLAIQVYRLLTNEALRQEMVDKAYEVATDMAGALERTLRALDPFLQPLVIQTVLSQHRGRYAY
str.JK12        LTCDAACMVQDTKQLAIQVYRLLTNEALRQEMVDKAYEVATDMAGALERTLRALDPFLQPLVIQTVLSQHRGRYAY
str.CCUG45777   LTCDAACMVQDTKQLAIQVYRLLTNEALRQEMVDKAYEVATDMAGALERTLRALDPFLQPLVIQTVLSQHRGRYAY
str.JK56        LTCDAACMVQDTKQLAIQVYRLLTNEALRQEMVDKAYEVATDMAGALERTLRALDPFLQPLVIQTVLSQHRGRYAY
str.JK67        LTCDAACMVQDTKQLAIQVYRLLTNEALRQEMVDKAYEVATDMAGALERTLRALDPFLQPLVIQTVLSQHRGRYAY
str.JK63        LTCDAACMVQDTKQLAIQVYRLLTNEALRQEMVDKAYEVATDMAGALERTLRALDPFLQPLVIQTVLSQHRGRYAY
str.JK19        LTCDAACMVQDTKQLAIQVYRLLTNEALRQEMVDKAYEVATDMAGALERTLRALDPFLQPLVIQTVLSQHRGRYAY
str.JK39        LTCDAACMVQDTKQLAIQVYRLLTNEALRQEMVDKAYEVATDMAGALERTLRALDPFLQPLVIQTVLSQHRGRYAY
str.JK68        LTCDAACMVQDTKQLAIQVYRLLTNEALRQEMVDKAYEVATDMAGALERTLRALDPFLQPLVIQTVLSQHRGRYAY
str.JK31        LTCDAACMVQDTKQLAIQVYRLLTNEALRQEMVDKAYEVATDMAGALERTLRALDPFLQPLVIQTVLSQHRGRYAY
str.G1713       LTCDAACMVQDTKQLAIQVYRLLTNEALRQEMVDKAYEVATDMAGALERTLRALDPFLQPLVIQTVLSQHRGRYAY
str.G1712       LTCDAACMVQDTKQLAIQVYRLLTNEALRQEMVDKAYEVATDMAGALERTLRALDPFLQPLVIQTVLSQHRGRYAY
str.JK73        LTCDAACMVQDTKQLAIQVYRLLTNEALRQEMVDKAYEVATDMAGALERTLRALDPFLQPLVIQTVLSQHRGRYAY
str.JK7         LTCDAACMVQDTKQLAIQVYRLLTNEALRQEMVDKAYEVATDMAGALERTLRALDPFLQPLVIQTVLSQHRGRYAY
str.JK73rel     LTCDAACMVQDTKQLAIQVYRLLTNEALRQEMVDKAYEVATDMAGALERTLRALDPFLQPLVIQTVLSQHRGRYAY
str.BQ2-D70     LTCDAACMVQDTKQLAIQVYRLLTNEALRQEMVDKAYEVATDMAGALERTLRALDPFLQPLVIQTVLSQHRGRYAY
str.CO20_0321   LTCDAACMVQDTKQLAIQVYRLLTNEALRQEMVDKAYEVATDMAGALERTLRALDPFLQPLVIQTVLSQHRGRYAY
str.CO21_0024   LTCDAACMVQDTKQLAIQVYRLLTNEALRQEMVDKAYEVATDMAGALERTLRALDPFLQPLVIQTVLSQHRGRYAY
str.CO20_0297   LTCDAACMVQDTKQLAIQVYRLLTNEALRQEMVDKAYEVATDMAGALERTLRALDPFLQPLVIQTVLSQHRGRYAY
str.CO20_0256   LTCDAACMVQDTKQLAIQVYRLLTNEALRQEMVDKAYEVATDMAGALERTLRALDPFLQPLVIQTVLSQHRGRYAY
str.CO20_0257   LTCDAACMVQDTKQLAIQVYRLLTNEALRQEMVDKAYEVATDMAGALERTLRALDPFLQPLVIQTVLSQHRGRYAY
str.NCTC12899   LTCDAACMVQDTKQLAIQVYRLLTNEALRQEMVDKAYEVATDMAGALERTLRALDPFLQPLVIQTVLSQHRGRYAY
str.RM-11       LTCDAACMVQDTKQLAIQVYRLLTNEALRQEMVDKAYEVATDMAGALERTLKALDPFLQPLVIQTVLSQHRGRYAY
str.MF1-1       LTCDAACMVQDTKQLAIQVYRLLTNEALRQEMVDKAYEVATDMAGALERTLKALDPFLQPLVIQTVLSQHRGRYAY
```

>WP_011179067.1 tetraacyldisaccharide 4'-kinase [Bartonella quintana str. Toulouse]

```
                 1        10        20        30        40        50        60        70        80        90
str.Toulouse     MLISTPHFWWKDKSFLRFLLTPVSWGYGYFSHRCMVRELPVIDLPVLCIGNFTCGGAGKTPVVIAFAKVAKELGFLPGVVSRGYGGAVKGV
str.JK12         MLISTPHFWWKDKSFLRFLLTPVSWGYGYFSHRCMVRELPVIDLPVLCIGNFTCGGAGKTPVVIAFAKVAKELGFLPGVVSRGYGGAVKGV
str.CCUG45777    MLISTPHFWWKDKSFLRFLLTPVSWGYGYFSHRCMVRELPVIDLPVLCIGNFTCGGAGKTPVVIAFAKVAKELGFLPGVVSRGYGGAVKGV
str.JK56         MLISTPHFWWKDKSFLRFLLTPVSWGYGYFSHRCMVRELPVIDLPVLCIGNFTCGGAGKTPVVIAFAKVAKELGFLPGVVSRGYGGAVKGV
str.JK67         MLISTPHFWWKDKSFLRFLLTPVSWGYGYFSHRCMVRELPVIDLPVLCIGNFTCGGAGKTPVVIAFAKVAKELGFLPGVVSRGYGGAVKGV
str.JK19         MLISTPHFWWKDKSFLRFLLTPVSWGYGYFSHRCMVRELPVIDLPVLCIGNFTCGGAGKTPVVIAFAKVAKELGFLPGVVSRGYGGAVKGV
str.JK63         MLISTPHFWWKDKSFLRFLLTPVSWGYGYFSHRCMVRELPVIDLPVLCIGNFTCGGAGKTPVVIAFAKVAKELGFLPGVVSRGYGGAVKGV
str.JK39         MLISTPHFWWKDKSFLRFLLTPVSWGYGYFSHRCMVRELPVIDLPVLCIGNFTCGGAGKTPVVIAFAKVAKELGFLPGVVSRGYGGAVKGV
str.JK68         MLISTPHFWWKDKSFLRFLLTPVSWGYGYFSHRCMVRELPVIDLPVLCIGNFTCGGAGKTPVVIAFAKVAKELGFLPGVVSRGYGGAVKGV
str.JK31         MLISTPHFWWKDKSFLRFLLTPVSWGYGYFSHRCMVRELPVIDLPVLCIGNFTCGGAGKTPVVIAFAKVAKELGFLPGVVSRGYGGAVKGV
str.G1713        MLISTPHFWWKDKSFLRFLLTPVSWGYGYFSHRCMVRELPVIDLPVLCIGNFTCGGAGKTPVVIAFAKVAKELGFLPGVVSRGYGGAVKGV
str.G1712        MLISTPHFWWKDKSFLRFLLTPVSWGYGYFSHRCMVRELPVIDLPVLCIGNFTCGGAGKTPVVIAFAKVAKELGFLPGVVSRGYGGAVKGV
str.JK73         MLISTPHFWWKDKSFLRFLLTPVSWGYGYFSHRCMVRELPVIDLPVLCIGNFTCGGAGKTPVVIAFAKVAKELGFLPGVVSRGYGGAVKGV
str.JK7          MLISTPHFWWKDKSFLRFLLTPVSWGYGYFSHRCMVRELPVIDLPVLCIGNFTCGGAGKTPVVIAFAKVAKELGFLPGVVSRGYGGAVKGV
str.JK73rel      MLISTPHFWWKDKSFLRFLLTPVSWGYGYFSHRCMVRELPVIDLPVLCIGNFTCGGAGKTPVVIAFAKVAKELGFLPGVVSRGYGGAVKGV
str.BQ2-D70      MLISTPHFWWKDKSFLRFLLTPVSWGYGYFSHRCMVRELPVIDLPVLCIGNFTCGGAGKTPVVIAFAKVAKELGFLPGVVSRGYGGAVKGV
str.CO20_0321    MLISTPHFWWKDKSFLRFLLTPVSWGYGYFSHRCMVRELPVIDLPVLCIGNFTCGGAGKTPVVIAFAKVAKELGFLPGVVSRGYGGAVKGV
str.CO21_0024    MLISTPHFWWKDKSFLRFLLTPVSWGYGYFSHRCMVRELPVIDLPVLCIGNFTCGGAGKTPVVIAFAKVAKELGFLPGVVSRGYGGAVKGV
str.CO20_0297    MLISTPHFWWKDKSFLRFLLTPVSWGYGYFSHRCMVRELPVIDLPVLCIGNFTCGGAGKTPVVIAFAKVAKELGFLPGVVSRGYGGAVKGV
str.CO20_0256    MLISTPHFWWKDKSFLRFLLTPVSWGYGYFSHRCMVRELPVIDLPVLCIGNFTCGGAGKTPVVIAFAKVAKELGFLPGVVSRGYGGAVKGV
str.CO20_0257    MLISTPHFWWKDKSFLRFLLTPVSWGYGYFSHRCMVRELPVIDLPVLCIGNFTCGGAGKTPVVIAFAKVAKELGFLPGVVSRGYGGAVKGV
str.NCTC12899    MLISTPHFWWKDKSFLRFLLTPVSWGYGYFSHRCMVRELPVIDLPVLCIGNFTCGGAGKTPVVIAFAKVAKELGFLPGVVSRGYGGAVKGV
str.RM-11        MLISTPHFWWKDKSFLRFLLTPVSWVYGYFSHRCMARELPVIDLPVLCIGNFTCGGAGKTPVVIAFAKVAKELGFLPGVVSRGYGGAVKGV
str.MF1-1        MLISTPHFWWKDKSFLRFLLTPVSWVYGYFSHRCMARELPVIDLPVLCIGNFTCGGAGKTPVVIAFAKVAKELGFLPGVVSRGYGGAVKGV
```

```
                        100       110       120       130       140       150       160       170       180
str.Toulouse     HLINEQFDNARDVGDEALLLARHAFVAISSNRYVAAQRLKEEGCNLILMDDGFQSRRLYMDYALLVVDAMRGFGNGAVFPAGPLRAPLKTQ
str.JK12         HLINEQFDNARDVGDEALLLARHAFVAISSNRYVAAQRLKEEGCNLILMDDGFQSRRLYMDYALLVVDAMRGFGNGAVFPAGPLRAPLKTQ
str.CCUG45777    HLINEQFDNARDVGDEALLLARHAFVAISSNRYVAAQRLKEEGCNLILMDDGFQSRRLYMDYALLVVDAMRGFGNGAVFPAGPLRAPLKTQ
str.JK56         HLINEQFDNARDVGDEALLLARHAFVAISSNRYVAAQRLKEEGCNLILMDDGFQSRRLYMDYALLVVDAMRGFGNGAVFPAGPLRAPLKTQ
str.JK67         HLINEQFDNARDVGDEALLLARHAFVAISSNRYVAAQRLKEEGCNLILMDDGFQSRRLYMDYALLVVDAMRGFGNGAVFPAGPLRAPLKTQ
str.JK19         HLINEQFDNARDVGDEALLLARHAFVAISSNRYVAAQRLKEEGCNLILMDDGFQSRRLYMDYALLVVDAMRGFGNGAVFPAGPLRAPLKTQ
str.JK63         HLINEQFDNARDVGDEALLLARHAFVAISSNRYVAAQRLKEEGCNLILMDDGFQSRRLYMDYALLVVDAMRGFGNGAVFPAGPLRAPLKTQ
str.JK39         HLINEQFDNARDVGDEALLLARHAFVAISSNRYVAAQRLKEEGCNLILMDDGFQSRRLYMDYALLVVDAMRGFGNGAVFPAGPLRAPLKTQ
str.JK68         HLINEQFDNARDVGDEALLLARHAFVAISSNRYVAAQRLKEEGCNLILMDDGFQSRRLYMDYALLVVDAMRGFGNGAVFPAGPLRAPLKTQ
str.JK31         HLINEQFDNARDVGDEALLLARHAFVAISSNRYVAAQRLKEEGCNLILMDDGFQSRRLYMDYALLVVDAMRGFGNGAVFPAGPLRAPLKTQ
str.G1713        HLINEQFDNARDVGDEALLLARHAFVAISSNRYVAAQRLKEEGCNLILMDDGFQSRRLYMDYALLVVDAMRGFGNGAVFPAGPLRAPLKTQ
str.G1712        HLINEQFDNARDVGDEALLLARHAFVAISSNRYVAAQRLKEEGCNLILMDDGFQSRRLYMDYALLVVDAMRGFGNGAVFPAGPLRAPLKTQ
str.JK73         HLINEQFDNARDVGDEALLLARHAFVAISSNRYVAAQRLKEEGCNLILMDDGFQSRRLYMDYALLVVDAMRGFGNGAVFPAGPLRAPLKTQ
str.JK7          HLINEQFDNARDVGDEALLLARHAFVAISSNRYVAAQRLKEEGCNLILMDDGFQSRRLYMDYALLVVDAMRGFGNGAVFPAGPLRAPLKTQ
str.JK73rel      HLINEQFDNARDVGDEALLLARHAFVAISSNRYVAAQRLKEEGCNLILMDDGFQSRRLYMDYALLVVDAMRGFGNGAVFPAGPLRAPLKTQ
str.BQ2-D70      HLINEQFDNARDVGDEALLLARHAFVAISSNRYVAAQRLKEEGCNLILMDDGFQSRRLYMDYALLVVDAMRGFGNGAVFPAGPLRAPLKTQ
str.CO20_0321    HLINEQFDNARDVGDEALLLARHAFVAISSNRYVAAQRLKEEGCNLILMDDGFQSRRLYMDYALLVVDAMRGFGNGAVFPAGPLRAPLKTQ
str.CO21_0024    HLINEQFDNARDVGDEALLLARHAFVAISSNRYVAAQRLKEEGCNLILMDDGFQSRRLYMDYALLVVDAMRGFGNGAVFPAGPLRAPLKTQ
str.CO20_0297    HLINEQFDNARDVGDEALLLARHAFVAISSNRYVAAQRLKEEGCNLILMDDGFQSRRLYMDYALLVVDAMRGFGNGAVFPAGPLRAPLKTQ
str.CO20_0256    HLINEQFDNARDVGDEALLLARHAFVAISSNRYVAAQRLKEEGCNLILMDDGFQSRRLYMDYALLVVDAMRGFGNGAVFPAGPLRAPLKTQ
str.CO20_0257    HLINEQFDNARDVGDEALLLARHAFVAISSNRYVAAQRLKEEGCNLILMDDGFQSRRLYMDYALLVVDAMRGFGNGAVFPAGPLRAPLKTQ
str.NCTC12899    HLINEQFDNARDVGDEALLLARHAFVAISSNRYVAAQRLKEEGCNLILMDDGFQSRRLYMDYALLVVDAMRGFGNGAVFPAGPLRAPLKTQ
str.RM-11        HLINEQFDNARDVGDEALLLARHAFVAISSNRYAAAQRLKEEGCNLILMDDGFQSRRLYMDYALLVVDAMRGFGNGAVFPAGPLRAPLKTQ
str.MF1-1        HLINEQFDNARDVGDEALLLARHAFVAISSNRYAAAQRLKEEGCNLILMDDGFQSRRLYMDYALLVVDAMRGFGNGAVFPAGPLRAPLKTQ
```

```
                      190       200       210       220       230       240       250       260       270
str.Toulouse     FSLMDSVLLIGHSDACENVVFLVTRTGKALHHAHLKSLASDEVMGKSFLAFAGIGNPNKFFKSIKELSGRVVQTYSYPDHYFFTDTDLKNL
str.JK12         FSLMDSVLLIGHSDACENVVFLVTRTGKALHHAHLKSLASDEVMGKSFLAFAGIGNPNKFFKSIKELSGRVVQTYSYPDHYFFTDTDLKNL
str.CCUG45777    FSLMDSVLLIGHSDACENVVFLVTRTGKALHHAHLKSLASDEVMGKSFLAFAGIGNPNKFFKSIKELSGRVVQTYSYPDHYFFTDTDLKNL
str.JK56         FSLMDSVLLIGHSDACENVVFLVTRTGKALHHAHLKSLASDEVMGKSFLAFAGIGNPNKFFKSIKELSGRVVQTYSYPDHYFFTDTDLKNL
str.JK67         FSLMDSVLLIGHSDACENVVFLVTRTGKALHHAHLKSLASDEVMGKSFLAFAGIGNPNKFFKSIKELSGRVVQTYSYPDHYFFTDTDLKNL
str.JK19         FSLMDSVLLIGHSDACENVVFLVTRTGKALHHAHLKSLASDEVMGKSFLAFAGIGNPNKFFKSIKELSGRVVQTYSYPDHYFFTDTDLKNL
str.JK63         FSLMDSVLLIGHSDACENVVFLVTRTGKALHHAHLKSLASDEVMGKSFLAFAGIGNPNKFFKSIKELSGRVVQTYSYPDHYFFTDTDLKNL
str.JK39         FSLMDSVLLIGHSDACENVVFLVTRTGKALHHAHLKSLASDEVMGKSFLAFAGIGNPNKFFKSIKELSGRVVQTYSYPDHYFFTDTDLKNL
str.JK68         FSLMDSVLLIGHSDACENVVFLVTRTGKALHHAHLKSLASDEVMGKSFLAFAGIGNPNKFFKSIKELSGRVVQTYSYPDHYFFTDTDLKNL
str.JK31         FSLMDSVLLIGHSDACENVVFLVTRTGKALHHAHLKSLASDEVMGKSFLAFAGIGNPNKFFKSIKELSGRVVQTYSYPDHYFFTDTDLKNL
str.G1713        FSLMDSVLLIGHSDACENVVFLVTRTGKALHHAHLKSLASDEVMGKSFLAFAGIGNPNKFFKSIKELSGRVVQTYSYPDHYFFTDTDLKNL
str.G1712        FSLMDSVLLIGHSDACENVVFLVTRTGKALHHAHLKSLASDEVMGKSFLAFAGIGNPNKFFKSIKELSGRVVQTYSYPDHYFFTDTDLKNL
str.JK73         FSLMDSVLLIGHSDACENVVFLVTRTGKALHHAHLKSLASDEVMGKSFLAFAGIGNPNKFFKSIKELSGRVVQTYSYPDHYFFTDTDLKNL
str.JK7          FSLMDSVLLIGHSDACENVVFLVTRTGKALHHAHLKSLASDEVMGKSFLAFAGIGNPNKFFKSIKELSGRVVQTYSYPDHYFFTDTDLKNL
str.JK73rel      FSLMDSVLLIGHSDACENVVFLVTRTGKALHHAHLKSLASDEVMGKSFLAFAGIGNPNKFFKSIKELSGRVVQTYSYPDHYFFTDTDLKNL
str.BQ2-D70      FSLMDSVLLIGHSDACENVVFLVTRTGKALHHAHLKSLASDEVMGKSFLAFAGIGNPNKFFKSIKELSGRVVQTYSYPDHYFFTDTDLKNL
str.CO20_0321    FSLMDSVLLIGHSDACENVVFLVTRTGKALHHAHLKSLASDEVMGKSFLAFAGIGNPNKFFKSIKELSGRVVQTYSYPDHYFFTDTDLKNL
str.CO21_0024    FSLMDSVLLIGHSDACENVVFLVTRTGKALHHAHLKSLASDEVMGKSFLAFAGIGNPNKFFKSIKELSGRVVQTYSYPDHYFFTDTDLKNL
str.CO20_0297    FSLMDSVLLIGHSDACENVVFLVTRTGKALHHAHLKSLASDEVMGKSFLAFAGIGNPNKFFKSIKELSGRVVQTYSYPDHYFFTDTDLKNL
str.CO20_0256    FSLMDSVLLIGHSDACENVVFLVTRTGKALHHAHLKSLASDEVMGKSFLAFAGIGNPNKFFKSIKELSGRVVQTYSYPDHYFFTDTDLKNL
str.CO20_0257    FSLMDSVLLIGHSDACENVVFLVTRTGKALHHAHLKSLASDEVMGKSFLAFAGIGNPNKFFKSIKELSGRVVQTYSYPDHYFFTDTDLKNL
str.NCTC12899    FSLMDSVLLIGHSDACENVVFLVTRTGKALHHAHLKSLASDEVMGKSFLAFAGIGNPNKFFKSIKELSGRVVQTYSYPDHYFFTDTDLKNL
str.RM-11        FSLMDSVLLIGHSDACENVVFLVTRTGKALHHAHLKSLASDEVMGKSFLAFAGIGNPNKFFKSIKELSGHVVQTYSYPDHYFFTDTDLKNL
str.MF1-1        FSLMDSVLLIGHSNACENVVFLVTRTGKALHHAHLKSLASDEVMGKSFLAFAGIGNPNKFFKSIKELSGHVVQTYSYPDHYFFTDTDLKNL
```

```
                     280       290       300       310       320       330       340
str.Toulouse     IQQAKMNNLWLATTAKDYTRIQTSHVQKDLKNLIVFDVEVDFVQEDFCRMILEEVMSRFERKTLFNLHF
str.JK12         IQQAKMNNLWLATTAKDYTRIQTSHVQKDLKNLIVFDVEVDFVQEDFCRMILEEVMSRFERKTLFNLHF
str.CCUG45777    IQQAKMNNLWLATTAKDYTRIQTSHVQKDLKNLIVFDVEVDFVQEDFCRMILEEVMSRFERKTLFNLHF
str.JK56         IQQAKMNNLWLATTAKDYTRIQTSHVQKDLKNLIVFDVEVDFVQEDFCRMILEEVMSRFERKTLFNLHF
str.JK67         IQQAKMNNLWLATTAKDYTRIQTSHVQKDLKNLIVFDVEVDFVQEDFCRMILEEVMSRFERKTLFNLHF
str.JK19         IQQAKMNNLWLATTAKDYTRIQTSHVQKDLKNLIVFDVEVDFVQEDFCRMILEEVMSRFERKTLFNLHF
str.JK63         IQQAKMNNLWLATTAKDYTRIQTSHVQKDLKNLIVFDVEVDFVQEDFCRMILEEVMSRFERKTLFNLHF
str.JK39         IQQAKMNNLWLATTAKDYTRIQTSHVQKDLKNLIVFDVEVDFVQEDFCRMILEEVMSRFERKTLFNLHF
str.JK68         IQQAKMNNLWLATTAKDYTRIQTSHVQKDLKNLIVFDVEVDFVQEDFCRMILEEVMSRFERKTLFNLHF
str.JK31         IQQAKMNNLWLATTAKDYTRIQTSHVQKDLKNLIVFDVEVDFVQEDFCRMILEEVMSRFERKTLFNLHF
str.G1713        IQQAKMNNLWLATTAKDYTRIQTSHVQKDLKNLIVFDVEVDFVQEDFCRMILEEVMSRFERKTLFNLHF
str.G1712        IQQAKMNNLWLATTAKDYTRIQTSHVQKDLKNLIVFDVEVDFVQEDFCRMILEEVMSRFERKTLFNLHF
str.JK73         IQQAKMNNLWLATTAKDYTRIQTSHVQKDLKNLIVFDVEVDFVQEDFCRMILEEVMSRFERKTLFNLHF
str.JK7          IQQAKMNNLWLATTAKDYTRIQTSHVQKDLKNLIVFDVEVDFVQEDFCRMILEEVMSRFERKTLFNLHF
str.JK73rel      IQQAKMNNLWLATTAKDYTRIQTSHVQKDLKNLIVFDVEVDFVQEDFCRMILEEVMSRFERKTLFNLHF
str.BQ2-D70      IQQAKMNNLWLATTAKDYTRIQTSHVQKDLKNLIVFDVEVDFVQEDFCRMILEEVMSRFERKTLFNLHF
str.CO20_0321    IQQAKMNNLWLATTAKDYTRIQTSHVQKDLKNLIVFDVEVDFVQEDFCRMILEEVMSRFERKTLFNLHF
str.CO21_0024    IQQAKMNNLWLATTAKDYTRIQTSHVQKDLKNLIVFDVEVDFVQEDFCRMILEEVMSRFERKTLFNLHF
str.CO20_0297    IQQAKMNNLWLATTAKDYTRIQTSHVQKDLKNLIVFDVEVDFVQEDFCRMILEEVMSRFERKTLFNLHF
str.CO20_0256    IQQAKMNNLWLATTAKDYTRIQTSHVQKDLKNLIVFDVEVDFVQEDFCRMILEEVMSRFERKTLFNLHF
str.CO20_0257    IQQAKMNNLWLATTAKDYTRIQTSHVQKDLKNLIVFDVEVDFVQEDFCRMILEEVMSRFERKTLFNLHF
str.NCTC12899    IQQAKMNNLWLATTAKDYTRIQTSHVQKDLKNLIVFDVEVDFVQEDFCRMILEEVMSRFERKTLFNLHF
str.RM-11        IQQAKMNNLWLATTAKDYTRIQTSHMQKDLKNLIVFDVEVDFVQEDFCRMILEEVMSRFERKTLFNLHF
str.MF1-1        IQQAKMNNLWLATTAKDYTRIQTSHMQKDLKNLIVFDVEVDFVQEDFCRMILEEVMSRFERKTLFNLHF
```

>WP_011179434.1 lipid-A-disaccharide synthase [Bartonella quintana str. Toulouse]

```
               1        10        20        30        40        50        60        70        80        90        100
str.Toulouse   MNNCFLKIAVVAGEESGDSLGADLISCLSQQTGCNIHLIGVGGRHLKTLGLSIFNFHDIALIGLGAVLKKLPLLLIHIHNLSKLIAQEQPDCLIIIDSPDFTHRV
str.CO20_0321  MNNCFLKIAVVAGEESGDSLGADLISCLSQQTGCNIHLIGVGGRHLKTLGLSIFNFHDIALIGLGAVLKKLPLLLIHIHNLSKLIAQEQPDCLIIIDSPDFTHRV
str.JK39       MNNCFLKIAVVAGEESGDSLGADLISCLSQQTGCNIHLIGVGGRHLKTLGLSIFNFHDIALIGLGAVLKKLPLLLIHIHNLSKLIAQEQPDCLIIIDSPDFTHRV
str.JK19       MNNCFLKIAVVAGEESGDSLGADLISCLSQQTGCNIHLIGVGGRHLKTLGLSIFNFHDIALIGLGAVLKKLPLLLIHIHNLSKLIAQEQPDCLIIIDSPDFTHRV
str.JK56       MNNCFLKIAVVAGEESGDSLGADLISCLSQQTGCNIHLIGVGGRHLKTLGLSIFNFHDIALIGLGAVLKKLPLLLIHIHNLSKFIAQEQPDCLIIIDSPDFTHRV
str.JK12       MNNCFLKIAVVAGEESGDSLGADLISCLSQQTGCNIHLIGVGGRHLKTLGLSIFNFHDIALIGLGAVLKKLPLLLIHIHNLSKFIAQEQPDCLIIIDSPDFTHRV
str.JK63       MNNCFLKIAVVAGEESGDSLGADLISCLSQQTGCNIHLIGVGGRHLKTLGLSIFNFHDIALIGLGAVLKKLPLLLIHIHNLSKFIAQEQPDCLIIIDSPDFTHRV
str.JK67       MNNCFLKIAVVAGEESGDSLGADLISCLSQQTGCNIHLIGVGGRHLKTLGLSIFNFHDIALIGLGAVLKKLPLLLIHIHNLSKFIAQEQPDCLIIIDSPDFTHRV
str.JK31       MNNCFLKIAVVAGEESGDSLGADLISCLSQQTGCNIHLIGVGGRHLKTLGLSIFNFHDIALIGLGAVLKKLPLLLIHIHNLSKFIAQEQPDCLIIIDSPDFTHRV
str.JK68       MNNCFLKIAVVAGEESGDSLGADLISCLSQQTGCNIHLIGVGGRHLKTLGLSIFNFHDIALIGLGAVLKKLPLLLIHIHNLSKFIAQEQPDCLIIIDSPDFTHRV
str.JK7        MNNCFLKIAVVAGEESGDSLGADLISCLSQQTGCNIHLIGVGGRHLKTLGLSIFNFHDIALIGLGAVLKKLPLLLIHIHNLSKFIAQEQPDCLIIIDSPDFTHRV
str.JK73       MNNCFLKIAVVAGEESGDSLGADLISCLSQQTGCNIHLIGVGGRHLKTLGLSIFNFHDIALIGLGAVLKKLPLLLIHIHNLSKFIAQEQPDCLIIIDSPDFTHRV
str.BQ2-D70    MNNCFLKIAVVAGEESGDSLGADLISCLSQQTGCNIHLIGVGGRHLKTLGLSIFNFHDIALIGLGAVLKKLPLLLIHIHNLSKFIAQEQPDCLIIIDSPDFTHRV
str.JK73rel    MNNCFLKIAVVAGEESGDSLGADLISCLSQQTGCNIHLIGVGGRHLKTLGLSIFNFHDIALIGLGAVLKKLPLLLIHIHNLSKFIAQEQPDCLIIIDSPDFTHRV
str.CCUG45777  MNNCFLKIAVVAGEESGDSLGADLISCLSQQTGCNIHLIGVGGRHLKTLGLSIFNFHDIALIGLGAVLKKLPLLLIHIHNLSKFIAQEQPDCLIIIDSPDFTHRV
str.G1713      MNNCFLKIAVVAGEESGDSLGADLISCLSQQTGCNIHLIGVGGRHLKTLGLSIFNFHDIALIGLGAVLKKLPLLLIHIHNLSKFIAQEQPDCLIIIDSPDFTHRV
str.G1712      MNNCFLKIAVVAGEESGDSLGADLISCLSQQTGCNIHLIGVGGRHLKTLGLSIFNFHDIALIGLGAVLKKLPLLLIHIHNLSKFIAQEQPDCLIIIDSPDFTHRV
str.CO20_0256  MNNCFLKIAVVAGEESGDSLGADLISCLSQQTGCNIHLIGVGGRHLKTLGLSIFNFHDIALIGLGAVLKKLPLLLIHIHNLSKFIAQEQPDCLIIIDSPDFTHRV
str.CO20_0297  MNNCFLKIAVVAGEESGDSLGADLISCLSQQTGCNIHLIGVGGRHLKTLGLSIFNFHDIALIGLGAVLKKLPLLLIHIHNLSKFIAQEQPDCLIIIDSPDFTHRV
str.CO21_0024  MNNCFLKIAVVAGEESGDSLGADLISCLSQQTGCNIHLIGVGGRHLKTLGLSIFNFHDIALIGLGAVLKKLPLLLIHIHNLSKFIAQEQPDCLIIIDSPDFTHRV
str.CO20_0257  MNNCFLKIAVVAGEESGDSLGADLISCLSQQTGCNIHLIGVGGRHLKTLGLSIFNFHDIALIGLGAVLKKLPLLLIHIHNLSKFIAQEQPDCLIIIDSPDFTHRV
str.NCTC12899  MNNCFLKIAVVAGEESGDSLGADLISCLSQQTGCNIHLIGVGGRHLKTLGLSIFNFHDIALIGLGAVLKKLPLLLIHIHNLSKFIAQEQPDCLIIIDSPDFTHRV
str.MF1-1      MNNCFLKIAVVAGEESGDSLGADLISCLSQQTGCNIHLIGVGGRHLKTLGLSIFNFHDIALIGLGAVLKKLPLLLIHIHNLSKFIAQEQPDCLIIIDSPDFTHRV
str.RM-11      MNNCFLKIAVVAGEESGDSLGADLISCLSQQTGRNIHLIGVGGRHLKTLGLSIFNFHDIALIGLGAVLKKLPLLLIHIHNLSKFIAQEQPDCLIIIDSPDFTHRV

                   110       120       130       140       150       160       170       180       190       200       210
str.Toulouse   AKKVRSLAPSIPIIKYVAPTVWAWRPERARAMRKFVDHVLAVFPFEEKIMTDLEGPPTTYVGHRLLTYPPLLTVQSEKKHSFGKQASFLTLIVLPGSRNLEIRYL
str.CO20_0321  AKKVRSLAPSIPIIKYVAPTVWAWRPERARAMRKFVDHVLAVFPFEEKIMTDLEGPPTTYVGHRLLTYPPLLTVQSEKKHSFGKQASFLTLIVLPGSRNLEIRYL
str.JK39       AKKVRSLAPSIPIIKYVAPTVWAWRPERARAMRKFVDHVLAVFPFEEKIMTDLEGPPTTYVGHRLLTYPPLLTVQSEKKHSFGKQASFLTLIVLPGSRNLEIRYL
str.JK19       AKKVRSLAPSIPIIKYVAPTVWAWRPERARAMRKFVDHVLAVFPFEEKIMTDLEGPPTTYVGHRLLTYPPLLTVQSEKKHSFGKQASFLTLIVLPGSRNLEIRYL
str.JK56       AKKVRSLAPSIPIIKYVAPTVWAWRPERARAMRKFVDHVLAVFPFEEKIMTDLEGPPTTYVGHRLLTYPPLLTVQSEKKHSFGKQASFLTLIVLPGSRNLEIRYL
str.JK12       AKKVRSLAPSIPIIKYVAPTVWAWRPERARAMRKFVDHVLAVFPFEEKIMTDLEGPPTTYVGHRLLTYPPLLTVQSEKKHSFGKQASFLTLIVLPGSRNLEIRYL
str.JK63       AKKVRSLAPSIPIIKYVAPTVWAWRPERARAMRKFVDHVLAVFPFEEKIMTDLEGPPTTYVGHRLLTYPPLLTVQSEKKHSFGKQASFLTLIVLPGSRNLEIRYL
str.JK67       AKKVRSLAPSIPIIKYVAPTVWAWRPERARAMRKFVDHVLAVFPFEEKIMTDLEGPPTTYVGHRLLTYPPLLTVQSEKKHSFGKQASFLTLIVLPGSRNLEIRYL
str.JK31       AKKVRSLAPSIPIIKYVAPTVWAWRPERARAMRKFVDHVLAVFPFEEKIMTDLEGPPTTYVGHRLLTYPPLLTVQSEKKHSFGKQASFLTLIVLPGSRNLEIRYL
str.JK68       AKKVRSLAPSIPIIKYVAPTVWAWRPERARAMRKFVDHVLAVFPFEEKIMTDLEGPPTTYVGHRLLTYPPLLTVQSEKKHSFGKQASFLTLIVLPGSRNLEIRYL
str.JK7        AKKVRSLAPSIPIIKYVAPTVWAWRPERARAMRKFVDHVLAVFPFEEKIMTDLEGPPTTYVGHRLLTYPPLLTVQSEKKHSFGKQASFLTLIVLPGSRNLEIRYL
str.JK73       AKKVRSLAPSIPIIKYVAPTVWAWRPERARAMRKFVDHVLAVFPFEEKIMTDLEGPPTTYVGHRLLTYPPLLTVQSEKKHSFGKQASFLTLIVLPGSRNLEIRYL
str.BQ2-D70    AKKVRSLAPSIPIIKYVAPTVWAWRPERARAMRKFVDHVLAVFPFEEKIMTDLEGPPTTYVGHRLLTYPPLLTVQSEKKHSFGKQASFLTLIVLPGSRNLEIRYL
str.JK73rel    AKKVRSLAPSIPIIKYVAPTVWAWRPERARAMRKFVDHVLAVFPFEEKIMTDLEGPPTTYVGHRLLTYPPLLTVQSEKKHSFGKQASFLTLIVLPGSRNLEIRYL
str.CCUG45777  AKKVRSLAPSIPIIKYVAPTVWAWRPERARAMRKFVDHVLAVFPFEEKIMTDLEGPPTTYVGHRLLTYPPLLTVQSEKKHSFGKQASFLTLIVLPGSRNLEIRYL
str.G1713      AKKVRSLAPSIPIIKYVAPTVWAWRPERARAMRKFVDHVLAVFPFEEKIMTDLEGPPTTYVGHRLLTYPPLLTVQSEKKHSFGKQASFLTLIVLPGSRNLEIRYL
str.G1712      AKKVRSLAPSIPIIKYVAPTVWAWRPERARAMRKFVDHVLAVFPFEEKIMTDLEGPPTTYVGHRLLTYPPLLTVQSEKKHSFGKQASFLTLIVLPGSRNLEIRYL
str.CO20_0256  AKKVRSLAPSIPIIKYVAPTVWAWRPERARAMRKFVDHVLAVFPFEEKIMTDLEGPPTTYVGHRLLTYPPLLTVQSEKKHSFGKQASFLTLIVLPGSRNLEIRYL
str.CO20_0297  AKKVRSLAPSIPIIKYVAPTVWAWRPERARAMRKFVDHVLAVFPFEEKIMTDLEGPPTTYVGHRLLTYPPLLTVQSEKKHSFGKQASFLTLIVLPGSRNLEIRYL
str.CO21_0024  AKKVRSLAPSIPIIKYVAPTVWAWRPERARAMRKFVDHVLAVFPFEEKIMTDLEGPPTTYVGHRLLTYPPLLTVQSEKKHSFGKQASFLTLIVLPGSRNLEIRYL
str.CO20_0257  AKKVRSLAPSIPIIKYVAPTVWAWRPERARAMRKFVDHVLAVFPFEEKIMTDLEGPPTTYVGHRLLTYPPLLTVQSEKKHSFGKQASFLTLIVLPGSRNLEIRYL
str.NCTC12899  AKKVRSLAPSIPIIKYVAPTVWAWRPERARAMRKFVDHVLAVFPFEEKIMTDLEGPPTTYVGHRLLTYPPLLTVQSEKKHSFGKQASFLTLIVLPGSRNLEIRYL
str.MF1-1      AKKVRSLAPSIPIIKYVAPTVWAWRPERARTMRKFVDHVLAVFPFEKKIMTDLEGPPTTYVGHRLLTYPPLLTVQSEKKHSFGKQESFLTLIVLPGSRNLEIRYL
str.RM-11      AKKVRSLAPSIPIIKYVAPTVWAWRPERARTMRKFVDHVLAVFPFEEKIMTDLEGPPTTYVGHRLLTYPPLLTVQSEKKHSFGKQESFLTLIVLPGSRNLEIRYL

                        220       230       240       250       260       270       280       290       300       310
str.Toulouse   MPIFGEAVEILAQRIPNLRIIILPTLPHLVDEIRCFVQKWSKVEIVVGEEAKWRAFADANVALAALGTVSLELALARIPMVLCYKLDRFSKFFIFPKIMLWSAALP
str.CO20_0321  MPIFGEAVEILAQRIPNLRIIILPTLPHLVDEIRCFVQKWSKVEIVVGEEAKWRAFADANVALAALGTVSLELALARIPMVLCYKLDRFSKFFIFPKIMLWSAALP
str.JK39       MPIFGEAVEILAQRIPNLRIIILPTLPHLVDEIRCFVQKWSKVEIVVGEEAKWRAFADANVALAALGTVSLELALARIPMVLCYKLDRFSKFFIFPKIMLWSAALP
str.JK19       MPIFGEAVEILAQRIPNLRIIILPTLPHLVDEIRCFVQKWSKVEIVVGEEAKWRAFADANVALAALGTVSLELALARIPMVLCYKLDRFSKFFIFPKIMLWSAALP
str.JK56       MPIFGEAVEILAQRIPNLRIIILPTLPHLVDEIRCFVQKWSKVEIVVGEEAKWRAFADANVALAALGTVSLELALARIPMVLCYKLDRFSKFFIFPKIMLWSAALP
str.JK12       MPIFGEAVEILAQRIPNLRIIILPTLPHLVDEIRCFVQKWSKVEIVVGEEAKWRAFADANVALAALGTVSLELALARIPMVLCYKLDRFSKFFIFPKIMLWSAALP
str.JK63       MPIFGEAVEILAQRIPNLRIIILPTLPHLVDEIRCFVQKWSKVEIVVGEEAKWRAFADANVALAALGTVSLELALARIPMVLCYKLDRFSKFFIFPKIMLWSAALP
str.JK67       MPIFGEAVEILAQRIPNLRIIILPTLPHLVDEIRCFVQKWSKVEIVVGEEAKWRAFADANVALAALGTVSLELALARIPMVLCYKLDRFSKFFIFPKIMLWSAALP
str.JK31       MPIFGEAVEILAQRIPNLRIIILPTLPHLVDEIRCFVQKWSKVEIVVGEEAKWRAFADANVALAALGTVSLELALARIPMVLCYKLDRFSKFFIFPKIMLWSAALP
str.JK68       MPIFGEAVEILAQRIPNLRIIILPTLPHLVDEIRCFVQKWSKVEIVVGEEAKWRAFADANVALAALGTVSLELALARIPMVLCYKLDRFSKFFIFPKIMLWSAALP
str.JK7        MPIFGEAVEILAQRIPNLRIIILPTLPHLVDEIRCFVQKWSKVEIVVGEEAKWRAFADANVALAALGTVSLELALARIPMVLCYKLDRFSKFFIFPKIMLWSAALP
str.JK73       MPIFGEAVEILAQRIPNLRIIILPTLPHLVDEIRCFVQKWSKVEIVVGEEAKWRAFADANVALAALGTVSLELALARIPMVLCYKLDRFSKFFIFPKIMLWSAALP
str.BQ2-D70    MPIFGEAVEILAQRIPNLRIIILPTLPHLVDEIRCFVQKWSKVEIVVGEEAKWRAFADANVALAALGTVSLELALARIPMVLCYKLDRFSKFFIFPKIMLWSAALP
str.JK73rel    MPIFGEAVEILAQRIPNLRIIILPTLPHLVDEIRCFVQKWSKVEIVVGEEAKWRAFADANVALAALGTVSLELALARIPMVLCYKLDRFSKFFIFPKIMLWSAALP
str.CCUG45777  MPIFGEAVEILAQRIPNLRIIILPTLPHLVDEIRCFVQKWSKVEIVVGEEAKWRAFADANVALAALGTVSLELALARIPMVLCYKLDRFSKFFIFPKIMLWSAALP
str.G1713      MPIFGEAVEILAQRIPNLRIIILPTLPHLVDEIRCFVQKWSKVEIVVGEEAKWRAFADANVALAALGTVSLELALARIPMVLCYKLDRFSKFFIFPKIMLWSAALP
str.G1712      MPIFGEAVEILAQRIPNLRIIILPTLPHLVDEIRCFVQKWSKVEIVVGEEAKWRAFADANVALAALGTVSLELALARIPMVLCYKLDRFSKFFIFPKIMLWSAALP
str.CO20_0256  MPIFGEAVEILAQRIPNLRIIILPTLPHLVDEIRCFVQKWSKVEIVVGEEAKWRAFADANVALAALGTVSLELALARIPMVLCYKLDRFSKFFIFPKIMLWSAALP
str.CO20_0297  MPIFGEAVEILAQRIPNLRIIILPTLPHLVDEIRCFVQKWSKVEIVVGEEAKWRAFADANVALAALGTVSLELALARIPMVLCYKLDRFSKFFIFPKIMLWSAALP
str.CO21_0024  MPIFGEAVEILAQRIPNLRIIILPTLPHLVDEIRCFVQKWSKVEIVVGEEAKWRAFADANVALAALGTVSLELALARIPMVLCYKLDRFSKFFIFPKIMLWSAALP
str.CO20_0257  MPIFGEAVEILAQRIPNLRIIILPTLPHLVDEIRCFVQKWSKVEIVVGEEAKWRAFADANVALAALGTVSLELALARIPMVLCYKLDRFSKFFIFPKIMLWSAALP
str.NCTC12899  MPIFGEAVEILAQRIPNLRIIILPTLPHLVDEIRCFVQKWSKVEIVVGEEAKWRAFADANVALAALGTVSLELALARIPMVLCYKLDRFSKFFIFPKIMLWSAALP
str.MF1-1      MPIFGEAVEILAQRIPNLRIIILPTLPHLVDEIRCFVQKWSKVEIVVGEEAKWRAFADANVALAALGTVSLELALARIPMVLCYKLDRFSKFFIFPKIMLWSAALP
str.RM-11      MPIFGEAVEILAQRIPNLRIIILPTLPHLVDEIRCFVQKWSKVEIVVGEEAKWRAFADANVALAALGTVSLELALARIPMVLCYKLDRFSKFFIFPKIMLWSAALP

                    320       330       340       350       360       370       380       390
str.Toulouse   NILSDKPIVPEYFNEFLRPGMLARQIEQLLHNPLLRQAQLDAFELMEQKMKTEVPPGIIAAQTIITLLKEKLGHLKFS
str.CO20_0321  NILSDKPIVPEYFNEFLRPGMLARQIEQLLHNPLLRQAQLDAFELMEQKMKTEVPPGIIAAQTIITLLKEKLGHLKFS
str.JK39       NILSDKPIVPEYFNEFLRPGMLARQIEQLLHNPLLRQAQLDAFELMEQKMKTEVPPGIIAAQTIITLLKEKLGHLKFS
str.JK19       NILSDKPIVPEYFNEFLRPGMLARQIEQLLHNPLLRQAQLDAFELMEQKMKTEVPPGIIAAQTIITLLKEKLGHLKFS
str.JK56       NILSDKPIVPEYFNEFLRPGMLARQIEQLLHNPLLRQAQLDAFELMEQKMKTEVPPGIIAAQTIITLLKEKFGHLKFS
str.JK12       NILSDKPIVPEYFNEFLRPGMLARQIEQLLHNPLLRQAQLDAFELMEQKMKTEVPPGIIAAQTIITLLKEKFGHLKFS
str.JK63       NILSDKPIVPEYFNEFLRPGMLARQIEQLLHNPLLRQAQLDAFELMEQKMKTEVPPGIIAAQTIITLLKEKFGHLKFS
str.JK67       NILSDKPIVPEYFNEFLRPGMLARQIEQLLHNPLLRQAQLDAFELMEQKMKTEVPPGIIAAQTIITLLKEKFGHLKFS
str.JK31       NILSDKPIVPEYFNEFLRPGMLARQIEQLLHNPLLRQAQLDAFELMEQKMKTEVPPGIIAAQTIITLLKEKFGHLKFS
str.JK68       NILSDKPIVPEYFNEFLRPGMLARQIEQLLHNPLLRQAQLDAFELMEQKMKTEVPPGIIAAQTIITLLKEKFGHLKFS
str.JK7        NILSDKPIVPEYFNEFLRPGMLARQIEQLLHNPLLRQAQLDAFELMEQKMKTEVPPGIIAAQTIITLLKEKFGHLKFS
str.JK73       NILSDKPIVPEYFNEFLRPGMLARQIEQLLHNPLLRQAQLDAFELMEQKMKTEVPPGIIAAQTIITLLKEKFGHLKFS
str.BQ2-D70    NILSDKPIVPEYFNEFLRPGMLARQIEQLLHNPLLRQAQLDAFELMEQKMKTEVPPGIIAAQTIITLLKEKFGHLKFS
str.JK73rel    NILSDKPIVPEYFNEFLRPGMLARQIEQLLHNPLLRQAQLDAFELMEQKMKTEVPPGIIAAQTIITLLKEKFGHLKFS
str.CCUG45777  NILSDKPIVPEYFNEFLRPGMLARQIEQLLHNPLLRQAQLDAFELMEQKMKTEVPPGIIAAQTIITLLKEKFGHLKFS
str.G1713      NILSDKPIVPEYFNEFLRPGMLARQIEQLLHNPLLRQAQLDAFELMEQKMKTEVPPGIIAAQTIITLLKEKFGHLKFS
str.G1712      NILSDKPIVPEYFNEFLRPGMLARQIEQLLHNPLLRQAQLDAFELMEQKMKTEVPPGIIAAQTIITLLKEKFGHLKFS
str.CO20_0256  NILSDKPIVPEYFNEFLRPGMLARQIEQLLHNPLLRQAQLDAFELMEQKMKTEVPPGIIAAQTIITLLKEKFGHLKFS
str.CO20_0297  NILSDKPIVPEYFNEFLRPGMLARQIEQLLHNPLLRQAQLDAFELMEQKMKTEVPPGIIAAQTIITLLKEKFGHLKFS
str.CO21_0024  NILSDKPIVPEYFNEFLRPGMLARQIEQLLHNPLLRQAQLDAFELMEQKMKTEVPPGIIAAQTIITLLKEKFGHLKFS
str.CO20_0257  NILSDKPIVPEYFNEFLRPGMLARQIEQLLHNPLLRQAQLDAFELMEQKMKTEVPPGIIAAQTIITLLKEKFGHLKFS
str.NCTC12899  NILSDKPIVPEYFNEFLRPGMLARQIEQLLHNPLLRQAQLDAFELMEQKMKTEVPPGIIAAQTIITLLKEKFGHLKFS
str.MF1-1      NILSDKPIVPEYFNEFLRPGMLARQIEQLLHNPLLRQAQLDAFELMEQKMKTEVPPGIIAAQTIITLLKEKLGHLKFS
str.RM-11      NILSDKPIVPEYFNEFLRPGMLARQIEQLLHNPLLRQAQLDAFELMEQKMKTEVPPGIIAAQTIITLLKEKLGHLKFS
```

>WP_011179438.1 UDP-3-O-(3-hydroxymyristoyl)glucosamine N-acyltransferase [Bartonella quintana str. Toulouse]

```
               1        10        20        30        40        50        60        70        80        90
str.Toulouse   MADTFFFTPSRRLTVANVAELTGAKLLNPEFSNIVISTLSSLEGAGEGSLVFVEHRKFSDALLGSSAVAVFCTNEIVFKVPESMAILVTST
str.MF1-1      MADTFFFTPSRRLTVANVAELTGAKLLNPEFSNIVISTLSSLEGAGEGSLVFVEHRKFSDALLGSSAVAVFCTNEIVFKVPESMAILVTST
str.NCTC12899  MADTFFFTPSRRLTVANVAELTGAKLLNPEFSNIVISTLSSLEGAGEGSLVFVEHRKFSDALLGSSAVAVFCTNEIVFKVPESMAILVTST
str.CO20_0257  MADTFFFTPSRRLTVANVAELTGAKLLNPEFSNIVISTLSSLEGAGEGSLVFVEHRKFSDALLGSSAVAVFCTNEIVFKVPESMAILVTST
str.CO20_0256  MADTFFFTPSRRLTVANVAELTGAKLLNPEFSNIVISTLSSLEGAGEGSLVFVEHRKFSDALLGSSAVAVFCTNEIVFKVPESMAILVTST
str.CO20_0297  MADTFFFTPSRRLTVANVAELTGAKLLNPEFSNIVISTLSSLEGAGEGSLVFVEHRKFSDALLGSSAVAVFCTNEIVFKVPESMAILVTST
str.CO21_0024  MADTFFFTPSRRLTVANVAELTGAKLLNPEFSNIVISTLSSLEGAGEGSLVFVEHRKFSDALLGSSAVAVFCTNEIVFKVPESMAILVTST
str.CO20_0321  MADTFFFTPSRRLTVANVAELTGAKLLNPEFSNIVISTLSSLEGAGEGSLVFVEHRKFSDALLGSSAVAVFCTNEIVFKVPESMAILVTST
str.RM-11      MADTFFFTPSRRLTVANVAELTGAKLLNPEFSNIVISTLSSLEGAGEGSLVFVEHRKFSDALLGSSAVAVFCTNEIVFKVPESMAILVTST
str.BQ2-D70    MADTFFFTPSRRLTVANVAELTGAKLLNPEFSNIVISTLSSLEGAGEGSLVFVEHRKFSDALLGSSAVAVFCTNEIVFKVPESMAILVTST
str.JK73rel    MADTFFFTPSRRLTVANVAELTGAKLLNPEFSNIVISTLSSLEGAGEGSLVFVEHRKFSDALLGSSAVAVFCTNEIVFKVPESMAILVTST
str.JK7        MADTFFFTPSRRLTVANVAELTGAKLLNPEFSNIVISTLSSLEGAGEGSLVFVEHRKFSDALLGSSAVAVFCTNEIVFKVPESMAILVTST
str.JK73       MADTFFFTPSRRLTVANVAELTGAKLLNPEFSNIVISTLSSLEGAGEGSLVFVEHRKFSDALLGSSAVAVFCTNEIVFKVPESMAILVTST
str.G1712      MADTFFFTPSRRLTVANVAELTGAKLLNPEFSNIVISTLSSLEGAGEGSLVFVEHRKFSDALLGSSAVAVFCTNEIVFKVPESMAILVTST
str.G1713      MADTFFFTPSRRLTVANVAELTGAKLLNPEFSNIVISTLSSLEGAGEGSLVFVEHRKFSDALLGSSAVAVFCTNEIVFKVPESMAILVTST
str.JK31       MADTFFFTPSRRLTVANVAELTGAKLLNPEFSNIVISTLSSLEGAGEGSLVFVEHRKFSDALLGSSAVAVFCTNEIVFKVPESMAILVTST
str.JK68       MADTFFFTPSRRLTVANVAELTGAKLLNPEFSNIVISTLSSLEGAGEGSLVFVEHRKFSDALLGSSAVAVFCTNEIVFKVPESMAILVTST
str.JK39       MADTFFFTPSRRLTVANVAELTGAKLLNPEFSNIVISTLSSLEGAGEGSLVFVEHRKFSDALLGSSAVAVFCTNEIVFKVPESMAILVTST
str.JK63       MADTFFFTPSRRLTVANVAELTGAKLLNPEFSNIVISTLSSLEGAGEGSLVFVEHRKFSDALLGSSAVAVFCTNEIVFKVPESMAILVTST
str.JK19       MADTFFFTPSRRLTVANVAELTGAKLLNPEFSNIVISTLSSLEGAGEGSLVFVEHRKFSDALLGSSAVAVFCTNEIVFKVPESMAILVTST
str.JK67       MADTFFFTPSRRLTVANVAELTGAKLLNPEFSNIVISTLSSLEGAGEGSLVFVEHRKFSDALLGSSAVAVFCTNEIVFKVPESMAILVTST
str.JK56       MADTFFFTPSRRLTVANVAELTGAKLLNPEFSNIVISTLSSLEGAGEGSLVFVEHRKFSDALLGSSAVAVFCTNEIVFKVPESMAILVTST
str.CCUG45777  MADTFFFTPSRRLTVANVAELTGAKLLNPEFSNIVISTLSSLEGAGEGSLVFVEHRKFSDALLGSSAVAVFCTNEIVFKVPESMAILVTST
str.JK12       MADTFFFTPSRRLTVANVAELTGAKLLNPEFSNIVISTLSSLEGAGEGSLVFVEHRKFSDALLGSSAVAVFCTNEIVFKVPESMAILVTST

                       100       110       120       130       140       150       160       170       180
str.Toulouse   PQRDFAQIGRILFPDSVKPMPWFGQREISPYAHIHPSAKFGHDVCIEAGAVIGKNVEIGSGSLISSTAVIGENCRIGRDCYIAPKVTVQYS
str.MF1-1      PQRDFAQIGRILFPDSVKPMPWFGQREISPYAHIHPSAKFGHDVCIEAGAVIGKNVEIGSGSLISSTAVIGENCRIGRDCYIAPKVTVQYS
str.NCTC12899  PQRDFAQIGRILFPDSVKPMPWFGQREISPYAHIHPSAKFGHDVCIEAGAVIGKNVEIGSGSLISSTAVIGENCRIGRDCYIAPKVTVQYS
str.CO20_0257  PQRDFAQIGRILFPDSVKPMPWFGQREISPYAHIHPSAKFGHDVCIEAGAVIGKNVEIGSGSLISSTAVIGENCRIGRDCYIAPKVTVQYS
str.CO20_0256  PQRDFAQIGRILFPDSVKPMPWFGQREISPYAHIHPSAKFGHDVCIEAGAVIGKNVEIGSGSLISSTAVIGENCRIGRDCYIAPKVTVQYS
str.CO20_0297  PQRDFAQIGRILFPDSVKPMPWFGQREISPYAHIHPSAKFGHDVCIEAGAVIGKNVEIGSGSLISSTAVIGENCRIGRDCYIAPKVTVQYS
str.CO21_0024  PQRDFAQIGRILFPDSVKPMPWFGQREISPYAHIHPSAKFGHDVCIEAGAVIGKNVEIGSGSLISSTAVIGENCRIGRDCYIAPKVTVQYS
str.CO20_0321  PQRDFAQIGRILFPDSVKPMPWFGQREISPYAHIHPSAKFGHDVCIEAGAVIGKNVEIGSGSLISSTAVIGENCRIGRDCYIAPKVTVQYS
str.RM-11      PQRDFAQIGRILFPDSVKPMPWFGQREISPYAHIHPSAKFGHDVCIEAGAVIGKNVEIGSGSLISSTAVIGENCRIGRDCYIAPKVTVQYS
str.BQ2-D70    PQRDFAQIGRILFPDSVKPMPWFGQREISPYAHIHPSAKFGHDVCIEAGAVIGKNVEIGSGSLISSTAVIGENCRIGRDCYIAPKVTVQYS
str.JK73rel    PQRDFAQIGRILFPDSVKPMPWFGQREISPYAHIHPSAKFGHDVCIEAGAVIGKNVEIGSGSLISSTAVIGENCRIGRDCYIAPKVTVQYS
str.JK7        PQRDFAQIGRILFPDSVKPMPWFGQREISPYAHIHPSAKFGHDVCIEAGAVIGKNVEIGSGSLISSTAVIGENCRIGRDCYIAPKVTVQYS
str.JK73       PQRDFAQIGRILFPDSVKPMPWFGQREISPYAHIHPSAKFGHDVCIEAGAVIGKNVEIGSGSLISSTAVIGENCRIGRDCYIAPKVTVQYS
str.G1712      PQRDFAQIGRILFPDSVKPMPWFGQREISPYAHIHPSAKFGHDVCIEAGAVIGKNVEIGSGSLISSTAVIGENCRIGRDCYIAPKVTVQYS
str.G1713      PQRDFAQIGRILFPDSVKPMPWFGQREISPYAHIHPSAKFGHDVCIEAGAVIGKNVEIGSGSLISSTAVIGENCRIGRDCYIAPKVTVQYS
str.JK31       PQRDFAQIGRILFPDSVKPMPWFGQREISPYAHIHPSAKFGHDVCIEAGAVIGKNVEIGSGSLISSTAVIGENCRIGRDCYIAPKVTVQYS
str.JK68       PQRDFAQIGRILFPDSVKPMPWFGQREISPYAHIHPSAKFGHDVCIEAGAVIGKNVEIGSGSLISSTAVIGENCRIGRDCYIAPKVTVQYS
str.JK39       PQRDFAQIGRILFPDSVKPMPWFGQREISPYAHIHPSAKFGHDVCIEAGAVIGKNVEIGSGSLISSTAVIGENCRIGRDCYIAPKVTVQYS
str.JK63       PQRDFAQIGRILFPDSVKPMPWFGQREISPYAHIHPSAKFGHDVCIEAGAVIGKNVEIGSGSLISSTAVIGENCRIGRDCYIAPKVTVQYS
str.JK19       PQRDFAQIGRILFPDSVKPMPWFGQREISPYAHIHPSAKFGHDVCIEAGAVIGKNVEIGSGSLISSTAVIGENCRIGRDCYIAPKVTVQYS
str.JK67       PQRDFAQIGRILFPDSVKPMPWFGQREISPYAHIHPSAKFGHDVCIEAGAVIGKNVEIGSGSLISSTAVIGENCRIGRDCYIAPKVTVQYS
str.JK56       PQRDFAQIGRILFPDSVKPMPWFGQREISPYAHIHPSAKFGHDVCIEAGAVIGKNVEIGSGSLISSTAVIGENCRIGRDCYIAPKVTVQYS
str.CCUG45777  PQRDFAQIGRILFPDSVKPMPWFGQREISPYAHIHPSAKFGHDVCIEAGAVIGKNVEIGSGSLISSTAVIGENCRIGRDCYIAPKVTVQYS
str.JK12       PQRDFAQIGRILFPDSVKPMPWFGQREISPYAHIHPSAKFGHDVCIEAGAVIGKNVEIGSGSLISSTAVIGENCRIGRDCYIAPKVTVQYS

                      190       200       210       220       230       240       250       260       270
str.Toulouse   LIGDRVYLYPGTCIGQDGFGYVGGASGIEKVPQLGRVIIKDGVEIGANTTIDRGTFEDTIIGEGSKIDNLVQIAHNVKIGRYCLIAAQCGI
str.MF1-1      LIGDRVYLYPGTCIGQDGFGYVGGASGIEKVPQLGRVIIKDGVEIGANTTIDRGTFEDTIIGEGSKIDNLVQIAHNVKIGRYCLIAAQCGI
str.NCTC12899  LIGDRVYLYPGTCIGQDGFGYVGGASGIEKVPQLGRVIIKDGVEIGANTTIDRGTFEDTIIGEGSKIDNLVQIAHNVKIGRYCLIAAQCGI
str.CO20_0257  LIGDRVYLYPGTCIGQDGFGYVGGASGIEKVPQLGRVIIKDGVEIGANTTIDRGTFEDTIIGEGSKIDNLVQIAHNVKIGRYCLIAAQCGI
str.CO20_0256  LIGDRVYLYPGTCIGQDGFGYVGGASGIEKVPQLGRVIIKDGVEIGANTTIDRGTFEDTIIGEGSKIDNLVQIAHNVKIGRYCLIAAQCGI
str.CO20_0297  LIGDRVYLYPGTCIGQDGFGYVGGASGIEKVPQLGRVIIKDGVEIGANTTIDRGTFEDTIIGEGSKIDNLVQIAHNVKIGRYCLIAAQCGI
str.CO21_0024  LIGDRVYLYPGTCIGQDGFGYVGGASGIEKVPQLGRVIIKDGVEIGANTTIDRGTFEDTIIGEGSKIDNLVQIAHNVKIGRYCLIAAQCGI
str.CO20_0321  LIGDRVYLYPGTCIGQDGFGYVGGASGIEKVPQLGRVIIKDGVEIGANTTIDRGTFEDTIIGEGSKIDNLVQIAHNVKIGRYCLIAAQCGI
str.RM-11      LIGDRVYLYPGTCIGQDGFGYVGGASGIEKVPQLGRVIIKDGVEIGANTTIDRGTFEDTIIGEGSKIDNLVQIAHNVKIGRYCLIAAQCGI
str.BQ2-D70    LIGDRVYLYPGTCIGQDGFGYVGGASGIEKVPQLGRVIIKDGVEIGANTTIDRGTFEDTIIGEGSKIDNLVQIAHNVKIGRYCLIAAQCGI
str.JK73rel    LIGDRVYLYPGTCIGQDGFGYVGGASGIEKVPQLGRVIIKDGVEIGANTTIDRGTFEDTIIGEGSKIDNLVQIAHNVKIGRYCLIAAQCGI
str.JK7        LIGDRVYLYPGTCIGQDGFGYVGGASGIEKVPQLGRVIIKDGVEIGANTTIDRGTFEDTIIGEGSKIDNLVQIAHNVKIGRYCLIAAQCGI
str.JK73       LIGDRVYLYPGTCIGQDGFGYVGGASGIEKVPQLGRVIIKDGVEIGANTTIDRGTFEDTIIGEGSKIDNLVQIAHNVKIGRYCLIAAQCGI
str.G1712      LIGDRVYLYPGTCIGQDGFGYVGGASGIEKVPQLGRVIIKDGVEIGANTTIDRGTFEDTIIGEGSKIDNLVQIAHNVKIGRYCLIAAQCGI
str.G1713      LIGDRVYLYPGTCIGQDGFGYVGGASGIEKVPQLGRVIIKDGVEIGANTTIDRGTFEDTIIGEGSKIDNLVQIAHNVKIGRYCLIAAQCGI
str.JK31       LIGDRVYLYPGTCIGQDGFGYVGGASGIEKVPQLGRVIIKDGVEIGANTTIDRGTFEDTIIGEGSKIDNLVQIAHNVKIGRYCLIAAQCGI
str.JK68       LIGDRVYLYPGTCIGQDGFGYVGGASGIEKVPQLGRVIIKDGVEIGANTTIDRGTFEDTIIGEGSKIDNLVQIAHNVKIGRYCLIAAQCGI
str.JK39       LIGDRVYLYPGTCIGQDGFGYVGGASGIEKVPQLGRVIIKDGVEIGANTTIDRGTFEDTIIGEGSKIDNLVQIAHNVKIGRYCLIAAQCGI
str.JK63       LIGDRVYLYPGTCIGQDGFGYVGGASGIEKVPQLGRVIIKDGVEIGANTTIDRGTFEDTIIGEGSKIDNLVQIAHNVKIGRYCLIAAQCGI
str.JK19       LIGDRVYLYPGTCIGQDGFGYVGGASGIEKVPQLGRVIIKDGVEIGANTTIDRGTFEDTIIGEGSKIDNLVQIAHNVKIGRYCLIAAQCGI
str.JK67       LIGDRVYLYPGTCIGQDGFGYVGGASGIEKVPQLGRVIIKDGVEIGANTTIDRGTFEDTIIGEGSKIDNLVQIAHNVKIGRYCLIAAQCGI
str.JK56       LIGDRVYLYPGTCIGQDGFGYVGGASGIEKVPQLGRVIIKDGVEIGANTTIDRGTFEDTIIGEGSKIDNLVQIAHNVKIGRYCLIAAQCGI
str.CCUG45777  LIGDRVYLYPGTCIGQDGFGYVGGASGIEKVPQLGRVIIKDGVEIGANTTIDRGTFEDTIIGEGSKIDNLVQIAHNVKIGRYCLIAAQCGI
str.JK12       LIGDRVYLYPGTCIGQDGFGYVGGASGIEKVPQLGRVIIKDGVEIGANTTIDRGTFEDTIIGEGSKIDNLVQIAHNVKIGRYCLIAAQCGI

                     280       290       300       310       320       330       340
str.Toulouse   AGSTSIGDMSQLGGSVGVADHIVIGKCVQIAAGSGVMNDIPDGEKWGGSPARPFKQWFREVAALRNIGKVKKEKR
str.MF1-1      AGSTSIGDMSQLGGSVGVADHIVIGKCVQIAAGSGVMNDIPDGEKWGGSPARPFKQWFREVAALRNIGKVKKEKR
str.NCTC12899  AGSTSIGDMSQLGGSVGVADHIVIGKCVQIAAGSGVMNDIPDGEKWGGSPARPFKQWFREVAALRNIGKVKKEKR
str.CO20_0257  AGSTSIGDMSQLGGSVGVADHIVIGKCVQIAAGSGVMNDIPDGEKWGGSPARPFKQWFREVAALRNIGKVKKEKR
str.CO20_0256  AGSTSIGDMSQLGGSVGVADHIVIGKCVQIAAGSGVMNDIPDGEKWGGSPARPFKQWFREVAALRNIGKVKKEKR
str.CO20_0297  AGSTSIGDMSQLGGSVGVADHIVIGKCVQIAAGSGVMNDIPDGEKWGGSPARPFKQWFREVAALRNIGKVKKEKR
str.CO21_0024  AGSTSIGDMSQLGGSVGVADHIVIGKCVQIAAGSGVMNDIPDGEKWGGSPARPFKQWFREVAALRNIGKVKKEKR
str.CO20_0321  AGSTSIGDMSQLGGSVGVADHIVIGKCVQIAAGSGVMNDIPDGEKWGGSPARPFKQWFREVAALRNIGKVKKEKR
str.RM-11      AGSTSIGDMSQLGGSVGVADHIVIGKCVQIAAGSGVMNDIPDGEKWGGSPARPFKQWFREVAALRNIGKVKKEKR
str.BQ2-D70    AGSTSIGDMSQLGGSVGVADHIVIGKCVQIAAGSGVMNDIPDGEKWGGSPARPFKQWFREVAALRNIGKVKKEKR
str.JK73rel    AGSTSIGDMSQLGGSVGVADHIVIGKCVQIAAGSGVMNDIPDGEKWGGSPARPFKQWFREVAALRNIGKVKKEKR
str.JK7        AGSTSIGDMSQLGGSVGVADHIVIGKCVQIAAGSGVMNDIPDGEKWGGSPARPFKQWFREVAALRNIGKVKKEKR
str.JK73       AGSTSIGDMSQLGGSVGVADHIVIGKCVQIAAGSGVMNDIPDGEKWGGSPARPFKQWFREVAALRNIGKVKKEKR
str.G1712      AGSTSIGDMSQLGGSVGVADHIVIGKCVQIAAGSGVMNDIPDGEKWGGSPARPFKQWFREVAALRNIGKVKKEKR
str.G1713      AGSTSIGDMSQLGGSVGVADHIVIGKCVQIAAGSGVMNDIPDGEKWGGSPARPFKQWFREVAALRNIGKVKKEKR
str.JK31       AGSTSIGDMSQLGGSVGVADHIVIGKCVQIAAGSGVMNDIPDGEKWGGSPARPFKQWFREVAALRNIGKVKKEKR
str.JK68       AGSTSIGDMSQLGGSVGVADHIVIGKCVQIAAGSGVMNDIPDGEKWGGSPARPFKQWFREVAALRNIGKVKKEKR
str.JK39       AGSTSIGDMSQLGGSVGVADHIVIGKCVQIAAGSGVMNDIPDGEKWGGSPARPFKQWFREVAALRNIGKVKKEKR
str.JK63       AGSTSIGDMSQLGGSVGVADHIVIGKCVQIAAGSGVMNDIPDGEKWGGSPARPFKQWFREVAALRNIGKVKKEKR
str.JK19       AGSTSIGDMSQLGGSVGVADHIVIGKCVQIAAGSGVMNDIPDGEKWGGSPARPFKQWFREVAALRNIGKVKKEKR
str.JK67       AGSTSIGDMSQLGGSVGVADHIVIGKCVQIAAGSGVMNDIPDGEKWGGSPARPFKQWFREVAALRNIGKVKKEKR
str.JK56       AGSTSIGDMSQLGGSVGVADHIVIGKCVQIAAGSGVMNDIPDGEKWGGSPARPFKQWFREVAALRNIGKVKKEKR
str.CCUG45777  AGSTSIGDMSQLGGSVGVADHIVIGKCVQIAAGSGVMNDIPDGEKWGGSPARPFKQWFREVAALRNIGKVKKEKR
str.JK12       AGSTSIGDMSQLGGSVGVADHIVIGKCVQIAAGSGVMNDIPDGEKWGGSPARPFKQWFREVAALRNIGKVKKEKR
```

>WP_011179451.1 D-alanyl-D-alanine carboxypeptidase [Bartonella quintana str. Toulouse]

```
                1        10        20        30        40        50        60        70        80        90
str.Toulouse    MYINCPKIVHCYKKVAIAFIVLALSCFCASATPQEVYPDKYAAIVIDANTGKTLFQANATLKRYPASLTKMMTLYMLFQAMHMRRVTPNTP
str.CO20_0321   MYINCPKIVHCYKKVAIAFIVLALSCFCASATPQEVYPDKYAAIVIDANTGKTLFQANATLKRYPASLTKMMTLYMLFQAMHMRRVTPNTP
str.G1712       MYINCPKIVHCYKKVAIAFIVLALSCFCASATPQEVYPDKYAAIVIDANTGKTLFQANATLKRYPASLTKMMTLYMLFQAMHMRRVTPNTP
str.G1713       MYINCPKIVHCYKKVAIAFIVLALSCFCASATPQEVYPDKYAAIVIDANTGKTLFQANATLKRYPASLTKMMTLYMLFQAMHMRRVTPNTP
str.CCUG45777   MYINCPKIVHCYKKVAIAFIVLALSCFCASATPQEVYPDKYAAIVIDANTGKTLFQANATLKRYPASLTKMMTLYMLFQAMHMRRVTPNTP
str.J67         MYINCPKIVHCYKKVAIAFIVLALSCFCASATPQEVYPDKYAAIVIDANTGKTLFQANATLKRYPASLTKMMTLYMLFQAMHMRRVTPNTP
str.J56         MYINCPKIVHCYKKVAIAFIVLALSCFCASATPQEVYPDKYAAIVIDANTGKTLFQANATLKRYPASLTKMMTLYMLFQAMHMRRVTPNTP
str.J39         MYINCPKIVHCYKKVAIAFIVLALSCFCASATPQEVYPDKYAAIVIDANTGKTLFQANATLKRYPASLTKMMTLYMLFQAMHMRRVTPNTP
str.J63         MYINCPKIVHCYKKVAIAFIVLALSCFCASATPQEVYPDKYAAIVIDANTGKTLFQANATLKRYPASLTKMMTLYMLFQAMHMRRVTPNTP
str.J31         MYINCPKIVHCYKKVAIAFIVLALSCFCASATPQEVYPDKYAAIVIDANTGKTLFQANATLKRYPASLTKMMTLYMLFQAMHMRRVTPNTP
str.J68         MYINCPKIVHCYKKVAIAFIVLALSCFCASATPQEVYPDKYAAIVIDANTGKTLFQANATLKRYPASLTKMMTLYMLFQAMHMRRVTPNTP
str.BQ2-D70     MYINCPKIVHCYKKVAIAFIVLALSCFCASATPQEVYPDKYAAIVIDANTGKTLFQANATLKRYPASLTKMMTLYMLFQAMHMRRVTPNTP
str.J73rel      MYINCPKIVHCYKKVAIAFIVLALSCFCASATPQEVYPDKYAAIVIDANTGKTLFQANATLKRYPASLTKMMTLYMLFQAMHMRRVTPNTP
str.J7          MYINCPKIVHCYKKVAIAFIVLALSCFCASATPQEVYPDKYAAIVIDANTGKTLFQANATLKRYPASLTKMMTLYMLFQAMHMRRVTPNTP
str.J73         MYINCPKIVHCYKKVAIAFIVLALSCFCASATPQEVYPDKYAAIVIDANTGKTLFQANATLKRYPASLTKMMTLYMLFQAMHMRRVTPNTP
str.J12         MYINCPKIVHCYKKVAIAFIVLALSCFCASATPQEVYPDKYAAIVIDANTGKTLFQANATLKRYPASLTKMMTLYMLFQAMHMRRVTPNTP
str.CO20_0297   MYINCPKIVHCYKKVAIAFIVLALSCFCASATPQEVYPDKYAAIVIDANTGKTLFQANATLKRYPASLTKMMTLYMLFQAMHMRRVTPNTP
str.CO21_0024   MYINCPKIVHCYKKVAIAFIVLALSCFCASATPQEVYPDKYAAIVIDANTGKTLFQANATLKRYPASLTKMMTLYMLFQAMHMRRVTPNTP
str.NCTC12899   MYINCPKIVHCYKKVAIAFIVLALSCFCASATPQEVYPDKYAAIVIDANTGKTLFQANATLKRYPASLTKMMTLYMLFQAMHMRRVTPNTP
str.CO20_0257   MYINCPKIVHCYKKVAIAFIVLALSCFCASATPQEVYPDKYAAIVIDANTGKTLFQANATLKRYPASLTKMMTLYMLFQAMHMRRVTPNTP
str.CO20_0256   MYINCPKIVHCYKKVAIAFIVLALSCFCASATPQEVYPDKYAAIVIDANTGKTLFQANATLKRYPASLTKMMTLYMLFQAMHMRRVTPNTP
str.J19         MYINCPKIVHCYKKVAISFIVLALSCFCASATPQEVYPDKYAAIVIDANTGKTLFQANATLKRYPASLTKMMTLYMLFQAMHMRRVTPNTP
str.RM-11       MYINCPKIVHCYKKVAIAFIVLALSCFCASATPQEVYPDKYAAIVIDANTGKTLFQANATLKRYPASLTKMMTLYMLFQAMHMRRVTPNTP
str.MF1-1       MYINCPKIVHCYKKVAIAFIVLALSCFCASATPQEVYPDKYAAIVIDANTGKTLFQANATLKRYPASLTKMMTLYMLFQAMHMRRVTPNTP

                         100       110       120       130       140       150       160       170       180
str.Toulouse    IPVSRNAAARPPTKLGLKAGQTISAQEAAKALITRSANDVATAVAEYLGGNEKKFARMMTAKARKLGMTNTHFANASGLPDLRNYSTARDM
str.CO20_0321   IPVSRNAAARPPTKLGLKAGQTISAQEAAKALITRSANDVATAVAEYLGGNEKKFARMMTAKARKLGMTNTHFANASGLPDLRNYSTARDM
str.G1712       IPVSRNAAARPPTKLGLKAGQTISAQEAAKALITRSANDIATAVAEYLGGNEKKFARMMTAKARKLGMTNTHFANASGLPDLRNYSTARDM
str.G1713       IPVSRNAAARPPTKLGLKAGQTISAQEAAKALITRSANDIATAVAEYLGGNEKKFARMMTAKARKLGMTNTHFANASGLPDLRNYSTARDM
str.CCUG45777   IPVSRNAAARPPTKLGLKAGQTISAQEAAKALITRSANDIATAVAEYLGGNEKKFARMMTAKARKLGMTNTHFANASGLPDLRNYSTARDM
str.J67         IPVSRNAAARPPTKLGLKAGQTISAQEAAKALITRSANDVATAVAEYLGGNEKKFARMMTAKARKLGMTNTHFANASGLPDLRNYSTARDM
str.J56         IPVSRNAAARPPTKLGLKAGQTISAQEAAKALITRSANDVATAVAEYLGGNEKKFARMMTAKARKLGMTNTHFANASGLPDLRNYSTARDM
str.J39         IPVSRNAAARPPTKLGLKAGQTISAQEAAKALITRSANDVATAVAEYLGGNEKKFARMMTAKARKLGMTNTHFANASGLPDLRNYSTARDM
str.J63         IPVSRNAAARPPTKLGLKAGQTISAQEAAKALITRSANDVATAVAEYLGGNEKKFARMMTAKARKLGMTNTHFANASGLPDLRNYSTARDM
str.J31         IPVSRNAAARPPTKLGLKAGQTISAQEAAKALITRSANDVATAVAEYLGGNEKKFARMMTAKARKLGMTNTHFANASGLPDLRNYSTARDM
str.J68         IPVSRNAAARPPTKLGLKAGQTISAQEAAKALITRSANDVATAVAEYLGGNEKKFARMMTAKARKLGMTNTHFANASGLPDLRNYSTARDM
str.BQ2-D70     IPVSRNAAARPPTKLGLKAGQTISAQEAAKALITRSANDVATAVAEYLGGNEKKFARMMTAKARKLGMTNTHFANASGLPDLRNYSTARDM
str.J73rel      IPVSRNAAARPPTKLGLKAGQTISAQEAAKALITRSANDVATAVAEYLGGNEKKFARMMTAKARKLGMTNTHFANASGLPDLRNYSTARDM
str.J7          IPVSRNAAARPPTKLGLKAGQTISAQEAAKALITRSANDVATAVAEYLGGNEKKFARMMTAKARKLGMTNTHFANASGLPDLRNYSTARDM
str.J73         IPVSRNAAARPPTKLGLKAGQTISAQEAAKALITRSANDVATAVAEYLGGNEKKFARMMTAKARKLGMTNTHFANASGLPDLRNYSTARDM
str.J12         IPVSRNAAARPPTKLGLKAGQTISAQEAAKALITRSANDVATAVAEYLGGNEKKFARMMTAKARKLGMTNTHFANASGLPDLRNYSTARDM
str.CO20_0297   IPVSRNAAARPPTKLGLKAGQTISAQEAAKALITRSANDVATAVAEYLGGNEKKFARMMTAKARKLGMTNTHFANASGLPDLRNYSTARDM
str.CO21_0024   IPVSRNAAARPPTKLGLKAGQTISAQEAAKALITRSANDVATAVAEYLGGNEKKFARMMTAKARKLGMTNTHFANASGLPDLRNYSTARDM
str.NCTC12899   IPVSRNAAARPPTKLGLKAGQTISAQEAAKALITRSANDVATAVAEYLGGNEKKFARMMTAKARKLGMTNTHFANASGLPDLRNYSTARDM
str.CO20_0257   IPVSRNAAARPPTKLGLKAGQTISAQEAAKALITRSANDVATAVAEYLGGNEKKFARMMTAKARKLGMTNTHFANASGLPDLRNYSTARDM
str.CO20_0256   IPVSRNAAARPPTKLGLKAGQTISAQEAAKALITRSANDVATAVAEYLGGNEKKFARMMTAKARKLGMTNTHFANASGLPDLRNYSTARDM
str.J19         IPVSRNAAARPPTKLGLKAGQTISAQEAAKALITRSANDVATAVAEYLGGNEKKFARMMTAKARKLGMTNTHFANASGLPDLRNYSTARDM
str.RM-11       IPVSRNAAARPPTKLGLKAGQTISAQEAAKALITRSANDVATAIAEYLGGNEKKFARMMTAKARKLGMTNTHFANASGLPDLRNYSTARDM
str.MF1-1       IPVSRNAAARPPTKLGLKAGQTISAQEAAKALITRSANDVATAIAEYLGGNEKKFARMMTAKARKLGMTNTHFANASGLPDLRNYSTARDM

                         190       200       210       220       230       240       250       260       270
str.Toulouse    ATLSLALRKHFPQQYKLFKTKSFVFRGHTVKSHNKLVKTMKGVDGIKTGYTQMSGSNLATSMHIEGRSLVAVVMGGKSSTARDRHMANLLS
str.CO20_0321   ATLSLALRKHFPQQYKLFKTKSFVFRGHTVKSHNKLVKTMKGVDGIKTGYTQMSGSNLATSMHIEGRSLVAVVMGGKSSTARDRHMANLLS
str.G1712       ATLSLALRKHFPQQYKLFKTKSFVFRGHTVKSHNKLVKTMKGVDGIKTGYTQMSGSNLATSMHIEGRSLVAVVMGGKSSTARDRHMANLLS
str.G1713       ATLSLALRKHFPQQYKLFKTKSFVFRGHTVKSHNKLVKTMKGVDGIKTGYTQMSGSNLATSMHIEGRSLVAVVMGGKSSTARDRHMANLLS
str.CCUG45777   ATLSLALRKHFPQQYKLFKTKSFVFRGHTVKSHNKLVKTMKGVDGIKTGYTQMSGSNLATSMHIEGRSLVAVVMGGKSSTARDRHMANLLS
str.J67         ATLSLALRKHFPQQYKLFKTKSFVFRGHTVKSHNKLVKTMKGVDGIKTGYTQMSGSNLATSMHIEGRSLVAVVMGGKSSTARDRHMANLLS
str.J56         ATLSLALRKHFPQQYKLFKTKSFVFRGHTVKSHNKLVKTMKGVDGIKTGYTQMSGSNLATSMHIEGRSLVAVVMGGKSSTARDRHMANLLS
str.J39         ATLSLALRKHFPQQYKLFKTKSFVFRGHTVKSHNKLVKTMKGVDGIKTGYTQMSGSNLATSMHIEGRSLVAVVMGGKSSTARDRHMANLLS
str.J63         ATLSLALRKHFPQQYKLFKTKSFVFRGHTVKSHNKLVKTMKGVDGIKTGYTQMSGSNLATSMHIEGRSLVAVVMGGKSSTARDRHMANLLS
str.J31         ATLSLALRKHFPQQYKLFKTKSFVFRGHTVKSHNKLVKTMKGVDGIKTGYTQMSGSNLATSMHIEGRSLVAVVMGGKSSTARDRHMANLLS
str.J68         ATLSLALRKHFPQQYKLFKTKSFVFRGHTVKSHNKLVKTMKGVDGIKTGYTQMSGSNLATSMHIEGRSLVAVVMGGKSSTARDRHMANLLS
str.BQ2-D70     ATLSLALRKHFPQQYKLFKTKSFVFRGHTVKSHNKLVKTMKGVDGIKTGYTQMSGSNLATSMHIEGRSLVAVVMGGKSSTARDRHMANLLS
str.J73rel      ATLSLALRKHFPQQYKLFKTKSFVFRGHTVKSHNKLVKTMKGVDGIKTGYTQMSGSNLATSMHIEGRSLVAVVMGGKSSTARDRHMANLLS
str.J7          ATLSLALRKHFPQQYKLFKTKSFVFRGHTVKSHNKLVKTMKGVDGIKTGYTQMSGSNLATSMHIEGRSLVAVVMGGKSSTARDRHMANLLS
str.J73         ATLSLALRKHFPQQYKLFKTKSFVFRGHTVKSHNKLVKTMKGVDGIKTGYTQMSGSNLATSMHIEGRSLVAVVMGGKSSTARDRHMANLLS
str.J12         ATLSLALRKHFPQQYKLFKTKSFVFRGHTVKSHNKLVKTMKGVDGIKTGYTQMSGSNLATSMHIEGRSLVAVVMGGKSSTARDRHMANLLS
str.CO20_0297   ATLSLALRKHFPQQYKLFKTKSFVFRGHTVKSHNKLVKTMKGVDGIKTGYTQMSGSNLATSMHIEGRSLVAVVMGGKSSTARDRHMANLLS
str.CO21_0024   ATLSLALRKHFPQQYKLFKTKSFVFRGHTVKSHNKLVKTMKGVDGIKTGYTQMSGSNLATSMHIEGRSLVAVVMGGKSSTARDRHMANLLS
str.NCTC12899   ATLSLALRKHFPQQYKLFKTKSFVFRGHTVKSHNKLVKTMKGVDGIKTGYTQMSGSNLATSMHIEGRSLVAVVMGGKSSTARDRHMANLLS
str.CO20_0257   ATLSLALRKHFPQQYKLFKTKSFVFRGHTVKSHNKLVKTMKGVDGIKTGYTQMSGSNLATSMHIEGRSLVAVVMGGKSSTARDRHMANLLS
str.CO20_0256   ATLSLALRKHFPQQYKLFKTKSFVFRGHTVKSHNKLVKTMKGVDGIKTGYTQMSGSNLATSMHIEGRSLVAVVMGGKSSTARDRHMANLLS
str.J19         ATLSLALRKHFPQQYKLFKTKSFVFRGHTVKSHNKLVKTMKGVDGIKTGYTQMSGSNLATSMHIEGRSLVAVVMGGKSSTARDRHMANLLS
str.RM-11       ATLSLALRKHFPQQYKLFKTKSFVFRGHTVKSHNKLVKTMKGVDGIKTGYTQMSGSNLATSMRIEGRSLVAVVMGGKSSTARDRHMANLLS
str.MF1-1       ATLSLALRKHFPQQYKLFKIKSFVFRGHTVKSHNKLLKTMKGVDGIKTGYTQMSGSNLATSMRIEGRSLVAVVMGGKSSTARDRHMANLLS

                         280       290       300       310       320       330       340       350       360
str.Toulouse    QYLPKANSMKNDGRLMASAHYNLPTGANIPIPTVKADPTDSDDEISTILTAFAEQPRDFNIAIAAVNQAIIPIPNPQKVPPVRTDKIVTAS
str.CO20_0321   QYLPKANSMKNDGRLMASAHYNLPTGANIPIPTVKADPTDSDDEISTILTAFAEQPRDFNIAIAAVNQAIIPIPNPQKVPPVRTDKIVTAS
str.G1712       QYLPKANSMKNDGRLMASAHYNLPTGANIPIPTVKADPTDSDDEISTILTAFAEQPRDFNIAIAAVNQAIIPIPNPQKVPPVRTDKIVTAS
str.G1713       QYLPKANSMKNDGRLMASAHYNLPTGANIPIPTVKADPTDSDDEISTILTAFAEQPRDFNIAIAAVNQAIIPIPNPQKVPPVRTDKIVTAS
str.CCUG45777   QYLPKANSMKNDGRLMASAHYNLPTGANIPIPTVKADPTDSDDEISTILTAFAEQPRDFNIAIAAVNQAIIPIPNPQKVPPVRTDKIVTAS
str.J67         QYLPKANSMKNDGRLMASAHYNLPTGANIPIPTVKADPTDSDDEISTILTAFAEQPRDFNIAIAAVNQAIIPIPNPQKVPPVRTDKIVTAS
str.J56         QYLPKANSMKNDGRLMASAHYNLPTGANIPIPTVKADPTDSDDEISTILTAFAEQPRDFNIAIAAVNQAIIPIPNPQKVPPVRTDKIVTAS
str.J39         QYLPKANSMKNDGRLMASAHYNLPTGANIPIPTVKADPTDSDDEISTILTAFAEQPRDFNIAIAAVNQAIIPIPNPQKVPPVRTDKIVTAS
str.J63         QYLPKANSMKNDGRLMASAHYNLPTGANIPIPTVKADPTDSDDEISTILTAFAEQPRDFNIAIAAVNQAIIPIPNPQKVPPVRTDKIVTAS
str.J31         QYLPKANSMKNDGRLMASAHYNLPTGANIPIPTVKADPTDSDDEISTILTAFAEQPRDFNIAIAAVNQAIIPIPNPQKVPPVRTDKIVTAS
str.J68         QYLPKANSMKNDGRLMASAHYNLPTGANIPIPTVKADPTDSDDEISTILTAFAEQPRDFNIAIAAVNQAIIPIPNPQKVPPVRTDKIVTAS
str.BQ2-D70     QYLPKANSMKNDGRLMASAHYNLPTGANIPIPTVKADPTDSDDEISTILTAFAEQPRDFNIAIAAVNQAIIPIPNPQKVPPVRTDKIVTAS
str.J73rel      QYLPKANSMKNDGRLMASAHYNLPTGANIPIPTVKADPTDSDDEISTILTAFAEQPRDFNIAIAAVNQAIIPIPNPQKVPPVRTDKIVTAS
str.J7          QYLPKANSMKNDGRLMASAHYNLPTGANIPIPTVKADPTDSDDEISTILTAFAEQPRDFNIAIAAVNQAIIPIPNPQKVPPVRTDKIVTAS
str.J73         QYLPKANSMKNDGRLMASAHYNLPTGANIPIPTVKADPTDSDDEISTILTAFAEQPRDFNIAIAAVNQAIIPIPNPQKVPPVRTDKIVTAS
str.J12         QYLPKANSMKNDGRLMASAHYNLPTGANIPIPTVKADPTDSDDEISTILTAFAEQPRDFNIAIAAVNQAIIPIPNPQKVPPVRTDKIVTAS
str.CO20_0297   QYLPKANSMKNDGRLMASAHYNLPTGANIPIPTVKADPTDSDDEISTILTAFAEQPRDFNIAIAAVNQAIIPIPNPQKVPPVRTDKIVTAS
str.CO21_0024   QYLPKANSMKNDGRLMASAHYNLPTGANIPIPTVKADPTDSDDEISTILTAFAEQPRDFNIAIAAVNQAIIPIPNPQKVPPVRTDKIVTAS
str.NCTC12899   QYLPKANSMKNDGRLMASAHYNLPTGANIPIPTVKADPTDSDDEISTILTAFAEQPRDFNIAIAAVNQAIIPIPNPQKVPPVRTDKIVTAS
str.CO20_0257   QYLPKANSMKNDGRLMASAHYNLPTGANIPIPTVKADPTDSDDEISTILTAFAEQPRDFNIAIAAVNQAIIPIPNPQKVPPVRTDKIVTAS
str.CO20_0256   QYLPKANSMKNDGRLMASAHYNLPTGANIPIPTVKADPTDSDDEISTILTAFAEQPRDFNIAIAAVNQAIIPIPNPQKVPPVRTDKIVTAS
str.J19         QYLPKANSMKNDGRLMASAHYNLPTGANIPIPTVKADPTDSDDEISTILTAFAEQPRDFNIAIAAVNQAIIPIPNPQKVPPVRTDKIVTAS
str.RM-11       QYLPKANSMKNDGRLMASAHYNLPTGANIPIPTVKADPTDSDDEISTILTAFAEQPKDFNIAIAAVNQAIIPIPNPQKIPPVRTDKIVTAS
str.MF1-1       QYLPKANSMKNDGRLMASAHYNLPTGANIPIPTVKADPTDSDDEISTILTAFAEQPKDFNIAIAAVNQAIIPIPNPQKVPPVRTDKIVTAS
```

```
                  370       380       390       400       410       420       430       440       450
str.Toulouse      LPTSAGWAIQIGSLPSKEQAKTLLLKAKNTAYSALKHASSHMQLFEKSGHRYYRARFIGFQSKKAAFNACSTLKKANFNCYTVVAH
str.CO20_0321     LPTSAGWAIQIGSLPSKEQAKTLLLKAKNTAYSALKHASSHMQLFEKSGHRYYRARFIGFQSKKAAFNACSTLKKANFNCYTVVAH
str.G1712         LPTSAGWAIQIGSLPSKEQAKTLLLKAKNTAYSALKHASSHMQLFEKSGHRYYRARFIGFQSKKAAFNACSTLKKANFNCYTVVAH
str.G1713         LPTSAGWAIQIGSLPSKEQAKTLLLKAKNTAYSALKHASSHMQLFEKSGHRYYRARFIGFQSKKAAFNACSTLKKANFNCYTVVAH
str.CCUG45777     LPTSAGWAIQIGSLPSKEQAKTLLLKAKNTAYSALKHASSHMQLFEKSGHRYYRARFIGFQSKKAAFNACSTLKKANFNCYTVVAH
str.J67           LPTGAGWAIQIGSLPSKEQAKTLLLKAKNTAYSALKHASSHMQLFEKSGHRYYRARFIGFQSKKAAFNACSTLKKANFNCYTVVAH
str.J56           LPTGAGWAIQIGSLPSKEQAKTLLLKAKNTAYSALKHASSHMQLFEKSGHRYYRARFIGFQSKKAAFNACSTLKKANFNCYTVVAH
str.J39           LPTGAGWAIQIGSLPSKEQAKTLLLKAKNTAYSALKHASSHMQLFEKSGHRYYRARFIGFQSKKAAFNACSTLKKANFNCYTVVAH
str.J63           LPTGAGWAIQIGSLPSKEQAKTLLLKAKNTAYSALKHASSHMQLFEKSGHRYYRARFIGFQSKKAAFNACSTLKKANFNCYTVVAH
str.J31           LPTGAGWAIQIGSLPSKEQAKTLLLKAKNTAYSALKHASSHMQLFEKSGHRYYRARFIGFQSKKAAFNACSTLKKANFNCYTVVAH
str.J68           LPTGAGWAIQIGSLPSKEQAKTLLLKAKNTAYSALKHASSHMQLFEKSGHRYYRARFIGFQSKKAAFNACSTLKKANFNCYTVVAH
str.BQ2-D70       LPTGAGWAIQIGSLPSKEQAKTLLLKAKNTAYSALKHASSHMQLFEKSGHRYYRARFIGFQSKKAAFNACSTLKKANFNCYTVVAH
str.J73rel        LPTGAGWAIQIGSLPSKEQAKTLLLKAKNTAYSALKHASSHMQLFEKSGHRYYRARFIGFQSKKAAFNACSTLKKANFNCYTVVAH
str.J7            LPTGAGWAIQIGSLPSKEQAKTLLLKAKNTAYSALKHASSHMQLFEKSGHRYYRARFIGFQSKKAAFNACSTLKKANFNCYTVVAH
str.J73           LPTGAGWAIQIGSLPSKEQAKTLLLKAKNTAYSALKHASSHMQLFEKSGHRYYRARFIGFQSKKAAFNACSTLKKANFNCYTVVAH
str.J12           LPTGAGWAIQIGSLPSKEQAKTLLLKAKNTAYSALKHASSHMQLFEKSGHRYYRARFIGFQSKKAAFNACSTLKKANFNCYTVVAH
str.CO20_0297     LPTGAGWAIQIGSLPSKEQAKTLLLKAKNTAYSALKHASSHMQLFEKSGHRYYRARFIGFQSKKAAFNACSTLKKANFNCYTVVAH
str.CO21_0024     LPTGAGWAIQIGSLPSKEQAKTLLLKAKNTAYSALKHASSHMQLFEKSGHRYYRARFIGFQSKKAAFNACSTLKKANFNCYTVVAH
str.NCTC12899     LPTGAGWAIQIGSLPSKEQAKTLLLKAKNTAYSALKHASSHMQLFEKSGHRYYRARFIGFQSKKAAFNACSTLKKANFNCYTVVAH
str.CO20_0257     LPTGAGWAIQIGSLPSKEQAKTLLLKAKNTAYSALKHASSHMQLFEKSGHRYYRARFIGFQSKKAAFNACSTLKKANFNCYTVVAH
str.CO20_0256     LPTGAGWAIQIGSLPSKEQAKTLLLKAKNTAYSALKHASSHMQLFEKSGHRYYRARFIGFQSKKAAFNACSTLKKANFNCYTVVAH
str.J19           LPTGAGWAIQIGSLPSKEQAKTLLLKAKNTAYSALKHASSHMQLFEKSGHRYYRARFIGFQSKKAAFNACSTLKKANFNCYTVVAH
str.RM-11         LPTNAGWAIQIGSLPSKEQAKTLLLKAKNTAYSALKHASSHMQLFEKSGHRYYRARFIGFQSKKAAFNACSTLKKANFNCYTVVAH
str.MF1-1         LPTNAGWAIQIGSLPSKEQAKTLLLKAKNTAYSALKHASSHMQLFEKSGHRYYRARFIGFQSKKAAFNACSTLKKANFNCYTVVAH
```

>WP_011179558.1 transporter substrate-binding domain-containing protein [Bartonella quintana str. Toulouse]

```
                1        10        20        30        40        50        60        70        80        90
str.Toulouse    MKKSLSIFTAVTVTVMGLIGIAKAENDTLEKIKKTGEITLGVRESSGLAYALGNGKYVGFHTEMAERIIDDISKKIGKPVKIHYLPITSQN
str.JK12        MKKSLSIFTAVTVTVMGLIGIAKAENDTLEKIKKTGEITLGVRESSGLAYALGNGKYVGFHTEMAERIIDDISKKIGKPVKIHYLPITSQN
str.CCUG45777   MKKSLSIFTAVTVTVMGLIGIAKAENDTLEKIKKTGEITLGVRESSGLAYALGNGKYVGFHTEMAERIIDDISKKIGKPVKIHYLPITSQN
str.JK56        MKKSLSIFTAVTVTVMGLIGIAKAENDTLEKIKKTGEITLGVRESSGLAYALGNGKYVGFHTEMAERIIDDISKKIGKPVKIHYLPITSQN
str.JK67        MKKSLSIFTAVTVTVMGLIGIAKAENDTLEKIKKTGEITLGVRESSGLAYALGNGKYVGFHTEMAERIIDDISKKIGKPVKIHYLPITSQN
str.JK19        MKKSLSIFTAVTVTVMGLIGIAKAENDTLEKIKKTGEITLGVRESSGLAYALGNGKYVGFHTEMAERIIDDISKKIGKPVKIHYLPITSQN
str.JK63        MKKSLSIFTAVTVTVMGLIGIAKAENDTLEKIKKTGEITLGVRESSGLAYALGNGKYVGFHTEMAERIIDDISKKIGKPVKIHYLPITSQN
str.JK39        MKKSLSIFTAVTVTVMGLIGIAKAENDTLEKIKKTGEITLGVRESSGLAYALGNGKYVGFHTEMAERIIDDISKKIGKPVKIHYLPITSQN
str.JK68        MKKSLSIFTAVTVTVMGLIGIAKAENDTLEKIKKTGEITLGVRESSGLAYALGNGKYVGFHTEMAERIIDDISKKIGKPVKIHYLPITSQN
str.JK31        MKKSLSIFTAVTVTVMGLIGIAKAENDTLEKIKKTGEITLGVRESSGLAYALGNGKYVGFHTEMAERIIDDISKKIGKPVKIHYLPITSQN
str.G1713       MKKSLSIFTAVTVTVMGLIGIAKAENDTLEKIKKTGEITLGVRESSGLAYALGNGKYVGFHTEMAERIIDDISKKIGKPVKIHYLPITSQN
str.G1712       MKKSLSIFTAVTVTVMGLIGIAKAENDTLEKIKKTGEITLGVRESSGLAYALGNGKYVGFHTEMAERIIDDISKKIGKPVKIHYLPITSQN
str.JK7         MKKSLSIFTAVTVTVMGLIGIAKAENDTLEKIKKTGEITLGVRESSGLAYALGNGKYVGFHTEMAERIIDDISKKIGKPVKIHYLPITSQN
str.BQ2-D70     MKKSLSIFTAVTVTVMGLIGIAKAENDTLEKIKKTGEITLGVRESSGLAYALGNGKYVGFHTEMAERIIDDISKKIGKPVKIHYLPITSQN
str.CO20_0321   MKKSLSIFTAVTVTVMGLIGIAKAENDTLEKIKKTGEITLGVRESSGLAYALGNGKYVGFHTEMAERIIDDISKKIGKPVKIHYLPITSQN
str.CO21_0024   MKKSLSIFTAVTVTVMGLIGIAKAENDTLEKIKKTGEITLGVRESSGLAYALGNGKYVGFHTEMAERIIDDISKKIGKPVKIHYLPITSQN
str.CO20_0297   MKKSLSIFTAVTVTVMGLIGIAKAENDTLEKIKKTGEITLGVRESSGLAYALGNGKYVGFHTEMAERIIDDISKKIGKPVKIHYLPITSQN
str.CO20_0256   MKKSLSIFTAVTVTVMGLIGIAKAENDTLEKIKKTGEITLGVRESSGLAYALGNGKYVGFHTEMAERIIDDISKKIGKPVKIHYLPITSQN
str.CO20_0257   MKKSLSIFTAVTVTVMGLIGIAKAENDTLEKIKKTGEITLGVRESSGLAYALGNGKYVGFHTEMAERIIDDISKKIGKPVKIHYLPITSQN
str.NCTC12899   MKKSLSIFTAVTVTVMGLIGIAKAENDTLEKIKKTGEITLGVRESSGLAYALGNGKYVGFHTEMAERIIDDISKKIGKPVKIHYLPITSQN
str.JK73rel     MKKSLSIFTAVTVTVMGLIGIAKAENDTLEKIKKTGEITLGVRESSGLAYALGNGKYVGFHTEMAERIIDDISKKIGKPVKIHYLPITSQN
str.JK73        MKKSLSIFTAVTVTVMGLIGIAKAENDTLEKIKKTGEITLGVRESSGLAYALGNGKYVGFHTEMAERIIDDISKKIGKPVKIHYLPITSQN
str.MF1-1       MKKSLSIFTAVTVTVMGLIGIAKAENDTLEKIKKTGEITLGVRESSGLAYALGNGKYVGFHTEMAERIIDDISKKIGKPIKIHYLPITSQN
str.RM-11       MKKSLSIFTAVTVTVMGLIGIAKAENDTLEKIKKTGEITLGVRESSGLAYALGNGKYVGFHTEMAERIIDDISKKIGKPIKIHYLPITSQN
```

```
                         100       110       120       130       140       150       160       170       180
str.Toulouse    RIPLLKNSTYDFECGSTTNDVSSSKEAAFAYTTYVEDVRIAVKKNSNIKSFDDLNGKTVATTGTTSVQIIRKNQRSKNINFNVVKGKDHG
str.JK12        RIPLLKNSTYDFECGSTTNDVSSSKEAAFAYTTYVEDVRIAVKKNSNIKSFDDLNGKTVATTGTTSVQIIRKNQRSKNINFNVVKGKDHG
str.CCUG45777   RIPLLKNSTYDFECGSTTNDVSSSKEAAFAYTTYVEDVRIAVKKNSNIKSFDDLNGKTVATTGTTSVQIIRKNQRSKNINFNVVKGKDHG
str.JK56        RIPLLKNSTYDFECGSTTNDVSSSKEAAFAYTTYVEDVRIAVKKNSNIKSFDDLNGKTVATTGTTSVQIIRKNQRSKNINFNVVKGKDHG
str.JK67        RIPLLKNSTYDFECGSTTNDVSSSKEAAFAYTTYVEDVRIAVKKNSNIKSFDDLNGKTVATTGTTSVQIIRKNQRSKNINFNVVKGKDHG
str.JK19        RIPLLKNSTYDFECGSTTNDVSSSKEAAFAYTTYVEDVRIAVKKNSNIKSFDDLNGKTVATTGTTSVQIIRKNQRSKNINFNVVKGKDHG
str.JK63        RIPLLKNSTYDFECGSTTNDVSSSKEAAFAYTTYVEDVRIAVKKNSNIKSFDDLNGKTVATTGTTSVQIIRKNQRSKNINFNVVKGKDHG
str.JK39        RIPLLKNSTYDFECGSTTNDVSSSKEAAFAYTTYVEDVRIAVKKNSNIKSFDDLNGKTVATTGTTSVQIIRKNQRSKNINFNVVKGKDHG
str.JK68        RIPLLKNSTYDFECGSTTNDVSSSKEAAFAYTTYVEDVRIAVKKNSNIKSFDDLNGKTVATTGTTSVQIIRKNQRSKNINFNVVKGKDHG
str.JK31        RIPLLKNSTYDFECGSTTNDVSSSKEAAFAYTTYVEDVRIAVKKNSNIKSFDDLNGKTVATTGTTSVQIIRKNQRSKNINFNVVKGKDHG
str.G1713       RIPLLKNSTYDFECGSTTNDVSSSKEAAFAYTTYVEDVRIAVKKNSNIKSFDDLNGKTVATTGTTSVQIIRKNQRSKNINFNVVKGKDHG
str.G1712       RIPLLKNSTYDFECGSTTNDVSSSKEAAFAYTTYVEDVRIAVKKNSNIKSFDDLNGKTVATTGTTSVQIIRKNQRSKNINFNVVKGKDHG
str.JK7         RIPLLKNSTYDFECGSTTNDVSSSKEAAFAYTTYVEDVRIAVKKNSNIKSFDDLNGKTVATTGTTSVQIIRKNQRSKNINFNVVKGKDHG
str.BQ2-D70     RIPLLKNSTYDFECGSTTNDVSSSKEAAFAYTTYVEDVRIAVKKNSNIKSFDDLNGKTVATTGTTSVQIIRKNQRSKNINFNVVKGKDHG
str.CO20_0321   RIPLLKNSTYDFECGSTTNDVSSSKEAAFAYTTYVEDVRIAVKKNSNIKSFDDLNGKTVATTGTTSVQIIRKNQRSKNINFNVVKGKDHG
str.CO21_0024   RIPLLKNSTYDFECGSTTNDVSSSKEAAFAYTTYVEDVRIAVKKNSNIKSFDDLNGKTVATTGTTSVQIIRKNQRSKNINFNVVKGKDHG
str.CO20_0297   RIPLLKNSTYDFECGSTTNDVSSSKEAAFAYTTYVEDVRIAVKKNSNIKSFDDLNGKTVATTGTTSVQIIRKNQRSKNINFNVVKGKDHG
str.CO20_0256   RIPLLKNSTYDFECGSTTNDVSSSKEAAFAYTTYVEDVRIAVKKNSNIKSFDDLNGKTVATTGTTSVQIIRKNQRSKNINFNVVKGKDHG
str.CO20_0257   RIPLLKNSTYDFECGSTTNDVSSSKEAAFAYTTYVEDVRIAVKKNSNIKSFDDLNGKTVATTGTTSVQIIRKNQRSKNINFNVVKGKDHG
str.NCTC12899   RIPLLKNSTYDFECGSTTNDVSSSKEAAFAYTTYVEDVRIAVKKNSNIKSFDDLNGKTVATTGTTSVQIIRKNQRSKNINFNVVKGKDHG
str.JK73rel     RIPLLKNSTYDFECGSTTNDVSSSKEAAFAYTTYVEDVRIAVKKNSNIKSFDDLNGKTVATTGTTSVQIIRKNQRSKNINFNVVKGKDHG
str.JK73        RIPLLKNSTYDFECGSTTNDVSSSKEAAFAYTTYVEDVRIAVKKNSNIKSFDDLNGKTVATTGTTSVQIIRKNQRSKNINFNVVKGKDHG
str.MF1-1       RIPLLKNSTYDFECGSTTNDVSSSKEAAFAYTTYVEDVRIAVKKNSNIKSFDDLNGKTVATTGTTSVQIIRKNQRSKNINFNVVKGKDHG
str.RM-11       RIPLLKNSTYDFECGSTTNDVSSSKEAAFAYTTYVEDVRIAVKKNSNIKSFDDLNGKTVATTGTTSVQIIRKNQRSKNINFNVVKGKDHG
```

```
                        190       200       210       220       230       240       250       260       270
str.Toulouse    DSFLLLESGRADAFIMDASILAGHIAKSKNPSDYIILDTVLSVEPIACMLRLNDKNLKQAINDSIVRQIKDESLKKLHDKWFMKPTPPANI
str.JK12        DSFLLLESGRADAFIMDASILAGHIAKSKNPSDYIILDTVLSVEPIACMLRLNDKNLKQAINDSIVRQIKDESLKKLHDKWFMKPTPPANI
str.CCUG45777   DSFLLLESGRADAFIMDASILAGHIAKSKNPSDYIILDTVLSVEPIACMLRLNDKNLKQAINDSIVRQIKDESLKKLHDKWFMKPTPPANI
str.JK56        DSFLLLESGRADAFIMDASILAGHIAKSKNPSDYIILDTVLSVEPIACMLRLNDKNLKQAINDSIVRQIKDESLKKLHDKWFMKPTPPANI
str.JK67        DSFLLLESGRADAFIMDASILAGHIAKSKNPSDYIILDTVLSVEPIACMLRLNDKNLKQAINDSIVRQIKDESLKKLHDKWFMKPTPPANI
str.JK19        DSFLLLESGRADAFIMDASILAGHIAKSKNPSDYIILDTVLSVEPIACMLRLNDKNLKQAINDSIVRQIKDESLKKLHDKWFMKPTPPANI
str.JK63        DSFLLLESGRADAFIMDASILAGHIAKSKNPSDYIILDTVLSVEPIACMLRLNDKNLKQAINDSIVRQIKDESLKKLHDKWFMKPTPPANI
str.JK39        DSFLLLESGRADAFIMDASILAGHIAKSKNPSDYIILDTVLSVEPIACMLRLNDKNLKQAINDSIVRQIKDESLKKLHDKWFMKPTPPANI
str.JK68        DSFLLLESGRADAFIMDASILAGHIAKSKNPSDYIILDTVLSVEPIACMLRLNDKNLKQAINDSIVRQIKDESLKKLHDKWFMKPTPPANI
str.JK31        DSFLLLESGRADAFIMDASILAGHIAKSKNPSDYIILDTVLSVEPIACMLRLNDKNLKQAINDSIVRQIKDESLKKLHDKWFMKPTPPANI
str.G1713       DSFLLLESGRADAFIMDASILAGHIAKSKNPSDYIILDTVLSVEPIACMLRLNDKNLKQAINDSIVRQIKDESLKKLHDKWFMKPTPPANI
str.G1712       DSFLLLESGRADAFIMDASILAGHIAKSKNPSDYIILDTVLSVEPIACMLRLNDKNLKQAINDSIVRQIKDESLKKLHDKWFMKPTPPANI
str.JK7         DSFLLLESGRADAFIMDASILAGHIAKSKNPSDYIILDTVLSVEPIACMLRLNDKNLKQAINDSIVRQIKDESLKKLHDKWFMKPTPPANI
str.BQ2-D70     DSFLLLESGRADAFIMDASILAGHIAKSKNPSDYIILDTVLSVEPIACMLRLNDKNLKQAINDSIVRQIKDESLKKLHDKWFMKPTPPANI
str.CO20_0321   DSFLLLESGRADAFIMDASILAGHIAKSKNPSDYIILDTVLSVEPIACMLRLNDKNLKQAINDSIVRQIKDESLKKLHDKWFMKPTPPANI
str.CO21_0024   DSFLLLESGRADAFIMDASILAGHIAKSKNPSDYIILDTVLSVEPIACMLRLNDKNLKQAINDSIVRQIKDESLKKLHDKWFMKPTPPANI
str.CO20_0297   DSFLLLESGRADAFIMDASILAGHIAKSKNPSDYIILDTVLSVEPIACMLRLNDKNLKQAINDSIVRQIKDESLKKLHDKWFMKPTPPANI
str.CO20_0256   DSFLLLESGRADAFIMDASILAGHIAKSKNPSDYIILDTVLSVEPIACMLRLNDKNLKQAINDSIVRQIKDESLKKLHDKWFMKPTPPANI
str.CO20_0257   DSFLLLESGRADAFIMDASILAGHIAKSKNPSDYIILDTVLSVEPIACMLRLNDKNLKQAINDSIVRQIKDESLKKLHDKWFMKPTPPANI
str.NCTC12899   DSFLLLESGRADAFIMDASILAGHIAKSKNPSDYIILDTVLSVEPIACMLRLNDKNLKQAINDSIVRQIKDESLKKLHDKWFMKPTPPANI
str.JK73rel     DSFLLLESGRADAFIMDASILAGHIAKSKNPSDYIILDTVLSVEPIACMLRLNDKNLKQAINDSIVRQIKDESLKRLHDKWFMKPTPPANI
str.JK73        DSFLLLESGRADAFIMDASILAGHIAKSKNPSDYIILDTVLSVEPIACMLRLNDKNLKQAINDSIVRQIKDESLKRLHDKWFMKPTPPANI
str.MF1-1       DSFLLLESGRADAFIMDASILAGHIAKSKNPSDYIILDTVLSVEPIACMLRLNDKNLKQAINDSIVRQIKDESLKKLHDKWFMKPTPPANI
str.RM-11       DSFLLLESGRADAFIMDASILAGHIAKSKNPSDYIILDTVLSVEPIACMLRLNDKNLKQAINDSIVRQIKDESLKKLHDKWFMKPTPPANI
```

```
                       280       290       300
str.Toulouse    IINLPLSEATKYAWEHPNDKPRENYTENNL
str.JK12        IINLPLSEATKYAWEHPNDKPRENYTENNL
str.CCUG45777   IINLPLSEATKYAWEHPNDKPRENYTENNL
str.JK56        IINLPLSEATKYAWEHPNDKPRENYTENNL
str.JK67        IINLPLSEATKYAWEHPNDKPRENYTENNL
str.JK19        IINLPLSEATKYAWEHPNDKPRENYTENNL
str.JK63        IINLPLSEATKYAWEHPNDKPRENYTENNL
str.JK39        IINLPLSEATKYAWEHPNDKPRENYTENNL
str.JK68        IINLPLSEATKYAWEHPNDKPRENYTENNL
str.JK31        IINLPLSEATKYAWEHPNDKPRENYTENNL
str.G1713       IINLPLSEATKYAWEHPNDKPRENYTENNL
str.G1712       IINLPLSEATKYAWEHPNDKPRENYTENNL
str.JK7         IINLPLSEATKYAWEHPNDKPRENYTENNL
str.BQ2-D70     IINLPLSEATKYAWEHPNDKPRENYTENNL
str.CO20_0321   IINLPLSEATKYAWEHPNDKPRENYTENNL
str.CO21_0024   IINLPLSEATKYAWEHPNDKPRENYTENNL
str.CO20_0297   IINLPLSEATKYAWEHPNDKPRENYTENNL
str.CO20_0256   IINLPLSEATKYAWEHPNDKPRENYTENNL
str.CO20_0257   IINLPLSEATKYAWEHPNDKPRENYTENNL
str.NCTC12899   IINLPLSEATKYAWEHPNDKPRENYTENNL
str.JK73rel     IINLPLSEATKYAWEHPNDKPRENYTENNL
str.JK73        IINLPLSEATKYAWEHPNDKPRENYTENNL
str.MF1-1       IINLPLSEATKYAWEHPNDKPRENYTENNL
str.RM-11       IINLPLSEATKYAWEHPNDKPRENYTENNL
```

>WP_011179596.1 UDP-3-O-acyl-N-acetylglucosamine deacetylase [Bartonella quintana str. Toulouse]

```
               1         10        20        30        40        50        60        70        80        90
str.Toulouse   MKPAQKYQSTLKKAVTFKGYGVHSGCLSVVKVCPADVGCGIVFKRFGMDGTEQIFQAHASQTGATELSTGLGYGDVRIETIEHLMAAIVAY
str.JK12       MKPAQKYQSTLKKAVTFKGYGVHSGCLSVVKVCPADVGCGIVFKRFGMDGTEQIFQAHASQTGATELSTGLGYGDVRIETIEHLMAAIVAY
str.CCUG45777  MKPAQKYQSTLKKAVTFKGYGVHSGCLSVVKVCPADVGCGIVFKRFGMDGTEQIFQAHASQTGATELSTGLGYGDVRIETIEHLMAAIVAY
str.JK56       MKPAQKYQSTLKKAVTFKGYGVHSGCLSVVKVCPADVGCGIVFKRFGMDGTEQIFQAHASQTGATELSTGLGYGDVRIETIEHLMAAIVAY
str.JK67       MKPAQKYQSTLKKAVTFKGYGVHSGCLSVVKVCPADVGCGIVFKRFGMDGTEQIFQAHASQTGATELSTGLGYGDVRIETIEHLMAAIVAY
str.JK63       MKPAQKYQSTLKKAVTFKGYGVHSGCLSVVKVCPADVGCGIVFKRFGMDGTEQIFQAHASQTGATELSTGLGYGDVRIETIEHLMAAIVAY
str.JK19       MKPAQKYQSTLKKAVTFKGYGVHSGCLSVVKVCPADVGCGIVFKRFGMDGTEQIFQAHASQTGATELSTGLGYGDVRIETIEHLMAAIVAY
str.JK39       MKPAQKYQSTLKKAVTFKGYGVHSGCLSVVKVCPADVGCGIVFKRFGMDGTEQIFQAHASQTGATELSTGLGYGDVRIETIEHLMAAIVAY
str.JK68       MKPAQKYQSTLKKAVTFKGYGVHSGCLSVVKVCPADVGCGIVFKRFGMDGTEQIFQAHASQTGATELSTGLGYGDVRIETIEHLMAAIVAY
str.JK31       MKPAQKYQSTLKKAVTFKGYGVHSGCLSVVKVCPADVGCGIVFKRFGMDGTEQIFQAHASQTGATELSTGLGYGDVRIETIEHLMAAIVAY
str.G1713      MKPAQKYQSTLKKAVTFKGYGVHSGCLSVVKVCPADVGCGIVFKRFGMDGTEQIFQAHASQTGATELSTGLGYGDVRIETIEHLMAAIVAY
str.G1712      MKPAQKYQSTLKKAVTFKGYGVHSGCLSVVKVCPADVGCGIVFKRFGMDGTEQIFQAHASQTGATELSTGLGYGDVRIETIEHLMAAIVAY
str.JK73       MKPAQKYQSTLKKAVTFKGYGVHSGCLSVVKVCPADVGCGIVFKRFGMDGTEQIFQAHASQTGATELSTGLGYGDVRIETIEHLMAAIVAY
str.JK7        MKPAQKYQSTLKKAVTFKGYGVHSGCLSVVKVCPADVGCGIVFKRFGMDGTEQIFQAHASQTGATELSTGLGYGDVRIETIEHLMAAIVAY
str.JK73rel    MKPAQKYQSTLKKAVTFKGYGVHSGCLSVVKVCPADVGCGIVFKRFGMDGTEQIFQAHASQTGATELSTGLGYGDVRIETIEHLMAAIVAY
str.BQ2-D70    MKPAQKYQSTLKKAVTFKGYGVHSGCLSVVKVCPADVGCGIVFKRFGMDGTEQIFQAHASQTGATELSTGLGYGDVRIETIEHLMAAIVAY
str.CO20_0321  MKPAQKYQSTLKKAVTFKGYGVHSGCLSVVKVCPADVGCGIVFKRFGMDGTEQIFQAHASQTGATELSTGLGYGDVRIETIEHLMAAIVAY
str.CO21_0024  MKPAQKYQSTLKKAVTFKGYGVHSGCLSVVKVCPADVGCGIVFKRFGMDGTEQIFQAHASQTGATELSTGLGYGDVRIETIEHLMAAIVAY
str.CO20_0297  MKPAQKYQSTLKKAVTFKGYGVHSGCLSVVKVCPADVGCGIVFKRFGMDGTEQIFQAHASQTGATELSTGLGYGDVRIETIEHLMAAIVAY
str.CO20_0256  MKPAQKYQSTLKKAVTFKGYGVHSGCLSVVKVCPADVGCGIVFKRFGMDGTEQIFQAHASQTGATELSTGLGYGDVRIETIEHLMAAIVAY
str.CO20_0257  MKPAQKYQSTLKKAVTFKGYGVHSGCLSVVKVCPADVGCGIVFKRFGMDGTEQIFQAHASQTGATELSTGLGYGDVRIETIEHLMAAIVAY
str.NCTC12899  MKPAQKYQSTLKKAVTFKGYGVHSGCLSVVKVCPADVGCGIVFKRFGMDGTEQIFQAHASQTGATELSTGLGYGDVRIETIEHLMAAIVAY
str.MF1-1      MKLAQKYQSTLKKAVTFKGYGVHSGCLSVVKVCPADVGCGIVFKRFGMDGTEQIFQAHASQTGATELSTGLGYGDVRIETIEHLMAAIVAY
str.RM-11      MKLAQKYQSTLKKAVTFKGYGVHSGCLSVVKVCPADVGCGIVFKRFGLDGTEQIFQAHASQTGATELSTGLGYGDVRIETIEHLMAAIVAY

                       100       110       120       130       140       150       160       170       180
str.Toulouse   NLDNLVIEVSSHEIPILDGSSWQYCQGFEEVGIVQQNALRSYFIIKKPLRVEGTGGVAEFLPLEGRCFDITISFPSSVIGKQHLNFDLTTQ
str.JK12       NLDNLVIEVSSHEIPILDGSSWQYCQGFEEVGIVQQNALRSYFIIKKPLRVEGTGGVAEFLPLEGRCFDITISFPSSVIGKQHLNFDLTTQ
str.CCUG45777  NLDNLVIEVSSHEIPILDGSSWQYCQGFEEVGIVQQNALRSYFIIKKPLRVEGTGGVAEFLPLEGRCFDITISFPSSVIGKQHLNFDLTTQ
str.JK56       NLDNLVIEVSSHEIPILDGSSWQYCQGFEEVGIVQQNALRSYFIIKKPLRVEGTGGVAEFLPLEGRCFDITISFPSSVIGKQHLNFDLTTQ
str.JK67       NLDNLVIEVSSHEIPILDGSSWQYCQGFEEVGIVQQNALRSYFIIKKPLRVEGTGGVAEFLPLEGRCFDITISFPSSVIGKQHLNFDLTTQ
str.JK63       NLDNLVIEVSSHEIPILDGSSWQYCQGFEEVGIVQQNALRSYFIIKKPLRVEGTGGVAEFLPLEGRCFDITISFPSSVIGKQHLNFDLTTQ
str.JK19       NLDNLVIEVSSHEIPILDGSSWQYCQGFEEVGIVQQNALRSYFIIKKPLRVEGTGGVAEFLPLEGRCFDITISFPSSVIGKQHLNFDLTTQ
str.JK39       NLDNLVIEVSSHEIPILDGSSWQYCQGFEEVGIVQQNALRSYFIIKKPLRVEGTGGVAEFLPLEGRCFDITISFPSSVIGKQHLNFDLTTQ
str.JK68       NLDNLVIEVSSHEIPILDGSSWQYCQGFEEVGIVQQNALRSYFIIKKPLRVEGTGGVAEFLPLEGRCFDITISFPSSVIGKQHLNFDLTTQ
str.JK31       NLDNLVIEVSSHEIPILDGSSWQYCQGFEEVGIVQQNALRSYFIIKKPLRVEGTGGVAEFLPLEGRCFDITISFPSSVIGKQHLNFDLTTQ
str.G1713      NLDNLVIEVSSHEIPILDGSSWQYCQGFEEVGIVQQNALRSYFIIKKPLRVEGTGGVAEFLPLEGRCFDITISFPSSVIGKQHLNFDLTTQ
str.G1712      NLDNLVIEVSSHEIPILDGSSWQYCQGFEEVGIVQQNALRSYFIIKKPLRVEGTGGVAEFLPLEGRCFDITISFPSSVIGKQHLNFDLTTQ
str.JK73       NLDNLVIEVSSHEIPILDGSSWQYCQGFEEVGIVQQNALRSYFIIKKPLRVEGTGGVAEFLPLEGRCFDITISFPSSVIGKQHLNFDLTTQ
str.JK7        NLDNLVIEVSSHEIPILDGSSWQYCQGFEEVGIVQQNALRSYFIIKKPLRVEGTGGVAEFLPLEGRCFDITISFPSSVIGKQHLNFDLTTQ
str.JK73rel    NLDNLVIEVSSHEIPILDGSSWQYCQGFEEVGIVQQNALRSYFIIKKPLRVEGTGGVAEFLPLEGRCFDITISFPSSVIGKQHLNFDLTTQ
str.BQ2-D70    NLDNLVIEVSSHEIPILDGSSWQYCQGFEEVGIVQQNALRSYFIIKKPLRVEGTGGVAEFLPLEGRCFDITISFPSSVIGKQHLNFDLTTQ
str.CO20_0321  NLDNLVIEVSSHEIPILDGSSWQYCQGFEEVGIVQQNALRSYFIIKKPLRVEGTGGVAEFLPLEGRCFDITISFPSSVIGKQHLNFDLTTQ
str.CO21_0024  NLDNLVIEVSSHEIPILDGSSWQYCQGFEEVGIVQQNALRSYFIIKKPLRVEGTGGVAEFLPLEGRCFDITISFPSSVIGKQHLNFDLTTQ
str.CO20_0297  NLDNLVIEVSSHEIPILDGSSWQYCQGFEEVGIVQQNALRSYFIIKKPLRVEGTGGVAEFLPLEGRCFDITISFPSSVIGKQHLNFDLTTQ
str.CO20_0256  NLDNLVIEVSSHEIPILDGSSWQYCQGFEEVGIVQQNALRSYFIIKKPLRVEGTGGVAEFLPLEGRCFDITISFPSSVIGKQHLNFDLTTQ
str.CO20_0257  NLDNLVIEVSSHEIPILDGSSWQYCQGFEEVGIVQQNALRSYFIIKKPLRVEGTGGVAEFLPLEGRCFDITISFPSSVIGKQHLNFDLTTQ
str.NCTC12899  NLDNLVIEVSSHEIPILDGSSWQYCQGFEEVGIVQQNALRSYFIIKKPLRVEGTGGVAEFLPLEGRCFDITISFPSSVIGKQHLNFDLTTQ
str.MF1-1      NLDNLVIEVSSHEIPILDGSSWQYCQGFEEVGIVQQNALRSYFIIKKPLRVEGTGGVAEFLPLEGRCFDITISFPSSAIGKQHLNFDLTTQ
str.RM-11      NLDNLVIEVSSHEIPILDGSSWQYCQGFEEVGIVQQNALRSYFIIKKPLRVEGTGGVAEFLPLEGRCFDITISFPSSAIGKQHLNFDLTTQ

                     190       200       210       220       230       240       250       260       270
str.Toulouse   GFRDDLSRARTFGFVKDVEKLWVSGKGIGASLENCLIIGLNDKIMNPEGPYWKNECVRHKMLDAIGDTALLGAPFIGLFRSYCSGHRINSQ
str.JK12       GFRDDLSRARTFGFVKDVEKLWVSGKGIGASLENCLIIGLNDKIMNPEGPYWKNECVRHKMLDAIGDTALLGAPFIGLFRSYCSGHRINSQ
str.CCUG45777  GFRDDLSRARTFGFVKDVEKLWVSGKGIGASLENCLIIGLNDKIMNPEGPYWKNECVRHKMLDAIGDTALLGAPFIGLFRSYCSGHRINSQ
str.JK56       GFRDDLSRARTFGFVKDVEKLWVSGKGIGASLENCLIIGLNDKIMNPEGPYWKNECVRHKMLDAIGDTALLGAPFIGLFRSYCSGHRINSQ
str.JK67       GFRDDLSRARTFGFVKDVEKLWVSGKGIGASLENCLIIGLNDKIMNPEGPYWKNECVRHKMLDAIGDTALLGAPFIGLFRSYCSGHRINSQ
str.JK63       GFRDDLSRARTFGFVKDVEKLWVSGKGIGASLENCLIIGLNDKIMNPEGPYWKNECVRHKMLDAIGDTALLGAPFIGLFRSYCSGHRINSQ
str.JK19       GFRDDLSRARTFGFVKDVEKLWVSGKGIGASLENCLIIGLNDKIMNPEGPYWKNECVRHKMLDAIGDTALLGAPFIGLFRSYCSGHRINSQ
str.JK39       GFRDDLSRARTFGFVKDVEKLWVSGKGIGASLENCLIIGLNDKIMNPEGPYWKNECVRHKMLDAIGDTALLGAPFIGLFRSYCSGHRINSQ
str.JK68       GFRDDLSRARTFGFVKDVEKLWVSGKGIGASLENCLIIGLNDKIMNPEGPYWKNECVRHKMLDAIGDTALLGAPFIGLFRSYCSGHRINSQ
str.JK31       GFRDDLSRARTFGFVKDVEKLWVSGKGIGASLENCLIIGLNDKIMNPEGPYWKNECVRHKMLDAIGDTALLGAPFIGLFRSYCSGHRINSQ
str.G1713      GFRDDLSRARTFGFVKDVEKLWVSGKGIGASLENCLIIGLNDKIMNPEGPYWKNECVRHKMLDAIGDTALLGAPFIGLFRSYCSGHRINSQ
str.G1712      GFRDDLSRARTFGFVKDVEKLWVSGKGIGASLENCLIIGLNDKIMNPEGPYWKNECVRHKMLDAIGDTALLGAPFIGLFRSYCSGHRINSQ
str.JK73       GFRDDLSRARTFGFVKDVEKLWVSGKGIGASLENCLIIGLNDKIMNPEGPYWKNECVRHKMLDAIGDTALLGAPFIGLFRSYCSGHRINSQ
str.JK7        GFRDDLSRARTFGFVKDVEKLWVSGKGIGASLENCLIIGLNDKIMNPEGPYWKNECVRHKMLDAIGDTALLGAPFIGLFRSYCSGHRINSQ
str.JK73rel    GFRDDLSRARTFGFVKDVEKLWVSGKGIGASLENCLIIGLNDKIMNPEGPYWKNECVRHKMLDAIGDTALLGAPFIGLFRSYCSGHRINSQ
str.BQ2-D70    GFRDDLSRARTFGFVKDVEKLWVSGKGIGASLENCLIIGLNDKIMNPEGPYWKNECVRHKMLDAIGDTALLGAPFIGLFRSYCSGHRINSQ
str.CO20_0321  GFRDDLSRARTFGFVKDVEKLWVSGKGIGASLENCLIIGLNDKIMNPEGPYWKNECVRHKMLDAIGDTALLGAPFIGLFRSYCSGHRINSQ
str.CO21_0024  GFRDDLSRARTFGFVKDVEKLWVSGKGIGASLENCLIIGLNDKIMNPEGPYWKNECVRHKMLDAIGDTALLGAPFIGLFRSYCSGHRINSQ
str.CO20_0297  GFRDDLSRARTFGFVKDVEKLWVSGKGIGASLENCLIIGLNDKIMNPEGPYWKNECVRHKMLDAIGDTALLGAPFIGLFRSYCSGHRINSQ
str.CO20_0256  GFRDDLSRARTFGFVKDVEKLWVSGKGIGASLENCLIIGLNDKIMNPEGPYWKNECVRHKMLDAIGDTALLGAPFIGLFRSYCSGHRINSQ
str.CO20_0257  GFRDDLSRARTFGFVKDVEKLWVSGKGIGASLENCLIIGLNDKIMNPEGPYWKNECVRHKMLDAIGDTALLGAPFIGLFRSYCSGHRINSQ
str.NCTC12899  GFRDDLSRARTFGFVKDVEKLWVSGKGIGASLENCLIIGLNDKIMNPEGPYWKNECVRHKMLDAIGDTALLGAPFIGLFRSYCSGHRINSQ
str.MF1-1      GFRDDLSRARTFGFVKDVEKLWVSGKGIGASLENCLIIGLNDKIMNPEGPYWKNECVRHKMLDAIGDTALLGAPFIGLFRSYCSGHRINSQ
str.RM-11      GFRDDLSRARTFGFVKDVEKLWVSGKGIGASLENCLIIGLNDKIMNPEGPYWKNECVRHKMLDAIGDTALLGAPFIGLFRSYCSGHRINSQ

                    280       290
str.Toulouse   LVKAVLADESCYEKSHL
str.JK12       LVKAVLADESCYEKSHL
str.CCUG45777  LVKAVLADESCYEKSHL
str.JK56       LVKAVLADESCYEKSHL
str.JK67       LVKAVLADESCYEKSHL
str.JK63       LVKAVLADESCYEKSHL
str.JK19       LVKAVLADESCYEKSHL
str.JK39       LVKAVLADESCYEKSHL
str.JK68       LVKAVLADESCYEKSHL
str.JK31       LVKAVLADESCYEKSHL
str.G1713      LVKAVLADESCYEKSHL
str.G1712      LVKAVLADESCYEKSHL
str.JK73       LVKAVLADESCYEKSHL
str.JK7        LVKAVLADESCYEKSHL
str.JK73rel    LVKAVLADESCYEKSHL
str.BQ2-D70    LVKAVLADESCYEKSHL
str.CO20_0321  LVKAVLADESCYEKSHL
str.CO21_0024  LVKAVLADESCYEKSHL
str.CO20_0297  LVKAVLADESCYEKSHL
str.CO20_0256  LVKAVLADESCYEKSHL
str.CO20_0257  LVKAVLADESCYEKSHL
str.NCTC12899  LVKAVLADESCYEKSHL
str.MF1-1      LVKAVLADESCYEKSHL
str.RM-11      LVKAVLADESCYEKSHL
```

>WP_011179600.1 D-alanine - D-alanine ligase [Bartonella quintana str. Toulouse]

```
               1        10        20        30        40        50        60        70        80        90
str.Toulouse   MKGEHVAVLMGGGSSERSVSLSSGTACADILEARGYRVSRVDVDSHIASVLERLQPDVAFNALHGPFGEDGCIQGVLEYLKIPYTHSGVMA
str.JK73rel    MKGEHVAVLMGGGSSERSVSLSSGTACADILEARGYRVSRVDVDSHIASVLERLQPDVAFNALHGPFGEDGCIQGVLEYLKIPYTHSGVMA
str.JK73       MKGEHVAVLMGGGSSERSVSLSSGTACADILEARGYRVSRVDVDSHIASVLERLQPDVAFNALHGPFGEDGCIQGVLEYLKIPYTHSGVMA
str.NCTC12899  MKGEHVAVLMGGGSSERSVSLSSGTACADILEARGYRVSRVDVDSHIASVLERLQPDVAFNALHGPFGEDGCIQGVLEYLKIPYTHSGVMA
str.CO20_0257  MKGEHVAVLMGGGSSERSVSLSSGTACADILEARGYRVSRVDVDSHIASVLERLQPDVAFNALHGPFGEDGCIQGVLEYLKIPYTHSGVMA
str.CO20_0256  MKGEHVAVLMGGGSSERSVSLSSGTACADILEARGYRVSRVDVDSHIASVLERLQPDVAFNALHGPFGEDGCIQGVLEYLKIPYTHSGVMA
str.CO20_0297  MKGEHVAVLMGGGSSERSVSLSSGTACADILEARGYRVSRVDVDSHIASVLERLQPDVAFNALHGPFGEDGCIQGVLEYLKIPYTHSGVMA
str.CO21_0024  MKGEHVAVLMGGGSSERSVSLSSGTACADILEARGYRVSRVDVDSHIASVLERLQPDVAFNALHGPFGEDGCIQGVLEYLKIPYTHSGVMA
str.CO20_0321  MKGEHVAVLMGGGSSERSVSLSSGTACADILEARGYRVSRVDVDSHIASVLERLQPDVAFNALHGPFGEDGCIQGVLEYLKIPYTHSGVMA
str.BQ2-D70    MKGEHVAVLMGGGSSERSVSLSSGTACADILEARGYRVSRVDVDSHIASVLERLQPDVAFNALHGPFGEDGCIQGVLEYLKIPYTHSGVMA
str.JK7        MKGEHVAVLMGGGSSERSVSLSSGTACADILEARGYRVSRVDVDSHIASVLERLQPDVAFNALHGPFGEDGCIQGVLEYLKIPYTHSGVMA
str.G1712      MKGEHVAVLMGGGSSERSVSLSSGTACADILEARGYRVSRVDVDSHIASVLERLQPDVAFNALHGPFGEDGCIQGVLEYLKIPYTHSGVMA
str.JK12       MKGEHVAVLMGGGSSERSVSLSSGTACADILEARGYRVSRVDVDSHIASVLERLQPDVAFNALHGPFGEDGCIQGVLEYLKIPYTHSGVMA
str.CCUG45777  MKGEHVAVLMGGGSSERSVSLSSGTACADILEARGYRVSRVDVDSHIASVLERLQPDVAFNALHGPFGEDGCIQGVLEYLKIPYTHSGVMA
str.JK56       MKGEHVAVLMGGGSSERSVSLSSGTACADILEARGYRVSRVDVDSHIASVLERLQPDVAFNALHGPFGEDGCIQGVLEYLKIPYTHSGVMA
str.JK67       MKGEHVAVLMGGGSSERSVSLSSGTACADILEARGYRVSRVDVDSHIASVLERLQPDVAFNALHGPFGEDGCIQGVLEYLKIPYTHSGVMA
str.JK19       MKGEHVAVLMGGGSSERSVSLSSGTACADILEARGYRVSRVDVDSHIASVLERLQPDVAFNALHGPFGEDGCIQGVLEYLKIPYTHSGVMA
str.JK63       MKGEHVAVLMGGGSSERSVSLSSGTACADILEARGYRVSRVDVDSHIASVLERLQPDVAFNALHGPFGEDGCIQGVLEYLKIPYTHSGVMA
str.JK39       MKGEHVAVLMGGGSSERSVSLSSGTACADILEARGYRVSRVDVDSHIASVLERLQPDVAFNALHGPFGEDGCIQGVLEYLKIPYTHSGVMA
str.JK68       MKGEHVAVLMGGGSSERSVSLSSGTACADILEARGYRVSRVDVDSHIASVLERLQPDVAFNALHGPFGEDGCIQGVLEYLKIPYTHSGVMA
str.JK31       MKGEHVAVLMGGGSSERSVSLSSGTACADILEARGYRVSRVDVDSHIASVLERLQPDVAFNALHGPFGEDGCIQGVLEYLKIPYTHSGVMA
str.G1713      MKGEHVAVLMGGGSSERSVSLSSGTACADILEARGYRVSRVDVDSHIASVLERLQPDVAFNALHGPFGEDGCIQGVLEYLKIPYTHSGVMA
str.RM-11      MKGEHVAVLMGGGSSERSVSLSSGTACADILEERGYRVSRVDVDSHIASVLERLQPDVAFNALHGPFGEDGCIQGVLEYLKIPYTHSGVMA
str.MF1-1      MKGEHVAVLMGGSSSERSVSLSSGTACADILEERGYRVSRVDVDSHIASVLERLQPDVAFNALHGPFGEDGCIQGVLEYLKIPYTHSGVMA

                       100       110       120       130       140       150       160       170       180
str.Toulouse   SALAMDKGRAKIVVASVGVSVAPSCIMSRFAVGREHPMEPPYVIKPLCEGSSFGVVIVKENESAPPRNVVGAEWVYADEVIVEKYIPGREF
str.JK73rel    SALAMDKGRAKIVVASVGVSVAPSCIMSRFAVGREHPMEPPYVIKPLCEGSSFGVVIVKENESAPPRNVVGAEWVYADEVIVEKYIPGREF
str.JK73       SALAMDKGRAKIVVASVGVSVAPSCIMSRFAVGREHPMEPPYVIKPLCEGSSFGVVIVKENESAPPRNVVGAEWVYADEVIVEKYIPGREF
str.NCTC12899  SALAMDKGRAKIVVASVGVSVAPSCIMSRFAVGREHPMEPPYVIKPLCEGSSFGVVIVKENESAPPRNVVGAEWVYADEVIVEKYIPGREF
str.CO20_0257  SALAMDKGRAKIVVASVGVSVAPSCIMSRFAVGREHPMEPPYVIKPLCEGSSFGVVIVKENESAPPRNVVGAEWVYADEVIVEKYIPGREF
str.CO20_0256  SALAMDKGRAKIVVASVGVSVAPSCIMSRFAVGREHPMEPPYVIKPLCEGSSFGVVIVKENESAPPRNVVGAEWVYADEVIVEKYIPGREF
str.CO20_0297  SALAMDKGRAKIVVASVGVSVAPSCIMSRFAVGREHPMEPPYVIKPLCEGSSFGVVIVKENESAPPRNVVGAEWVYADEVIVEKYIPGREF
str.CO21_0024  SALAMDKGRAKIVVASVGVSVAPSCIMSRFAVGREHPMEPPYVIKPLCEGSSFGVVIVKENESAPPRNVVGAEWVYADEVIVEKYIPGREF
str.CO20_0321  SALAMDKGRAKIVVASVGVSVAPSCIMSRFAVGREHPMEPPYVIKPLCEGSSFGVVIVKENESAPPRNVVGAEWVYADEVIVEKYIPGREF
str.BQ2-D70    SALAMDKGRAKIVVASVGVSVAPSCIMSRFAVGREHPMEPPYVIKPLCEGSSFGVVIVKENESAPPRNVVGAEWVYADEVIVEKYIPGREF
str.JK7        SALAMDKGRAKIVVASVGVSVAPSCIMSRFAVGREHPMEPPYVIKPLCEGSSFGVVIVKENESAPPRNVVGAEWVYADEVIVEKYIPGREF
str.G1712      SALAMDKGRAKIVVASVGVSVAPSCIMSRFAVGREHPMEPPYVIKPLCEGSSFGVVIVKENESAPPRNVVGAEWVYADEVIVEKYIPGREF
str.JK12       SALAMDKGRAKIVVASVGVSVAPSCIMSRFAVGREHPMEPPYVIKPLCEGSSFGVVIVKENESAPPRNVVGAEWVYADEVIVEKYIPGREF
str.CCUG45777  SALAMDKGRAKIVVASVGVSVAPSCIMSRFAVGREHPMEPPYVIKPLCEGSSFGVVIVKENESAPPRNVVGAEWVYADEVIVEKYIPGREF
str.JK56       SALAMDKGRAKIVVASVGVSVAPSCIMSRFAVGREHPMEPPYVIKPLCEGSSFGVVIVKENESAPPRNVVGAEWVYADEVIVEKYIPGREF
str.JK67       SALAMDKGRAKIVVASVGVSVAPSCIMSRFAVGREHPMEPPYVIKPLCEGSSFGVVIVKENESAPPRNVVGAEWVYADEVIVEKYIPGREF
str.JK19       SALAMDKGRAKIVVASVGVSVAPSCIMSRFAVGREHPMEPPYVIKPLCEGSSFGVVIVKENESAPPRNVVGAEWVYADEVIVEKYIPGREF
str.JK63       SALAMDKGRAKIVVASVGVSVAPSCIMSRFAVGREHPMEPPYVIKPLCEGSSFGVVIVKENESAPPRNVVGAEWVYADEVIVEKYIPGREF
str.JK39       SALAMDKGRAKIVVASVGVSVAPSCIMSRFAVGREHPMEPPYVIKPLCEGSSFGVVIVKENESAPPRNVVGAEWVYADEVIVEKYIPGREF
str.JK68       SALAMDKGRAKIVVASVGVSVAPSCIMSRFAVGREHPMEPPYVIKPLCEGSSFGVVIVKENESAPPRNVVGAEWVYADEVIVEKYIPGREF
str.JK31       SALAMDKGRAKIVVASVGVSVAPSCIMSRFAVGREHPMEPPYVIKPLCEGSSFGVVIVKENESAPPRNVVGAEWVYADEVIVEKYIPGREF
str.G1713      SALAMDKGRAKIVVASVGVSVAPSCIMSRFAVGREHPMEPPYVIKPLCEGSSFGVVIVKENESAPPRNVVGAEWVYADEVIVEKYIPGREF
str.RM-11      SALAMDKGRAKIVVASVGVSVAPSCIMSRFAVGREHPMEPPYVIKPLCEGSSFGVVIVKENESAPPRNVVGAEWVYADEIIVEKYIPGREF
str.MF1-1      SALAMDKGRAKIVVASVGVSVAPSCIMSRFAVGREHPMEPPYVIKPLCEGSSFGVVIVKENESAPPRNVVGAEWVYADEIIVEKYIPGREF

                      190       200       210       220       230       240       250       260       270
str.Toulouse   TCAVLGNEALDVCEIFPDKHFQFYNYDSKYKSGGSLHICPAQLSPNIYQNVQRMSLAAHQAIGCRGVSRSDFRFNEETGELVWLEINTQPG
str.JK73rel    TCAVLGNEALDVCEIFPDKHFQFYNYDSKYRSGGSLHICPAQLSPNIYQNVQRMSLAAHQAIGCRGVSRSDFRFNEETGELVWLEINTQPG
str.JK73       TCAVLGNEALDVCEIFPDKHFQFYNYDSKYRSGGSLHICPAQLSPNIYQNVQRMSLAAHQAIGCRGVSRSDFRFNEETGELVWLEINTQPG
str.NCTC12899  TCAVLGNEALDVCEIFPDKHFQFYNYDSKYKSGGSLHICPAQLSPNIYQNVQRMSLAAHQAIGCRGVSRSDFRFNEETGELVWLEINTQPG
str.CO20_0257  TCAVLGNEALDVCEIFPDKHFQFYNYDSKYKSGGSLHICPAQLSPNIYQNVQRMSLAAHQAIGCRGVSRSDFRFNEETGELVWLEINTQPG
str.CO20_0256  TCAVLGNEALDVCEIFPDKHFQFYNYDSKYKSGGSLHICPAQLSPNIYQNVQRMSLAAHQAIGCRGVSRSDFRFNEETGELVWLEINTQPG
str.CO20_0297  TCAVLGNEALDVCEIFPDKHFQFYNYDSKYKSGGSLHICPAQLSPNIYQNVQRMSLAAHQAIGCRGVSRSDFRFNEETGELVWLEINTQPG
str.CO21_0024  TCAVLGNEALDVCEIFPDKHFQFYNYDSKYKSGGSLHICPAQLSPNIYQNVQRMSLAAHQAIGCRGVSRSDFRFNEETGELVWLEINTQPG
str.CO20_0321  TCAVLGNEALDVCEIFPDKHFQFYNYDSKYKSGGSLHICPAQLSPNIYQNVQRMSLAAHQAIGCRGVSRSDFRFNEETGELVWLEINTQPG
str.BQ2-D70    TCAVLGNEALDVCEIFPDKHFQFYNYDSKYKSGGSLHICPAQLSPNIYQNVQRMSLAAHQAIGCRGVSRSDFRFNEETGELVWLEINTQPG
str.JK7        TCAVLGNEALDVCEIFPDKHFQFYNYDSKYKSGGSLHICPAQLSPNIYQNVQRMSLAAHQAIGCRGVSRSDFRFNEETGELVWLEINTQPG
str.G1712      TCAVLGNEALDVCEIFPDKHFQFYNYDSKYKSGGSLHICPAQLSPNIYQNVQRMSLAAHQAIGCRGVSRSDFRFNEETGELVWLEINTQPG
str.JK12       TCAVLGNEALDVCEIFPDKHFQFYNYDSKYKSGGSLHICPAQLSPNIYQNVQRMSLAAHQAIGCRGVSRSDFRFNEETGELVWLEINTQPG
str.CCUG45777  TCAVLGNEALDVCEIFPDKHFQFYNYDSKYKSGGSLHICPAQLSPNIYQNVQRMSLAAHQAIGCRGVSRSDFRFNEETGELVWLEINTQPG
str.JK56       TCAVLGNEALDVCEIFPDKHFQFYNYDSKYKSGGSLHICPAQLSPNIYQNVQRMSLAAHQAIGCRGVSRSDFRFNEETGELVWLEINTQPG
str.JK67       TCAVLGNEALDVCEIFPDKHFQFYNYDSKYKSGGSLHICPAQLSPNIYQNVQRMSLAAHQAIGCRGVSRSDFRFNEETGELVWLEINTQPG
str.JK19       TCAVLGNEALDVCEIFPDKHFQFYNYDSKYKSGGSLHICPAQLSPNIYQNVQRMSLAAHQAIGCRGVSRSDFRFNEETGELVWLEINTQPG
str.JK63       TCAVLGNEALDVCEIFPDKHFQFYNYDSKYKSGGSLHICPAQLSPNIYQNVQRMSLAAHQAIGCRGVSRSDFRFNEETGELVWLEINTQPG
str.JK39       TCAVLGNEALDVCEIFPDKHFQFYNYDSKYKSGGSLHICPAQLSPNIYQNVQRMSLAAHQAIGCRGVSRSDFRFNEETGELVWLEINTQPG
str.JK68       TCAVLGNEALDVCEIFPDKHFQFYNYDSKYKSGGSLHICPAQLSPNIYQNVQRMSLAAHQAIGCRGVSRSDFRFNEETGELVWLEINTQPG
str.JK31       TCAVLGNEALDVCEIFPDKHFQFYNYDSKYKSGGSLHICPAQLSPNIYQNVQRMSLAAHQAIGCRGVSRSDFRFNEETGELVWLEINTQPG
str.G1713      TCAVLGNEALDVCEIFPDKHFQFYNYDSKYKSGGSLHICPAQLSPNIYQNVQRMSLAAHQAIGCRGVSRSDFRFNEETGELVWLEINTQPG
str.RM-11      TCAVLGNEALDVCEIFPDKHFQFYNYDSKYKSGGSLHICPAQLSPNIYQNVQRMSLAAHQAIGCRGVSRSDFRFNEETGELVWLEINTQPG
str.MF1-1      TCAVLGNEALDVCEIFPDKHFQFYNYDSKYKSGGSLHICPAQLSPNIYQNVQRMSLAAHQAIGCRGVSRSDFRFNEETGELVWLEINTQPG

                    280       290       300
str.Toulouse   MTPTSLFPDIAKASGRTYGDIVQWMVEDASCMR
str.JK73rel    MTPTSLFPDIAKASGRTYGDIVQWMVEDASCMR
str.JK73       MTPTSLFPDIAKASGRTYGDIVQWMVEDASCMR
str.NCTC12899  MTPTSLFPDIAKASGRTYGDIVQWMVEDASCMR
str.CO20_0257  MTPTSLFPDIAKASGRTYGDIVQWMVEDASCMR
str.CO20_0256  MTPTSLFPDIAKASGRTYGDIVQWMVEDASCMR
str.CO20_0297  MTPTSLFPDIAKASGRTYGDIVQWMVEDASCMR
str.CO21_0024  MTPTSLFPDIAKASGRTYGDIVQWMVEDASCMR
str.CO20_0321  MTPTSLFPDIAKASGRTYGDIVQWMVEDASCMR
str.BQ2-D70    MTPTSLFPDIAKASGRTYGDIVQWMVEDASCMR
str.JK7        MTPTSLFPDIAKASGRTYGDIVQWMVEDASCMR
str.G1712      MTPTSLFPDIAKASGRTYGDIVQWMVEDASCMR
str.JK12       MTPTSLFPDIAKASGRTYGDIVQWMVEDASCMR
str.CCUG45777  MTPTSLFPDIAKASGRTYGDIVQWMVEDASCMR
str.JK56       MTPTSLFPDIAKASGRTYGDIVQWMVEDASCMR
str.JK67       MTPTSLFPDIAKASGRTYGDIVQWMVEDASCMR
str.JK19       MTPTSLFPDIAKASGRTYGDIVQWMVEDASCMR
str.JK63       MTPTSLFPDIAKASGRTYGDIVQWMVEDASCMR
str.JK39       MTPTSLFPDIAKASGRTYGDIVQWMVEDASCMR
str.JK68       MTPTSLFPDIAKASGRTYGDIVQWMVEDASCMR
str.JK31       MTPTSLFPDIAKASGRTYGDIVQWMVEDASCMR
str.G1713      MTPTSLFPDIAKASGRTYGDIVQWMVEDASCMR
str.RM-11      MTPTSLFPDIAKASGRTYGDIVQWMVEDASCMR
str.MF1-1      MTPTSLFPDIAKASGRTYGDIVQWMVEDASCMR
```

>WP_011179602.1 UDP-N-acetylmuramate - L-alanine ligase [Bartonella quintana str. Toulouse]

```
               1        10        20        30        40        50        60        70        80        90
str.Toulouse   MKMPFNIGLIHFVGVGGIGMSGIAEVFHNLGYKVQGSDHVDSANVERLRGKGINIQIGHHAENLGDAEVVVLSTAIKKTNPEYIAAKEKHL
str.JK12       MKMPFNIGLIHFVGVGGIGMSGIAEVFHNLGYKVQGSDHVDSANVERLRGKGINIQIGHHAENLGDAEVVVLSTAIKKTNPEYIAAKEKHL
str.CCUG45777  MKMPFNIGLIHFVGVGGIGMSGIAEVFHNLGYKVQGSDHVDSANVERLRGKGINIQIGHHAENLGDAEVVVLSTAIKKTNPEYIAAKEKHL
str.JK67       MKMPFNIGLIHFVGVGGIGMSGIAEVFHNLGYKVQGSDHVDSANVERLRGKGINIQIGHHAENLGDAEVVVLSTAIKKTNPEYIAAKEKHL
str.JK56       MKMPFNIGLIHFVGVGGIGMSGIAEVFHNLGYKVQGSDHVDSANVERLRGKGINIQIGHHAENLGDAEVVVLSTAIKKTNPEYIAAKEKHL
str.JK19       MKMPFNIGLIHFVGVGGIGMSGIAEVFHNLGYKVQGSDHVDSANVERLRGKGINIQIGHHAENLGDAEVVVLSTAIKKTNPEYIAAKEKHL
str.JK63       MKMPFNIGLIHFVGVGGIGMSGIAEVFHNLGYKVQGSDHVDSANVERLRGKGINIQIGHHAENLGDAEVVVLSTAIKKTNPEYIAAKEKHL
str.JK39       MKMPFNIGLIHFVGVGGIGMSGIAEVFHNLGYKVQGSDHVDSANVERLRGKGINIQIGHHAENLGDAEVVVLSTAIKKTNPEYIAAKEKHL
str.JK68       MKMPFNIGLIHFVGVGGIGMSGIAEVFHNLGYKVQGSDHVDSANVERLRGKGINIQIGHHAENLGDAEVVVLSTAIKKTNPEYIAAKEKHL
str.JK31       MKMPFNIGLIHFVGVGGIGMSGIAEVFHNLGYKVQGSDHVDSANVERLRGKGINIQIGHHAENLGDAEVVVLSTAIKKTNPEYIAAKEKHL
str.G1713      MKMPFNIGLIHFVGVGGIGMSGIAEVFHNLGYKVQGSDHVDSANVERLRGKGINIQIGHHAENLGDAEVVVLSTAIKKTNPEYIAAKEKHL
str.G1712      MKMPFNIGLIHFVGVGGIGMSGIAEVFHNLGYKVQGSDHVDSANVERLRGKGINIQIGHHAENLGDAEVVVLSTAIKKTNPEYIAAKEKHL
str.JK73       MKMPFNIGLIHFVGVGGIGMSGIAEVFHNLGYKVQGSDHVDSANVERLRGKGINIQIGHHAENLGDAEVVVLSTAIKKTNPEYIAAKEKHL
str.JK7        MKMPFNIGLIHFVGVGGIGMSGIAEVFHNLGYKVQGSDHVDSANVERLRGKGINIQIGHHAENLGDAEVVVLSTAIKKTNPEYIAAKEKHL
str.JK73rel    MKMPFNIGLIHFVGVGGIGMSGIAEVFHNLGYKVQGSDHVDSANVERLRGKGINIQIGHHAENLGDAEVVVLSTAIKKTNPEYIAAKEKHL
str.BQ2-D70    MKMPFNIGLIHFVGVGGIGMSGIAEVFHNLGYKVQGSDHVDSANVERLRGKGINIQIGHHAENLGDAEVVVLSTAIKKTNPEYIAAKEKHL
str.CO20_0321  MKMPFNIGLIHFVGVGGIGMSGIAEVFHNLGYKVQGSDHVDSANVERLRGKGINIQIGHHAENLGDAEVVVLSTAIKKTNPEYIAAKEKHL
str.CO21_0024  MKMPFNIGLIHFVGVGGIGMSGIAEVFHNLGYKVQGSDHVDSANVERLRGKGINIQIGHHAENLGDAEVVVLSTAIKKTNPEYIAAKEKHL
str.CO20_0297  MKMPFNIGLIHFVGVGGIGMSGIAEVFHNLGYKVQGSDHVDSANVERLRGKGINIQIGHHAENLGDAEVVVLSTAIKKTNPEYIAAKEKHL
str.CO20_0256  MKMPFNIGLIHFVGVGGIGMSGIAEVFHNLGYKVQGSDHVDSANVERLRGKGINIQIGHHAENLGDAEVVVLSTAIKKTNPEYIAAKEKHL
str.CO20_0257  MKMPFNIGLIHFVGVGGIGMSGIAEVFHNLGYKVQGSDHVDSANVERLRGKGINIQIGHHAENLGDAEVVVLSTAIKKTNPEYIAAKEKHL
str.NCTC12899  MKMPFNIGLIHFVGVGGIGMSGIAEVFHNLGYKVQGSDHVDSANVERLRGKGINIQIGHHAENLGDAEVVVLSTAIKKTNPEYIAAKEKHL
str.RM-11      MKMPFNIGLIHFVGVGGIGMSGIAEVLHNLGYKVQGSDHVDSANVERLRGKGINIQIGHHAENLGDAEVVVLSTAIKKTNPEYIAAKEKHL
str.MF1-1      MKMPFNIGLIHFVGVGGIGMSGIAEVLHNLGYKVQGSDHVDSANVERLRGKGINIQIGHHAENLGDAEVVVLSTAIKKTNPEYIAAKEKHL
```

```
                      100       110       120       130       140       150       160       170       180
str.Toulouse   PIVRRAEMLAELMRFRRAIAVGGTHGKTTTTSMIAALLDAGRFDPMVINGGIINAYGTNARMGSGDWMVVEADESDGTFLKLPADIAVVTN
str.JK12       PIVRRAEMLAELMRFRRAIAVGGTHGKTTTTSMIAALLDAGRFDPMVINGGIINAYGTNARMGSGDWMVVEADESDGTFLKLPADIAVVTN
str.CCUG45777  PIVRRAEMLAELMRFRRAIAVGGTHGKTTTTSMIAALLDAGRFDPMVINGGIINAYGTNARMGSGDWMVVEADESDGTFLKLPADIAVVTN
str.JK67       PIVRRAEMLAELMRFRRAIAVGGTHGKTTTTSMIAALLDAGRFDPMVINGGIINAYGTNARMGSGDWMVVEADESDGTFLKLPADIAVVTN
str.JK56       PIVRRAEMLAELMRFRRAIAVGGTHGKTTTTSMIAALLDAGRFDPMVINGGIINAYGTNARMGSGDWMVVEADESDGTFLKLPADIAVVTN
str.JK19       PIVRRAEMLAELMRFRRAIAVGGTHGKTTTTSMIAALLDAGRFDPMVINGGIINAYGTNARMGSGDWMVVEADESDGTFLKLPADIAVVTN
str.JK63       PIVRRAEMLAELMRFRRAIAVGGTHGKTTTTSMIAALLDAGRFDPMVINGGIINAYGTNARMGSGDWMVVEADESDGTFLKLPADIAVVTN
str.JK39       PIVRRAEMLAELMRFRRAIAVGGTHGKTTTTSMIAALLDAGRFDPMVINGGIINAYGTNARMGSGDWMVVEADESDGTFLKLPADIAVVTN
str.JK68       PIVRRAEMLAELMRFRRAIAVGGTHGKTTTTSMIAALLDAGRFDPMVINGGIINAYGTNARMGSGDWMVVEADESDGTFLKLPADIAVVTN
str.JK31       PIVRRAEMLAELMRFRRAIAVGGTHGKTTTTSMIAALLDAGRFDPMVINGGIINAYGTNARMGSGDWMVVEADESDGTFLKLPADIAVVTN
str.G1713      PIVRRAEMLAELMRFRRAIAVGGTHGKTTTTSMIAALLDAGRFDPMVINGGIINAYGTNARMGSGDWMVVEADESDGTFLKLPADIAVVTN
str.G1712      PIVRRAEMLAELMRFRRAIAVGGTHGKTTTTSMIAALLDAGRFDPMVINGGIINAYGTNARMGSGDWMVVEADESDGTFLKLPADIAVVTN
str.JK73       PIVRRAEMLAELMRFRRAIAVGGTHGKTTTTSMIAALLDAGRFDPMVINGGIINAYGTNARMGSGDWMVVEADESDGTFLKLPADIAVVTN
str.JK7        PIVRRAEMLAELMRFRRAIAVGGTHGKTTTTSMIAALLDAGRFDPMVINGGIINAYGTNARMGSGDWMVVEADESDGTFLKLPADIAVVTN
str.JK73rel    PIVRRAEMLAELMRFRRAIAVGGTHGKTTTTSMIAALLDAGRFDPMVINGGIINAYGTNARMGSGDWMVVEADESDGTFLKLPADIAVVTN
str.BQ2-D70    PIVRRAEMLAELMRFRRAIAVGGTHGKTTTTSMIAALLDAGRFDPMVINGGIINAYGTNARMGSGDWMVVEADESDGTFLKLPADIAVVTN
str.CO20_0321  PIVRRAEMLAELMRFRRAIAVGGTHGKTTTTSMIAALLDAGRFDPMVINGGIINAYGTNARMGSGDWMVVEADESDGTFLKLPADIAVVTN
str.CO21_0024  PIVRRAEMLAELMRFRRAIAVGGTHGKTTTTSMIAALLDAGRFDPMVINGGIINAYGTNARMGSGDWMVVEADESDGTFLKLPADIAVVTN
str.CO20_0297  PIVRRAEMLAELMRFRRAIAVGGTHGKTTTTSMIAALLDAGRFDPMVINGGIINAYGTNARMGSGDWMVVEADESDGTFLKLPADIAVVTN
str.CO20_0256  PIVRRAEMLAELMRFRRAIAVGGTHGKTTTTSMIAALLDAGRFDPMVINGGIINAYGTNARMGSGDWMVVEADESDGTFLKLPADIAVVTN
str.CO20_0257  PIVRRAEMLAELMRFRRAIAVGGTHGKTTTTSMIAALLDAGRFDPMVINGGIINAYGTNARMGSGDWMVVEADESDGTFLKLPADIAVVTN
str.NCTC12899  PIVRRAEMLAELMRFRRAIAVGGTHGKTTTTSMIAALLDAGRFDPMVINGGIINAYGTNARMGSGDWMVVEADESDGTFLKLPADIAVVTN
str.RM-11      PIVRRAEMLAELMRFRRAIAVGGTHGKTTTTSMIAALLDAGRFDPMVINGGIINAYGTNARMGSGDWMVVEADESDGTFLKLPADIAVVTN
str.MF1-1      PIVRRAEMLAELMRFRRAIAVGGTHGKTTTTSMIAALLDAGRFDPMVINGGIINAYGTNARMGSGDWMVVEADESDGTFLKLPADIAVVTN
```

```
                    190       200       210       220       230       240       250       260       270
str.Toulouse   IDREHLDHYGSFCAVREAFRQFVENVPFYGFAVLCLDHPEVQSLASRIDDRWVITYGTNPQADIRFLNLSMDGQKTHFDVFIRSRKTGKET
str.JK12       IDREHLDHYGSFCAVREAFRQFVENVPFYGFAVLCLDHPEVQSLASRIDDRWVITYGTNPQADIRFLNLSMDGQKTHFDVFIRSRKTGKET
str.CCUG45777  IDREHLDHYGSFCAVREAFRQFVENVPFYGFAVLCLDHPEVQSLASRIDDRWVITYGTNPQADIRFLNLSMDGQKTHFDVFIRSRKTGKET
str.JK67       IDREHLDHYGSFCAVREAFRQFVENVPFYGFAVLCLDHPEVQSLASRIDDRWVITYGTNPQADIRFLNLSMDGQKTHFDVFIRSRKTGKET
str.JK56       IDREHLDHYGSFCAVREAFRQFVENVPFYGFAVLCLDHPEVQSLASRIDDRWVITYGTNPQADIRFLNLSMDGQKTHFDVFIRSRKTGKET
str.JK19       IDREHLDHYGSFCAVREAFRQFVENVPFYGFAVLCLDHPEVQSLASRIDDRWVITYGTNPQADIRFLNLSMDGQKTHFDVFIRSRKTGKET
str.JK63       IDREHLDHYGSFCAVREAFRQFVENVPFYGFAVLCLDHPEVQSLASRIDDRWVITYGTNPQADIRFLNLSMDGQKTHFDVFIRSRKTGKET
str.JK39       IDREHLDHYGSFCAVREAFRQFVENVPFYGFAVLCLDHPEVQSLASRIDDRWVITYGTNPQADIRFLNLSMDGQKTHFDVFIRSRKTGKET
str.JK68       IDREHLDHYGSFCAVREAFRQFVENVPFYGFAVLCLDHPEVQSLASRIDDRWVITYGTNPQADIRFLNLSMDGQKTHFDVFIRSRKTGKET
str.JK31       IDREHLDHYGSFCAVREAFRQFVENVPFYGFAVLCLDHPEVQSLASRIDDRWVITYGTNPQADIRFLNLSMDGQKTHFDVFIRSRKTGKET
str.G1713      IDREHLDHYGSFCAVREAFRQFVENVPFYGFAVLCLDHPEVQSLASRIDDRWVITYGTNPQADIRFLNLSMDGQKTHFDVFIRSRKTGKET
str.G1712      IDREHLDHYGSFCAVREAFRQFVENVPFYGFAVLCLDHPEVQSLASRIDDRWVITYGTNPQADIRFLNLSMDGQKTHFDVFIRSRKTGKET
str.JK73       IDREHLDHYGSFCAVREAFRQFVENVPFYGFAVLCLDHPEVQSLASRIDDRWVITYGTNPQADIRFLNLSMDGQKTHFDVFIRSRKTGKET
str.JK7        IDREHLDHYGSFCAVREAFRQFVENVPFYGFAVLCLDHPEVQSLASRIDDRWVITYGTNPQADIRFLNLSMDGQKTHFDVFIRSRKTGKET
str.JK73rel    IDREHLDHYGSFCAVREAFRQFVENVPFYGFAVLCLDHPEVQSLASRIDDRWVITYGTNPQADIRFLNLSMDGQKTHFDVFIRSRKTGKET
str.BQ2-D70    IDREHLDHYGSFCAVREAFRQFVENVPFYGFAVLCLDHPEVQSLASRIDDRWVITYGTNPQADIRFLNLSMDGQKTHFDVFIRSRKTGKET
str.CO20_0321  IDREHLDHYGSFCAVREAFRQFVENVPFYGFAVLCLDHPEVQSLASRIDDRWVITYGTNPQADIRFLNLSMDGQKTHFDVFIRSRKTGKET
str.CO21_0024  IDREHLDHYGSFCAVREAFRQFVENVPFYGFAVLCLDHPEVQSLASRIDDRWVITYGTNPQADIRFLNLSMDGQKTHFDVFIRSRKTGKET
str.CO20_0297  IDREHLDHYGSFCAVREAFRQFVENVPFYGFAVLCLDHPEVQSLASRIDDRWVITYGTNPQADIRFLNLSMDGQKTHFDVFIRSRKTGKET
str.CO20_0256  IDREHLDHYGSFCAVREAFRQFVENVPFYGFAVLCLDHPEVQSLASRIDDRWVITYGTNPQADIRFLNLSMDGQKTHFDVFIRSRKTGKET
str.CO20_0257  IDREHLDHYGSFCAVREAFRQFVENVPFYGFAVLCLDHPEVQSLASRIDDRWVITYGTNPQADIRFLNLSMDGQKTHFDVFIRSRKTGKET
str.NCTC12899  IDREHLDHYGSFCAVREAFRQFVENVPFYGFAVLCLDHPEVQSLASRIDDRWVITYGTNPQADIRFLNLSMDGQKTHFDVFIRSRKTGKET
str.RM-11      IDREHLDHYGSFCAVREAFRKFVENVPFYGFAVLCLDHPEVQSLASRIDDRWVITYGTNPQADIRFLNLSMDGQKTHFDVFIRSRKTGKET
str.MF1-1      IDREHLDHYGSFCAVREAFRQFVENVPFYGFAVLCLDHPEVQSLASRIDDRWVITYGTNPQADIRFLNLSMDGQKTHFDVFIRSRKTGKET
```

```
                   280       290       300       310       320       330       340       350       360
str.Toulouse   ELKNLVLPMSGQHNVSNATAAIAIAHELGISNESIKKGLAEFGGVKRRFTQTGSWRGIEIFDDYGHHPVEIKAVLYAARESAKGRVIAIVQ
str.JK12       ELKNLVLPMSGQHNVSNATAAIAIAHELGISNESIKKGLAEFGGVKRRFTQTGSWRGIEIFDDYGHHPVEIKAVLYAARESAKGRVIAIVQ
str.CCUG45777  ELKNLVLPMSGQHNVSNATAAIAIAHELGISNESIKKGLAEFGGVKRRFTQTGSWRGIEIFDDYGHHPVEIKAVLYAARESAKGRVIAIVQ
str.JK67       ELKNLVLPMSGQHNVSNATAAIAIAHELGISNESIKKGLAEFGGVKRRFTQTGSWRGIEIFDDYGHHPVEIKAVLYAARESAKGRVIAIVQ
str.JK56       ELKNLVLPMSGQHNVSNATAAIAIAHELGISNESIKKGLAEFGGVKRRFTQTGSWRGIEIFDDYGHHPVEIKAVLYAARESAKGRVIAIVQ
str.JK19       ELKNLVLPMSGQHNVSNATAAIAIAHELGISNESIKKGLAEFGGVKRRFTQTGSWRGIEIFDDYGHHPVEIKAVLYAARESAKGRVIAIVQ
str.JK63       ELKNLVLPMSGQHNVSNATAAIAIAHELGISNESIKKGLAEFGGVKRRFTQTGSWRGIEIFDDYGHHPVEIKAVLYAARESAKGRVIAIVQ
str.JK39       ELKNLVLPMSGQHNVSNATAAIAIAHELGISNESIKKGLAEFGGVKRRFTQTGSWRGIEIFDDYGHHPVEIKAVLYAARESAKGRVIAIVQ
str.JK68       ELKNLVLPMSGQHNVSNATAAIAIAHELGISNESIKKGLAEFGGVKRRFTQTGSWRGIEIFDDYGHHPVEIKAVLYAARESAKGRVIAIVQ
str.JK31       ELKNLVLPMSGQHNVSNATAAIAIAHELGISNESIKKGLAEFGGVKRRFTQTGSWRGIEIFDDYGHHPVEIKAVLYAARESAKGRVIAIVQ
str.G1713      ELKNLVLPMSGQHNVSNATAAIAIAHELGISNESIKKGLAEFGGVKRRFTQTGSWRGIEIFDDYGHHPVEIKAVLYAARESAKGRVIAIVQ
str.G1712      ELKNLVLPMSGQHNVSNATAAIAIAHELGISNESIKKGLAEFGGVKRRFTQTGSWRGIEIFDDYGHHPVEIKAVLYAARESAKGRVIAIVQ
str.JK73       ELKNLVLPMSGQHNVSNATAAIAIAHELGISNESIKKGLAEFGGVKRRFTQTGSWRGIEIFDDYGHHPVEIKAVLYAARESAKGRVIAIVQ
str.JK7        ELKNLVLPMSGQHNVSNATAAIAIAHELGISNESIKKGLAEFGGVKRRFTQTGSWRGIEIFDDYGHHPVEIKAVLYAARESAKGRVIAIVQ
str.JK73rel    ELKNLVLPMSGQHNVSNATAAIAIAHELGISNESIKKGLAEFGGVKRRFTQTGSWRGIEIFDDYGHHPVEIKAVLYAARESAKGRVIAIVQ
str.BQ2-D70    ELKNLVLPMSGQHNVSNATAAIAIAHELGISNESIKKGLAEFGGVKRRFTQTGSWRGIEIFDDYGHHPVEIKAVLYAARESAKGRVIAIVQ
str.CO20_0321  ELKNLVLPMSGQHNVSNATAAIAIAHELGISNESIKKGLAEFGGVKRRFTQTGSWRGIEIFDDYGHHPVEIKAVLYAARESAKGRVIAIVQ
str.CO21_0024  ELKNLVLPMSGQHNVSNATAAIAIAHELGISNESIKKGLAEFGGVKRRFTQTGSWRGIEIFDDYGHHPVEIKAVLYAARESAKGRVIAIVQ
str.CO20_0297  ELKNLVLPMSGQHNVSNATAAIAIAHELGISNESIKKGLAEFGGVKRRFTQTGSWRGIEIFDDYGHHPVEIKAVLYAARESAKGRVIAIVQ
str.CO20_0256  ELKNLVLPMSGQHNVSNATAAIAIAHELGISNESIKKGLAEFGGVKRRFTQTGSWRGIEIFDDYGHHPVEIKAVLYAARESAKGRVIAIVQ
str.CO20_0257  ELKNLVLPMSGQHNVSNATAAIAIAHELGISNESIKKGLAEFGGVKRRFTQTGSWRGIEIFDDYGHHPVEIKAVLYAARESAKGRVIAIVQ
str.NCTC12899  ELKNLVLPMSGQHNVSNATAAIAIAHELGISNESIKKGLAEFGGVKRRFTQTGSWRGIEIFDDYGHHPVEIKAVLYAARESAKGRVIAIVQ
str.RM-11      ELKNLVLPMSGQHNVSNATAAIAIAHELGISNESIKKGLAEFGGVKRRFTQTGSWRGIEIFDDYGHHPVEIKAVLYAARESAKGRVIAIVQ
str.MF1-1      ELKNLVLPMSGQHNVSNATAAIAIAHELGISNESIKKGLAEFGGVKRRFTQTGSWRGIEIFDDYGHHPVEIKAVLYAARESAKGRVIAIVQ
```

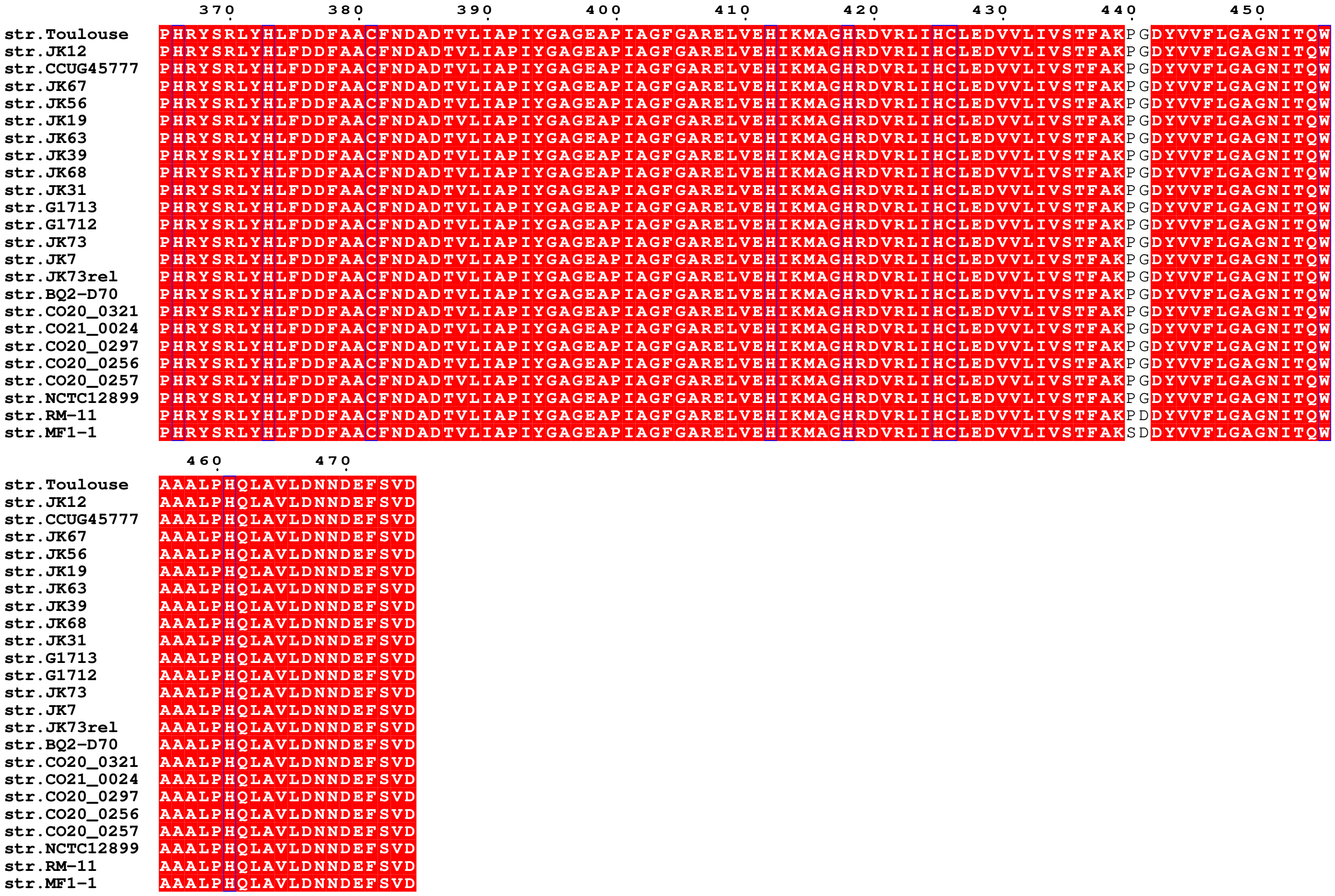
370 380 390 400 410 420 430 440 450
str.Toulouse PHRYSRLYHLFDDFAACFNDADTVLIAPIYGAGEAPIAGFGARELVEHIKMAGHRDVRLIHCLEDVVLIVSTFAKPGDYVVFLGAGNITQW
str.JK12 PHRYSRLYHLFDDFAACFNDADTVLIAPIYGAGEAPIAGFGARELVEHIKMAGHRDVRLIHCLEDVVLIVSTFAKPGDYVVFLGAGNITQW
str.CCUG45777 PHRYSRLYHLFDDFAACFNDADTVLIAPIYGAGEAPIAGFGARELVEHIKMAGHRDVRLIHCLEDVVLIVSTFAKPGDYVVFLGAGNITQW
str.JK67 PHRYSRLYHLFDDFAACFNDADTVLIAPIYGAGEAPIAGFGARELVEHIKMAGHRDVRLIHCLEDVVLIVSTFAKPGDYVVFLGAGNITQW
str.JK56 PHRYSRLYHLFDDFAACFNDADTVLIAPIYGAGEAPIAGFGARELVEHIKMAGHRDVRLIHCLEDVVLIVSTFAKPGDYVVFLGAGNITQW
str.JK19 PHRYSRLYHLFDDFAACFNDADTVLIAPIYGAGEAPIAGFGARELVEHIKMAGHRDVRLIHCLEDVVLIVSTFAKPGDYVVFLGAGNITQW
str.JK63 PHRYSRLYHLFDDFAACFNDADTVLIAPIYGAGEAPIAGFGARELVEHIKMAGHRDVRLIHCLEDVVLIVSTFAKPGDYVVFLGAGNITQW
str.JK39 PHRYSRLYHLFDDFAACFNDADTVLIAPIYGAGEAPIAGFGARELVEHIKMAGHRDVRLIHCLEDVVLIVSTFAKPGDYVVFLGAGNITQW
str.JK68 PHRYSRLYHLFDDFAACFNDADTVLIAPIYGAGEAPIAGFGARELVEHIKMAGHRDVRLIHCLEDVVLIVSTFAKPGDYVVFLGAGNITQW
str.JK31 PHRYSRLYHLFDDFAACFNDADTVLIAPIYGAGEAPIAGFGARELVEHIKMAGHRDVRLIHCLEDVVLIVSTFAKPGDYVVFLGAGNITQW
str.G1713 PHRYSRLYHLFDDFAACFNDADTVLIAPIYGAGEAPIAGFGARELVEHIKMAGHRDVRLIHCLEDVVLIVSTFAKPGDYVVFLGAGNITQW
str.G1712 PHRYSRLYHLFDDFAACFNDADTVLIAPIYGAGEAPIAGFGARELVEHIKMAGHRDVRLIHCLEDVVLIVSTFAKPGDYVVFLGAGNITQW
str.JK73 PHRYSRLYHLFDDFAACFNDADTVLIAPIYGAGEAPIAGFGARELVEHIKMAGHRDVRLIHCLEDVVLIVSTFAKPGDYVVFLGAGNITQW
str.JK7 PHRYSRLYHLFDDFAACFNDADTVLIAPIYGAGEAPIAGFGARELVEHIKMAGHRDVRLIHCLEDVVLIVSTFAKPGDYVVFLGAGNITQW
str.JK73rel PHRYSRLYHLFDDFAACFNDADTVLIAPIYGAGEAPIAGFGARELVEHIKMAGHRDVRLIHCLEDVVLIVSTFAKPGDYVVFLGAGNITQW
str.BQ2-D70 PHRYSRLYHLFDDFAACFNDADTVLIAPIYGAGEAPIAGFGARELVEHIKMAGHRDVRLIHCLEDVVLIVSTFAKPGDYVVFLGAGNITQW
str.CO20_0321 PHRYSRLYHLFDDFAACFNDADTVLIAPIYGAGEAPIAGFGARELVEHIKMAGHRDVRLIHCLEDVVLIVSTFAKPGDYVVFLGAGNITQW
str.CO21_0024 PHRYSRLYHLFDDFAACFNDADTVLIAPIYGAGEAPIAGFGARELVEHIKMAGHRDVRLIHCLEDVVLIVSTFAKPGDYVVFLGAGNITQW
str.CO20_0297 PHRYSRLYHLFDDFAACFNDADTVLIAPIYGAGEAPIAGFGARELVEHIKMAGHRDVRLIHCLEDVVLIVSTFAKPGDYVVFLGAGNITQW
str.CO20_0256 PHRYSRLYHLFDDFAACFNDADTVLIAPIYGAGEAPIAGFGARELVEHIKMAGHRDVRLIHCLEDVVLIVSTFAKPGDYVVFLGAGNITQW
str.CO20_0257 PHRYSRLYHLFDDFAACFNDADTVLIAPIYGAGEAPIAGFGARELVEHIKMAGHRDVRLIHCLEDVVLIVSTFAKPGDYVVFLGAGNITQW
str.NCTC12899 PHRYSRLYHLFDDFAACFNDADTVLIAPIYGAGEAPIAGFGARELVEHIKMAGHRDVRLIHCLEDVVLIVSTFAKPGDYVVFLGAGNITQW
str.RM-11 PHRYSRLYHLFDDFAACFNDADTVLIAPIYGAGEAPIAGFGARELVEHIKMAGHRDVRLIHCLEDVVLIVSTFAKPDDYVVFLGAGNITQW
str.MF1-1 PHRYSRLYHLFDDFAACFNDADTVLIAPIYGAGEAPIAGFGARELVEHIKMAGHRDVRLIHCLEDVVLIVSTFAKSDDYVVFLGAGNITQW
460 470
str.Toulouse AAALPHQLAVLDNNDEFSVD
str.JK12 AAALPHQLAVLDNNDEFSVD
str.CCUG45777 AAALPHQLAVLDNNDEFSVD
str.JK67 AAALPHQLAVLDNNDEFSVD
str.JK56 AAALPHQLAVLDNNDEFSVD
str.JK19 AAALPHQLAVLDNNDEFSVD
str.JK63 AAALPHQLAVLDNNDEFSVD
str.JK39 AAALPHQLAVLDNNDEFSVD
str.JK68 AAALPHQLAVLDNNDEFSVD
str.JK31 AAALPHQLAVLDNNDEFSVD
str.G1713 AAALPHQLAVLDNNDEFSVD
str.G1712 AAALPHQLAVLDNNDEFSVD
str.JK73 AAALPHQLAVLDNNDEFSVD
str.JK7 AAALPHQLAVLDNNDEFSVD
str.JK73rel AAALPHQLAVLDNNDEFSVD
str.BQ2-D70 AAALPHQLAVLDNNDEFSVD
str.CO20_0321 AAALPHQLAVLDNNDEFSVD
str.CO21_0024 AAALPHQLAVLDNNDEFSVD
str.CO20_0297 AAALPHQLAVLDNNDEFSVD
str.CO20_0256 AAALPHQLAVLDNNDEFSVD
str.CO20_0257 AAALPHQLAVLDNNDEFSVD
str.NCTC12899 AAALPHQLAVLDNNDEFSVD
str.RM-11 AAALPHQLAVLDNNDEFSVD
str.MF1-1 AAALPHQLAVLDNNDEFSVD

>WP_011179603.1 undecaprenyldiphospho-muramoylpentapeptide beta-N-acetylglucosaminyltransferase [Bartonella quintana str. Toulouse]

```
                 1         10            20            30            40            50            60            70            80            90
str.Toulouse     MAHKKVIVLVAGGTGGHLFPAEAVAVELRQRGYDVHLVTDRRAKCFVSCVDEEHTHIVSSATFTRRHPFALIKTCWILLKGMGQSLALFYK
str.BQ2-D70      MAHKKVIVLVAGGTGGHLFPAEAVAVELRQRGYDVHLVTDRRAKCFVSCVDEEHTHIVSSATFTRRHPFALIKTCWILLKGMGQSLALFYK
str.JK7          MAHKKVIVLVAGGTGGHLFPAEAVAVELRQRGYDVHLVTDRRAKCFVSCVDEEHTHIVSSATFTRRHPFALIKTCWILLKGMGQSLALFYK
str.JK31         MAHKKVIVLVAGGTGGHLFPAEAVAVELRQRGYDVHLVTDRRAKCFVSCVDEEHTHIVSSATFTRRHPFALIKTCWILLKGMGQSLALFYK
str.JK68         MAHKKVIVLVAGGTGGHLFPAEAVAVELRQRGYDVHLVTDRRAKCFVSCVDEEHTHIVSSATFTRRHPFALIKTCWILLKGMGQSLALFYK
str.JK63         MAHKKVIVLVAGGTGGHLFPAEAVAVELRQRGYDVHLVTDRRAKCFVSCVDEEHTHIVSSATFTRRHPFALIKTCWILLKGMGQSLALFYK
str.JK67         MAHKKVIVLVAGGTGGHLFPAEAVAVELRQRGYDVHLVTDRRAKCFVSCVDEEHTHIVSSATFTRRHPFALIKTCWILLKGMGQSLALFYK
str.JK56         MAHKKVIVLVAGGTGGHLFPAEAVAVELRQRGYDVHLVTDRRAKCFVSCVDEEHTHIVSSATFTRRHPFALIKTCWILLKGMGQSLALFYK
str.JK12         MAHKKVIVLVAGGTGGHLFPAEAVAVELRQRGYDVHLVTDRRAKCFVSCVDEEHTHIVSSATFTRRHPFALIKTCWILLKGMGQSLALFYK
str.NCTC12899    MAHKKVIVLVAGGTGGHLFPAEAVAVELRQRGYDVHLVTDRRAKCFVSCVDEEHTHIVSSATFTRRHPFALIKTCWILLKGMGQSLALFYK
str.JK39         MAHKKVIVLVAGGTGGHLFPAEAVAVELRQRGYDVHLVTDRRAKCFVSCVDEEHTHIVSSATFTRRHPFALIKTCWILLKGMGQSLALFYK
str.JK19         MAHKKVIVLVAGGTGGHLFPAEAVAVELRQRGYDVHLVTDRRAKCFVSCVDEEHTHIVSSATFTRRHPFALIKTCWILLKGMGQSLALFYK
str.CO21_0024    MAHKKVIVLVAGGTGGHLFPAEAVAVELRQRGYDVHLVTDRRAKCFVSCVDEEHTHIVSSATFTRRHPFALIKTCWILLKGMGQSLALFYK
str.CO20_0321    MAHKKVIVLVAGGTGGHLFPAEAVAVELRQRGYDVHLVTDRRAKCFVSCVDEEHTHIVSSATFTRRHPFALIKTCWILLKGMGQSLALFYK
str.JK73rel      MAHKKVIVLVAGGTGGHLFPAEAVAVELRQRGYDVHLVTDRRAKCFVSCVDEEHTHIVSSATFTRRHPFALIKTCWILLKGMGQSLALFYK
str.JK73         MAHKKVIVLVAGGTGGHLFPAEAVAVELRQRGYDVHLVTDRRAKCFVSCVDEEHTHIVSSATFTRRHPFALIKTCWILLKGMGQSLALFYK
str.G1712        MAHKKVIVLVAGGTGGHLFPAEAVAVELRQRGYDVHLVTDRRAKCFVSCVDEEHTHIVSSATFTRRHPFALIKTCWILLKGMGQSLALFYK
str.G1713        MAHKKVIVLVAGGTGGHLFPAEAVAVELRQRGYDVHLVTDRRAKCFVSCVDEEHTHIVSSATFTRRHPFALIKTCWILLKGMGQSLALFYK
str.CO20_0256    MAHKKVIVLVAGGTGGHLFPAEAVAVELRQRGYDVHLVTDRRAKCFVSCVDEEHTHIVSSATFTRRHPFALIKTCWILLKGMGQSLALFYK
str.CO20_0297    MAHKKVIVLVAGGTGGHLFPAEAVAVELRQRGYDVHLVTDRRAKCFVSCVDEEHTHIVSSATFTRRHPFALIKTCWILLKGMGQSLALFYK
str.CCUG45777    MAHKKVIVLVAGGTGGHLFPAEAVAVELRQRGYDVHLVTDRRAKCFVSCVDEEHTHIVSSATFTRRHPFALIKTCWILLKGMGQSLALFYK
str.CO20_0257    MAHKKVIVLVAGGTGGHLFPAEAVAVELRQRGYDVHLVTDRRAKCFVSCVDEEHTHIVSSATFTRRHPFALIKTCWILLKGMGQSLALFYK
str.MF1-1        MAHKKVIVLVAGGTGGHLFPAEAVAVELRQRGYDVHLVTDRRAKCFVSCVDEEHTHIVSSATFTRRHPFALIKTCWILLKGMGQSLALFYK
str.RM-11        MAHKKVIVLVAGGTGGHLFPAEAVAVELRQRGYDVHLVTDRRAKCFVSCVDEEHTHIVSSATFTRRHPFALIKTCWILLKGMGQSLALFYK

                      100         110         120         130         140         150         160         170         180
str.Toulouse     LRPVLVGGFGGYPTFPPLLVAALMRCVTFIHEQNAIMGRANRVLAVFVHAIAGGLLSQTGTHAHKTLLIGNPMREVVLKAAKIPYRPSLGE
str.BQ2-D70      LRPVLVGGFGGYPTFPPLLVAALMRCVTFIHEQNAIMGRANRVLAVFVHAIAGGLLSQTGTHAHKTLLIGNPMREVVLKAAKIPYRPSLGE
str.JK7          LRPVLVGGFGGYPTFPPLLVAALMRCVTFIHEQNAIMGRANRVLAVFVHAIAGGLLSQTGTHAHKTLLIGNPMREVVLKAAKIPYRPSLGE
str.JK31         LRPVLVGGFGGYPTFPPLLVAALMRCVTFIHEQNAIMGRANRVLAVFVHAIAGGLLSQTGTHAHKTLLIGNPMREVVLKAAKIPYRPSLGE
str.JK68         LRPVLVGGFGGYPTFPPLLVAALMRCVTFIHEQNAIMGRANRVLAVFVHAIAGGLLSQTGTHAHKTLLIGNPMREVVLKAAKIPYRPSLGE
str.JK63         LRPVLVGGFGGYPTFPPLLVAALMRCVTFIHEQNAIMGRANRVLAVFVHAIAGGLLSQTGTHAHKTLLIGNPMREVVLKAAKIPYRPSLGE
str.JK67         LRPVLVGGFGGYPTFPPLLVAALMRCVTFIHEQNAIMGRANRVLAVFVHAIAGGLLSQTGTHAHKTLLIGNPMREVVLKAAKIPYRPSLGE
str.JK56         LRPVLVGGFGGYPTFPPLLVAALMRCVTFIHEQNAIMGRANRVLAVFVHAIAGGLLSQTGTHAHKTLLIGNPMREVVLKAAKIPYRPSLGE
str.JK12         LRPVLVGGFGGYPTFPPLLVAALMRCVTFIHEQNAIMGRANRVLAVFVHAIAGGLLSQTGTHAHKTLLIGNPMREVVLKAAKIPYRPSLGE
str.NCTC12899    LRPVLVGGFGGYPTFPPLLVAALMRCVTFIHEQNAIMGRANRVLAVFVHAIAGGLLSQTGTHAHKTLLIGNPMREVVLKAAKIPYRPSLGE
str.JK39         LRPVLVGGFGGYPTFPPLLVAALMRCVTFIHEQNAIMGRANRVLAVFVHAIAGGLLSQTGTHAHKTLLIGNPMREVVLKAAKIPYRPSLGE
str.JK19         LRPVLVGGFGGYPTFPPLLVAALMRCVTFIHEQNAIMGRANRVLAVFVHAIAGGLLSQTGTHAHKTLLIGNPMREVVLKAAKIPYRPSLGE
str.CO21_0024    LRPVLVGGFGGYPTFPPLLVAALMRCVTFIHEQNAIMGRANRVLAVFVHAIAGGLLSQTGTHAHKTLLIGNPMREVVLKAAKIPYRPSLGE
str.CO20_0321    LRPVLVGGFGGYPTFPPLLVAALMRCVTFIHEQNAIMGRANRVLAVFVHAIAGGLLSQTGTHAHKTLLIGNPMREVVLKAAKIPYRPSLGE
str.JK73rel      LRPVLVGGFGGYPTFPPLLVAALMRCVTFIHEQNAIMGRANRVLAVFVHAIAGGLLSQTGTHAHKTLLIGNPMREVVLKAAKIPYRPSLGE
str.JK73         LRPVLVGGFGGYPTFPPLLVAALMRCVTFIHEQNAIMGRANRVLAVFVHAIAGGLLSQTGTHAHKTLLIGNPMREVVLKAAKIPYRPSLGE
str.G1712        LRPVLVGGFGGYPTFPPLLVAALMRCVTFIHEQNAIMGRANRVLAVFVHAIAGGLLSQTGTHAHKTLLIGNPMREVVLKAAKIPYRPSLGE
str.G1713        LRPVLVGGFGGYPTFPPLLVAALMRCVTFIHEQNAIMGRANRVLAVFVHAIAGGLLSQTGTHAHKTLLIGNPMREVVLKAAKIPYRPSLGE
str.CO20_0256    LRPVLVGGFGGYPTFPPLLVAALMRCVTFIHEQNAIMGRANRVLAVFVHAIAGGLLSQTGTHAHKTLLIGNPMREVVLKAAKIPYRPSLGE
str.CO20_0297    LRPVLVGGFGGYPTFPPLLVAALMRCVTFIHEQNAIMGRANRVLAVFVHAIAGGLLSQTGTHAHKTLLIGNPMREVVLKAAKIPYRPSLGE
str.CCUG45777    LRPVLVGGFGGYPTFPPLLVAALMRCVTFIHEQNAIMGRANRVLAVFVHAIAGGLLSQTGTHAHKTLLIGNPMREVVLKAAKIPYRPSLGE
str.CO20_0257    LRPVLVGGFGGYPTFPPLLVAALMRCVTFIHEQNAIMGRANRVLAVFVHAIAGGLLSQTGTHAHKTLLIGNPMREVVLKAAKIPYRPSLGE
str.MF1-1        LRPVLVGGFGGYPTFPPLLVAVLMRCVTFIHEQNAIMGRANRVLAVFVHAIAGGLLSQTGPHAHKTLLIGNPMRDVVLKAAKIPYRPSLGE
str.RM-11        LRPVLVGGFGGYPTFPPLLVAVLMRCVTFIHEQNAIMGRANRVLAVFVHAIAGGLLSQTGPHAHKTLLIGNPMRDVVLKAAKIPYHPSLGE

                     190           200           210           220           230           240           250           260           270
str.Toulouse     KPFHFLVFGGSQGASFFSRIVPEAIALLNNKIRRRLRIIQQVRGEVVELMKTYRSMGVQAEVAPFFNDMAERMAHAHFILSRAGASSVCEI
str.BQ2-D70      KPFHFLVFGGSQGASFFSRIVPEAIALLNNKIRRRLRIIQQVRAEVVELMKTYRSMGVQAEVAPFFNDMAERMAHAHFILSRAGASSVCEI
str.JK7          KPFHFLVFGGSQGASFFSRIVPEAIALLNNKIRRRLRIIQQVRAEVVELMKTYRSMGVQAEVAPFFNDMAERMAHAHFILSRAGASSVCEI
str.JK31         KPFHFLVFGGSQGASFFSRIVPEAIALLNNKIRRRLRIIQQVRAEVVELMKTYRSMGVQAEVAPFFNDMAERMAHAHFILSRAGASSVCEI
str.JK68         KPFHFLVFGGSQGASFFSRIVPEAIALLNNKIRRRLRIIQQVRAEVVELMKTYRSMGVQAEVAPFFNDMAERMAHAHFILSRAGASSVCEI
str.JK63         KPFHFLVFGGSQGASFFSRIVPEAIALLNNKIRRRLRIIQQVRAEVVELMKTYRSMGVQAEVAPFFNDMAERMAHAHFILSRAGASSVCEI
str.JK67         KPFHFLVFGGSQGASFFSRIVPEAIALLNNKIRRRLRIIQQVRAEVVELMKTYRSMGVQAEVAPFFNDMAERMAHAHFILSRAGASSVCEI
str.JK56         KPFHFLVFGGSQGASFFSRIVPEAIALLNNKIRRRLRIIQQVRAEVVELMKTYRSMGVQAEVAPFFNDMAERMAHAHFILSRAGASSVCEI
str.JK12         KPFHFLVFGGSQGASFFSRIVPEAIALLNNKIRRRLRIIQQVRAEVVELMKTYRSMGVQAEVAPFFNDMAERMAHAHFILSRAGASSVCEI
str.NCTC12899    KPFHFLVFGGSQGASFFSRIVPEAIALLNNKIRRRLRIIQQVRGEVVELMKTYRSMGVQAEVAPFFNDMAERMAHAHFILSRAGASSVCEI
str.JK39         KPFHFLVFGGSQGASFFSRIVPEAIALLNNKIRRRLRIIQQVRGEVVELMKTYRSMGVQAEVAPFFNDMAERMAHAHFILSRAGASSVCEI
str.JK19         KPFHFLVFGGSQGASFFSRIVPEAIALLNNKIRRRLRIIQQVRGEVVELMKTYRSMGVQAEVAPFFNDMAERMAHAHFILSRAGASSVCEI
str.CO21_0024    KPFHFLVFGGSQGASFFSRIVPEAIALLNNKIRRRLRIIQQVRGEVVELMKTYRSMGVQAEVAPFFNDMAERMAHAHFILSRAGASSVCEI
str.CO20_0321    KPFHFLVFGGSQGASFFSRIVPEAIALLNNKIRRRLRIIQQVRGEVVELMKTYRSMGVQAEVAPFFNDMAERMAHAHFILSRAGASSVCEI
str.JK73rel      KPFHFLVFGGSQGASFFSRIVPEAIALLNNKIRRRLRIIQQVRGEVVELMKTYRSMGVQAEVAPFFNDMAERMAHAHFILSRAGASSVCEI
str.JK73         KPFHFLVFGGSQGASFFSRIVPEAIALLNNKIRRRLRIIQQVRGEVVELMKTYRSMGVQAEVAPFFNDMAERMAHAHFILSRAGASSVCEI
str.G1712        KPFHFLVFGGSQGASFFSRIVPEAIALLNNKIRRRLRIIQQVRGEVVELMKTYRSMGVQAEVAPFFNDMAERMAHAHFILSRAGASSVCEI
str.G1713        KPFHFLVFGGSQGASFFSRIVPEAIALLNNKIRRRLRIIQQVRGEVVELMKTYRSMGVQAEVAPFFNDMAERMAHAHFILSRAGASSVCEI
str.CO20_0256    KPFHFLVFGGSQGASFFSRIVPEAIALLNNKIRRRLRIIQQVRGEVVELMKTYRSMGVQAEVAPFFNDMAERMAHAHFILSRAGASSVCEI
str.CO20_0297    KPFHFLVFGGSQGASFFSRIVPEAIALLNNKIRRRLRIIQQVRGEVVELMKTYRSMGVQAEVAPFFNDMAERMAHAHFILSRAGASSVCEI
str.CCUG45777    KPFHFLVFGGSQGASFFSRIVPEAIALLNNKIRRRLRIIQQVRGEVVELMKTYRSMGVQAEVAPFFNDMAERMAHAHFILSRAGASSVCEI
str.CO20_0257    KPFHFLVFGGSQGASFFSRIVPEAIALLNNKIRRRLRIIQQVRGEVVELMKTYRSMGVQAEVAPFFNDMAERMAHAHFILSRAGASSVCEI
str.MF1-1        KPFHFLVFGGSQGASFFSRIVPEAIALLNNKIRRRLRIIQQVRGEIVELMKTYRGMGVQAEVAPFFNDMAERMAHAHFILSRAGASSVCEI
str.RM-11        KPFHFLVFGGSQGASFFSRIVPEAIALLNNKIRRRLRIIQQVRGEIVELMKTYRGMGVQAEVAPFFNDMAERMAHAHFILSRAGASSVCEI

                       280           290           300           310           320           330           340           350           360
str.Toulouse     AVIGRPALLIPYPYALDHDQAANAALLARVGGAQIVSEKDLTAQRLASLLTQACCAPHLLEKQALAAKKVGQPYATRRLADMAEALIAGRS
str.BQ2-D70      AVIGRPALLIPYPYALDHDQAANAALLARVGGAQIVSEKDLTAQRLASLLTQACCAPHLLEKQALAAKKVGQPYATRRLADMAEALIAGRS
str.JK7          AVIGRPALLIPYPYALDHDQAANAALLARVGGAQIVSEKDLTAQRLASLLTQACCAPHLLEKQALAAKKVGQPYATRRLADMAEALIAGRS
str.JK31         AVIGRPALLIPYPYALDHDQAANAALLARVGGAQIVSEKDLTAQRLASLLTQACCAPHLLEKQALAAKKVGQPYATRRLADMAEALIAGRS
str.JK68         AVIGRPALLIPYPYALDHDQAANAALLARVGGAQIVSEKDLTAQRLASLLTQACCAPHLLEKQALAAKKVGQPYATRRLADMAEALIAGRS
str.JK63         AVIGRPALLIPYPYALDHDQAANAALLARVGGAQIVSEKDLTAQRLASLLTQACCAPHLLEKQALAAKKVGQPYATRRLADMAEALIAGRS
str.JK67         AVIGRPALLIPYPYALDHDQAANAALLARVGGAQIVSEKDLTAQRLASLLTQACCAPHLLEKQALAAKKVGQPYATRRLADMAEALIAGRS
str.JK56         AVIGRPALLIPYPYALDHDQAANAALLARVGGAQIVSEKDLTAQRLASLLTQACCAPHLLEKQALAAKKVGQPYATRRLADMAEALIAGRS
str.JK12         AVIGRPALLIPYPYALDHDQAANAALLARVGGAQIVSEKDLTAQRLASLLTQACCAPHLLEKQALAAKKVGQPYATRRLADMAEALIAGRS
str.NCTC12899    AVIGRPALLIPYPYALDHDQAANAALLARVGGAQIVSEKDLTAQRLASLLTQACCAPHLLEKQALAAKKVGQPYATRRLADMAEALIAGRS
str.JK39         AVIGRPALLIPYPYALDHDQAANAALLARVGGAQIVSEKDLTAQRLASLLTQACCAPHLLEKQALAAKKVGQPYATRRLADMAEALIAGRS
str.JK19         AVIGRPALLIPYPYALDHDQAANAALLARVGGAQIVSEKDLTAQRLASLLTQACCAPHLLEKQALAAKKVGQPYATRRLADMAEALIAGRS
str.CO21_0024    AVIGRPALLIPYPYALDHDQAANAALLARVGGAQIVSEKDLTAQRLASLLTQACCAPHLLEKQALAAKKVGQPYATRRLADMAEALIAGRS
str.CO20_0321    AVIGRPALLIPYPYALDHDQAANAALLARVGGAQIVSEKDLTAQRLASLLTQACCAPHLLEKQALAAKKVGQPYATRRLADMAEALIAGRS
str.JK73rel      AVIGRPALLIPYPYALDHDQAANAALLARVGGAQIVSEKDLTAQRLASLLTQACCAPHLLEKQALAAKKVGQPYATRRLADMAEALIAGRS
str.JK73         AVIGRPALLIPYPYALDHDQAANAALLARVGGAQIVSEKDLTAQRLASLLTQACCAPHLLEKQALAAKKVGQPYATRRLADMAEALIAGRS
str.G1712        AVIGRPALLIPYPYALDHDQAANAALLARVGGAQIVSEKDLTAQRLASLLTQACCAPHLLEKQALAAKKVGQPYATRRLADMAEALIAGRS
str.G1713        AVIGRPALLIPYPYALDHDQAANAALLARVGGAQIVSEKDLTAQRLASLLTQACCAPHLLEKQALAAKKVGQPYATRRLADMAEALIAGRS
str.CO20_0256    AVIGRPALLIPYPYALDHDQAANAALLARVGGAQIVSEKDLTAQRLASLLTQACCAPHLLEKQALAAKKVGQPYATRRLADMAEALIAGRS
str.CO20_0297    AVIGRPALLIPYPYALDHDQAANAALLARVGGAQIVSEKDLTAQRLASLLTQACCAPHLLEKQALAAKKVGQPYATRRLADMAEALIAGRS
str.CCUG45777    AVIGRPALLIPYPYALDHDQAANAALLARVGGAQIVSEKDLTAQRLASLLTQACCAPHLLEKQALAAKKVGQPYATRRLADMAEALIAGRS
str.CO20_0257    AVIGRPALLIPYPYALDHDQAANAALLARVGGAQIVSEKDLTAQRLASLLTQACCAPHLLEKQALAAKKVGQPYATRRLADMAEALIAGRS
str.MF1-1        AVIGRPALLIPYPYALDHDQAANAALLARVGGAQIVSEKDLTAQRLASLLTQACCAPHLLEKQALAAKKVGQPYATRRLADMAEALIAGRS
str.RM-11        AVIGRPALLIPYPYALDHDQAANAALLARVGGAQIVSEKDLTAQRLASLLTQACCAPHLLEKQALAAKKVGQPYATRRLADMAEALIAGRS
```

```
                       370
                         .
str.Toulouse      LSDVREEFFDENAV
str.BQ2-D70       LSDVREEFFDENAV
str.JK7           LSDVREEFFDENAV
str.JK31          LSDVREEFFDENAV
str.JK68          LSDVREEFFDENAV
str.JK63          LSDVREEFFDENAV
str.JK67          LSDVREEFFDENAV
str.JK56          LSDVREEFFDENAV
str.JK12          LSDVREEFFDENAV
str.NCTC12899     LSDVREEFFDENAV
str.JK39          LSDVREEFFDENAV
str.JK19          LSDVREEFFDENAV
str.CO21_0024     LSDVREEFFDENAV
str.CO20_0321     LSDVREEFFDENAV
str.JK73rel       LSDVREEFFDENAV
str.JK73          LSDVREEFFDENAV
str.G1712         LSDVREEFFDENAV
str.G1713         LSDVREEFFDENAV
str.CO20_0256     LSDVREEFFDENAV
str.CO20_0297     LSDVREEFFDENAV
str.CCUG45777     LSDVREEFFDENAV
str.CO20_0257     LSDVREEFFDENAV
str.MF1-1         LSDVREEFFDENAV
str.RM-11         LSDVREEFFDENAV
```

>WP_011179606.1 phospho-N-acetylmuramoyl-pentapeptide-transferase [Bartonella quintana str. Toulouse]

```
               1         10        20        30        40        50        60        70        80        90
str.Toulouse   MMLFFSSLSDWFPGVSVFRYITFRTVAAMLTSGLIVFLFGPSIIASLKLRQGKGQPIRADGPQTHFKKAGTPTMGGLMILTGIVVSAFLWC
str.JK12       MMLFFSSLSDWFPGVSVFRYITFRTVAAMLTSGLIVFLFGPSIIASLKLRQGKGQPIRADGPQTHFKKAGTPTMGGLMILTGIVVSAFLWC
str.CCUG45777  MMLFFSSLSDWFPGVSVFRYITFRTVAAMLTSGLIVFLFGPSIIASLKLRQGKGQPIRADGPQTHFKKAGTPTMGGLMILTGIVVSAFLWC
str.JK56       MMLFFSSLSDWFPGVSVFRYITFRTVAAMLTSGLIVFLFGPSIIASLKLRQGKGQPIRADGPQTHFKKAGTPTMGGLMILTGIVVSAFLWC
str.JK67       MMLFFSSLSDWFPGVSVFRYITFRTVAAMLTSGLIVFLFGPSIIASLKLRQGKGQPIRADGPQTHFKKAGTPTMGGLMILTGIVVSAFLWC
str.JK19       MMLFFSSLSDWFPGVSVFRYITFRTVAAMLTSGLIVFLFGPSIIASLKLRQGKGQPIRADGPQTHFKKAGTPTMGGLMILTGIVVSAFLWC
str.JK63       MMLFFSSLSDWFPGVSVFRYITFRTVAAMLTSGLIVFLFGPSIIASLKLRQGKGQPIRADGPQTHFKKAGTPTMGGLMILTGIVVSAFLWC
str.JK39       MMLFFSSLSDWFPGVSVFRYITFRTVAAMLTSGLIVFLFGPSIIASLKLRQGKGQPIRADGPQTHFKKAGTPTMGGLMILTGIVVSAFLWC
str.JK31       MMLFFSSLSDWFPGVSVFRYITFRTVAAMLTSGLIVFLFGPSIIASLKLRQGKGQPIRADGPQTHFKKAGTPTMGGLMILTGIVVSAFLWC
str.JK68       MMLFFSSLSDWFPGVSVFRYITFRTVAAMLTSGLIVFLFGPSIIASLKLRQGKGQPIRADGPQTHFKKAGTPTMGGLMILTGIVVSAFLWC
str.G1713      MMLFFSSLSDWFPGVSVFRYITFRTVAAMLTSGLIVFLFGPSIIASLKLRQGKGQPIRADGPQTHFKKAGTPTMGGLMILTGIVVSAFLWC
str.MF1-1      MMLFFSSLSDWFPGVSVFRYITFRTVAAMLTSGLIVFLFGPSIIASLKLRQGKGQPIRADGPQTHFKKAGTPTMGGLMILTGIVVSAFLWC
str.NCTC12899  MMLFFSSLSDWFPGVSVFRYITFRTVAAMLTSGLIVFLFGPSIIASLKLRQGKGQPIRADGPQTHFKKAGTPTMGGLMILTGIVVSAFLWC
str.CO20_0257  MMLFFSSLSDWFPGVSVFRYITFRTVAAMLTSGLIVFLFGPSIIASLKLRQGKGQPIRADGPQTHFKKAGTPTMGGLMILTGIVVSAFLWC
str.CO20_0256  MMLFFSSLSDWFPGVSVFRYITFRTVAAMLTSGLIVFLFGPSIIASLKLRQGKGQPIRADGPQTHFKKAGTPTMGGLMILTGIVVSAFLWC
str.CO20_0297  MMLFFSSLSDWFPGVSVFRYITFRTVAAMLTSGLIVFLFGPSIIASLKLRQGKGQPIRADGPQTHFKKAGTPTMGGLMILTGIVVSAFLWC
str.CO21_0024  MMLFFSSLSDWFPGVSVFRYITFRTVAAMLTSGLIVFLFGPSIIASLKLRQGKGQPIRADGPQTHFKKAGTPTMGGLMILTGIVVSAFLWC
str.CO20_0321  MMLFFSSLSDWFPGVSVFRYITFRTVAAMLTSGLIVFLFGPSIIASLKLRQGKGQPIRADGPQTHFKKAGTPTMGGLMILTGIVVSAFLWC
str.RM-11      MMLFFSSLSDWFPGVSVFRYITFRTVAAMLTSGLIVFLFGPSIIASLKLRQGKGQPIRADGPQTHFKKAGTPTMGGLMILTGIVVSAFLWC
str.BQ2-D70    MMLFFSSLSDWFPGVSVFRYITFRTVAAMLTSGLIVFLFGPSIIASLKLRQGKGQPIRADGPQTHFKKAGTPTMGGLMILTGIVVSAFLWC
str.JK73rel    MMLFFSSLSDWFPGVSVFRYITFRTVAAMLTSGLIVFLFGPSIIASLKLRQGKGQPIRADGPQTHFKKAGTPTMGGLMILTGIVVSAFLWC
str.JK7        MMLFFSSLSDWFPGVSVFRYITFRTVAAMLTSGLIVFLFGPSIIASLKLRQGKGQPIRADGPQTHFKKAGTPTMGGLMILTGIVVSAFLWC
str.JK73       MMLFFSSLSDWFPGVSVFRYITFRTVAAMLTSGLIVFLFGPSIIASLKLRQGKGQPIRADGPQTHFKKAGTPTMGGLMILTGIVVSAFLWC
str.G1712      MMLFFSSLSDWFPGVSVFRYITFRTVAAMLTSGLIVFLFGPSIIASLKLRQGKGQPIRADGPQTHFKKAGTPTMGGLMILTGIVVSAFLWC

                       100       110       120       130       140       150       160       170       180
str.Toulouse   NLSNIYFWVSLFVMLSFGMIGFYDDYLKVTKQTEKGFSGKARLSLEFLIAIIAAFVLLQVGSSGLALPFVKDYFINLSWFFLPFSAFVIVG
str.JK12       NLSNIYFWVSLFVMLSFGMIGFYDDYLKVTKQTEKGFSGKARLSLEFLIAIIAAFVLLQVGSSGLALPFVKDYFINLSWFFLPFSAFVIVG
str.CCUG45777  NLSNIYFWVSLFVMLSFGMIGFYDDYLKVTKQTEKGFSGKARLSLEFLIAIIAAFVLLQVGSSGLALPFVKDYFINLSWFFLPFSAFVIVG
str.JK56       NLSNIYFWVSLFVMLSFGMIGFYDDYLKVTKQTEKGFSGKARLSLEFLIAIIAAFVLLQVGSSGLALPFVKDYFINLSWFFLPFSAFVIVG
str.JK67       NLSNIYFWVSLFVMLSFGMIGFYDDYLKVTKQTEKGFSGKARLSLEFLIAIIAAFVLLQVGSSGLALPFVKDYFINLSWFFLPFSAFVIVG
str.JK19       NLSNIYFWVSLFVMLSFGMIGFYDDYLKVTKQTEKGFSGKARLSLEFLIAIIAAFVLLQVGSSGLALPFVKDYFINLSWFFLPFSAFVIVG
str.JK63       NLSNIYFWVSLFVMLSFGMIGFYDDYLKVTKQTEKGFSGKARLSLEFLIAIIAAFVLLQVGSSGLALPFVKDYFINLSWFFLPFSAFVIVG
str.JK39       NLSNIYFWVSLFVMLSFGMIGFYDDYLKVTKQTEKGFSGKARLSLEFLIAIIAAFVLLQVGSSGLALPFVKDYFINLSWFFLPFSAFVIVG
str.JK31       NLSNIYFWVSLFVMLSFGMIGFYDDYLKVTKQTEKGFSGKARLSLEFLIAIIAAFVLLQVGSSGLALPFVKDYFINLSWFFLPFSAFVIVG
str.JK68       NLSNIYFWVSLFVMLSFGMIGFYDDYLKVTKQTEKGFSGKARLSLEFLIAIIAAFVLLQVGSSGLALPFVKDYFINLSWFFLPFSAFVIVG
str.G1713      NLSNIYFWVSLFVMLSFGMIGFYDDYLKVTKQTEKGFSGKARLSLEFLIAIIAAFVLLQVGSSGLALPFVKDYFINLSWFFLPFSAFVIVG
str.MF1-1      NLSNIYFWVSLFVMLSFGMIGFYDDYLKVTKQTEKGFSGKARLSLEFLIAIIAAFVLLQVGSSGLALPFVKDYFINLSWFFLPFSAFVIVG
str.NCTC12899  NLSNIYFWVSLFVMLSFGMIGFYDDYLKVTKQTEKGFSGKARLSLEFLIAIIAAFVLLQVGSSGLALPFVKDYFINLSWFFLPFSAFVIVG
str.CO20_0257  NLSNIYFWVSLFVMLSFGMIGFYDDYLKVTKQTEKGFSGKARLSLEFLIAIIAAFVLLQVGSSGLALPFVKDYFINLSWFFLPFSAFVIVG
str.CO20_0256  NLSNIYFWVSLFVMLSFGMIGFYDDYLKVTKQTEKGFSGKARLSLEFLIAIIAAFVLLQVGSSGLALPFVKDYFINLSWFFLPFSAFVIVG
str.CO20_0297  NLSNIYFWVSLFVMLSFGMIGFYDDYLKVTKQTEKGFSGKARLSLEFLIAIIAAFVLLQVGSSGLALPFVKDYFINLSWFFLPFSAFVIVG
str.CO21_0024  NLSNIYFWVSLFVMLSFGMIGFYDDYLKVTKQTEKGFSGKARLSLEFLIAIIAAFVLLQVGSSGLALPFVKDYFINLSWFFLPFSAFVIVG
str.CO20_0321  NLSNIYFWVSLFVMLSFGMIGFYDDYLKVTKQTEKGFSGKARLSLEFLIAIIAAFVLLQVGSSGLALPFVKDYFINLSWFFLPFSAFVIVG
str.RM-11      NLSNIYFWVSLFVMLSFGMIGFYDDYLKVTKQTEKGFSGKARLSLEFLIAIIAAFVLLQVGSSGLALPFVKDYFINLSWFFLPFSAFVIVG
str.BQ2-D70    NLSNIYFWVSLFVMLSFGMIGFYDDYLKVTKQTEKGFSGKARLSLEFLIAIIAAFVLLQVGSSGLALPFVKDYFINLSWFFLPFSAFVIVG
str.JK73rel    NLSNIYFWVSLFVMLSFGMIGFYDDYLKVTKQTEKGFSGKARLSLEFLIAIIAAFVLLQVGSSGLALPFVKDYFINLSWFFLPFSAFVIVG
str.JK7        NLSNIYFWVSLFVMLSFGMIGFYDDYLKVTKQTEKGFSGKARLSLEFLIAIIAAFVLLQVGSSGLALPFVKDYFINLSWFFLPFSAFVIVG
str.JK73       NLSNIYFWVSLFVMLSFGMIGFYDDYLKVTKQTEKGFSGKARLSLEFLIAIIAAFVLLQVGSSGLALPFVKDYFINLSWFFLPFSAFVIVG
str.G1712      NLSNIYFWVSLFVMLSFGMIGFYDDYLKVTKQTEKGFSGKARLSLEFLIAIIAAFVLLQVGSSGLALPFVKDYFINLSWFFLPFSAFVIVG

                      190       200       210       220       230       240       250       260       270
str.Toulouse   TGNAVNLTDGLDGLAIVPVMVAALSFALIAYLSGNINFADYLQIHYVSGTGELAVLLGAVVGAGLGFLWFNAPPAAIFMGDTGSLALGGLL
str.JK12       TGNAVNLTDGLDGLAIVPVMVAALSFALIAYLSGNINFADYLQIHYVSGTGELAVLLGAVVGAGLGFLWFNAPPAAIFMGDTGSLALGGLL
str.CCUG45777  TGNAVNLTDGLDGLAIVPVMVAALSFALIAYLSGNINFADYLQIHYVSGTGELAVLLGAVVGAGLGFLWFNAPPAAIFMGDTGSLALGGLL
str.JK56       TGNAVNLTDGLDGLAIVPVMVAALSFALIAYLSGNINFADYLQIHYVSGTGELAVLLGAVVGAGLGFLWFNAPPAAIFMGDTGSLALGGLL
str.JK67       TGNAVNLTDGLDGLAIVPVMVAALSFALIAYLSGNINFADYLQIHYVSGTGELAVLLGAVVGAGLGFLWFNAPPAAIFMGDTGSLALGGLL
str.JK19       TGNAVNLTDGLDGLAIVPVMVAALSFALIAYLSGNINFADYLQIHYVSGTGELAVLLGAVVGAGLGFLWFNAPPAAIFMGDTGSLALGGLL
str.JK63       TGNAVNLTDGLDGLAIVPVMVAALSFALIAYLSGNINFADYLQIHYVSGTGELAVLLGAVVGAGLGFLWFNAPPAAIFMGDTGSLALGGLL
str.JK39       TGNAVNLTDGLDGLAIVPVMVAALSFALIAYLSGNINFADYLQIHYVSGTGELAVLLGAVVGAGLGFLWFNAPPAAIFMGDTGSLALGGLL
str.JK31       TGNAVNLTDGLDGLAIVPVMVAALSFALIAYLSGNINFADYLQIHYVSGTGELAVLLGAVVGAGLGFLWFNAPPAAIFMGDTGSLALGGLL
str.JK68       TGNAVNLTDGLDGLAIVPVMVAALSFALIAYLSGNINFADYLQIHYVSGTGELAVLLGAVVGAGLGFLWFNAPPAAIFMGDTGSLALGGLL
str.G1713      TGNAVNLTDGLDGLAIVPVMVAALSFALIAYLSGNINFADYLQIHYVSGTGELAVLLGAVVGAGLGFLWFNAPPAAIFMGDTGSLALGGLL
str.MF1-1      TGNAVNLTDGLDGLAIVPVMVAALSFALIAYLSGNINFADYLQIHYVSGTGELAVLLGAVVGAGLGFLWFNAPPAAIFMGDTGSLALGGLL
str.NCTC12899  TGNAVNLTDGLDGLAIVPVMVAALSFALIAYLSGNINFADYLQIHYVSGTGELAVLLGAVVGAGLGFLWFNAPPAAIFMGDTGSLALGGLL
str.CO20_0257  TGNAVNLTDGLDGLAIVPVMVAALSFALIAYLSGNINFADYLQIHYVSGTGELAVLLGAVVGAGLGFLWFNAPPAAIFMGDTGSLALGGLL
str.CO20_0256  TGNAVNLTDGLDGLAIVPVMVAALSFALIAYLSGNINFADYLQIHYVSGTGELAVLLGAVVGAGLGFLWFNAPPAAIFMGDTGSLALGGLL
str.CO20_0297  TGNAVNLTDGLDGLAIVPVMVAALSFALIAYLSGNINFADYLQIHYVSGTGELAVLLGAVVGAGLGFLWFNAPPAAIFMGDTGSLALGGLL
str.CO21_0024  TGNAVNLTDGLDGLAIVPVMVAALSFALIAYLSGNINFADYLQIHYVSGTGELAVLLGAVVGAGLGFLWFNAPPAAIFMGDTGSLALGGLL
str.CO20_0321  TGNAVNLTDGLDGLAIVPVMVAALSFALIAYLSGNINFADYLQIHYVSGTGELAVLLGAVVGAGLGFLWFNAPPAAIFMGDTGSLALGGLL
str.RM-11      TGNAVNLTDGLDGLAIVPVMVAALSFALIAYLSGNINFADYLQIHYVSGTGELAVLLGAVVGAGLGFLWFNAPPAAIFMGDTGSLALGGLL
str.BQ2-D70    TGNAVNLTDGLDGLAIVPVMVAALSFALIAYLSGNINFADYLQIHYVSGTGELAVLLGAVVGAGLGFLWFNAPPAAIFMGDTGSLALGGLL
str.JK73rel    TGNAVNLTDGLDGLAIVPVMVAALSFALIAYLSGNINFADYLQIHYVSGTGELAVLLGAVVGAGLGFLWFNAPPAAIFMGDTGSLALGGLL
str.JK7        TGNAVNLTDGLDGLAIVPVMVAALSFALIAYLSGNINFADYLQIHYVSGTGELAVLLGAVVGAGLGFLWFNAPPAAIFMGDTGSLALGGLL
str.JK73       TGNAVNLTDGLDGLAIVPVMVAALSFALIAYLSGNINFADYLQIHYVSGTGELAVLLGAVVGAGLGFLWFNAPPAAIFMGDTGSLALGGLL
str.G1712      TGNAVNLTDGLDGLAIVPVMVAALSFALIAYLSGNINFADYLQIHYVSGTGELAVLLGAVVGAGLGFLWFNAPPAAIFMGDTGSLALGGLL

                     280       290       300       310       320       330       340       350
str.Toulouse   GIVAVATKHEIVLALIGGLFVLEGFSVVIQVGYFKLKKKRVFLMAPIHHHFEKKGWTESQVVIRFWIISIVLALVGLSTLKLR
str.JK12       GIVAVATKHEIVLALIGGLFVLEGFSVVIQVGYFKLKKKRVFLMAPIHHHFEKKGWTESQVVIRFWIISIVLALVGLSTLKLR
str.CCUG45777  GIVAVATKHEIVLALIGGLFVLEGFSVVIQVGYFKLKKKRVFLMAPIHHHFEKKGWTESQVVIRFWIISIVLALVGLSTLKLR
str.JK56       GIVAVATKHEIVLALIGGLFVLEGFSVVIQVGYFKLKKKRVFLMAPIHHHFEKKGWTESQVVIRFWIISIVLALVGLSTLKLR
str.JK67       GIVAVATKHEIVLALIGGLFVLEGFSVVIQVGYFKLKKKRVFLMAPIHHHFEKKGWTESQVVIRFWIISIVLALVGLSTLKLR
str.JK19       GIVAVATKHEIVLALIGGLFVLEGFSVVIQVGYFKLKKKRVFLMAPIHHHFEKKGWTESQVVIRFWIISIVLALVGLSTLKLR
str.JK63       GIVAVATKHEIVLALIGGLFVLEGFSVVIQVGYFKLKKKRVFLMAPIHHHFEKKGWTESQVVIRFWIISIVLALVGLSTLKLR
str.JK39       GIVAVATKHEIVLALIGGLFVLEGFSVVIQVGYFKLKKKRVFLMAPIHHHFEKKGWTESQVVIRFWIISIVLALVGLSTLKLR
str.JK31       GIVAVATKHEIVLALIGGLFVLEGFSVVIQVGYFKLKKKRVFLMAPIHHHFEKKGWTESQVVIRFWIISIVLALVGLSTLKLR
str.JK68       GIVAVATKHEIVLALIGGLFVLEGFSVVIQVGYFKLKKKRVFLMAPIHHHFEKKGWTESQVVIRFWIISIVLALVGLSTLKLR
str.G1713      GIVAVATKHEIVLALIGGLFVLEGFSVVIQVGYFKLKKKRVFLMAPIHHHFEKKGWTESQVVIRFWIISIVLALVGLSTLKLR
str.MF1-1      GIVAVATKHEIVLALIGGLFVLEGFSVVIQVGYFKLKKKRVFLMAPIHHHFEKKGWTESQVVIRFWIISIVLALVGLSTLKLR
str.NCTC12899  GIVAVATKHEIVLALIGGLFVLEGFSVVIQVGYFKLKKKRVFLMAPIHHHFEKKGWTESQVVIRFWIISIVLALVGLSTLKLR
str.CO20_0257  GIVAVATKHEIVLALIGGLFVLEGFSVVIQVGYFKLKKKRVFLMAPIHHHFEKKGWTESQVVIRFWIISIVLALVGLSTLKLR
str.CO20_0256  GIVAVATKHEIVLALIGGLFVLEGFSVVIQVGYFKLKKKRVFLMAPIHHHFEKKGWTESQVVIRFWIISIVLALVGLSTLKLR
str.CO20_0297  GIVAVATKHEIVLALIGGLFVLEGFSVVIQVGYFKLKKKRVFLMAPIHHHFEKKGWTESQVVIRFWIISIVLALVGLSTLKLR
str.CO21_0024  GIVAVATKHEIVLALIGGLFVLEGFSVVIQVGYFKLKKKRVFLMAPIHHHFEKKGWTESQVVIRFWIISIVLALVGLSTLKLR
str.CO20_0321  GIVAVATKHEIVLALIGGLFVLEGFSVVIQVGYFKLKKKRVFLMAPIHHHFEKKGWTESQVVIRFWIISIVLALVGLSTLKLR
str.RM-11      GIVAVATKHEIVLALIGGLFVLEGFSVVIQVGYFKLKKKRVFLMAPIHHHFEKKGWTESQVVIRFWIISIVLALVGLSTLKLR
str.BQ2-D70    GIVAVATKHEIVLALIGGLFVLEGFSVVIQVGYFKLKKKRVFLMAPIHHHFEKKGWTESQVVIRFWIISIVLALVGLSTLKLR
str.JK73rel    GIVAVATKHEIVLALIGGLFVLEGFSVVIQVGYFKLKKKRVFLMAPIHHHFEKKGWTESQVVIRFWIISIVLALVGLSTLKLR
str.JK7        GIVAVATKHEIVLALIGGLFVLEGFSVVIQVGYFKLKKKRVFLMAPIHHHFEKKGWTESQVVIRFWIISIVLALVGLSTLKLR
str.JK73       GIVAVATKHEIVLALIGGLFVLEGFSVVIQVGYFKLKKKRVFLMAPIHHHFEKKGWTESQVVIRFWIISIVLALVGLSTLKLR
str.G1712      GIVAVATKHEIVLALIGGLFVLEGFSVVIQVGYFKLKKKRVFLMAPIHHHFEKKGWTESQVVIRFWIISIVLALVGLSTLKLR
```

>WP_011179607.1 UDP-N-acetylmuramoyl-tripeptide - D-alanyl-D-alanine ligase [Bartonella quintana str. Toulouse]

```
                 1          10        20        30        40        50        60        70        80        90
str.Toulouse     MTALWDKQALIAAIDGFVIGCMPETFSGISIDSRTLTEGDIFFCIKGHHLDGHDFAAQAYARGAGVLIVAENRLTDMEKISAPLIVVPDVL
str.JK73rel      MTALWDKQALIAAIDGFVIGCMPETFSGISIDSRTLTEGDIFFCIKGHHLDGHDFAAQAYARGAGVLIVAENRLTDMEKISAPLIVVPDVL
str.JK73         MTALWDKQALIAAIDGFVIGCMPETFSGISIDSRTLTEGDIFFCIKGHHLDGHDFAAQAYARGAGVLIVAENRLTDMEKISAPLIVVPDVL
str.RM-11        MTALWDKQALIAAIDGFVIGCMPETFSGISIDSRTLTEGDIFFCIKGHHLDGHDFAAQAYARGAGVLIVAENRLTDMEKISAPLIVVPDVL
str.NCTC12899    MTALWDKQALIAAIDGFVIGCMPETFSGISIDSRTLTEGDIFFCIKGHHLDGHDFAAQAYARGAGVLIVAENRLTDMEKISAPLIVVPDVL
str.CO20_0257    MTALWDKQALIAAIDGFVIGCMPETFSGISIDSRTLTEGDIFFCIKGHHLDGHDFAAQAYARGAGVLIVAENRLTDMEKISAPLIVVPDVL
str.CO20_0256    MTALWDKQALIAAIDGFVIGCMPETFSGISIDSRTLTEGDIFFCIKGHHLDGHDFAAQAYARGAGVLIVAENRLTDMEKISAPLIVVPDVL
str.CO20_0297    MTALWDKQALIAAIDGFVIGCMPETFSGISIDSRTLTEGDIFFCIKGHHLDGHDFAAQAYARGAGVLIVAENRLTDMEKISAPLIVVPDVL
str.CO21_0024    MTALWDKQALIAAIDGFVIGCMPETFSGISIDSRTLTEGDIFFCIKGHHLDGHDFAAQAYARGAGVLIVAENRLTDMEKISAPLIVVPDVL
str.CO20_0321    MTALWDKQALIAAIDGFVIGCMPETFSGISIDSRTLTEGDIFFCIKGHHLDGHDFAAQAYARGAGVLIVAENRLTDMEKISAPLIVVPDVL
str.BQ2-D70      MTALWDKQALIAAIDGFVIGCMPETFSGISIDSRTLTEGDIFFCIKGHHLDGHDFAAQAYARGAGVLIVAENRLTDMEKISAPLIVVPDVL
str.JK7          MTALWDKQALIAAIDGFVIGCMPETFSGISIDSRTLTEGDIFFCIKGHHLDGHDFAAQAYARGAGVLIVAENRLTDMEKISAPLIVVPDVL
str.G1712        MTALWDKQALIAAIDGFVIGCMPETFSGISIDSRTLTEGDIFFCIKGHHLDGHDFAAQAYARGAGVLIVAENRLTDMEKISAPLIVVPDVL
str.G1713        MTALWDKQALIAAIDGFVIGCMPETFSGISIDSRTLTEGDIFFCIKGHHLDGHDFAAQAYARGAGVLIVAENRLTDMEKISAPLIVVPDVL
str.JK31         MTALWDKQALIAAIDGFVIGCMPETFSGISIDSRTLTEGDIFFCIKGHHLDGHDFAAQAYARGAGVLIVAENRLTDMEKISAPLIVVPDVL
str.JK68         MTALWDKQALIAAIDGFVIGCMPETFSGISIDSRTLTEGDIFFCIKGHHLDGHDFAAQAYARGAGVLIVAENRLTDMEKISAPLIVVPDVL
str.JK39         MTALWDKQALIAAIDGFVIGCMPETFSGISIDSRTLTEGDIFFCIKGHHLDGHDFAAQAYARGAGVLIVAENRLTDMEKISAPLIVVPDVL
str.JK12         MTALWDKQALIAAIDGFVIGCMPETFSGISIDSRTLTEGDIFFCIKGHHLDGHDFAAQAYARGAGVLIVAENRLTDMEKISAPLIVVPDVL
str.CCUG45777    MTALWDKQALIAAIDGFVIGCMPETFSGISIDSRTLTEGDIFFCIKGHHLDGHDFAAQAYARGAGVLIVAENRLTDMEKISAPLIVVPDVL
str.JK56         MTALWDKQALIAAIDGFVIGCMPETFSGISIDSRTLTEGDIFFCIKGHHLDGHDFAAQAYARGAGVLIVAENRLTDMEKISAPLIVVPDVL
str.JK67         MTALWDKQALIAAIDGFVIGCMPETFSGISIDSRTLTEGDIFFCIKGHHLDGHDFAAQAYARGAGVLIVAENRLTDMEKISAPLIVVPDVL
str.JK19         MTALWDKQALIAAIDGFVIGCMPETFSGISIDSRTLTEGDIFFCIKGHHLDGHDFAAQAYARGAGVLIVAENRLTDMEKISAPLIVVPDVL
str.JK63         MTALWDKQALIAAIDGFVIGCMPETFSGISIDSRTLTEGDIFFCIKGHHLDGHDFAAQAYARGAGVLIVAENRLTDMEKISAPLIVVPDVL
str.MF1-1        MTALWDKQALIAAIDGFVIGCMPETFSGISIDSRTLTEGDIFFCIKGHHLDGHDFAAQAYARGAGVLIVAENRLTDMEKISAPLIVVPDVL
```

```
                          100       110       120       130       140       150       160       170       180
str.Toulouse     QALEKLAQAARKRSRAKIIAITGSVGKTTTKEALKQVLATVGKVHANLASLNNCWGVPLTLARMPVESDYGIFEIGMNHADEIRPLVKLVC
str.JK73rel      QALEKLAQAARKRSRAKIIAITGSVGKTTTKEALKQVLATVGKVHANLASLNNCWGVPLTLARMPVESDYGIFEIGMNHADEIRPLVKLVC
str.JK73         QALEKLAQAARKRSRAKIIAITGSVGKTTTKEALKQVLATVGKVHANLASLNNCWGVPLTLARMPVESDYGIFEIGMNHADEIRPLVKLVC
str.RM-11        QALEKLAQAARKRSRAKIIAITGSVGKTTTKEALKQVLATVGKVHANLASLNNCWGVPLTLARMPVESDYGIFEIGMNHADEIRPLVKLVC
str.NCTC12899    QALEKLAQAARKRSRAKIIAITGSVGKTTTKEALKQVLATVGKVHANLASLNNCWGVPLTLARMPVESDYGIFEIGMNHADEIRPLVKLVC
str.CO20_0257    QALEKLAQAARKRSRAKIIAITGSVGKTTTKEALKQVLATVGKVHANLASLNNCWGVPLTLARMPVESDYGIFEIGMNHADEIRPLVKLVC
str.CO20_0256    QALEKLAQAARKRSRAKIIAITGSVGKTTTKEALKQVLATVGKVHANLASLNNCWGVPLTLARMPVESDYGIFEIGMNHADEIRPLVKLVC
str.CO20_0297    QALEKLAQAARKRSRAKIIAITGSVGKTTTKEALKQVLATVGKVHANLASLNNCWGVPLTLARMPVESDYGIFEIGMNHADEIRPLVKLVC
str.CO21_0024    QALEKLAQAARKRSRAKIIAITGSVGKTTTKEALKQVLATVGKVHANLASLNNCWGVPLTLARMPVESDYGIFEIGMNHADEIRPLVKLVC
str.CO20_0321    QALEKLAQAARKRSRAKIIAITGSVGKTTTKEALKQVLATVGKVHANLASLNNCWGVPLTLARMPVESDYGIFEIGMNHADEIRPLVKLVC
str.BQ2-D70      QALEKLAQAARKRSRAKIIAITGSVGKTTTKEALKQVLATVGKVHANLASLNNCWGVPLTLARMPVESDYGIFEIGMNHADEIRPLVKLVC
str.JK7          QALEKLAQAARKRSRAKIIAITGSVGKTTTKEALKQVLATVGKVHANLASLNNCWGVPLTLARMPVESDYGIFEIGMNHADEIRPLVKLVC
str.G1712        QALEKLAQAARKRSRAKIIAITGSVGKTTTKEALKQVLATVGKVHANLASLNNCWGVPLTLARMPVESDYGIFEIGMNHADEIRPLVKLVC
str.G1713        QALEKLAQAARKRSRAKIIAITGSVGKTTTKEALKQVLATVGKVHANLASLNNCWGVPLTLARMPVESDYGIFEIGMNHADEIRPLVKLVC
str.JK31         QALEKLAQAARKRSRAKIIAITGSVGKTTTKEALKQVLATVGKVHANLASLNNCWGVPLTLARMPVESDYGIFEIGMNHADEIRPLVKLVC
str.JK68         QALEKLAQAARKRSRAKIIAITGSVGKTTTKEALKQVLATVGKVHANLASLNNCWGVPLTLARMPVESDYGIFEIGMNHADEIRPLVKLVC
str.JK39         QALEKLAQAARKRSRAKIIAITGSVGKTTTKEALKQVLATVGKVHANLASLNNCWGVPLTLARMPVESDYGIFEIGMNHADEIRPLVKLVC
str.JK12         QALEKLAQAARKRSRAKIIAITGSVGKTTTKEALKQVLATVGKVHANLASLNNCWGVPLTLARMPVESDYGIFEIGMNHADEIRPLVKLVC
str.CCUG45777    QALEKLAQAARKRSRAKIIAITGSVGKTTTKEALKQVLATVGKVHANLASLNNCWGVPLTLARMPVESDYGIFEIGMNHADEIRPLVKLVC
str.JK56         QALEKLAQAARKRSRAKIIAITGSVGKTTTKEALKQVLATVGKVHANLASLNNCWGVPLTLARMPVESDYGIFEIGMNHADEIRPLVKLVC
str.JK67         QALEKLAQAARKRSRAKIIAITGSVGKTTTKEALKQVLATVGKVHANLASLNNCWGVPLTLARMPVESDYGIFEIGMNHADEIRPLVKLVC
str.JK19         QALEKLAQAARKRSRAKIIAITGSVGKTTTKEALKQVLATVGKVHANLASLNNCWGVPLTLARMPVESDYGIFEIGMNHADEIRPLVKLVC
str.JK63         QALEKLAQAARKRSRAKIIAITGSVGKTTTKEALKQVLATVGKVHANLASLNNCWGVPLTLARMPVESDYGIFEIGMNHADEIRPLVKLVC
str.MF1-1        QALEKLAQAARKRSRAKIIAITGSVGKTTTKEALKQVLATVGKVHANLASLNNCWGVPLTLARMPVESDYGIFEIGMNHADEIRSLVKLVC
```

```
                         190       200       210       220       230       240       250       260       270
str.Toulouse     PHVVLVTHIAAGHMGFFKNLKGIADAKAEIFEGLDEEGVALLNADNGFFSYLVRKAKQCGVKKILSFGEAENSDYQARNIRLRTDFSSMIV
str.JK73rel      PHVVLVTHIAAGHMGFFKNLKGIADAKAEIFEGLDEEGVALLNADNGFFSYLVRKAKQCGVKKILSFGEAENSDYQARNIRLRTDFSSMIV
str.JK73         PHVVLVTHIAAGHMGFFKNLKGIADAKAEIFEGLDEEGVALLNADNGFFSYLVRKAKQCGVKKILSFGEAENSDYQARNIRLRTDFSSMIV
str.RM-11        PHVVLVTHIAAGHMGFFKNLKGIADAKAEIFEGLDEEGVALLNADNGFFSYLVRKAKQCGVKKILSFGEAKNSDYQARNIRLRTDFSSMIV
str.NCTC12899    PHVVLVTHIAAGHMGFFKNLKGIADAKAEIFEGLDEEGVALLNADNGFFSYLVRKAKQCGVKKILSFGEAENSDYQARNIRLRTDFSSMIV
str.CO20_0257    PHVVLVTHIAAGHMGFFKNLKGIADAKAEIFEGLDEEGVALLNADNGFFSYLVRKAKQCGVKKILSFGEAENSDYQARNIRLRTDFSSMIV
str.CO20_0256    PHVVLVTHIAAGHMGFFKNLKGIADAKAEIFEGLDEEGVALLNADNGFFSYLVRKAKQCGVKKILSFGEAENSDYQARNIRLRTDFSSMIV
str.CO20_0297    PHVVLVTHIAAGHMGFFKNLKGIADAKAEIFEGLDEEGVALLNADNGFFSYLVRKAKQCGVKKILSFGEAENSDYQARNIRLRTDFSSMIV
str.CO21_0024    PHVVLVTHIAAGHMGFFKNLKGIADAKAEIFEGLDEEGVALLNADNGFFSYLVRKAKQCGVKKILSFGEAENSDYQARNIRLRTDFSSMIV
str.CO20_0321    PHVVLVTHIAAGHMGFFKNLKGIADAKAEIFEGLDEEGVALLNADNGFFSYLVRKAKQCGVKKILSFGEAENSDYQARNIRLRTDFSSMIV
str.BQ2-D70      PHVVLVTHIAAGHMGFFKNLKGIADAKAEIFEGLDEEGVALLNADNGFFSYLVRKAKQCGVKKILSFGEAENSDYQARNIRLRTDFSSMIV
str.JK7          PHVVLVTHIAAGHMGFFKNLKGIADAKAEIFEGLDEEGVALLNADNGFFSYLVRKAKQCGVKKILSFGEAENSDYQARNIRLRTDFSSMIV
str.G1712        PHVVLVTHIAAGHMGFFKNLKGIADAKAEIFEGLDEEGVALLNADNGFFSYLVRKAKQCGVKKILSFGEAENSDYQARNIRLRTDFSSMIV
str.G1713        PHVVLVTHIAAGHMGFFKNLKGIADAKAEIFEGLDEEGVALLNADNGFFSYLVRKAKQCGVKKILSFGEAENSDYQARNIRLRTDFSSMIV
str.JK31         PHVVLVTHIAAGHMGFFKNLKGIADAKAEIFEGLDEEGVALLNADNGFFSYLVRKAKQCGVKKILSFGEAENSDYQARNIRLRTDFSSMIV
str.JK68         PHVVLVTHIAAGHMGFFKNLKGIADAKAEIFEGLDEEGVALLNADNGFFSYLVRKAKQCGVKKILSFGEAENSDYQARNIRLRTDFSSMIV
str.JK39         PHVVLVTHIAAGHMGFFKNLKGIADAKAEIFEGLDEEGVALLNADNGFFSYLVRKAKQCGVKKILSFGEAENSDYQARNIRLRTDFSSMIV
str.JK12         PHVVLVTHIAAGHMGFFKNLKGIADAKAEIFEGLDEEGVALLNADNGFFSYLVRKAKQCGVKKILSFGEAENSDYQARNIRLRTDFSSMIV
str.CCUG45777    PHVVLVTHIAAGHMGFFKNLKGIADAKAEIFEGLDEEGVALLNADNGFFSYLVRKAKQCGVKKILSFGEAENSDYQARNIRLRTDFSSMIV
str.JK56         PHVVLVTHIAAGHMGFFKNLKGIADAKAEIFEGLDEEGVALLNADNGFFSYLVRKAKQCGVKKILSFGEAENSDYQARNIRLRTDFSSMIV
str.JK67         PHVVLVTHIAAGHMGFFKNLKGIADAKAEIFEGLDEEGVALLNADNGFFSYLVRKAKQCGVKKILSFGEAENSDYQARNIRLRTDFSSMIV
str.JK19         PHVVLVTHIAAGHMGFFKNLKGIADAKAEIFEGLDEEGVALLNADNGFFSYLVRKAKQCGVKKILSFGEAENSDYQARNIRLRTDFSSMIV
str.JK63         PHVVLVTHIAAGHMGFFKNLKGIADAKAEIFEGLDEEGVALLNADNGFFSYLVRKAKQCGVKKILSFGEAENSDYQARNIRLRTDFSSMIV
str.MF1-1        PHVVLVTHIAAGHMGFFKNLKGIADAKAEIFEGLDEEGVALLNADNGFFSYLVRKAKQCGVKKILSFGEAEKSDYQARNIRLRTDFSSMIV
```

```
                         280       290       300       310       320       330       340       350       360
str.Toulouse     HIGGQEKVIKIGAPGRHIVQNSLAVIAACDVIGIDFACVSLSLSRFSLQKGRGVRYQLFLPNGGEFHLIDESYNANPASMRAALDLLATGP
str.JK73rel      HIGGQEKVIKIGAPGRHIVQNSLAVIAACDVIGIDFACVSLSLSRFSLQKGRGVRYQLFLPNGGEFHLIDESYNANPASMRAALDLLATGP
str.JK73         HIGGQEKVIKIGAPGRHIVQNSLAVIAACDVIGIDFACVSLSLSRFSLQKGRGVRYQLFLPNGGEFHLIDESYNANPASMRAALDLLATGP
str.RM-11        HIGGQEKVIKIGAPGRHIVQNSLAVIAACDVIGIDFACVSLSLSRFSLQKGRGVRYQLFLPNGGEFHLIDESYNANPASMRAALDLLATGP
str.NCTC12899    HIGGQEKVIKIGAPGRHIVQNSLAVIAACDVIGIDFACVSLSLSRFSLQKGRGVRYQLFLPNGGEFHLIDESYNANPASMRAALDLLATGP
str.CO20_0257    HIGGQEKVIKIGAPGRHIVQNSLAVIAACDVIGIDFACVSLSLSRFSLQKGRGVRYQLFLPNGGEFHLIDESYNANPASMRAALDLLATGP
str.CO20_0256    HIGGQEKVIKIGAPGRHIVQNSLAVIAACDVIGIDFACVSLSLSRFSLQKGRGVRYQLFLPNGGEFHLIDESYNANPASMRAALDLLATGP
str.CO20_0297    HIGGQEKVIKIGAPGRHIVQNSLAVIAACDVIGIDFACVSLSLSRFSLQKGRGVRYQLFLPNGGEFHLIDESYNANPASMRAALDLLATGP
str.CO21_0024    HIGGQEKVIKIGAPGRHIVQNSLAVIAACDVIGIDFACVSLSLSRFSLQKGRGVRYQLFLPNGGEFHLIDESYNANPASMRAALDLLATGP
str.CO20_0321    HIGGQEKVIKIGAPGRHIVQNSLAVIAACDVIGIDFACVSLSLSRFSLQKGRGVRYQLFLPNGGEFHLIDESYNANPASMRAALDLLATGP
str.BQ2-D70      HIGGQEKVIKIGAPGRHIVQNSLAVIAACDVIGIDFACVSLSLSRFSLQKGRGVRYQLFLPNGGEFHLIDESYNANPASMRAALDLLATGP
str.JK7          HIGGQEKVIKIGAPGRHIVQNSLAVIAACDVIGIDFACVSLSLSRFSLQKGRGVRYQLFLPNGGEFHLIDESYNANPASMRAALDLLATGP
str.G1712        HIGGQEKVIKIGAPGRHIVQNSLAVIAACDVIGIDFACVSLSLSRFSLQKGRGVRYQLFLPNGGEFHLIDESYNANPASMRAALDLLATGP
str.G1713        HIGGQEKVIKIGAPGRHIVQNSLAVIAACDVIGIDFACVSLSLSRFSLQKGRGVRYQLFLPNGGEFHLIDESYNANPASMRAALDLLATGP
str.JK31         HIGGQEKVIKIGAPGRHIVQNSLAVIAACDVIGIDFACVSLSLSRFSLQKGRGVRYQLFLPNGGEFHLIDESYNANPASMRAALDLLATGP
str.JK68         HIGGQEKVIKIGAPGRHIVQNSLAVIAACDVIGIDFACVSLSLSRFSLQKGRGVRYQLFLPNGGEFHLIDESYNANPASMRAALDLLATGP
str.JK39         HIGGQEKVIKIGAPGRHIVQNSLAVIAACDVIGIDFACVSLSLSRFSLQKGRGVRYQLFLPNGGEFHLIDESYNANPASMRAALDLLATGP
str.JK12         HIGGQEKVIKIGAPGRHIVQNSLAVIAACDVIGIDFACVSLSLSRFSLQKGRGVRYQLFLPNGGEFHLIDESYNANPASMRAALDLLATGP
str.CCUG45777    HIGGQEKVIKIGAPGRHIVQNSLAVIAACDVIGIDFACVSLSLSRFSLQKGRGVRYQLFLPNGGEFHLIDESYNANPASMRAALDLLATGP
str.JK56         HIGGQEKVIKIGAPGRHIVQNSLAVIAACDVIGIDFACVSLSLSRFSLQKGRGVRYQLFLPNGGEFHLIDESYNANPASMRAALDLLATGP
str.JK67         HIGGQEKVIKIGAPGRHIVQNSLAVIAACDVIGIDFACVSLSLSRFSLQKGRGVRYQLFLPNGGEFHLIDESYNANPASMRAALDLLATGP
str.JK19         HIGGQEKVIKIGAPGRHIVQNSLAVIAACDVIGIDFACVSLSLSRFSLQKGRGVRYQLFLPNGGEFHLIDESYNANPASMRAALDLLATGP
str.JK63         HIGGQEKVIKIGAPGRHIVQNSLAVIAACDVIGIDFACVSLSLSRFSLQKGRGVRYQLFLPNGGEFHLIDESYNANPASMRAALDLLATGP
str.MF1-1        HIGGQEKVIKIGAPGRHIVQNSLAVIAACDVIGIDFACVSLSLSRFSLQKGRGVRYQLFLPNGGEFHLIDESYNANPASMRAALDLLATGP
```

```
                 370       380       390       400       410       420       430       440       450
str.Toulouse     VGVGGRRIALLGDMLELGAYSEKLHRDLVKPICRSSANPVFLFGEAMKFLAIDLSAYVKVYYAENIEKILPLIFAEISNGDLLMIKSSRSL
str.JK73rel      VGVGGRRIALLGDMLELGAYSEKLHRDLVKPICRSSANPVFLFGEAMKFLAIDLSAYVKVHYAENIEKILPLIFAEISNGDLLMIKSSRSL
str.JK73         VGVGGRRIALLGDMLELGAYSEKLHRDLVKPICRSSANPVFLFGEAMKFLAIDLSAYVKVHYAENIEKILPLIFAEISNGDLLMIKSSRSL
str.RM-11        VGVGGRRIALLGDMLELGAYSEKLHRDLVKPICRSSANPVFLFGEAMKFLAIDLSAYVKVHYAENIEKILPLIFAEISNGDLLMIKSSRSL
str.NCTC12899    VGVGGRRIALLGDMLELGAYSEKLHRDLVKPICRSSANPVFLFGEAMKFLAIDLSAYVKVYYAENIEKILPLIFAEISNGDLLMIKSSRSL
str.CO20_0257    VGVGGRRIALLGDMLELGAYSEKLHRDLVKPICRSSANPVFLFGEAMKFLAIDLSAYVKVYYAENIEKILPLIFAEISNGDLLMIKSSRSL
str.CO20_0256    VGVGGRRIALLGDMLELGAYSEKLHRDLVKPICRSSANPVFLFGEAMKFLAIDLSAYVKVYYAENIEKILPLIFAEISNGDLLMIKSSRSL
str.CO20_0297    VGVGGRRIALLGDMLELGAYSEKLHRDLVKPICRSSANPVFLFGEAMKFLAIDLSAYVKVYYAENIEKILPLIFAEISNGDLLMIKSSRSL
str.CO21_0024    VGVGGRRIALLGDMLELGAYSEKLHRDLVKPICRSSANPVFLFGEAMKFLAIDLSAYVKVYYAENIEKILPLIFAEISNGDLLMIKSSRSL
str.CO20_0321    VGVGGRRIALLGDMLELGAYSEKLHRDLVKPICRSSANPVFLFGEAMKFLAIDLSAYVKVYYAENIEKILPLIFAEISNGDLLMIKSSRSL
str.BQ2-D70      VGVGGRRIALLGDMLELGAYSEKLHRDLVKPICRSSANPVFLFGEAMKFLAIDLSAYVKVYYAENIEKILPLIFAEISNGDLLMIKSSRSL
str.JK7          VGVGGRRIALLGDMLELGAYSEKLHRDLVKPICRSSANPVFLFGEAMKFLAIDLSAYVKVYYAENIEKILPLIFAEISNGDLLMIKSSRSL
str.G1712        VGVGGRRIALLGDMLELGAYSEKLHRDLVKPICRSSANPVFLFGEAMKFLAIDLSAYVKVYYAENIEKILPLIFAEISNGDLLMIKSSRSL
str.G1713        VGVGGRRIALLGDMLELGAYSEKLHRDLVKPICRSSANPVFLFGEAMKFLAIDLSAYVKVYYAENIEKILPLIFAEISNGDLLMIKSSRSL
str.JK31         VGVGGRRIALLGDMLELGAYSEKLHRDLVKPICRSSANPVFLFGEAMKFLAIDLSAYVKVYYAENIEKILPLIFAEISNGDLLMIKSSRSL
str.JK68         VGVGGRRIALLGDMLELGAYSEKLHRDLVKPICRSSANPVFLFGEAMKFLAIDLSAYVKVYYAENIEKILPLIFAEISNGDLLMIKSSRSL
str.JK39         VGVGGRRIALLGDMLELGAYSEKLHRDLVKPICRSSANPVFLFGEAMKFLAIDLSAYVKVYYAENIEKILPLIFAEISNGDLLMIKSSRSL
str.JK12         VGVGGRRIALLGDMLELGAYSEKLHRDLVKPICRSSANPVFLFGEAMKFLAIDLSAYVKVYYAENIEKILPLIFAEISNGDLLMIKSSRSL
str.CCUG45777    VGVGGRRIALLGDMLELGAYSEKLHRDLVKPICRSSANPVFLFGEAMKFLAIDLSAYVKVYYAENIEKILPLIFAEISNGDLLMIKSSRSL
str.JK56         VGVGGRRIALLGDMLELGAYSEKLHRDLVKPICRSSANPVFLFGEAMKFLAIDLSAYVKVYYAENIEKILPLIFAEISNGDLLMIKSSRSL
str.JK67         VGVGGRRIALLGDMLELGAYSEKLHRDLVKPICRSSANPVFLFGEAMKFLAIDLSAYVKVYYAENIEKILPLIFAEISNGDLLMIKSSRSL
str.JK19         VGVGGRRIALLGDMLELGAYSEKLHRDLVKPICRSSANPVFLFGEAMKFLAIDLSAYVKVYYAENIEKILPLIFAEISNGDLLMIKSSRSL
str.JK63         VGVGGRRIALLGDMLELGAYSEKLHRDLVKPICRSSANPVFLFGEAMKFLAIDLSAYVKVYYAENIEKILPLIFAEISNGDLLMIKSSRSL
str.MF1-1        VGVGGRRIALLGDMLELGAYSEKLHRDLVKPICRSSANPVFLFGEAMKFLAIDLSAYVKVHYAENIEKILPLIFAEISNGDLLMIKSSRSL
```

```
                460       470
str.Toulouse     YSSDIVTALLDHYKVASL
str.JK73rel      YSSDIVTALLDHYKVASL
str.JK73         YSSDIVTALLDHYKVASL
str.RM-11        YSSDIVTALLDHYKVASL
str.NCTC12899    YSSDIVTALLDHYKVASL
str.CO20_0257    YSSDIVTALLDHYKVASL
str.CO20_0256    YSSDIVTALLDHYKVASL
str.CO20_0297    YSSDIVTALLDHYKVASL
str.CO21_0024    YSSDIVTALLDHYKVASL
str.CO20_0321    YSSDIVTALLDHYKVASL
str.BQ2-D70      YSSDIVTALLDHYKVASL
str.JK7          YSSDIVTALLDHYKVASL
str.G1712        YSSDIVTALLDHYKVASL
str.G1713        YSSDIVTALLDHYKVASL
str.JK31         YSSDIVTALLDHYKVASL
str.JK68         YSSDIVTALLDHYKVASL
str.JK39         YSSDIVTALLDHYKVASL
str.JK12         YSSDIVTALLDHYKVASL
str.CCUG45777    YSSDIVTALLDHYKVASL
str.JK56         YSSDIVTALLDHYKVASL
str.JK67         YSSDIVTALLDHYKVASL
str.JK19         YSSDIVTALLDHYKVASL
str.JK63         YSSDIVTALLDHYKVASL
str.MF1-1        YSSDIVTALLDHYKVASL
```

>WP_011179608.1 UDP-N-acetylmuramoyl-L-alanyl-D-glutamate - 2,6-diaminopimelate ligase [Bartonella quintana str. Toulouse]

```
              1        10        20        30        40        50        60        70        80        90
str.Toulouse  MLFGTVFTECIENSRLFSMEITGITADSRQVLPGYVFVALQGNQDDGRHYINDAIKRGAQAIVTDCNAVLENFSVPVLRVLDVRHSLAIAA
str.NCTC12899 MLFGTVFTECIENSRLFSMEITGITADSRQVLPGYVFVALQGNQDDGRHYINDAIKRGAQAIVTDCNAVLENFSVPVLRVLDVRHSLAIAA
str.CO20_0257 MLFGTVFTECIENSRLFSMEITGITADSRQVLPGYVFVALQGNQDDGRHYINDAIKRGAQAIVTDCNAVLENFSVPVLRVLDVRHSLAIAA
str.CO20_0256 MLFGTVFTECIENSRLFSMEITGITADSRQVLPGYVFVALQGNQDDGRHYINDAIKRGAQAIVTDCNAVLENFSVPVLRVLDVRHSLAIAA
str.CO20_0297 MLFGTVFTECIENSRLFSMEITGITADSRQVLPGYVFVALQGNQDDGRHYINDAIKRGAQAIVTDCNAVLENFSVPVLRVLDVRHSLAIAA
str.CO21_0024 MLFGTVFTECIENSRLFSMEITGITADSRQVLPGYVFVALQGNQDDGRHYINDAIKRGAQAIVTDCNAVLENFSVPVLRVLDVRHSLAIAA
str.CO20_0321 MLFGTVFTECIENSRLFSMEITGITADSRQVLPGYVFVALQGNQDDGRHYINDAIKRGAQAIVTDCNAVLENFSVPVLRVLDVRHSLAIAA
str.G1712     MLFGTVFTECIENSRLFSMEITGITADSRQVLPGYVFVALQGNQDDGRHYINDAIKRGAQAIVTDCNAVLENFSVPVLRVLDVRHSLAIAA
str.G1713     MLFGTVFTECIENSRLFSMEITGITADSRQVLPGYVFVALQGNQDDGRHYINDAIKRGAQAIVTDCNAVLENFSVPVLRVLDVRHSLAIAA
str.CCUG45777 MLFGTVFTECIENSRLFSMEITGITADSRQVLPGYVFVALQGNQDDGRHYINDAIKRGAQAIVTDCNAVLENFSVPVLRVLDVRHSLAIAA
str.JK67      MLFGTVFTECIENSRLFSMEITGITADSRQVLPGYVFVALQGNQDDGRHYINDAIKRGAQAIVTDCNAVLENFSVPVLRVLDVRHSLAIAA
str.JK56      MLFGTVFTECIENSRLFSMEITGITADSRQVLPGYVFVALQGNQDDGRHYINDAIKRGAQAIVTDCNAVLENFSVPVLRVLDVRHSLAIAA
str.JK63      MLFGTVFTECIENSRLFSMEITGITADSRQVLPGYVFVALQGNQDDGRHYINDAIKRGAQAIVTDCNAVLENFSVPVLRVLDVRHSLAIAA
str.JK68      MLFGTVFTECIENSRLFSMEITGITADSRQVLPGYVFVALQGNQDDGRHYINDAIKRGAQAIVTDCNAVLENFSVPVLRVLDVRHSLAIAA
str.JK31      MLFGTVFTECIENSRLFSMEITGITADSRQVLPGYVFVALQGNQDDGRHYINDAIKRGAQAIVTDCNAVLENFSVPVLRVLDVRHSLAIAA
str.BQ2-D70   MLFGTVFTECIENSRLFSMEITGITADSRQVLPGYVFVALQGNQDDGRHYINDAIKRGAQAIVTDCNAVLENFSVPVLRVLDVRHSLAIAA
str.JK12      MLFGTVFTECIENSRLFSMEITGITADSRQVLPGYVFVALQGNQDDGRHYINDAIKRGAQAIVTDCNAVLENFSVPVLRVLDVRHSLAIAA
str.JK19      MLFGTVFTECIENSRLFSMEITGITADSRQVLPGYVFVALQGNQDDGRHYINDAIKRGAQAIVTDCNAVLENFSVPVLRVLDVRHSLAIAA
str.JK39      MLFGTVFTECIENSRLFSMEITGITADSRQVLPGYVFVALQGNQDDGRHYINDAIKRGAQAIVTDCNAVLENFSVPVLRVLDVRHSLAIAA
str.JK73      MLFGTVFTECIENSRLFSMEITGITADSRQVLPGYVFVALQGNQDDGRHYINDAIKRGAQAIVTDCNAVLENFSVPVLRVLDVRHSLAIAA
str.JK7       MLFGTVFTECIENSRLFSMEITGITADSRQVLPGYVFVALQGNQDDGRHYINDAIKRGAQAIVTDCNAVLENFSVPVLRVLDVRHSLAIAA
str.JK73rel   MLFGTVFTECIENSRLFSMEITGITADSRQVLPGYVFVALQGNQDDGRHYINDAIKRGAQAIVTDCNAVLENFSVPVLRVLDVRHSLAIAA
str.RM-11     MLFGTVFTECIENSRLFSMEITGITADSRQVLPGYVFVALQGNQDDGRHYINDAIKRGAQAIITDCNAVLENFSVPVLHVLDVRHSLAIAA
str.MF1-1     MLFGTVFTECIENSRLFSMEITGITADSRQVLPGYVFVALQGNQDDGRHYINDAIKRGAQAIVTDCNAVLENFSVPVLHVLDVRHSLAIAA
```

```
                     100       110       120       130       140       150       160       170       180
str.Toulouse  ARFYGSQPEIVVAVTGTSGKTSVVSFLRQIWTHVGLCAASIGTVGIVSPHRNDSCSFTTPDPVVLQRLLYEMSNEGVTHAALEASSHGLDQ
str.NCTC12899 ARFYGSQPEIVVAVTGTSGKTSVVSFLRQIWTHVGLCAASIGTVGIVSPHRNDSCSFTTPDPVVLQRLLYEMSNEGVTHAALEASSHGLDQ
str.CO20_0257 ARFYGSQPEIVVAVTGTSGKTSVVSFLRQIWTHVGLCAASIGTVGIVSPHRNDSCSFTTPDPVVLQRLLYEMSNEGVTHAALEASSHGLDQ
str.CO20_0256 ARFYGSQPEIVVAVTGTSGKTSVVSFLRQIWTHVGLCAASIGTVGIVSPHRNDSCSFTTPDPVVLQRLLYEMSNEGVTHAALEASSHGLDQ
str.CO20_0297 ARFYGSQPEIVVAVTGTSGKTSVVSFLRQIWTHVGLCAASIGTVGIVSPHRNDSCSFTTPDPVVLQRLLYEMSNEGVTHAALEASSHGLDQ
str.CO21_0024 ARFYGSQPEIVVAVTGTSGKTSVVSFLRQIWTHVGLCAASIGTVGIVSPHRNDSCSFTTPDPVVLQRLLYEMSNEGVTHAALEASSHGLDQ
str.CO20_0321 ARFYGSQPEIVVAVTGTSGKTSVVSFLRQIWTHVGLCAASIGTVGIVSPHRNDSCSFTTPDPVVLQRLLYEMSNEGVTHAALEASSHGLDQ
str.G1712     ARFYGSQPEIVVAVTGTSGKTSVVSFLRQIWTHVGLCAASIGTVGIVSPHRNDSCSFTTPDPVVLQRLLYEMSNEGVTHAALEASSHGLDQ
str.G1713     ARFYGSQPEIVVAVTGTSGKTSVVSFLRQIWTHVGLCAASIGTVGIVSPHRNDSCSFTTPDPVVLQRLLYEMSNEGVTHAALEASSHGLDQ
str.CCUG45777 ARFYGSQPEIVVAVTGTSGKTSVVSFLRQIWTHVGLCAASIGTVGIVSPHRNDSCSFTTPDPVVLQRLLYEMSNEGVTHAALEASSHGLDQ
str.JK67      ARFYGSQPEIVVAVTGTSGKTSVVSFLRQIWTHVGLCAASIGTVGIVSPHRNDSCSFTTPDPVVLQRLLYEMSNEGVTHAALEASSHGLDQ
str.JK56      ARFYGSQPEIVVAVTGTSGKTSVVSFLRQIWTHVGLCAASIGTVGIVSPHRNDSCSFTTPDPVVLQRLLYEMSNEGVTHAALEASSHGLDQ
str.JK63      ARFYGSQPEIVVAVTGTSGKTSVVSFLRQIWTHVGLCAASIGTVGIVSPHRNDSCSFTTPDPVVLQRLLYEMSNEGVTHAALEASSHGLDQ
str.JK68      ARFYGSQPEIVVAVTGTSGKTSVVSFLRQIWTHVGLCAASIGTVGIVSPHRNDSCSFTTPDPVVLQRLLYEMSNEGVTHAALEASSHGLDQ
str.JK31      ARFYGSQPEIVVAVTGTSGKTSVVSFLRQIWTHVGLCAASIGTVGIVSPHRNDSCSFTTPDPVVLQRLLYEMSNEGVTHAALEASSHGLDQ
str.BQ2-D70   ARFYGSQPEIVVAVTGTSGKTSVVSFLRQIWTHVGLCAASIGTVGIVSPHRNDSCSFTTPDPVVLQRLLYEMSNEGVTHAALEASSHGLDQ
str.JK12      ARFYGSQPEIVVAVTGTSGKTSVVSFLRQIWTHVGLCAASIGTVGIVSPHRNDSCSFTTPDPVVLQRLLYEMSNEGVTHAALEASSHGLDQ
str.JK19      ARFYGSQPEIVVAVTGTSGKTSVVSFLRQIWTHVGLCAASIGTVGIVSPHRNDSCSFTTPDPVVLQRLLYEMSNEGVTHAALEASSHGLDQ
str.JK39      ARFYGSQPEIVVAVTGTSGKTSVVSFLRQIWTHVGLCAASIGTVGIVSPHRNDSCSFTTPDPVVLQRLLYEMSNEGVTHAALEASSHGLDQ
str.JK73      ARFYGSQPEIVVAVTGTSGKTSVVSFLRQIWTHVGLCAASIGTVGIVSPHRNDSCSFTTPDPVVLQRLLYEMSNEGVTHAALEASSHGLDQ
str.JK7       ARFYGSQPEIVVAVTGTSGKTSVVSFLRQIWTHVGLCAASIGTVGIVSPHRNDSCSFTTPDPVVLQRLLYEMSNEGVTHAALEASSHGLDQ
str.JK73rel   ARFYGSQPEIVVAVTGTSGKTSVVSFLRQIWTHVGLCAASIGTVGIVSPHRNDSCSFTTPDPVVLQRLLYEMSNEGVTHAALEASSHGLDQ
str.RM-11     ARFYGSQPEIVVAVTGTSGKTSVVSFLRQIWTHVGLCAASIGTVGIVSPHRNDSCSFTTPDPVVLQRLLYEMSNEGVTHAALEASSHGLDQ
str.MF1-1     ARFYGSQPEIVVAVTGTSGKTSVVSFLRQIWTHVGLCAASIGTVGIVSPHRNDSCSFTTPDPVVLQRLLYEMSNEGVTHAALEASSHGLDQ
```

```
                    190       200       210       220       230       240       250       260       270
str.Toulouse  SRLDGVRLTAAAFTNLGRDHMDYHKDVEDYLQAKMRLFDTLLPQDAPALVFADDIYSQKVIDIVTKARRRILTIGRKGKFITINRIEHQRL
str.NCTC12899 SRLDGVRLTAAAFTNLGRDHMDYHKDVEDYLQAKMRLFDTLLPQDAPALVFADDIYSQKVIDIVTKARRRILTIGRKGKFITINRIEHQRL
str.CO20_0257 SRLDGVRLTAAAFTNLGRDHMDYHKDVEDYLQAKMRLFDTLLPQDAPALVFADDIYSQKVIDIVTKARRRILTIGRKGKFITINRIEHQRL
str.CO20_0256 SRLDGVRLTAAAFTNLGRDHMDYHKDVEDYLQAKMRLFDTLLPQDAPALVFADDIYSQKVIDIVTKARRRILTIGRKGKFITINRIEHQRL
str.CO20_0297 SRLDGVRLTAAAFTNLGRDHMDYHKDVEDYLQAKMRLFDTLLPQDAPALVFADDIYSQKVIDIVTKARRRILTIGRKGKFITINRIEHQRL
str.CO21_0024 SRLDGVRLTAAAFTNLGRDHMDYHKDVEDYLQAKMRLFDTLLPQDAPALVFADDIYSQKVIDIVTKARRRILTIGRKGKFITINRIEHQRL
str.CO20_0321 SRLDGVRLTAAAFTNLGRDHMDYHKDVEDYLQAKMRLFDTLLPQDAPALVFADDIYSQKVIDIVTKARRRILTIGRKGKFITINRIEHQRL
str.G1712     SRLDGVRLTAAAFTNLGRDHMDYHKDVEDYLQAKMRLFDTLLPQDAPALVFADDIYSQKVIDIVTKARRRILTIGRKGKFITINRIEHQRL
str.G1713     SRLDGVRLTAAAFTNLGRDHMDYHKDVEDYLQAKMRLFDTLLPQDAPALVFADDIYSQKVIDIVTKARRRILTIGRKGKFITINRIEHQRL
str.CCUG45777 SRLDGVRLTAAAFTNLGRDHMDYHKDVEDYLQAKMRLFDTLLPQDAPALVFADDIYSQKVIDIVTKARRRILTIGRKGKFITINRIEHQRL
str.JK67      SRLDGVRLTAAAFTNLGRDHMDYHKDVEDYLQAKMRLFDTLLPQDAPALVFADDIYSQKVIDIVTKARRRILTIGRKGKFITINRIEHQRL
str.JK56      SRLDGVRLTAAAFTNLGRDHMDYHKDVEDYLQAKMRLFDTLLPQDAPALVFADDIYSQKVIDIVTKARRRILTIGRKGKFITINRIEHQRL
str.JK63      SRLDGVRLTAAAFTNLGRDHMDYHKDVEDYLQAKMRLFDTLLPQDAPALVFADDIYSQKVIDIVTKARRRILTIGRKGKFITINRIEHQRL
str.JK68      SRLDGVRLTAAAFTNLGRDHMDYHKDVEDYLQAKMRLFDTLLPQDAPALVFADDIYSQKVIDIVTKARRRILTIGRKGKFITINRIEHQRL
str.JK31      SRLDGVRLTAAAFTNLGRDHMDYHKDVEDYLQAKMRLFDTLLPQDAPALVFADDIYSQKVIDIVTKARRRILTIGRKGKFITINRIEHQRL
str.BQ2-D70   SRLDGVRLTAAAFTNLGRDHMDYHKDVEDYLQAKMRLFDTLLPQDAPALVFADDIYSQKVIDIVTKARRRILTIGRKGKFITINRIEHQRL
str.JK12      SRLDGVRLTAAAFTNLGRDHMDYHKDVEDYLQAKMRLFDTLLPQDAPALVFADDIYSQKVIDIVTKARRRILTIGRKGKFITINRIEHQRL
str.JK19      SRLDGVRLTAAAFTNLGRDHMDYHKDVEDYLQAKMRLFDTLLPQDAPALVFADDIYSQKVIDIVTKARRRILTIGRKGKFITINRIEHQRL
str.JK39      SRLDGVRLTAAAFTNLGRDHMDYHKDVEDYLQAKMRLFDTLLPQDAPALVFADDIYSQKVIDIVTKARRRILTIGRKGKFITINRIEHQRL
str.JK73      SRLDGVRLTAAAFTNLGRDHMDYHKDVEDYLQAKMRLFDTLLPQDAPALVFADDIYSQKVIDIVTKARRRILTIGRKGKFITINRIEHQRL
str.JK7       SRLDGVRLTAAAFTNLGRDHMDYHKDVEDYLQAKMRLFDTLLPQDAPALVFADDIYSQKVIDIVTKARRRILTIGRKGKFITINRIEHQRL
str.JK73rel   SRLDGVRLTAAAFTNLGRDHMDYHKDVEDYLQAKMRLFDTLLPQDAPALVFADDIYSQKVIDIVTKARRRILTIGRKGKFITINRIEHQRL
str.RM-11     SRLDGVRLTAAAFTNLGRDHMDYHKDVEDYLQAKMRLFDTLLPQDAPALVFADDIYSQKVIDIVTKARRRILTIGRKGKFITINRIEHQRL
str.MF1-1     SRLDGVRLTAAAFTNLGRDHMDYHKDVEDYLQAKMRLFDTLLPQDAPALVFADDIYSQKVIDIVTKARRRILTIGRKGKFITINRIEHQRL
```

```
                   280       290       300       310       320       330       340       350       360
str.Toulouse  KQCVECRVENNIYTFDLPLAGNFQVTNALMAAGLAIATGVSPKKVFHSLEILQGAPGRLELVGKTENNAPVYIDYAHKPEALEQVLLSVRP
str.NCTC12899 KQCVECRVENNIYTFDLPLAGNFQVTNALMAAGLAIATGVSPKKVFHSLEILQGAPGRLELVGKTENNAPVYIDYAHKPEALEQVLLSVRP
str.CO20_0257 KQCVECRVENNIYTFDLPLAGNFQVTNALMAAGLAIATGVSPKKVFHSLEILQGAPGRLELVGKTENNAPVYIDYAHKPEALEQVLLSVRP
str.CO20_0256 KQCVECRVENNIYTFDLPLAGNFQVTNALMAAGLAIATGVSPKKVFHSLEILQGAPGRLELVGKTENNAPVYIDYAHKPEALEQVLLSVRP
str.CO20_0297 KQCVECRVENNIYTFDLPLAGNFQVTNALMAAGLAIATGVSPKKVFHSLEILQGAPGRLELVGKTENNAPVYIDYAHKPEALEQVLLSVRP
str.CO21_0024 KQCVECRVENNIYTFDLPLAGNFQVTNALMAAGLAIATGVSPKKVFHSLEILQGAPGRLELVGKTENNAPVYIDYAHKPEALEQVLLSVRP
str.CO20_0321 KQCVECRVENNIYTFDLPLAGNFQVTNALMAAGLAIATGVSPKKVFHSLEILQGAPGRLELVGKTENNAPVYIDYAHKPEALEQVLLSVRP
str.G1712     KQCVECRVENNIYTFDLPLAGNFQVTNALMAAGLAIATGVSPKKVFHSLEILQGAPGRLELVGKTENNAPVYIDYAHKPEALEQVLLSVRP
str.G1713     KQCVECRVENNIYTFDLPLAGNFQVTNALMAAGLAIATGVSPKKVFHSLEILQGAPGRLELVGKTENNAPVYIDYAHKPEALEQVLLSVRP
str.CCUG45777 KQCVECRVENNIYTFDLPLAGNFQVTNALMAAGLAIATGVSPKKVFHSLEILQGAPGRLELVGKTENNAPVYIDYAHKPEALEQVLLSVRP
str.JK67      KQCVECRVENNIYTFDLPLAGNFQVTNALMAAGLAIATGISPKKVFHSLEILQGAPGRLELVGKTENNAPVYIDYAHKPEALEQVLLSVRP
str.JK56      KQCVECRVENNIYTFDLPLAGNFQVTNALMAAGLAIATGISPKKVFHSLEILQGAPGRLELVGKTENNAPVYIDYAHKPEALEQVLLSVRP
str.JK63      KQCVECRVENNIYTFDLPLAGNFQVTNALMAAGLAIATGISPKKVFHSLEILQGAPGRLELVGKTENNAPVYIDYAHKPEALEQVLLSVRP
str.JK68      KQCVECRVENNIYTFDLPLAGNFQVTNALMAAGLAIATGISPKKVFHSLEILQGAPGRLELVGKTENNAPVYIDYAHKPEALEQVLLSVRP
str.JK31      KQCVECRVENNIYTFDLPLAGNFQVTNALMAAGLAIATGISPKKVFHSLEILQGAPGRLELVGKTENNAPVYIDYAHKPEALEQVLLSVRP
str.BQ2-D70   KQCVECRVENNIYTFDLPLAGNFQVTNALMAAGLAIATGISPKKVFHSLEILQGAPGRLELVGKTENNAPVYIDYAHKPEALEQVLLSVRP
str.JK12      KQCVECRVENNIYTFDLPLAGNFQVTNALMAAGLAIATGISPKKVFHSLEILQGAPGRLELVGKTENNAPVYIDYAHKPEALEQVLLSVRP
str.JK19      KQCVECRVENNIYTFDLPLAGNFQVTNALMAAGLAIATGISPKKVFHSLEILQGAPGRLELVGKTENNAPVYIDYAHKPEALEQVLLSVRP
str.JK39      KQCVECRVENNIYTFDLPLAGNFQVTNALMAAGLAIATGISPKKVFHSLEILQGAPGRLELVGKTENNAPVYIDYAHKPEALEQVLLSVRP
str.JK73      KQCVECRVENNIYTFDLPLAGNFQVTNALMAAGLAIATGISPKKVFHSLEILQGAPGRLELVGKTENNAPVYIDYAHKPEALEQVLLSVRP
str.JK7       KQCVECRVENNIYTFDLPLAGNFQVTNALMAAGLAIATGISPKKVFHSLEILQGAPGRLELVGKTENNAPVYIDYAHKPEALEQVLLSVRP
str.JK73rel   KQCVECRVENNIYTFDLPLAGNFQVTNALMAAGLAIATGISPKKVFHSLEILQGAPGRLELVGKTENNAPVYIDYAHKPEALEQVLLSVRP
str.RM-11     KQCVECRVENNIYTFDLPLAGNFQVTNALMAAGLAIATGISPKKVFHSLEILQGAPGRLELVGKTENNAPVYIDYAHKPEALEQVLLSVRP
str.MF1-1     KQCVECRVENNIYTFDLPLAGNFQVTNALMAAGLAIATGISPKKVFHSLEVLQGAPGRLELVGKTENNAPVYIDYAHKPEALEQVLLSVRP
```

```
                    370       380       390       400       410       420       430       440       450
str.Toulouse    FTQGRLILVFGCGGDRDQGKRPLMGKIAENKADIVIVTDDNPRTEMPEKIRKDILQAAPRAIEIADRGEAISYAVGLLKAEDTLIIAGKGH
str.NCTC12899   FTQGRLILVFGCGGDRDQGKRPLMGKIAENKADIVIVTDDNPRTEMPEKIRKDILQAAPRAIEIADRGEAISYAVGLLKAEDTLIIAGKGH
str.CO20_0257   FTQGRLILVFGCGGDRDQGKRPLMGKIAENKADIVIVTDDNPRTEMPEKIRKDILQAAPRAIEIADRGEAISYAVGLLKAEDTLIIAGKGH
str.CO20_0256   FTQGRLILVFGCGGDRDQGKRPLMGKIAENKADIVIVTDDNPRTEMPEKIRKDILQAAPRAIEIADRGEAISYAVGLLKAEDTLIIAGKGH
str.CO20_0297   FTQGRLILVFGCGGDRDQGKRPLMGKIAENKADIVIVTDDNPRTEMPEKIRKDILQAAPRAIEIADRGEAISYAVGLLKAEDTLIIAGKGH
str.CO21_0024   FTQGRLILVFGCGGDRDQGKRPLMGKIAENKADIVIVTDDNPRTEMPEKIRKDILQAAPRAIEIADRGEAISYAVGLLKAEDTLIIAGKGH
str.CO20_0321   FTQGRLILVFGCGGDRDQGKRPLMGKIAENKADIVIVTDDNPRTEMPEKIRKDILQAAPRAIEIADRGEAISYAVGLLKAEDTLIIAGKGH
str.G1712       FTQGRLILVFGCGGDRDQGKRPLMGKIAENKADIVIVTDDNPRTEMPEKIRKDILQAAPRAIEIADRGEAISYAVGLLKAEDTLIIAGKGH
str.G1713       FTQGRLILVFGCGGDRDQGKRPLMGKIAENKADIVIVTDDNPRTEMPEKIRKDILQAAPRAIEIADRGEAISYAVGLLKAEDTLIIAGKGH
str.CCUG45777   FTQGRLILVFGCGGDRDQGKRPLMGKIAENKADIVIVTDDNPRTEMPEKIRKDILQAAPRAIEIADRGEAISYAVGLLKAEDTLIIAGKGH
str.JK67        FTQGRLILVFGCGGDRDQGKRPLMGKIAENKADIVIVTDDNPRTEMPEKIRKDILQAAPRAIEIADRGEAISYAVGLLKAEDTLIIAGKGH
str.JK56        FTQGRLILVFGCGGDRDQGKRPLMGKIAENKADIVIVTDDNPRTEMPEKIRKDILQAAPRAIEIADRGEAISYAVGLLKAEDTLIIAGKGH
str.JK63        FTQGRLILVFGCGGDRDQGKRPLMGKIAENKADIVIVTDDNPRTEMPEKIRKDILQAAPRAIEIADRGEAISYAVGLLKAEDTLIIAGKGH
str.JK68        FTQGRLILVFGCGGDRDQGKRPLMGKIAENKADIVIVTDDNPRTEMPEKIRKDILQAAPRAIEIADRGEAISYAVGLLKAEDTLIIAGKGH
str.JK31        FTQGRLILVFGCGGDRDQGKRPLMGKIAENKADIVIVTDDNPRTEMPEKIRKDILQAAPRAIEIADRGEAISYAVGLLKAEDTLIIAGKGH
str.BQ2-D70     FTQGRLILVFGCGGDRDQGKRPLMGKIAENKADIVIVTDDNPRTEMPEKIRKDILQAAPRAIEIADRGEAISYAVGLLKAEDTLIIAGKGH
str.JK12        FTQGRLILVFGCGGDRDQGKRPLMGKIAENKADIVIVTDDNPRTEMPEKIRKDILQAAPRAIEIADRGEAISYAVGLLKAEDTLIIAGKGH
str.JK19        FTQGRLILVFGCGGDRDQGKRPLMGKIAENKADIVIVTDDNPRTEMPEKIRKDILQAAPRAIEIADRGEAISYAVGLLKAEDTLIIAGKGH
str.JK39        FTQGRLILVFGCGGDRDQGKRPLMGKIAENKADIVIVTDDNPRTEMPEKIRKDILQAAPRAIEIADRGEAISYAVGLLKAEDTLIIAGKGH
str.JK73        FTQGRLILVFGCGGDRDQGKRPLMGKIAENKADIVIVTDDNPRTEMPEKIRKDILQAAPRAIEIADRGEAISYAVGLLKAEDTLIIAGKGH
str.JK7         FTQGRLILVFGCGGDRDQGKRPLMGKIAENKADIVIVTDDNPRTEMPEKIRKDILQAAPRAIEIADRGEAISYAVGLLKAEDTLIIAGKGH
str.JK73rel     FTQGRLILVFGCGGDRDQGKRPLMGKIAENKADIVIVTDDNPRTEMPEKIRKDILQAAPRAIEIADRGEAISYAVGLLKAEDTLIIAGKGH
str.RM-11       FTQGRLILVFGCGGDRDQGKRPLMGKIAENKADIVIVTDDNPRTEMPEKIRKDILQAAPRAIEIADRGEAISYAVGLLKAEDTLIIAGKGH
str.MF1-1       FTQGRLILVFGCGGDRDQGKRPLMGKIAENKADIVIVTDDNPRTEMPEKIRKDILQAAPRAIEIADRGEAISYAVGLLKAEDTLIIAGKGH

                  460       470       480
str.Toulouse    ENGQVIGKKTYPFSDRLKVIDALQERMR
str.NCTC12899   ENGQVIGKKTYPFSDRLKVIDALQERMR
str.CO20_0257   ENGQVIGKKTYPFSDRLKVIDALQERMR
str.CO20_0256   ENGQVIGKKTYPFSDRLKVIDALQERMR
str.CO20_0297   ENGQVIGKKTYPFSDRLKVIDALQERMR
str.CO21_0024   ENGQVIGKKTYPFSDRLKVIDALQERMR
str.CO20_0321   ENGQVIGKKTYPFSDRLKVIDALQERMR
str.G1712       ENGQVIGKKTYPFSDRLKVIDALQERMR
str.G1713       ENGQVIGKKTYPFSDRLKVIDALQERMR
str.CCUG45777   ENGQVIGKKTYPFSDRLKVIDALQERMR
str.JK67        ENGQVIGKKTYPFSDRLKVIDALQERMR
str.JK56        ENGQVIGKKTYPFSDRLKVIDALQERMR
str.JK63        ENGQVIGKKTYPFSDRLKVIDALQERMR
str.JK68        ENGQVIGKKTYPFSDRLKVIDALQERMR
str.JK31        ENGQVIGKKTYPFSDRLKVIDALQERMR
str.BQ2-D70     ENGQVIGKKTYPFSDRLKVIDALQERMR
str.JK12        ENGQVIGKKTYPFSDRLKVIDALQERMR
str.JK19        ENGQVIGKKTYPFSDRLKVIDALQERMR
str.JK39        ENGQVIGKKTYPFSDRLKVIDALQERMR
str.JK73        ENGQVIGKKTYPFSDRLKVIDALQERMR
str.JK7         ENGQVIGKKTYPFSDRLKVIDALQERMR
str.JK73rel     ENGQVIGKKTYPFSDRLKVIDALQERMR
str.RM-11       ENGQVIGKKTYPFSDRLKVIDALQERMR
str.MF1-1       ENGQVIGKKTYPFSDRLKAIDSLQERMR
```

>WP_011179609.1 penicillin-binding protein 2 [Bartonella quintana str. Toulouse]

```
               1         10         20         30         40         50         60         70         80         90
str.Toulouse   MKSLFLFSQKKKRLNNQLDFHNFSIRRSYSARPRLLFSLLCFLILYGVIGACLISYGLEGGQIEEAKGPGVLQLTARPDIIDRNGRLLATD
str.BQ2-D70    MKSLFLFSQKKKRLNNQLDFHNFSIRRSYSARPRLLFSLLCFLILYGVIGACLISYGLEGGQIEEAKGPGVLQLTARPDIIDRNGRLLATD
str.JK31       MKSLFLFSQKKKRLNNQLDFHNFSIRRSYSARPRLLFSLLCFLILYGVIGACLISYGLEGGQIEEAKGPGVLQLTARPDIIDRNGRLLATD
str.JK68       MKSLFLFSQKKKRLNNQLDFHNFSIRRSYSARPRLLFSLLCFLILYGVIGACLISYGLEGGQIEEAKGPGVLQLTARPDIIDRNGRLLATD
str.JK63       MKSLFLFSQKKKRLNNQLDFHNFSIRRSYSARPRLLFSLLCFLILYGVIGACLISYGLEGGQIEEAKGPGVLQLTARPDIIDRNGRLLATD
str.JK67       MKSLFLFSQKKKRLNNQLDFHNFSIRRSYSARPRLLFSLLCFLILYGVIGACLISYGLEGGQIEEAKGPGVLQLTARPDIIDRNGRLLATD
str.JK56       MKSLFLFSQKKKRLNNQLDFHNFSIRRSYSARPRLLFSLLCFLILYGVIGACLISYGLEGGQIEEAKGPGVLQLTARPDIIDRNGRLLATD
str.CO20_0321  MKSLFLFSQKKKRLNNQLDFHNFSIRRSYSARPRLLFSLLCFLILYGVIGACLISYGLEGGQIEEAKGPGVLQLTARPDIIDRNGRLLATD
str.JK73       MKSLFLFSQKKKRLNNQLDFHNFSIRRSYSARPRLLFSLLCFLILYGVIGACLISYGLEGGQIEEAKGPGVLQLTARPDIIDRNGRLLATD
str.JK39       MKSLFLFSQKKKRLNNQLDFHNFSIRRSYSARPRLLFSLLCFLILYGVIGACLISYGLEGGQIEEAKGPGVLQLTARPDIIDRNGRLLATD
str.CO20_0257  MKSLFLFSQKKKRLNNQLDFHNFSIRRSYSARPRLLFSLLCFLILYGVIGACLISYGLEGGQIEEAKGPGVLQLTARPDIIDRNGRLLATD
str.CO20_0256  MKSLFLFSQKKKRLNNQLDFHNFSIRRSYSARPRLLFSLLCFLILYGVIGACLISYGLEGGQIEEAKGPGVLQLTARPDIIDRNGRLLATD
str.CO20_0297  MKSLFLFSQKKKRLNNQLDFHNFSIRRSYSARPRLLFSLLCFLILYGVIGACLISYGLEGGQIEEAKGPGVLQLTARPDIIDRNGRLLATD
str.CO21_0024  MKSLFLFSQKKKRLNNQLDFHNFSIRRSYSARPRLLFSLLCFLILYGVIGACLISYGLEGGQIEEAKGPGVLQLTARPDIIDRNGRLLATD
str.NCTC12899  MKSLFLFSQKKKRLNNQLDFHNFSIRRSYSARPRLLFSLLCFLILYGVIGACLISYGLEGGQIEEAKGPGVLQLTARPDIIDRNGRLLATD
str.JK7        MKSLFLFSQKKKRLNNQLDFHNFSIRRSYSARPRLLFSLLCFLILYGVIGACLISYGLEGGQIEEAKGPGVLQLTARPDIIDRNGRLLATD
str.G1712      MKSLFLFSQKKKRLNNQLDFHNFSIRRSYSARPRLLFSLLCFLILYGVIGACLISYGLEGGQIEEAKGPGVLQLTARPDIIDRNGRLLATD
str.JK12       MKSLFLFSQKKKRLNNQLDFHNFSIRRSYSARPRLLFSLLCFLILYGVIGACLISYGLEGGQIEEAKGPGVLQLTARPDIIDRNGRLLATD
str.CCUG45777  MKSLFLFSQKKKRLNNQLDFHNFSIRRSYSARPRLLFSLLCFLILYGVIGACLISYGLEGGQIEEAKGPGVLQLTARPDIIDRNGRLLATD
str.JK19       MKSLFLFSQKKKRLNNQLDFHNFSIRRSYSARPRLLFSLLCFLILYGVIGACLISYGLEGGQIEEAKGPGVLQLTARPDIIDRNGRLLATD
str.G1713      MKSLFLFSQKKKRLNNQLDFHNFSIRRSYSARPRLLFSLLCFLILYGVIGACLISYGLEGGQIEEAKGPGVLQLTARPDIIDRNGRLLATD
str.JK73rel    MKSLFLFSQKKKRLNNQLDFHNFSIRRSYSARPRLLFSLLCFLILYGVIGACLISYGLEGGQIEEAKGPGVLQLTARPDIIDRNGRLLATD
str.RM-11      MKSFFLFSQKKKRLNNQLDFHNFSIRRSYSARPRLLFSLLCFLILYGVIGACLISYGLEGGQIEEAKGPGVLQLTARPDIIDRNGRLLATD
str.MF1-1      MKSFFLFSQKKKRLNNQLDFRNFSIRRSYSARPRLLFSLLCFLILYGVIGACLISYGLEGGQIEEAKGPGVLQLTARPDIIDRNGRLLATD
```

```
                     100       110       120       130       140       150       160       170       180
str.Toulouse   IKTYSLFAEPRRVIDVDETIELLSTVLSDLNWHETYKRLKRKSGFSWIQRGLTPTQKAQIMALGIPGIGFRPEIRRFYPGGSVASHILGMV
str.BQ2-D70    IKTYSLFAEPRRVIDVDETIELLSTVLSDLNWHETYKRLKRKSGFSWIQRGLTPTQKAQIMALGIPGIGFRPEIRRFYPGGSVASHILGMV
str.JK31       IKTYSLFAEPRRVIDVDETIELLSTVLSDLNWHETYKRLKRKSGFSWIQRGLTPTQKAQIMALGIPGIGFRPEIRRFYPGGSVASHILGMV
str.JK68       IKTYSLFAEPRRVIDVDETIELLSTVLSDLNWHETYKRLKRKSGFSWIQRGLTPTQKAQIMALGIPGIGFRPEIRRFYPGGSVASHILGMV
str.JK63       IKTYSLFAEPRRVIDVDETIELLSTVLSDLNWHETYKRLKRKSGFSWIQRGLTPTQKAQIMALGIPGIGFRPEIRRFYPGGSVASHILGMV
str.JK67       IKTYSLFAEPRRVIDVDETIELLSTVLSDLNWHETYKRLKRKSGFSWIQRGLTPTQKAQIMALGIPGIGFRPEIRRFYPGGSVASHILGMV
str.JK56       IKTYSLFAEPRRVIDVDETIELLSTVLSDLNWHETYKRLKRKSGFSWIQRGLTPTQKAQIMALGIPGIGFRPEIRRFYPGGSVASHILGMV
str.CO20_0321  IKTYSLFAEPRRVIDVDETIELLSTVLSDLNWHETYKRLKRKSGFSWIQRGLTPTQKAQIMALGIPGIGFRPEIRRFYPGGSVASHILGMV
str.JK73       IKTYSLFAEPRRVIDVDETIELLSTVLSDLNWHETYKRLKRKSGFSWIQRGLTPTQKAQIMALGIPGIGFRPEIRRFYPGGSVASHILGMV
str.JK39       IKTYSLFAEPRRVIDVDETIELLSTVLSDLNWHETYKRLKRKSGFSWIQRGLTPTQKAQIMALGIPGIGFRPEIRRFYPGGSVASHILGMV
str.CO20_0257  IKTYSLFAEPRRVIDVDETIELLSTVLSDLNWHETYKRLKRKSGFSWIQRGLTPTQKAQIMALGIPGIGFRPEIRRFYPGGSVASHILGMV
str.CO20_0256  IKTYSLFAEPRRVIDVDETIELLSTVLSDLNWHETYKRLKRKSGFSWIQRGLTPTQKAQIMALGIPGIGFRPEIRRFYPGGSVASHILGMV
str.CO20_0297  IKTYSLFAEPRRVIDVDETIELLSTVLSDLNWHETYKRLKRKSGFSWIQRGLTPTQKAQIMALGIPGIGFRPEIRRFYPGGSVASHILGMV
str.CO21_0024  IKTYSLFAEPRRVIDVDETIELLSTVLSDLNWHETYKRLKRKSGFSWIQRGLTPTQKAQIMALGIPGIGFRPEIRRFYPGGSVASHILGMV
str.NCTC12899  IKTYSLFAEPRRVIDVDETIELLSTVLSDLNWHETYKRLKRKSGFSWIQRGLTPTQKAQIMALGIPGIGFRPEIRRFYPGGSVASHILGMV
str.JK7        IKTYSLFAEPRRVIDVDETIELLSTVLSDLNWHETYKRLKRKSGFSWIQRGLTPTQKAQIMALGIPGIGFRPEIRRFYPGGSVASHILGMV
str.G1712      IKTYSLFAEPRRVIDVDETIELLSTVLSDLNWHETYKRLKRKSGFSWIQRGLTPTQKAQIMALGIPGIGFRPEIRRFYPGGSVASHILGMV
str.JK12       IKTYSLFAEPRRVIDVDETIELLSTVLSDLNWHETYKRLKRKSGFSWIQRGLTPTQKAQIMALGIPGIGFRPEIRRFYPGGSVASHILGMV
str.CCUG45777  IKTYSLFAEPRRVIDVDETIELLSTVLSDLNWHETYKRLKRKSGFSWIQRGLTPTQKAQIMALGIPGIGFRPEIRRFYPGGSVASHILGMV
str.JK19       IKTYSLFAEPRRVIDVDETIELLSTVLSDLNWHETYKRLKRKSGFSWIQRGLTPTQKAQIMALGIPGIGFRPEIRRFYPGGSVASHILGMV
str.G1713      IKTYSLFAEPRRVIDVDETIELLSTVLSDLNWHETYKRLKRKSGFSWIQRGLTPTQKAQIMALGIPGIGFRPEIRRFYPGGSVASHILGMV
str.JK73rel    IKTYSLFAEPRRVIDVDETIELLSTVLSDLNWHETYKRLKRKSGFSWIQRGLTPTQKAQIMALGIPGIGFRPEIRRFYPGGSVASHILGMV
str.RM-11      IKTYSLFAEPRRVIDVDETIELLSTVLSDLNWHETYKRLKRKSGFSWIQRGLTPTQKAQIMALGIPGIGFRPEIRRFYPGGSVASHILGMV
str.MF1-1      IKTYSLFAEPRRVIDVDETIELLSTVLSDLNWHETYKRLKRKSGFSWIQRGLTPTQKAQIMALGIPGIGFRPEIRRFYPGGSVASHILGMV
```

```
                   190       200       210       220       230       240       250       260       270
str.Toulouse   NVDNQGIAGMEKYIDDAGLSALRSAGLATEEALKPIQLSIDVRIQAIVHDELIKAMKRYKAIAAGAVILNIHTNEVLAMVSVPDFDPGNPV
str.BQ2-D70    NVDNQGIAGMEKYIDDAGLSALRSAGLATEEALKPIQLSIDVRIQAIVHDELIKAMKRYKAIAAGAVILNIHTNEVLAMVSVPDFDPGNPV
str.JK31       NVDNQGIAGMEKYIDDAGLSALRSAGLATEEALKPIQLSIDVRIQAIVHDELIKAMKRYKAIAAGAVILNIHTNEVLAMVSVPDFDPGNPV
str.JK68       NVDNQGIAGMEKYIDDAGLSALRSAGLATEEALKPIQLSIDVRIQAIVHDELIKAMKRYKAIAAGAVILNIHTNEVLAMVSVPDFDPGNPV
str.JK63       NVDNQGIAGMEKYIDDAGLSALRSAGLATEEALKPIQLSIDVRIQAIVHDELIKAMKRYKAIAAGAVILNIHTNEVLAMVSVPDFDPGNPV
str.JK67       NVDNQGIAGMEKYIDDAGLSALRSAGLATEEALKPIQLSIDVRIQAIVHDELIKAMKRYKAIAAGAVILNIHTNEVLAMVSVPDFDPGNPV
str.JK56       NVDNQGIAGMEKYIDDAGLSALRSAGLATEEALKPIQLSIDVRIQAIVHDELIKAMKRYKAIAAGAVILNIHTNEVLAMVSVPDFDPGNPV
str.CO20_0321  NVDNQGIAGMEKYIDDAGLSALRSAGLATEEALKPIQLSIDVRIQAIVHDELIKAMKRYKAIAAGAVILNIHTNEVLAMVSVPDFDPGNPV
str.JK73       NVDNQGIAGMEKYIDDAGLSALRSAGLATEEALKPIQLSIDVRIQAIVHDELIKAMKRYKAIAAGAVILNIHTNEVLAMVSVPDFDPGNPV
str.JK39       NVDNQGIAGMEKYIDDAGLSALRSAGLATEEALKPIQLSIDVRIQAIVHDELIKAMKRYKAIAAGAVILNIHTNEVLAMVSVPDFDPGNPV
str.CO20_0257  NVDNQGIAGMEKYIDDAGLSALRSAGLATEEALKPIQLSIDVRIQAIVHDELINAMKRYKAIAAGAVILNIHTNEVLAMVSVPDFDPGNPV
str.CO20_0256  NVDNQGIAGMEKYIDDAGLSALRSAGLATEEALKPIQLSIDVRIQAIVHDELINAMKRYKAIAAGAVILNIHTNEVLAMVSVPDFDPGNPV
str.CO20_0297  NVDNQGIAGMEKYIDDAGLSALRSAGLATEEALKPIQLSIDVRIQAIVHDELINAMKRYKAIAAGAVILNIHTNEVLAMVSVPDFDPGNPV
str.CO21_0024  NVDNQGIAGMEKYIDDAGLSALRSAGLATEEALKPIQLSIDVRIQAIVHDELINAMKRYKAIAAGAVILNIHTNEVLAMVSVPDFDPGNPV
str.NCTC12899  NVDNQGIAGMEKYIDDAGLSALRSAGLATEEALKPIQLSIDVRIQAIVHDELIKAMKRYKAIAAGAVILNIHTNEVLAMVSVPDFDPGNPV
str.JK7        NVDNQGIAGMEKYIDDAGLSALRSAGLATEEALKPIQLSIDVRIQAIVHDELIKAMKRYKAIAAGAVILNIHTNEVLAMVSVPDFDPGNPV
str.G1712      NVDNQGIAGMEKYIDDAGLSALRSAGLATEEALKPIQLSIDVRIQAIVHDELIKAMKRYKAIAAGAVILNIHTNEVLAMVSVPDFDPGNPV
str.JK12       NVDNQGIAGMEKYIDDAGLSALRSAGLATEEALKPIQLSIDVRIQAIVHDELIKAMKRYKAIAAGAVILNIHTNEVLAMVSVPDFDPGNPV
str.CCUG45777  NVDNQGIAGMEKYIDDAGLSALRSAGLATEEALKPIQLSIDVRIQAIVHDELIKAMKRYKAIAAGAVILNIHTNEVLAMVSVPDFDPGNPV
str.JK19       NVDNQGIAGMEKYIDDAGLSALRSAGLATEEALKPIQLSIDVRIQAIVHDELIKAMKRYKAIAAGAVILNIHTNEVLAMVSVPDFDPGNPV
str.G1713      NVDNQGIAGMEKYIDDAGLSALRSAGLATEEALKPIQLSIDVRIQAIVHDELIKAMKRYKAIAAGAVILNIHTNEVLAMVSVPDFDPGNPV
str.JK73rel    NVDNQGIAGMEKYIDDAGLSALRSAGLATEEALKPIQLSIDVRIQAIVHDELIKAMKRYKAIAAGAVILNIHTNEVLAMVSVPDFDPGNPV
str.RM-11      NVDNQGIAGMEKYIDDAGLSALRSAGLATEEALKPIQLSIDVRIQAIVHDELIKAMKRYKAIAAGAVILNIHTNEVLAMVSVPDFDPGNPV
str.MF1-1      NVDNQGIAGMEKYIDDAGLSALRSAGLATEEALKPIQLSIDVRIQAIVHDELIKAMKRYKAIAAGAVILNIHTNEVLAMVSVPDFDPGNPV
```

```
                     280       290       300       310       320       330       340       350       360
str.Toulouse   DALKSDRLNRITAGAFEMGSIMKSFTTAMALDSDMFHLNSLIDASKPIQASSGYIIHDFHGKNRPLTLWEVFIYSSNIGSAKEALAIGIEK
str.BQ2-D70    DALKSDRLNRITAGAFEMGSIMKSFTTAMALDSDMFHLNSLIDASKPIQASSGYIIHDFHGKNRPLTLWEVFIYSSNIGSAKEALAIGIEK
str.JK31       DALKSDRLNRITAGAFEMGSIMKSFTTAMALDSDMFHLNSLIDASKPIQASSGYIIHDFHGKNRPLTLWEVFIYSSNIGSAKEALAIGIEK
str.JK68       DALKSDRLNRITAGAFEMGSIMKSFTTAMALDSDMFHLNSLIDASKPIQASSGYIIHDFHGKNRPLTLWEVFIYSSNIGSAKEALAIGIEK
str.JK63       DALKSDRLNRITAGAFEMGSIMKSFTTAMALDSDMFHLNSLIDASKPIQASSGYIIHDFHGKNRPLTLWEVFIYSSNIGSAKEALAIGIEK
str.JK67       DALKSDRLNRITAGAFEMGSIMKSFTTAMALDSDMFHLNSLIDASKPIQASSGYIIHDFHGKNRPLTLWEVFIYSSNIGSAKEALAIGIEK
str.JK56       DALKSDRLNRITAGAFEMGSIMKSFTTAMALDSDMFHLNSLIDASKPIQASSGYIIHDFHGKNRPLTLWEVFIYSSNIGSAKEALAIGIEK
str.CO20_0321  DALKSDRLNRITAGAFEMGSIMKSFTTAMALDSDMFHLNSLIDASKPIQASSGYIIHDFHGKNRPLTLWEVFIYSSNIGSAKEALAIGIEK
str.JK73       DALKSDRLNRITAGAFEMGSIMKSFTTAMALDSDMFHLNSLIDASKPIQASSGYIIHDFHGKNRPLTLWEVFIYSSNIGSAKEALAIGIEK
str.JK39       DALKSDRLNRITAGAFEMGSIMKSFTTAMALDSDMFHLNSLIDASKPIQASSGYIIHDFHGKNRPLTLWEVFIYSSNIGSAKEALAIGIEK
str.CO20_0257  DALKSDRLNRITAGAFEMGSIMKSFTTAMALDSDMFHLNSLIDASKPIQASSGYIIHDFHGKNRPLTLWEVFIYSSNIGSAKEALAIGIEK
str.CO20_0256  DALKSDRLNRITAGAFEMGSIMKSFTTAMALDSDMFHLNSLIDASKPIQASSGYIIHDFHGKNRPLTLWEVFIYSSNIGSAKEALAIGIEK
str.CO20_0297  DALKSDRLNRITAGAFEMGSIMKSFTTAMALDSDMFHLNSLIDASKPIQASSGYIIHDFHGKNRPLTLWEVFIYSSNIGSAKEALAIGIEK
str.CO21_0024  DALKSDRLNRITAGAFEMGSIMKSFTTAMALDSDMFHLNSLIDASKPIQASSGYIIHDFHGKNRPLTLWEVFIYSSNIGSAKEALAIGIEK
str.NCTC12899  DALKSDRLNRITAGAFEMGSIMKSFTTAMALDSDMFHLNSLIDASKPIQASSGYIIHDFHGKNRPLTLWEVFIYSSNIGSAKEALAIGIEK
str.JK7        DALKSDRLNRITAGAFEMGSIMKSFTTAMALDSDMFHLNSLIDASKPIQASSGYIIHDFHGKNRPLTLWEVFIYSSNIGSAKEALAIGIEK
str.G1712      DALKSDRLNRITAGAFEMGSIMKSFTTAMALDSDMFHLNSLIDASKPIQASSGYIIHDFHGKNRPLTLWEVFIYSSNIGSAKEALAIGIEK
str.JK12       DALKSDRLNRITAGAFEMGSIMKSFTTAMALDSDMFHLNSLIDASKPIQASSGYIIHDFHGKNRPLTLWEVFIYSSNIGSAKEALAIGIEK
str.CCUG45777  DALKSDRLNRITAGAFEMGSIMKSFTTAMALDSDMFHLNSLIDASKPIQASSGYIIHDFHGKNRPLTLWEVFIYSSNIGSAKEALAIGIEK
str.JK19       DALKSDRLNRITAGAFEMGSIMKSFTTAMALDSDMFHLNSLIDASKPIQASSGYIIHDFHGKNRPLTLWEVFIYSSNIGSAKEALAIGIEK
str.G1713      DALKSDRLNRITAGAFEMGSIMKSFTTAMALDSDMFHLNSLIDASKPIQASSGYIIHDFHGKNRPLTLWEVFIYSSNIGSAKEALAIGIEK
str.JK73rel    DALKSDRLNRITAGAFEMGSIMKSFTTAMALDSDMFHLNSLIDASKPIQASSGYIIHDFHGKNRPLTLWEVFIYSSNIGSAKEALAIGIEK
str.RM-11      DALKSDRLNRITAGAFEMGSIMKSFTTAMALDSDMFHLNSLIDASKPIQASSGYIIHDFHGKNRPLTLWEVFIYSSNIGSAKEALAIGIEK
str.MF1-1      DALKSDRLNRITAGAFEMGSIMKSFTTAMALDSDMFHLNSLIDASKPIQASSGYIIHDFHGKNRPLTLWEVFIYSSNIGSAKEALAIGIEK
```

```
                  370       380       390       400       410       420       430       440       450
str.Toulouse      HRAFLKKLGLLDRLTTELPEVTHPIVPRHWKDIHSMTISFGHGMATTPLQTAVGAAALMNGGWLIAPTFLKRTKEQALKQAKQVLQAKTSQ
str.BQ2-D70       HRAFLKKLGLLDRLTTELPEVTHPIVPRHWKDIHSMTISFGHGMATTPLQTAVGAAALMNGGWLIAPTFLKRTKEQALKQAKQVLQAKTSQ
str.JK31          HRAFLKKLGLLDRLTTELPEVTHPIVPRHWKDIHSMTISFGHGMATTPLQTAVGAAALMNGGWLIAPTFLKRTKEQALKQAKQVLQAKTSQ
str.JK68          HRAFLKKLGLLDRLTTELPEVTHPIVPRHWKDIHSMTISFGHGMATTPLQTAVGAAALMNGGWLIAPTFLKRTKEQALKQAKQVLQAKTSQ
str.JK63          HRAFLKKLGLLDRLTTELPEVTHPIVPRHWKDIHSMTISFGHGMATTPLQTAVGAAALMNGGWLIAPTFLKRTKEQALKQAKQVLQAKTSQ
str.JK67          HRAFLKKLGLLDRLTTELPEVTHPIVPRHWKDIHSMTISFGHGMATTPLQTAVGAAALMNGGWLIAPTFLKRTKEQALKQAKQVLQAKTSQ
str.JK56          HRAFLKKLGLLDRLTTELPEVTHPIVPRHWKDIHSMTISFGHGMATTPLQTAVGAAALMNGGWLIAPTFLKRTKEQALKQAKQVLQAKTSQ
str.CO20_0321     HRAFLKKLGLLDRLTTELPEVTHPIVPRHWKDIHSMTISFGHGMATTPLQTAVGAAALMNGGWLIAPTFLKRTKEQALKQAKQVLQAKTSQ
str.JK73          HRAFLKKLGLLDRLTTELPEVTHPIVPRHWKDIHSMTISFGHGMATTPLQTAVGAAALMNGGWLIAPTFLKRTKEQALKQAKQVLQAKTSQ
str.JK39          HRAFLKKLGLLDRLTTELPEVTHPIVPRHWKDIHSMTISFGHGMATTPLQTAVGAAALMNGGWLIAPTFLKRTKEQALKQAKQVLQAKTSQ
str.CO20_0257     HRAFLKKLGLLDRLTTELPEVTHPIVPRHWKDIHSMTISFGHGMATTPLQTAVGAAALMNGGWLIAPTFLKRTKEQALKQAKQVLQAKTSQ
str.CO20_0256     HRAFLKKLGLLDRLTTELPEVTHPIVPRHWKDIHSMTISFGHGMATTPLQTAVGAAALMNGGWLIAPTFLKRTKEQALKQAKQVLQAKTSQ
str.CO20_0297     HRAFLKKLGLLDRLTTELPEVTHPIVPRHWKDIHSMTISFGHGMATTPLQTAVGAAALMNGGWLIAPTFLKRTKEQALKQAKQVLQAKTSQ
str.CO21_0024     HRAFLKKLGLLDRLTTELPEVTHPIVPRHWKDIHSMTISFGHGMATTPLQTAVGAAALMNGGWLIAPTFLKRTKEQALKQAKQVLQAKTSQ
str.NCTC12899     HRAFLKKLGLLDRLTTELPEVTHPIVPRHWKDIHSMTISFGHGMATTPLQTAVGAAALMNGGWLIAPTFLKRTKEQALKQAKQVLQAKTSQ
str.JK7           HRAFLKKLGLLDRLTTELPEVTHPIVPRHWKDIHSMTISFGHGMATTPLQTAVGAAALMNGGWLIAPTFLKRTKEQALKQAKQVLQAKTSQ
str.G1712         HRAFLKKLGLLDRLTTELPEVTHPIVPRHWKDIHSMTISFGHGMATTPLQTAVGAAALMNGGWLIAPTFLKRTKEQALKQAKQVLQAKTSQ
str.JK12          HRAFLKKLGLLDRLTTELPEVTHPIVPRHWKDIHSMTISFGHGMATTPLQTAVGAAALMNGGWLIAPTFLKRTKEQALKQAKQVLQAKTSQ
str.CCUG45777     HRAFLKKLGLLDRLTTELPEVTHPIVPRHWKDIHSMTISFGHGMATTPLQTAVGAAALMNGGWLIAPTFLKRTKEQALKQAKQVLQAKTSQ
str.JK19          HRAFLKKLGLLDRLTTELPEVTHPIVPRHWKDIHSMTISFGHGMATTPLQTAVGAAALMNGGWLIAPTFLKRTKEQALKQAKQVLQAKTSQ
str.G1713         HRAFLKKLGLLDRLTTELPEVTHPIVPRHWKDIHSMTISFGHGMATTPLQTAVGAAALMNGGWLIAPTFLKRTKEQALKQAKQVLQAKTSQ
str.JK73rel       HRAFLKKLGLLDRLTTELPEVTHPIVPRHWKDIHSMTISFGHGMATTPLQTAVGAAALMNGGWLIAPTFLKRTKEQALKQAKQVLQAKTSQ
str.RM-11         HRAFLKKLGLLDRLTTELPEVTHPIVPRHWKDIHSMTISFGHGMATTPLQTAVGAAALMNGGWLIAPTFLKRTKEQALKQAKQVLQAKTSQ
str.MF1-1         HRAFLKKLGLLDRLTTELPEVTHPIVPRHWKDIHSMTISFGHGMATTPLQTAVGAAALMNGGWLIAPTFLKRTKEQALKQAKKVLQAKTSQ

                  460       470       480       490       500       510       520       530       540
str.Toulouse      NMRYLYKLNSDIGSGRNAKVEGYRVGGKTGTAEKVENGKYSKTKNFNSFLAAFPIEDPAYVVLTIIDEPKPEDGKYAATAGLNAGPMLSNI
str.BQ2-D70       NMRYLYKLNSDIGSGRNAKVEGYRVGGKTGTAEKVENGKYSKTKNFNSFLAAFPIEDPAYVVLTIIDEPKPEDGKYAATAGLNAGPMLSNI
str.JK31          NMRYLYKLNSDIGSGRNAKVEGYRVGGKTGTAEKVENGKYSKTKNFNSFLAAFPIEDPAYVVLTIIDEPKPEDGKYAATAGLNAGPMLSNI
str.JK68          NMRYLYKLNSDIGSGRNAKVEGYRVGGKTGTAEKVENGKYSKTKNFNSFLAAFPIEDPAYVVLTIIDEPKPEDGKYAATAGLNAGPMLSNI
str.JK63          NMRYLYKLNSDIGSGRNAKVEGYRVGGKTGTAEKVENGKYSKTKNFNSFLAAFPIEDPAYVVLTIIDEPKPEDGKYAATAGLNAGPMLSNI
str.JK67          NMRYLYKLNSDIGSGRNAKVEGYRVGGKTGTAEKVENGKYSKTKNFNSFLAAFPIEDPAYVVLTIIDEPKPEDGKYAATAGLNAGPMLSNI
str.JK56          NMRYLYKLNSDIGSGRNAKVEGYRVGGKTGTAEKVENGKYSKTKNFNSFLAAFPIEDPAYVVLTIIDEPKPEDGKYAATAGLNAGPMLSNI
str.CO20_0321     NMRYLYKLNSDIGSGRNAKVEGYRVGGKTGTAEKVENGKYSKTKNFNSFLAAFPIEDPAYVVLTIIDEPKPEDGKYAATAGLNAGPMLSNI
str.JK73          NMRYLYKLNSDIGSGRNAKVEGYRVGGKTGTAEKVENGKYSKTKNFNSFLAAFPIEDPAYVVLTIIDEPKPEDGKYAATAGLNAGPMLSNI
str.JK39          NMRYLYKLNSDIGSGRNAKVEGYRVGGKTGTAEKVENGKYSKTKNFNSFLAAFPIEDPAYVVLTIIDEPKPEDGKYAATAGLNAGPMLSNI
str.CO20_0257     NMRYLYKLNSDIGSGRNAKVEGYRVGGKTGTAEKVENGKYSKTKNFNSFLAAFPIEDPAYVVLTIIDEPKPEDGKYAATAGLNAGPMLSNI
str.CO20_0256     NMRYLYKLNSDIGSGRNAKVEGYRVGGKTGTAEKVENGKYSKTKNFNSFLAAFPIEDPAYVVLTIIDEPKPEDGKYAATAGLNAGPMLSNI
str.CO20_0297     NMRYLYKLNSDIGSGRNAKVEGYRVGGKTGTAEKVENGKYSKTKNFNSFLAAFPIEDPAYVVLTIIDEPKPEDGKYAATAGLNAGPMLSNI
str.CO21_0024     NMRYLYKLNSDIGSGRNAKVEGYRVGGKTGTAEKVENGKYSKTKNFNSFLAAFPIEDPAYVVLTIIDEPKPEDGKYAATAGLNAGPMLSNI
str.NCTC12899     NMRYLYKLNSDIGSGRNAKVEGYRVGGKTGTAEKVENGKYSKTKNFNSFLAAFPIEDPAYVVLTIIDEPKPEDGKYAATAGLNAGPMLSNI
str.JK7           NMRYLYKLNSDIGSGRNAKVEGYRVGGKTGTAEKVENGKYSKTKNFNSFLAAFPIEDPAYVVLTIIDEPKPEDGKYAATAGLNAGPMLSNI
str.G1712         NMRYLYKLNSDIGSGRNAKVEGYRVGGKTGTAEKVENGKYSKTKNFNSFLAAFPIEDPAYVVLTIIDEPKPEDGKYAATAGLNAGPMLSNI
str.JK12          NMRYLYKLNSDIGSGRNAKVEGYRVGGKTGTAEKVENGKYSKTKNFNSFLAAFPIEDPAYVVLTIIDEPKPEDGKYAATAGLNAGPMLSNI
str.CCUG45777     NMRYLYKLNSDIGSGRNAKVEGYRVGGKTGTAEKVENGKYSKTKNFNSFLAAFPIEDPAYVVLTIIDEPKPEDGKYAATAGLNAGPMLSNI
str.JK19          NMRYLYKLNSDIGSGRNAKVEGYRVGGKTGTAEKVENGKYSKTKNFNSFLAAFPIEDPAYVVLTIIDEPKPEDGKYAATAGLNAGPMLSNI
str.G1713         NMRYLYKLNSDIGSGRNAKVEGYRVGGKTGTAEKVENGKYSKTKNFNSFLAAFPIEDPAYVVLTIIDEPKPEDGKYAATAGLNAGPMLSNI
str.JK73rel       NMRYLYKLNSDIGSGRNAKVEGYRVGGKTGTAEKVENGKYSKTKNFNSFLAAFPIEDPAYVVLTIIDEPKPEDGKYAATAGLNAGPMLSNI
str.RM-11         NMRYLYKLNSDIGSGRNAKVEGYRVGGKTGTAEKVENGKYSKTKNFNSFLAAFPIEDPAYVVLTIIDEPKPEDGKYAATAGLNAGPMLSNI
str.MF1-1         NMRYLYKLNSDIGSGRNAKVEGYRVGGKTGTAEKVENGKYSKTKNFNSFLAAFPIEDPAYVVLTIIDEPKPEDGKYAATAGLNAGPMLSNI

                  550       560       570       580
str.Toulouse      VRRSASFLGIKPDFKKEYDSILSTKNSSRLVKQR
str.BQ2-D70       VRRSASFLGIKPDFKKEYDSILGTKNSSRLVKQR
str.JK31          VRRSASFLGIKPDFKKEYDSILGTKNSSRLVKQR
str.JK68          VRRSASFLGIKPDFKKEYDSILGTKNSSRLVKQR
str.JK63          VRRSASFLGIKPDFKKEYDSILGTKNSSRLVKQR
str.JK67          VRRSASFLGIKPDFKKEYDSILGTKNSSRLVKQR
str.JK56          VRRSASFLGIKPDFKKEYDSILGTKNSSRLVKQR
str.CO20_0321     VRRSASFLGIKPDFKKEYDSILSTKNSSRLVKQR
str.JK73          VRRSASFLGIKPDFKKEYDSILSTKNSSRLVKQR
str.JK39          VRRSASFLGIKPDFKKEYDSILSTKNSSRLVKQR
str.CO20_0257     VRRSASFLGIKPDFKKEYDSILSTKNSSRLVKQR
str.CO20_0256     VRRSASFLGIKPDFKKEYDSILSTKNSSRLVKQR
str.CO20_0297     VRRSASFLGIKPDFKKEYDSILSTKNSSRLVKQR
str.CO21_0024     VRRSASFLGIKPDFKKEYDSILSTKNSSRLVKQR
str.NCTC12899     VRRSASFLGIKPDFKKEYDSILSTKNSSRLVKQR
str.JK7           VRRSASFLGIKPDFKKEYDSILSTKNSSRLVKQR
str.G1712         VRRSASFLGIKPDFKKEYDSILSTKNSSRLVKQR
str.JK12          VRRSASFLGIKPDFKKEYDSILSTKNSSRLVKQR
str.CCUG45777     VRRSASFLGIKPDFKKEYDSILSTKNSSRLVKQR
str.JK19          VRRSASFLGIKPDFKKEYDSILSTKNSSRLVKQR
str.G1713         VRRSASFLGIKPDFKKEYDSILSTKNSSRLVKQR
str.JK73rel       VRRSASFLGIKPDFKKEYDSILSTKNSSRLVKQR
str.RM-11         VRRSASFLGIKPDFKKEYDSILSTKNSSRLVKQR
str.MF1-1         VRRSASFLGIKPDFKKEYDSILSTKNSSRLVKQR
```

>WP_011179659.1 ABC transporter permease [Bartonella quintana str. Toulouse]

```
               1        10        20        30        40        50        60        70        80        90
str.Toulouse   MAIMNHPKPQSLFSLSRWLNESVPRSIIQAKLQKIYHSVIKFSRNFSAVFGLIIIFVMILCAVFAPWIATHDFVSNDLAHRLQPPSMLHYL
str.CCUG45777  MAIMNHPKPQSLFSLSRWLNESVPRSIIQAKLQKIYHSVIKFSRNFSAVFGLIIIFVMILCAVFAPWIATHDFVSNDLAHRLQPPSMLHYL
str.Jk56       MAIMNHPKPQSLFSLSRWLNESVPRSIIQAKLQKIYHSVIKFSRNFSAVFGLIIIFVMILCAVFAPWIATHDFVSNDLAHRLQPPSMLHYL
str.BQ2-D70    MAIMNHPKPQSLFSLSRWLNESVPRSIIQAKLQKIYHSVIKFSRNFSAVFGLIIIFVMILCAVFAPWIATHDFVSNDLAHRLQPPSMLHYL
str.Jk67       MAIMNHPKPQSLFSLSRWLNESVPRSIIQAKLQKIYHSVIKFSRNFSAVFGLIIIFVMILCAVFAPWIATHDFVSNDLAHRLQPPSMLHYL
str.Jk12       MAIMNHPKPQSLFSLSRWLNESVPRSIIQAKLQKIYHSVIKFSRNFSAVFGLIIIFVMILCAVFAPWIATHDFVSNDLAHRLQPPSMLHYL
str.Jk19       MAIMNHPKPQSLFSLSRWLNESVPRSIIQAKLQKIYHSVIKFSRNFSAVFGLIIIFVMILCAVFAPWIATHDFVSNDLAHRLQPPSMLHYL
str.Jk7        MAIMNHPKPQSLFSLSRWLNESVPRSIIQAKLQKIYHSVIKFSRNFSAVFGLIIIFVMILCAVFAPWIATHDFVSNDLAHRLQPPSMLHYL
str.Jk73rel    MAIMNHPKPQSLFSLSRWLNESVPRSIIQAKLQKIYHSVIKFSRNFSAVFGLIIIFVMILCAVFAPWIATHDFVSNDLAHRLQPPSMLHYL
str.Jk63       MAIMNHPKPQSLFSLSRWLNESVPRSIIQAKLQKIYHSVIKFSRNFSAVFGLIIIFVMILCAVFAPWIATHDFVSNDLAHRLQPPSMLHYL
str.Jk73       MAIMNHPKPQSLFSLSRWLNESVPRSIIQAKLQKIYHSVIKFSRNFSAVFGLIIIFVMILCAVFAPWIATHDFVSNDLAHRLQPPSMLHYL
str.Jk68       MAIMNHPKPQSLFSLSRWLNESVPRSIIQAKLQKIYHSVIKFSRNFSAVFGLIIIFVMILCAVFAPWIATHDFVSNDLAHRLQPPSMLHYL
str.Jk39       MAIMNHPKPQSLFSLSRWLNESVPRSIIQAKLQKIYHSVIKFSRNFSAVFGLIIIFVMILCAVFAPWIATHDFVSNDLAHRLQPPSMLHYL
str.G1712      MAIMNHPKPQSLFSLSRWLNESVPRSIIQAKLQKIYHSVIKFSRNFSAVFGLIIIFVMILCAVFAPWIATHDFVSNDLAHRLQPPSMLHYL
str.G1713      MAIMNHPKPQSLFSLSRWLNESVPRSIIQAKLQKIYHSVIKFSRNFSAVFGLIIIFVMILCAVFAPWIATHDFVSNDLAHRLQPPSMLHYL
str.CO20_0297  MAIMNHPKPQSLFSLSRWLNESVPRSIIQAKLQKIYHSVIKFSRNFSAVFGLIIIFVMILCAVFAPWIATHDFVSNDLAHRLQPPSMLHYL
str.MF1-1      MAIMNHPKPQSLFSLSRWLNESVPRSIIQAKLQKIYHSVIKFSRNFSAVFGLIIIFVMILCAVFAPWIATHDFVSNDLAHRLQPPSMLHYL
str.CO21_0024  MAIMNHPKPQSLFSLSRWLNESVPRSIIQAKLQKIYHSVIKFSRNFSAVFGLIIIFVMILCAVFAPWIATHDFVSNDLAHRLQPPSMLHYL
str.CO20_0257  MAIMNHPKPQSLFSLSRWLNESVPRSIIQAKLQKIYHSVIKFSRNFSAVFGLIIIFVMILCAVFAPWIATHDFVSNDLAHRLQPPSMLHYL
str.CO20_0321  MAIMNHPKPQSLFSLSRWLNESVPRSIIQAKLQKIYHSVIKFSRNFSAVFGLIIIFVMILCAVFAPWIATHDFVSNDLAHRLQPPSMLHYL
str.CO20_0256  MAIMNHPKPQSLFSLSRWLNESVPRSIIQAKLQKIYHSVIKFSRNFSAVFGLIIIFVMILCAVFAPWIATHDFVSNDLAHRLQPPSMLHYL
str.Jk31       MAIMNHPKPQSLFSLSRWLNESVPRSIIQAKLQKIYHSVIKFSRNFSAVFGLIIIFVMILCAVFAPWIATHDFVSNDLAHRLQPPSMLHYL
str.NCTC12899  MAIMNHPKPQSLFSLSRWLNESVPRSIIQAKLQKIYHSVIKFSRNFSAVFGLIIIFVMILCAVFAPWIATHDFVSNDLAHRLQPPSMLHYL
str.RM-11      MAIMNHPKPQSLFSLSRWLNESVPRSIIQAKLQKIYHSVIKFSRNFSAVFGLIIIFFMILCAVFAPWIATHDFVSNDLAHRLQPPSMLHYL

                      100       110       120       130       140       150       160       170       180
str.Toulouse   GTDELGRDIFSRLVFGTRITLYIIFLTTIIVGPIGLIIGTVSGYIGGWVDTLLMRIVDIFLAFPGLILALAFAAALGPGIENASIAISIAA
str.CCUG45777  GTDELGRDIFSRLVFGTRITLYIIFLTTIIVGPIGLIIGTVSGYIGGWVDTLLMRIVDIFLAFPGLILALAFAAALGPGIENASIAISIAA
str.Jk56       GTDELGRDIFSRLVFGTRITLYIIFLTTIIVGPIGLIIGTVSGYIGGWVDTLLMRIVDIFLAFPGLILALAFAAALGPGIENASIAISIAA
str.BQ2-D70    GTDELGRDIFSRLVFGTRITLYIIFLTTIIVGPIGLIIGTVSGYIGGWVDTLLMRIVDIFLAFPGLILALAFAAALGPGIENASIAISIAA
str.Jk67       GTDELGRDIFSRLVFGTRITLYIIFLTTIIVGPIGLIIGTVSGYIGGWVDTLLMRIVDIFLAFPGLILALAFAAALGPGIENASIAISIAA
str.Jk12       GTDELGRDIFSRLVFGTRITLYIIFLTTIIVGPIGLIIGTVSGYIGGWVDTLLMRIVDIFLAFPGLILALAFAAALGPGIENASIAISIAA
str.Jk19       GTDELGRDIFSRLVFGTRITLYIIFLTTIIVGPIGLIIGTVSGYIGGWVDTLLMRIVDIFLAFPGLILALAFAAALGPGIENASIAISIAA
str.Jk7        GTDELGRDIFSRLVFGTRITLYIIFLTTIIVGPIGLIIGTVSGYIGGWVDTLLMRIVDIFLAFPGLILALAFAAALGPGIENASIAISIAA
str.Jk73rel    GTDELGRDIFSRLVFGTRITLYIIFLTTIIVGPIGLIIGTVSGYIGGWVDTLLMRIVDIFLAFPGLILALAFAAALGPGIENASIAISIAA
str.Jk63       GTDELGRDIFSRLVFGTRITLYIIFLTTIIVGPIGLIIGTVSGYIGGWVDTLLMRIVDIFLAFPGLILALAFAAALGPGIENASIAISIAA
str.Jk73       GTDELGRDIFSRLVFGTRITLYIIFLTTIIVGPIGLIIGTVSGYIGGWVDTLLMRIVDIFLAFPGLILALAFAAALGPGIENASIAISIAA
str.Jk68       GTDELGRDIFSRLVFGTRITLYIIFLTTIIVGPIGLIIGTVSGYIGGWVDTLLMRIVDIFLAFPGLILALAFAAALGPGIENASIAISIAA
str.Jk39       GTDELGRDIFSRLVFGTRITLYIIFLTTIIVGPIGLIIGTVSGYIGGWVDTLLMRIVDIFLAFPGLILALAFAAALGPGIENASIAISIAA
str.G1712      GTDELGRDIFSRLVFGTRITLYIIFLTTIIVGPIGLIIGTVSGYIGGWVDTLLMRIVDIFLAFPGLILALAFAAALGPGIENASIAISIAA
str.G1713      GTDELGRDIFSRLVFGTRITLYIIFLTTIIVGPIGLIIGTVSGYIGGWVDTLLMRIVDIFLAFPGLILALAFAAALGPGIENASIAISIAA
str.CO20_0297  GTDELGRDIFSRLVFGTRITLYIIFLTTIIVGPIGLIIGTVSGYIGGWVDTLLMRIVDIFLAFPGLILALAFAAALGPGIENASIAISIAA
str.MF1-1      GTDELGRDIFSRLVFGTRITLYIIFLTTIIVGPIGLIIGTVSGYIGGWVDTLLMRIVDIFLAFPGLILALAFAAALGPGIENASIAISIAA
str.CO21_0024  GTDELGRDIFSRLVFGTRITLYIIFLTTIIVGPIGLIIGTVSGYIGGWVDTLLMRIVDIFLAFPGLILALAFAAALGPGIENASIAISIAA
str.CO20_0257  GTDELGRDIFSRLVFGTRITLYIIFLTTIIVGPIGLIIGTVSGYIGGWVDTLLMRIVDIFLAFPGLILALAFAAALGPGIENASIAISIAA
str.CO20_0321  GTDELGRDIFSRLVFGTRITLYIIFLTTIIVGPIGLIIGTVSGYIGGWVDTLLMRIVDIFLAFPGLILALAFAAALGPGIENASIAISIAA
str.CO20_0256  GTDELGRDIFSRLVFGTRITLYIIFLTTIIVGPIGLIIGTVSGYIGGWVDTLLMRIVDIFLAFPGLILALAFAAALGPGIENASIAISIAA
str.Jk31       GTDELGRDIFSRLVFGTRITLYIIFLTTIIVGPIGLIIGTVSGYIGGWVDTLLMRIVDIFLAFPGLILALAFAAALGPGIENASIAISIAA
str.NCTC12899  GTDELGRDIFSRLVFGTRITLYIIFLTTIIVGPIGLIIGTVSGYIGGWVDTLLMRIVDIFLAFPGLILALAFAAALGPGIENASIAISIAA
str.RM-11      GTDELGRDIFSRLVFGTRITLYIIFLTTIIVGPIGLIIGTVSGYIGGWVDTLLMRIVDIFLAFPGLILALAFAAALGPGIENASIAISIAA

                    190       200       210       220       230       240       250       260       270
str.Toulouse   WPPIARLARAETLTIRSSDYVSAVRLQGASAWRIILFHVAPMCIPSVIVRLTLDMSGIILTAAGLGFLGLGAQLPSPEWGAMLSTGREFMM
str.CCUG45777  WPPIARLARAETLTIRSSDYVSAVRLQGASAWRIILFHVAPMCIPSVIVRLTLDMSGIILTAAGLGFLGLGAQLPSPEWGAMLSTGREFMM
str.Jk56       WPPIARLARAETLTIRSSDYVSAVRLQGASAWRIILFHVAPMCIPSVIVRLTLDMSGIILTAAGLGFLGLGAQLPSPEWGAMLSTGREFMM
str.BQ2-D70    WPPIARLARAETLTIRSSDYVSAVRLQGASAWRIILFHVAPMCIPSVIVRLTLDMSGIILTAAGLGFLGLGAQLPSPEWGAMLSTGREFMM
str.Jk67       WPPIARLARAETLTIRSSDYVSAVRLQGASAWRIILFHVAPMCIPSVIVRLTLDMSGIILTAAGLGFLGLGAQLPSPEWGAMLSTGREFMM
str.Jk12       WPPIARLARAETLTIRSSDYVSAVRLQGASAWRIILFHVAPMCIPSVIVRLTLDMSGIILTAAGLGFLGLGAQLPSPEWGAMLSTGREFMM
str.Jk19       WPPIARLARAETLTIRSSDYVSAVRLQGASAWRIILFHVAPMCIPSVIVRLTLDMSGIILTAAGLGFLGLGAQLPSPEWGAMLSTGREFMM
str.Jk7        WPPIARLARAETLTIRSSDYVSAVRLQGASAWRIILFHVAPMCIPSVIVRLTLDMSGIILTAAGLGFLGLGAQLPSPEWGAMLSTGREFMM
str.Jk73rel    WPPIARLARAETLTIRSSDYVSAVRLQGASAWRIILFHVAPMCIPSVIVRLTLDMSGIILTAAGLGFLGLGAQLPSPEWGAMLSTGREFMM
str.Jk63       WPPIARLARAETLTIRSSDYVSAVRLQGASAWRIILFHVAPMCIPSVIVRLTLDMSGIILTAAGLGFLGLGAQLPSPEWGAMLSTGREFMM
str.Jk73       WPPIARLARAETLTIRSSDYVSAVRLQGASAWRIILFHVAPMCIPSVIVRLTLDMSGIILTAAGLGFLGLGAQLPSPEWGAMLSTGREFMM
str.Jk68       WPPIARLARAETLTIRSSDYVSAVRLQGASAWRIILFHVAPMCIPSVIVRLTLDMSGIILTAAGLGFLGLGAQLPSPEWGAMLSTGREFMM
str.Jk39       WPPIARLARAETLTIRSSDYVSAVRLQGASAWRIILFHVAPMCIPSVIVRLTLDMSGIILTAAGLGFLGLGAQLPSPEWGAMLSTGREFMM
str.G1712      WPPIARLARAETLTIRSSDYVSAVRLQGASAWRIILFHVAPMCIPSVIVRLTLDMSGIILTAAGLGFLGLGAQLPSPEWGAMLSTGREFMM
str.G1713      WPPIARLARAETLTIRSSDYVSAVRLQGASAWRIILFHVAPMCIPSVIVRLTLDMSGIILTAAGLGFLGLGAQLPSPEWGAMLSTGREFMM
str.CO20_0297  WPPIARLARAETLTIRSSDYVSAVRLQGASAWRIILFHVAPMCIPSVIVRLTLDMSGIILTAAGLGFLGLGAQLPSPEWGAMLSTGREFMM
str.MF1-1      WPPIARLARAETLTIRSSDYVSAVRLQGASAWRIILFHVAPMCIPSVIVRLTLDMSGIILTAAGLGFLGLGAQLPSPEWGAMLSTGREFMM
str.CO21_0024  WPPIARLARAETLTIRSSDYVSAVRLQGASAWRIILFHVAPMCIPSVIVRLTLDMSGIILTAAGLGFLGLGAQLPSPEWGAMLSTGREFMM
str.CO20_0257  WPPIARLARAETLTIRSSDYVSAVRLQGASAWRIILFHVAPMCIPSVIVRLTLDMSGIILTAAGLGFLGLGAQLPSPEWGAMLSTGREFMM
str.CO20_0321  WPPIARLARAETLTIRSSDYVSAVRLQGASAWRIILFHVAPMCIPSVIVRLTLDMSGIILTAAGLGFLGLGAQLPSPEWGAMLSTGREFMM
str.CO20_0256  WPPIARLARAETLTIRSSDYVSAVRLQGASAWRIILFHVAPMCIPSVIVRLTLDMSGIILTAAGLGFLGLGAQLPSPEWGAMLSTGREFMM
str.Jk31       WPPIARLARAETLTIRSSDYVSAVRLQGASAWRIILFHVAPMCIPSVIVRLTLDMSGIILTAAGLGFLGLGAQLPSPEWGAMLSTGREFMM
str.NCTC12899  WPPIARLARAETLTIRSSDYVSAVRLQGASAWRIILFHVAPMCIPSVIVRLTLDMSGIILTAAGLGFLGLGAQLPSPEWGAMLSTGREFMM
str.RM-11      WPPIARLARAETLTIRSSDYVSAVRLQGASAWRIILFHVAPMCIPSVIVRLTLDMSGIILTAAGLGFLGLGAQLPSPEWGAMLSTGREFMM

                  280       290       300
str.Toulouse   TCWWVAAMPGCAILCASLAFNLLGDGLRDILDPRNG
str.CCUG45777  TCWWVAAMPGCAILCASLAFNLLGDGLRDILDPRNG
str.Jk56       TCWWVAAMPGCAILCASLAFNLLGDGLRDILDPRNG
str.BQ2-D70    TCWWVAAMPGCAILCASLAFNLLGDGLRDILDPRNG
str.Jk67       TCWWVAAMPGCAILCASLAFNLLGDGLRDILDPRNG
str.Jk12       TCWWVAAMPGCAILCASLAFNLLGDGLRDILDPRNG
str.Jk19       TCWWVAAMPGCAILCASLAFNLLGDGLRDILDPRNG
str.Jk7        TCWWVAAMPGCAILCASLAFNLLGDGLRDILDPRNG
str.Jk73rel    TCWWVAAMPGCAILCASLAFNLLGDGLRDILDPRNG
str.Jk63       TCWWVAAMPGCAILCASLAFNLLGDGLRDILDPRNG
str.Jk73       TCWWVAAMPGCAILCASLAFNLLGDGLRDILDPRNG
str.Jk68       TCWWVAAMPGCAILCASLAFNLLGDGLRDILDPRNG
str.Jk39       TCWWVAAMPGCAILCASLAFNLLGDGLRDILDPRNG
str.G1712      TCWWVAAMPGCAILCASLAFNLLGDGLRDILDPRNG
str.G1713      TCWWVAAMPGCAILCASLAFNLLGDGLRDILDPRNG
str.CO20_0297  TCWWVAAMPGCAILCASLAFNLLGDGLRDILDPRNG
str.MF1-1      TCWWVAAMPGCAILCASLAFNLLGDGLRDILDPRNG
str.CO21_0024  TCWWVAAMPGCAILCASLAFNLLGDGLRDILDPRNG
str.CO20_0257  TCWWVAAMPGCAILCASLAFNLLGDGLRDILDPRNG
str.CO20_0321  TCWWVAAMPGCAILCASLAFNLLGDGLRDILDPRNG
str.CO20_0256  TCWWVAAMPGCAILCASLAFNLLGDGLRDILDPRNG
str.Jk31       TCWWVAAMPGCAILCASLAFNLLGDGLRDILDPRNG
str.NCTC12899  TCWWVAAMPGCAILCASLAFNLLGDGLRDILDPRNG
str.RM-11      TCWWVAAMPGCAILCASLAFNLLGDGLRDILDPRNG
```

>WP_011179660.1 ABC transporter permease [Bartonella quintana str. Toulouse]

```
               1         10        20        30        40        50        60        70        80        90
str.Toulouse   MTLSFPKANGVISPWQKNLDIWVFLFKAFKLLISLFITLLGLVTITFFIGHLLPLDPVLAILGDNISQEAYDAMFYKLGLDKPLIVQYWNY
str.CO20_0321  MTLSFPKANGVISPWQKNLDIWVFLFKAFKLLISLFITLLGLVTITFFIGHLLPLDPVLAILGDNISQEAYDAMFYKLGLDKPLIVQYWNY
str.JK73rel    MTLSFPKANGVISPWQKNLDIWVFLFKAFKLLISLFITLLGLVTITFFIGHLLPLDPVLAILGDNISQEAYDAMFYKLGLDKPLIVQYWNY
str.JK7        MTLSFPKANGVISPWQKNLDIWVFLFKAFKLLISLFITLLGLVTITFFIGHLLPLDPVLAILGDNISQEAYDAMFYKLGLDKPLIVQYWNY
str.JK73       MTLSFPKANGVISPWQKNLDIWVFLFKAFKLLISLFITLLGLVTITFFIGHLLPLDPVLAILGDNISQEAYDAMFYKLGLDKPLIVQYWNY
str.G1712      MTLSFPKANGVISPWQKNLDIWVFLFKAFKLLISLFITLLGLVTITFFIGHLLPLDPVLAILGDNISQEAYDAMFYKLGLDKPLIVQYWNY
str.G1713      MTLSFPKANGVISPWQKNLDIWVFLFKAFKLLISLFITLLGLVTITFFIGHLLPLDPVLAILGDNISQEAYDAMFYKLGLDKPLIVQYWNY
str.JK31       MTLSFPKANGVISPWQKNLDIWVFLFKAFKLLISLFITLLGLVTITFFIGHLLPLDPVLAILGDNISQEAYDAMFYKLGLDKPLIVQYWNY
str.JK68       MTLSFPKANGVISPWQKNLDIWVFLFKAFKLLISLFITLLGLVTITFFIGHLLPLDPVLAILGDNISQEAYDAMFYKLGLDKPLIVQYWNY
str.JK39       MTLSFPKANGVISPWQKNLDIWVFLFKAFKLLISLFITLLGLVTITFFIGHLLPLDPVLAILGDNISQEAYDAMFYKLGLDKPLIVQYWNY
str.JK63       MTLSFPKANGVISPWQKNLDIWVFLFKAFKLLISLFITLLGLVTITFFIGHLLPLDPVLAILGDNISQEAYDAMFYKLGLDKPLIVQYWNY
str.JK19       MTLSFPKANGVISPWQKNLDIWVFLFKAFKLLISLFITLLGLVTITFFIGHLLPLDPVLAILGDNISQEAYDAMFYKLGLDKPLIVQYWNY
str.JK67       MTLSFPKANGVISPWQKNLDIWVFLFKAFKLLISLFITLLGLVTITFFIGHLLPLDPVLAILGDNISQEAYDAMFYKLGLDKPLIVQYWNY
str.JK12       MTLSFPKANGVISPWQKNLDIWVFLFKAFKLLISLFITLLGLVTITFFIGHLLPLDPVLAILGDNISQEAYDAMFYKLGLDKPLIVQYWNY
str.BQ2-D70    MTLSFPKANGVISPWQKNLDIWVFLFKAFKLLISLFITLLGLVTITFFIGHLLPLDPVLAILGDNISQEAYDAMFYKLGLDKPLIVQYWNY
str.CCUG45777  MTLSFPKANGVISPWQKNLDIWVFLFKAFKLLISLFITLLGLVTITFFIGHLLPLDPVLAILGDNISQEAYDAMFYKLGLDKPLIVQYWNY
str.JK56       MTLSFPKANGVISPWQKNLDIWVFLFKAFKLLISLFITLLGLVTITFFIGHLLPLDPVLAILGDNISQEAYDAMFYKLGLDKPLIVQYWNY
str.NCTC12899  MTLSFPKANGVISPWQKNLDIWVFLFKAFKLLISLFITLLGLVTITFFIGHLLPLDPVLAILGDNISQEAYDAMFYKLGLDKPLIVQYWNY
str.CO20_0257  MTLSFPKANGVISPWQKNLDIWVFLFKAFKLLISLFITLLGLVTITFFIGHLLPLDPVLAILGDNISQEAYDAMFYKLGLDKPLIVQYWNY
str.CO20_0256  MTLSFPKANGVISPWQKNLDIWVFLFKAFKLLISLFITLLGLVTITFFIGHLLPLDPVLAILGDNISQEAYDAMFYKLGLDKPLIVQYWNY
str.CO20_0297  MTLSFPKANGVISPWQKNLDIWVFLFKAFKLLISLFITLLGLVTITFFIGHLLPLDPVLAILGDNISQEAYDAMFYKLGLDKPLIVQYWNY
str.CO21_0024  MTLSFPKANGVISPWQKNLDIWVFLFKAFKLLISLFITLLGLVTITFFIGHLLPLDPVLAILGDNISQEAYDAMFYKLGLDKPLIVQYWNY
str.MF1-1      MTLSSPKANGVISPWQKNLDIWVFLFKAFKLLISVFITLLGLVTITFFIGHLLPLDPVLAILGDNISQEAYDAMFYKLGLDKPLIVQYWNY
str.RM-11      MTLSSPKANGVISPWQKNLDIWVFLFKAFKLLISVFITLLGLVTITFFIGHLLPLDPVLAILGDNISQEAYDAMFYKLGLDKPLIVQYWNY

               100       110       120       130       140       150       160       170       180
str.Toulouse   LHNILLFDFGDALTSGRPVLTDIMRVFPATLELATVAIVIGTAFGIPFGVFAAMYRDSFIDYFVRVFTLLRYSTPTFWLGLMALLVFYAKL
str.CO20_0321  LHNILLFDFGDALTSGRPVLTDIMRVFPATLELATVAIVIGTAFGIPFGVFAAMYRDSFIDYFVRVFTLLRYSTPTFWLGLMALLVFYAKL
str.JK73rel    LHNILLFDFGDALTSGRPVLTDIMRVFPATLELATVAIVIGTAFGIPFGVFAAMYRDSFIDYFVRVFTLLRYSTPTFWLGLMALLVFYAKL
str.JK7        LHNILLFDFGDALTSGRPVLTDIMRVFPATLELATVAIVIGTAFGIPFGVFAAMYRDSFIDYFVRVFTLLRYSTPTFWLGLMALLVFYAKL
str.JK73       LHNILLFDFGDALTSGRPVLTDIMRVFPATLELATVAIVIGTAFGIPFGVFAAMYRDSFIDYFVRVFTLLRYSTPTFWLGLMALLVFYAKL
str.G1712      LHNILLFDFGDALTSGRPVLTDIMRVFPATLELATVAIVIGTAFGIPFGVFAAMYRDSFIDYFVRVFTLLRYSTPTFWLGLMALLVFYAKL
str.G1713      LHNILLFDFGDALTSGRPVLTDIMRVFPATLELATVAIVIGTAFGIPFGVFAAMYRDSFIDYFVRVFTLLRYSTPTFWLGLMALLVFYAKL
str.JK31       LHNILLFDFGDALTSGRPVLTDIMRVFPATLELATVAIVIGTAFGIPFGVFAAMYRDSFIDYFVRVFTLLRYSTPTFWLGLMALLVFYAKL
str.JK68       LHNILLFDFGDALTSGRPVLTDIMRVFPATLELATVAIVIGTAFGIPFGVFAAMYRDSFIDYFVRVFTLLRYSTPTFWLGLMALLVFYAKL
str.JK39       LHNILLFDFGDALTSGRPVLTDIMRVFPATLELATVAIVIGTAFGIPFGVFAAMYRDSFIDYFVRVFTLLRYSTPTFWLGLMALLVFYAKL
str.JK63       LHNILLFDFGDALTSGRPVLTDIMRVFPATLELATVAIVIGTAFGIPFGVFAAMYRDSFIDYFVRVFTLLRYSTPTFWLGLMALLVFYAKL
str.JK19       LHNILLFDFGDALTSGRPVLTDIMRVFPATLELATVAIVIGTAFGIPFGVFAAMYRDSFIDYFVRVFTLLRYSTPTFWLGLMALLVFYAKL
str.JK67       LHNILLFDFGDALTSGRPVLTDIMRVFPATLELATVAIVIGTAFGIPFGVFAAMYRDSFIDYFVRVFTLLRYSTPTFWLGLMALLVFYAKL
str.JK12       LHNILLFDFGDALTSGRPVLTDIMRVFPATLELATVAIVIGTAFGIPFGVFAAMYRDSFIDYFVRVFTLLRYSTPTFWLGLMALLVFYAKL
str.BQ2-D70    LHNILLFDFGDALTSGRPVLTDIMRVFPATLELATVAIVIGTAFGIPFGVFAAMYRDSFIDYFVRVFTLLRYSTPTFWLGLMALLVFYAKL
str.CCUG45777  LHNILLFDFGDALTSGRPVLTDIMRVFPATLELATVAIVIGTAFGIPFGVFAAMYRDSFIDYFVRVFTLLRYSTPTFWLGLMALLVFYAKL
str.JK56       LHNILLFDFGDALTSGRPVLTDIMRVFPATLELATVAIVIGTAFGIPFGVFAAMYRDSFIDYFVRVFTLLRYSTPTFWLGLMALLVFYAKL
str.NCTC12899  LHNILLFDFGDALTSGRPVLTDIMRVFPATLELATVAIVIGTAFGIPFGVFAAMYRDSFIDYFVRVFTLLRYSTPTFWLGLMALLVFYAKL
str.CO20_0257  LHNILLFDFGDALTSGRPVLTDIMRVFPATLELATVAIVIGTAFGIPFGVFAAMYRDSFIDYFVRVFTLLRYSTPTFWLGLMALLVFYAKL
str.CO20_0256  LHNILLFDFGDALTSGRPVLTDIMRVFPATLELATVAIVIGTAFGIPFGVFAAMYRDSFIDYFVRVFTLLRYSTPTFWLGLMALLVFYAKL
str.CO20_0297  LHNILLFDFGDALTSGRPVLTDIMRVFPATLELATVAIVIGTAFGIPFGVFAAMYRDSFIDYFVRVFTLLRYSTPTFWLGLMALLVFYAKL
str.CO21_0024  LHNILLFDFGDALTSGRPVLTDIMRVFPATLELATVAIVIGTAFGIPFGVFAAMYRDSFIDYFVRVFTLLRYSTPTFWLGLMALLVFYAKL
str.MF1-1      LHNILLFDFGDALTSGRPVLTDIMRVFPATLELATVAIVIGTAFGIPFGVFAAMYRDSFIDYFVRVFTLLRYSTPTFWLGLMALLVFYAKL
str.RM-11      LHNILLFDFGDALTSGRPVLTDIMRVFPATLELATVAIVIGTAFGIPFGVFAAMYRDSFIDYFVRVFTLLRYSTPTFWLGLMALLVFYAKL

               190       200       210       220       230       240       250       260       270
str.Toulouse   DWISGPGRLDFLYEYSFEPRTGFFLWDTAMQGQWEAFGNVFSHIIMPALILAFGAMAYISRMTRGFMIEQLNQEYIITARVKGLSWMRTVW
str.CO20_0321  DWISGPGRLDFLYEYSFEPRTGFFLWDTAMQGQWEAFGNVFSHIIMPALILAFGAMAYISRMTRGFMIEQLNQEYIITARVKGLSWMRTVW
str.JK73rel    DWISGPGRLDFLYEYSFEPRTGFFLWDTAMQGQWEAFGNVFSHIIMPALILAFGAMAYISRMTRGFMIEQLNQEYIITARVKGLSWMRTVW
str.JK7        DWISGPGRLDFLYEYSFEPRTGFFLWDTAMQGQWEAFGNVFSHIIMPALILAFGAMAYISRMTRGFMIEQLNQEYIITARVKGLSWMRTVW
str.JK73       DWISGPGRLDFLYEYSFEPRTGFFLWDTAMQGQWEAFGNVFSHIIMPALILAFGAMAYISRMTRGFMIEQLNQEYIITARVKGLSWMRTVW
str.G1712      DWISGPGRLDFLYEYSFEPRTGFFLWDTAMQGQWEAFGNVFSHIIMPALILAFGAMAYISRMTRGFMIEQLNQEYIITARVKGLSWMRTVW
str.G1713      DWISGPGRLDFLYEYSFEPRTGFFLWDTAMQGQWEAFGNVFSHIIMPALILAFGAMAYISRMTRGFMIEQLNQEYIITARVKGLSWMRTVW
str.JK31       DWISGPGRLDFLYEYSFEPRTGFFLWDTAMQGQWEAFGNVFSHIIMPALILAFGAMAYISRMTRGFMIEQLNQEYIITARVKGLSWMRTVW
str.JK68       DWISGPGRLDFLYEYSFEPRTGFFLWDTAMQGQWEAFGNVFSHIIMPALILAFGAMAYISRMTRGFMIEQLNQEYIITARVKGLSWMRTVW
str.JK39       DWISGPGRLDFLYEYSFEPRTGFFLWDTAMQGQWEAFGNVFSHIIMPALILAFGAMAYISRMTRGFMIEQLNQEYIITARVKGLSWMRTVW
str.JK63       DWISGPGRLDFLYEYSFEPRTGFFLWDTAMQGQWEAFGNVFSHIIMPALILAFGAMAYISRMTRGFMIEQLNQEYIITARVKGLSWMRTVW
str.JK19       DWISGPGRLDFLYEYSFEPRTGFFLWDTAMQGQWEAFGNVFSHIIMPALILAFGAMAYISRMTRGFMIEQLNQEYIITARVKGLSWMRTVW
str.JK67       DWISGPGRLDFLYEYSFEPRTGFFLWDTAMQGQWEAFGNVFSHIIMPALILAFGAMAYISRMTRGFMIEQLNQEYIITARVKGLSWMRTVW
str.JK12       DWISGPGRLDFLYEYSFEPRTGFFLWDTAMQGQWEAFGNVFSHIIMPALILAFGAMAYISRMTRGFMIEQLNQEYIITARVKGLSWMRTVW
str.BQ2-D70    DWISGPGRLDFLYEYSFEPRTGFFLWDTAMQGQWEAFGNVFSHIIMPALILAFGAMAYISRMTRGFMIEQLNQEYIITARVKGLSWMRTVW
str.CCUG45777  DWISGPGRLDFLYEYSFEPRTGFFLWDTAMQGQWEAFGNVFSHIIMPALILAFGAMAYISRMTRGFMIEQLNQEYIITARVKGLSWMRTVW
str.JK56       DWISGPGRLDFLYEYSFEPRTGFFLWDTAMQGQWEAFGNVFSHIIMPALILAFGAMAYISRMTRGFMIEQLNQEYIITARVKGLSWMRTVW
str.NCTC12899  DWISGPGRLDFLYEYSFEPRTGFFLWDTAMQGQWEAFGNVFSHIIMPALILAFGAMAYISRMTRGFMIEQLNQEYIITARVKGLSWMRTVW
str.CO20_0257  DWISGPGRLDFLYEYSFEPRTGFFLWDTAMQGQWEAFGNVFSHIIMPALILAFGAIAYISRMTRGFMIEQLNQEYIITARVKGLSWMRTVW
str.CO20_0256  DWISGPGRLDFLYEYSFEPRTGFFLWDTAMQGQWEAFGNVFSHIIMPALILAFGAIAYISRMTRGFMIEQLNQEYIITARVKGLSWMRTVW
str.CO20_0297  DWISGPGRLDFLYEYSFEPRTGFFLWDTAMQGQWEAFGNVFSHIIMPALILAFGAIAYISRMTRGFMIEQLNQEYIITARVKGLSWMRTVW
str.CO21_0024  DWISGPGRLDFLYEYSFEPRTGFFLWDTAMQGQWEAFGNVFSHIIMPALILAFGAIAYISRMTRGFMIEQLNQEYIITARVKGLSWMRTVW
str.MF1-1      GWISGPGRLDFLYEYSFEPRTGFFLWDTAMQGQWEAFGNVFSHIIMPALILAFGAMAYISRMTRGFMIEQLNQEYIITARVKGLSWMRTVW
str.RM-11      GWISGPGRLDFLYEYSFEPRTGFFLWDTAMQGQWEAFGNVFSHIIMPALILAFGAMAYISRMTRGFMIEQLNQEYIITARVKGLSWMRTVW

               280       290       300       310       320       330       340       350
str.Toulouse   FHAFRNAAVQIITVIALSYAFLLEGAVLTETVFAWPGFGRYLTNALLSGDMNAVVGCTLLIGFLFVVINLFSDLLYRIFDPRTR
str.CO20_0321  FHAFRNAAVQIITVIALSYAFLLEGAVLTETVFAWPGFGRYLTNALLSGDMNAVVGCTLLIGFLFVVINLFSDLLYRIFDPRTR
str.JK73rel    FHAFRNAAVQIITVIALSYAFLLEGAVLTETVFAWPGFGRYLTNALLSGDMNAVVGCTLLIGFLFVVINLFSDLLYRIFDPRTR
str.JK7        FHAFRNAAVQIITVIALSYAFLLEGAVLTETVFAWPGFGRYLTNALLSGDMNAVVGCTLLIGFLFVVINLFSDLLYRIFDPRTR
str.JK73       FHAFRNAAVQIITVIALSYAFLLEGAVLTETVFAWPGFGRYLTNALLSGDMNAVVGCTLLIGFLFVVINLFSDLLYRIFDPRTR
str.G1712      FHAFRNAAVQIITVIALSYAFLLEGAVLTETVFAWPGFGRYLTNALLSGDMNAVVGCTLLIGFLFVVINLFSDLLYRIFDPRTR
str.G1713      FHAFRNAAVQIITVIALSYAFLLEGAVLTETVFAWPGFGRYLTNALLSGDMNAVVGCTLLIGFLFVVINLFSDLLYRIFDPRTR
str.JK31       FHAFRNAAVQIITVIALSYAFLLEGAVLTETVFAWPGFGRYLTNALLSGDMNAVVGCTLLIGFLFVVINLFSDLLYRIFDPRTR
str.JK68       FHAFRNAAVQIITVIALSYAFLLEGAVLTETVFAWPGFGRYLTNALLSGDMNAVVGCTLLIGFLFVVINLFSDLLYRIFDPRTR
str.JK39       FHAFRNAAVQIITVIALSYAFLLEGAVLTETVFAWPGFGRYLTNALLSGDMNAVVGCTLLIGFLFVVINLFSDLLYRIFDPRTR
str.JK63       FHAFRNAAVQIITVIALSYAFLLEGAVLTETVFAWPGFGRYLTNALLSGDMNAVVGCTLLIGFLFVVINLFSDLLYRIFDPRTR
str.JK19       FHAFRNAAVQIITVIALSYAFLLEGAVLTETVFAWPGFGRYLTNALLSGDMNAVVGCTLLIGFLFVVINLFSDLLYRIFDPRTR
str.JK67       FHAFRNAAVQIITVIALSYAFLLEGAVLTETVFAWPGFGRYLTNALLSGDMNAVVGCTLLIGFLFVVINLFSDLLYRIFDPRTR
str.JK12       FHAFRNAAVQIITVIALSYAFLLEGAVLTETVFAWPGFGRYLTNALLSGDMNAVVGCTLLIGFLFVVINLFSDLLYRIFDPRTR
str.BQ2-D70    FHAFRNAAVQIITVIALSYAFLLEGAVLTETVFAWPGFGRYLTNALLSGDMNAVVGCTLLIGFLFVVINLFSDLLYRIFDPRTR
str.CCUG45777  FHAFRNAAVQIITVIALSYAFLLEGAVLTETVFAWPGFGRYLTNALLSGDMNAVVGCTLLIGFLFVVINLFSDLLYRIFDPRTR
str.JK56       FHAFRNAAVQIITVIALSYAFLLEGAVLTETVFAWPGFGRYLTNALLSGDMNAVVGCTLLIGFLFVVINLFSDLLYRIFDPRTR
str.NCTC12899  FHAFRNAAVQIITVIALSYAFLLEGAVLTETVFAWPGFGRYLTNALLSGDMNAVVGCTLLIGFLFVVINLFSDLLYRIFDPRTR
str.CO20_0257  FHAFRNAAVQIITVIALSYAFLLEGAVLTETVFAWPGFGRYLTNALLSGDMNAVVGCTLLIGFLFVVINLFSDLLYRIFDPRTR
str.CO20_0256  FHAFRNAAVQIITVIALSYAFLLEGAVLTETVFAWPGFGRYLTNALLSGDMNAVVGCTLLIGFLFVVINLFSDLLYRIFDPRTR
str.CO20_0297  FHAFRNAAVQIITVIALSYAFLLEGAVLTETVFAWPGFGRYLTNALLSGDMNAVVGCTLLIGFLFVVINLFSDLLYRIFDPRTR
str.CO21_0024  FHAFRNAAVQIITVIALSYAFLLEGAVLTETVFAWPGFGRYLTNALLSGDMNAVVGCTLLIGFLFVVINLFSDLLYRIFDPRTR
str.MF1-1      FHAFRNAAVQIITVIALSYAFLLEGAVLTETVFAWPGFGRYLTNALLSGDMNAVVGCTLLIGFLFVVINLFSDLLYRIFDPRTR
str.RM-11      FHAFRNAAVQIITVIALSYAFLLEGAVLTETVFAWPGFGRYLTNALLSGDMNAVVGCTLLIGFLFVVINLFSDLLYRIFDPRTR
```

>WP_011179677.1 membrane protein insertase YidC [Bartonella quintana str. Toulouse]

```
               1        10        20        30        40        50        60        70        80        90
str.Toulouse   MEYNRNFFIAIGLSFGVLIAWHFFYVAPKQAQLQQKRLIAQQLSKQQSTLSTSTPYFSDNASITHESASIITHPITPEIRNEALAKTNRIA
str.CCUG45777  MEYNRNFFIAIGLSFGVLIAWHFFYVAPKQAQLQQKRLIAQQLSKQQSTLSTSTPYFSDNASITHESASIITHPITPEIRNEALAKTNRIA
str.JK56       MEYNRNFFIAIGLSFGVLIAWHFFYVAPKQAQLQQKRLIAQQLSKQQSTLSTSTPYFSDNASITHESASIITHPITPEIRNEALAKTNRIA
str.JK67       MEYNRNFFIAIGLSFGVLIAWHFFYVAPKQAQLQQKRLIAQQLSKQQSTLSTSTPYFSDNASITHESASIITHPITPEIRNEALAKTNRIA
str.JK19       MEYNRNFFIAIGLSFGVLIAWHFFYVAPKQAQLQQKRLIAQQLSKQQSTLSTSTPYFSDNASITHESASIITHPITPEIRNEALAKTNRIA
str.JK63       MEYNRNFFIAIGLSFGVLIAWHFFYVAPKQAQLQQKRLIAQQLSKQQSTLSTSTPYFSDNASITHESASIITHPITPEIRNEALAKTNRIA
str.JK39       MEYNRNFFIAIGLSFGVLIAWHFFYVAPKQAQLQQKRLIAQQLSKQQSTLSTSTPYFSDNASITHESASIITHPITPEIRNEALAKTNRIA
str.JK68       MEYNRNFFIAIGLSFGVLIAWHFFYVAPKQAQLQQKRLIAQQLSKQQSTLSTSTPYFSDNASITHESASIITHPITPEIRNEALAKTNRIA
str.G1713      MEYNRNFFIAIGLSFGVLIAWHFFYVAPKQAQLQQKRLIAQQLSKQQSTLSTSTPYFSDNASITHESASIITHPITPEIRNEALAKTNRIA
str.G1712      MEYNRNFFIAIGLSFGVLIAWHFFYVAPKQAQLQQKRLIAQQLSKQQSTLSTSTPYFSDNASITHESASIITHPITPEIRNEALAKTNRIA
str.JK73       MEYNRNFFIAIGLSFGVLIAWHFFYVAPKQAQLQQKRLIAQQLSKQQSTLSTSTPYFSDNASITHESASIITHPITPEIRNEALAKTNRIA
str.JK73rel    MEYNRNFFIAIGLSFGVLIAWHFFYVAPKQAQLQQKRLIAQQLSKQQSTLSTSTPYFSDNASITHESASIITHPITPEIRNEALAKTNRIA
str.CO20_0321  MEYNRNFFIAIGLSFGVLIAWHFFYVAPKQAQLQQKRLIAQQLSKQQSTLSTSTPYFSDNASITHESASIITHPITPEIRNEALAKTNRIA
str.CO21_0024  MEYNRNFFIAIGLSFGVLIAWHFFYVAPKQAQLQQKRLIAQQLSKQQSTLSTSTPYFSDNASITHESASIITHPITPEIRNEALAKTNRIA
str.CO20_0297  MEYNRNFFIAIGLSFGVLIAWHFFYVAPKQAQLQQKRLIAQQLSKQQSTLSTSTPYFSDNASITHESASIITHPITPEIRNEALAKTNRIA
str.JK31       MEYNRNFFIAIGLSFGVLIAWHFFYVAPKQVQLQQKRLIAQQLSKQQSTLSTSTPYFSDNASITHESASIITHPITPEIRNEALAKTNRIA
str.BQ2-D70    MEYNRNFFIAIGLSFGVLIAWHFFYVAPKQVQLQQKRLIAQQLSKQQSTLSTSTPYFSDNASITHESASIITHPITPEIRNEALAKTNRIA
str.CO20_0256  MEYNRNFFIAIGLSFGVLIAWHFFYVAPKQAQLQQKRLIAQQLSKQQSTLSTSTPYFSDNASITHESASIITHPITPEIRNEALAKTNRIA
str.CO20_0257  MEYNRNFFIAIGLSFGVLIAWHFFYVAPKQAQLQQKRLIAQQLSKQQSTLSTSTPYFSDNASITHESASIITHPITPEIRNEALAKTNRIA
str.MF1-1      MEYNRNFFIAIGLSFGVLIAWHFFYVAPKQAQLQQKRLIAQQLSKQQSTLSTSTPYFSDNASITHESASIITHPITPEIRNEALAKTNRIA
str.RM-11      MEYNRNFFIAIGLSFGVLIAWHFFYVAPKQAQLQQKRLIAQQLSKQQSTLSTSTPYFSDNASITHESASIITHPITPEIRNEALAKTNRIA
str.NCTC12899  MEYNRNFFIAIGLSFGVLIAWHFFYVAPKQAQLQQKRLIAQQLSKQQSTLSTSTPYFSDNASITHESASIITHPITPEIRNEALAKTNRIA
str.JK12       MEYNRNFFIAIGLSFGVLIAWHFFYVAPKQAQLQQKRLIAQQLSKQQSTLSTSTPYFSDNASITHESASIITHPITPEIRNEALAKTNRIA
str.JK7        MEYNRNFFIAIGLSFGVLIAWHFFYVAPKQAQLQQKRLIAQQLSKQQSTLSTSTPYFSDNASITHESASIITHPITPEIRNEALAKTNRIA

                      100       110       120       130       140       150       160       170       180
str.Toulouse   IKTDELEGSINLVGAQFDDLLLKKYRLTVDKKSPEIALLNPKGFTTTYLAEFGFTSSSLSAKALPQSDTQWQIEGNNTTLTPSTPVTLIYN
str.CCUG45777  IKTDELEGSINLVGAQFDDLLLKKYRLTVDKKSPEIALLNPKGFTTTYLAEFGFTSSSLSAKALPQSDTQWQIEGNNTTLTPSTPVTLIYN
str.JK56       IKTDELEGSINLVGAQFDDLLLKKYRLTVDKKSPEIALLNPKGFTTTYLAEFGFTSSSLSAKALPQSDTQWQIEGNNTTLTPSTPVTLIYN
str.JK67       IKTDELEGSINLVGAQFDDLLLKKYRLTVDKKSPEIALLNPKGFTTTYLAEFGFTSSSLSAKALPQSDTQWQIEGNNTTLTPSTPVTLIYN
str.JK19       IKTDELEGSINLVGAQFDDLLLKKYRLTVDKKSPEIALLNPKGFTTTYLAEFGFTSSSLSAKALPQSDTQWQIEGNNTTLTPSTPVTLIYN
str.JK63       IKTDELEGSINLVGAQFDDLLLKKYRLTVDKKSPEIALLNPKGFTTTYLAEFGFTSSSLSAKALPQSDTQWQIEGNNTTLTPSTPVTLIYN
str.JK39       IKTDELEGSINLVGAQFDDLLLKKYRLTVDKKSPEIALLNPKGFTTTYLAEFGFTSSSLSAKALPQSDTQWQIEGNNTTLTPSTPVTLIYN
str.JK68       IKTDELEGSINLVGAQFDDLLLKKYRLTVDKKSPEIALLNPKGFTTTYLAEFGFTSSSLSAKALPQSDTQWQIEGNNTTLTPSTPVTLIYN
str.G1713      IKTDELEGSINLVGAQFDDLLLKKYRLTVDKKSPEIALLNPKGFTTTYLAEFGFTSSSLSAKALPQSDTQWQIEGNNTTLTPSTPVTLIYN
str.G1712      IKTDELEGSINLVGAQFDDLLLKKYRLTVDKKSPEIALLNPKGFTTTYLAEFGFTSSSLSAKALPQSDTQWQIEGNNTTLTPSTPVTLIYN
str.JK73       IKTDELEGSINLVGAQFDDLLLKKYRLTVDKKSPEIALLNPKGFTTTYLAEFGFTSSSLSAKALPQSDTQWQIEGNNTTLTPSTPVTLIYN
str.JK73rel    IKTDELEGSINLVGAQFDDLLLKKYRLTVDKKSPEIALLNPKGFTTTYLAEFGFTSSSLSAKALPQSDTQWQIEGNNTTLTPSTPVTLIYN
str.CO20_0321  IKTDELEGSINLVGAQFDDLLLKKYRLTVDKKSPEIALLNPKGFTTTYLAEFGFTSSSLSAKALPQSDTQWQIEGNNTTLTPSTPVTLIYN
str.CO21_0024  IKTDELEGSINLVGAQFDDLLLKKYRLTVDKKSPEIALLNPKGFTTTYLAEFGFTSSSLSAKALPQSDTQWQIEGNNTTLTPSTPVTLIYN
str.CO20_0297  IKTDELEGSINLVGAQFDDLLLKKYRLTVDKKSPEIALLNPKGFTTTYLAEFGFTSSSLSAKALPQSDTQWQIEGNNTTLTPSTPVTLIYN
str.JK31       IKTDELEGSINLVGAQFDDLLLKKYRLTVDKKSPEIALLNPKGFTTTYLAEFGFTSSSLSAKALPQSDTQWQIEGNNTTLTPSTPVTLIYN
str.BQ2-D70    IKTDELEGSINLVGAQFDDLLLKKYRLTVDKKSPEIALLNPKGFTTTYLAEFGFTSSSLSAKALPQSDTQWQIEGNNTTLTPSTPVTLIYN
str.CO20_0256  IKTDELEGSINLVGAQFDDLLLKKYRLTVDKKSPEIALLNPKGFTTTYLAEFGFTSSSLSAKALPQSDTQWQIEGNNTTLTPSTPVTLIYN
str.CO20_0257  IKTDELEGSINLVGAQFDDLLLKKYRLTVDKKSPEIALLNPKGFTTTYLAEFGFTSSSLSAKALPQSDTQWQIEGNNTTLTPSTPVTLIYN
str.MF1-1      IKTDELEGSINLVGAQFDDLLLKKYRLTVDKKSPEIALLNPKGFTTTYLAEFGFTSSSLSAKALPQSDTQWQIEGNNTTLTPSTPVTLIYN
str.RM-11      IKTDELEGSINLVGAQFDDLLLKKYRLTVDKKSPEIALLNPKGFTTTYLAEFGFTSSSLSAKALPQSDTQWQIEGNNTTLTPSTPVTLIYN
str.NCTC12899  IKTDELEGSINLVGAQFDDLLLKKYRLTVDKKSPEIALLNPKGFTTTYLAEFGFTSSSLSAKSLPQSDTQWQIEGNNTTLTPSTPVTLIYN
str.JK12       IKTDELEGSINLVGAQFDDLLLKKYRLTVDKKSPEIALLNPKGFTTTYLAEFGFTSSSLSAKSLPQSDTQWQIEGNNTTLTPSTPVTLIYN
str.JK7        IKTDELEGSINLVGAQFDDLLLKKYRLTVDKKSPEIALLNPKGFTTTYLAEFGFTSSSLSAKSLPQSDTQWQIEGNNTTLTPSTPVTLIYN

                     190       200       210       220       230       240       250       260       270
str.Toulouse   NGQGQIFRRTLSVDNHYMFTIEDSIKNESDKPIYLSSYARVARAAPPEHTNATYLLHEGMIGIASDSLKTEKYKTLAELNPNPDNSQKSIT
str.CCUG45777  NGQGQIFRRTLSVDNHYMFTIEDSIKNESDKPIYLSSYARVARAAPPEHTNATYLLHEGMIGIASDSLKTEKYKTLAELNPNPDNSQKSIT
str.JK56       NGQGQIFRRTLSVDNHYMFTIEDSIKNESDKPIYLSSYARVARAAPPEHTNATYLLHEGMIGIASDSLKTEKYKTLAELNPNPDNSQKSIT
str.JK67       NGQGQIFRRTLSVDNHYMFTIEDSIKNESDKPIYLSSYARVARAAPPEHTNATYLLHEGMIGIASDSLKTEKYKTLAELNPNPDNSQKSIT
str.JK19       NGQGQIFRRTLSVDNHYMFTIEDSIKNESDKPIYLSSYARVARAAPPEHTNATYLLHEGMIGIASDSLKTEKYKTLAELNPNPDNSQKSIT
str.JK63       NGQGQIFRRTLSVDNHYMFTIEDSIKNESDKPIYLSSYARVARAAPPEHTNATYLLHEGMIGIASDSLKTEKYKTLAELNPNPDNSQKSIT
str.JK39       NGQGQIFRRTLSVDNHYMFTIEDSIKNESDKPIYLSSYARVARAAPPEHTNATYLLHEGMIGIASDSLKTEKYKTLAELNPNPDNSQKSIT
str.JK68       NGQGQIFRRTLSVDNHYMFTIEDSIKNESDKPIYLSSYARVARAAPPEHTNATYLLHEGMIGIASDSLKTEKYKTLAELNPNPDNSQKSIT
str.G1713      NGQGQIFRRTLSVDNHYMFTIEDSIKNESDKPIYLSSYARVARAAPPEHTNATYLLHEGMIGIASDSLKTEKYKTLAELNPNPDNSQKSIT
str.G1712      NGQGQIFRRTLSVDNHYMFTIEDSIKNESDKPIYLSSYARVARAAPPEHTNATYLLHEGMIGIASDSLKTEKYKTLAELNPNPDNSQKSIT
str.JK73       NGQGQIFRRTLSVDNHYMFTIEDSIKNESDKPIYLSSYARVARAAPPEHTNATYLLHEGMIGIASDSLKTEKYKTLAELNPNPDNSQKSIT
str.JK73rel    NGQGQIFRRTLSVDNHYMFTIEDSIKNESDKPIYLSSYARVARAAPPEHTNATYLLHEGMIGIASDSLKTEKYKTLAELNPNPDNSQKSIT
str.CO20_0321  NGQGQIFRRTLSVDNHYMFTIEDSIKNESDKPIYLSSYARVARAAPPEHTNATYLLHEGMIGIASDSLKTEKYKTLAELNPNPDNSQKSIT
str.CO21_0024  NGQGQIFRRTLSVDNHYMFTIEDSIKNESDKPIYLSSYARVARAAPPEHTNATYLLHEGMIGIASDSLKTEKYKTLAELNPNPDNSQKSIT
str.CO20_0297  NGQGQIFRRTLSVDNHYMFTIEDSIKNESDKPIYLSSYARVARAAPPEHTNATYLLHEGMIGIASDSLKTEKYKTLAELNPNPDNSQKSIT
str.JK31       NGQGQIFRRTLSVDNHYMFTIEDSIKNESDKPIYLSSYARVARAAPPEHTNATYLLHEGMIGIASDSLKTEKYKTLAELNPNPDNSQKSIT
str.BQ2-D70    NGQGQIFRRTLSVDNHYMFTIEDSIKNESDKPIYLSSYARVARAAPPEHTNATYLLHEGMIGIASDSLKTEKYKTLAELNPNPDNSQKSIT
str.CO20_0256  NGQGQIFRRTLSVDNHYMFTIEDSIKNESDKPIYLSSYARVARAAPPEHTNATYLLHEGMIGIASDSLKTEKYKTLAELNPNPDNSQKSIT
str.CO20_0257  NGQGQIFRRTLSVDNHYMFTIEDSIKNESDKPIYLSSYARVARAAPPEHTNATYLLHEGMIGIASDSLKTEKYKTLAELNPNPDNSQKSIT
str.MF1-1      NGQGQIFRRTLSVDNHYMFTIEDSIKNESDKPIYLSSYARVARAAPPEHTNATYLLHEGMIGIAGDSLKTEKYKTLAELNPNPDNSQKSIT
str.RM-11      NGQGQIFRRTLSVDNHYMFTIEDSIKNESDKPIYLSSYARVARAAPPEHTNATYLLHEGMIGIAGDSLKTEKYKTLAELNPNPDNSQKSIT
str.NCTC12899  NGQGQIFRRTLSVDNHYMFTIEDSIKNESDKPIYLSSYARVARAAPPEHTNATYLLHEGMIGIAGDSLKTEKYKTLAELNPNPDNSQKSIT
str.JK12       NGQGQIFRRTLSVDNHYMFTIEDSIKNESDKPIYLSSYARVARAAPPEHTNATYLLHEGMIGIAGDSLKTEKYKTLAELNPNPDNSQKSIT
str.JK7        NGQGQIFRRTLSVDNHYMFTIEDSIKNESDKPIYLSSYARVARAAPPEHTNATYLLHEGMIGIAGDSLKTEKYKTLAELNPNPDNSQKSIT

                    280       290       300       310       320       330       340       350       360
str.Toulouse   FSKNIGGWIGITDKYWAVAVIPPQDKEYTSRFIYFDRLNTHYQSDLLGSLLTVAPNETKIVTNRLFAGAKQVEIINHYQNDLKIKKFALLI
str.CCUG45777  FSKNIGGWIGITDKYWAVAVIPPQDKEYTSRFIYFDRLNTHYQSDLLGSLLTVAPNETKIVTNRLFAGAKQVEIINHYQNDLKIKKFALLI
str.JK56       FSKNIGGWIGITDKYWAVAVIPPQDKEYTSRFIYFDRLNTHYQSDLLGSLLTVAPNETKIVTNRLFAGAKQVEIINHYQNDLKIKKFALLI
str.JK67       FSKNIGGWIGITDKYWAVAVIPPQDKEYTSRFIYFDRLNTHYQSDLLGSLLTVAPNETKIVTNRLFAGAKQVEIINHYQNDLKIKKFALLI
str.JK19       FSKNIGGWIGITDKYWAVAVIPPQDKEYTSRFIYFDRLNTHYQSDLLGSLLTVAPNETKIVTNRLFAGAKQVEIINHYQNDLKIKKFALLI
str.JK63       FSKNIGGWIGITDKYWAVAVIPPQDKEYTSRFIYFDRLNTHYQSDLLGSLLTVAPNETKIVTNRLFAGAKQVEIINHYQNDLKIKKFALLI
str.JK39       FSKNIGGWIGITDKYWAVAVIPPQDKEYTSRFIYFDRLNTHYQSDLLGSLLTVAPNETKIVTNRLFAGAKQVEIINHYQNDLKIKKFALLI
str.JK68       FSKNIGGWIGITDKYWAVAVIPPQDKEYTSRFIYFDRLNTHYQSDLLGSLLTVAPNETKIVTNRLFAGAKQVEIINHYQNDLKIKKFALLI
str.G1713      FSKNIGGWIGITDKYWAVAVIPPQDKEYTSRFIYFDRLNTHYQSDLLGSLLTVAPNETKIVTNRLFAGAKQVEIINHYQNDLKIKKFALLI
str.G1712      FSKNIGGWIGITDKYWAVAVIPPQDKEYTSRFIYFDRLNTHYQSDLLGSLLTVAPNETKIVTNRLFAGAKQVEIINHYQNDLKIKKFALLI
str.JK73       FSKNIGGWIGITDKYWAVAVIPPQDKEYTSRFIYFDRLNTHYQSDLLGSLLTVAPNETKIVTNRLFAGAKQVEIINHYQNDLKIKKFALLI
str.JK73rel    FSKNIGGWIGITDKYWAVAVIPPQDKEYTSRFIYFDRLNTHYQSDLLGSLLTVAPNETKIVTNRLFAGAKQVEIINHYQNDLKIKKFALLI
str.CO20_0321  FSKNIGGWIGITDKYWAVAVIPPQDKEYTSRFIYFDRLNTHYQSDLLGSLLTVAPNETKIVTNRLFAGAKQVEIINHYQNDLKIKKFALLI
str.CO21_0024  FSKNIGGWIGITDKYWAVAVIPPQDKEYTSRFIYFDRLNTHYQSDLLGSLLTVAPNETKIVTNRLFAGAKQVEIINHYQNDLKIKKFALLI
str.CO20_0297  FSKNIGGWIGITDKYWAVAVIPPQDKEYTSRFIYFDRLNTHYQSDLLGSLLTVAPNETKIVTNRLFAGAKQVEIINHYQNDLKIKKFALLI
str.JK31       FSKNIGGWIGITDKYWAVAVIPPQDKEYTSRFIYFDRLNTHYQSDLLGSLLTVAPNETKIVTNRLFAGAKQVEIINHYQNDLKIKKFALLI
str.BQ2-D70    FSKNIGGWIGITDKYWAVAVIPPQDKEYTSRFIYFDRLNTHYQSDLLGSLLTVAPNETKIVTNRLFAGAKQVEIINHYQNDLKIKKFALLI
str.CO20_0256  FSKNIGGWIGITDKYWAVAVIPPQDKEYTSRFIYFDRLNTHYQSDLLGSLLTVAPNETKIVTNRLFAGAKQVEIINHYQNDLKIKKFALLI
str.CO20_0257  FSKNIGGWIGITDKYWAVAVIPPQDKEYTSRFIYFDRLNTHYQSDLLGSLLTVAPNETKIVTNRLFAGAKQVEIINHYQNDLKIKKFALLI
str.MF1-1      FSKNIGGWIGITDKYWAVAVIPPQDKEYTSRFIYFDRLNTHYQSDLLGSLLTVAPNETKIVTNRLFAGAKQVEIINHYQNDLKIKKFALLI
str.RM-11      FSKNIGGWIGITDKYWAVAVIPPQDKEYTSRFIYFDRLNTHYQSDLLGSLLTVAPNETKIVTNRLFAGAKQVEIINHYQNDLKIKKFALLI
str.NCTC12899  FSKNIGGWIGITDKYWAVAVIPPQDKEYTSRFIYFDRLNTHYQSDLLGSLLTVAPNETKIVTNRLFAGAKQVEIINHYQNDLKIKKFALLI
str.JK12       FSKNIGGWIGITDKYWAVAVIPPQDKEYTSRFIYFDRLNTHYQSDLLGSLLTVAPNETKIVTNRLFAGAKQVEIINHYQNDLKIKKFALLI
str.JK7        FSKNIGGWIGITDKYWAVAVIPPQDKEYTSRFIYFDRLNTHYQSDLLGSLLTVAPNETKIVTNRLFAGAKQVEIINHYQNDLKIKKFALLI
```

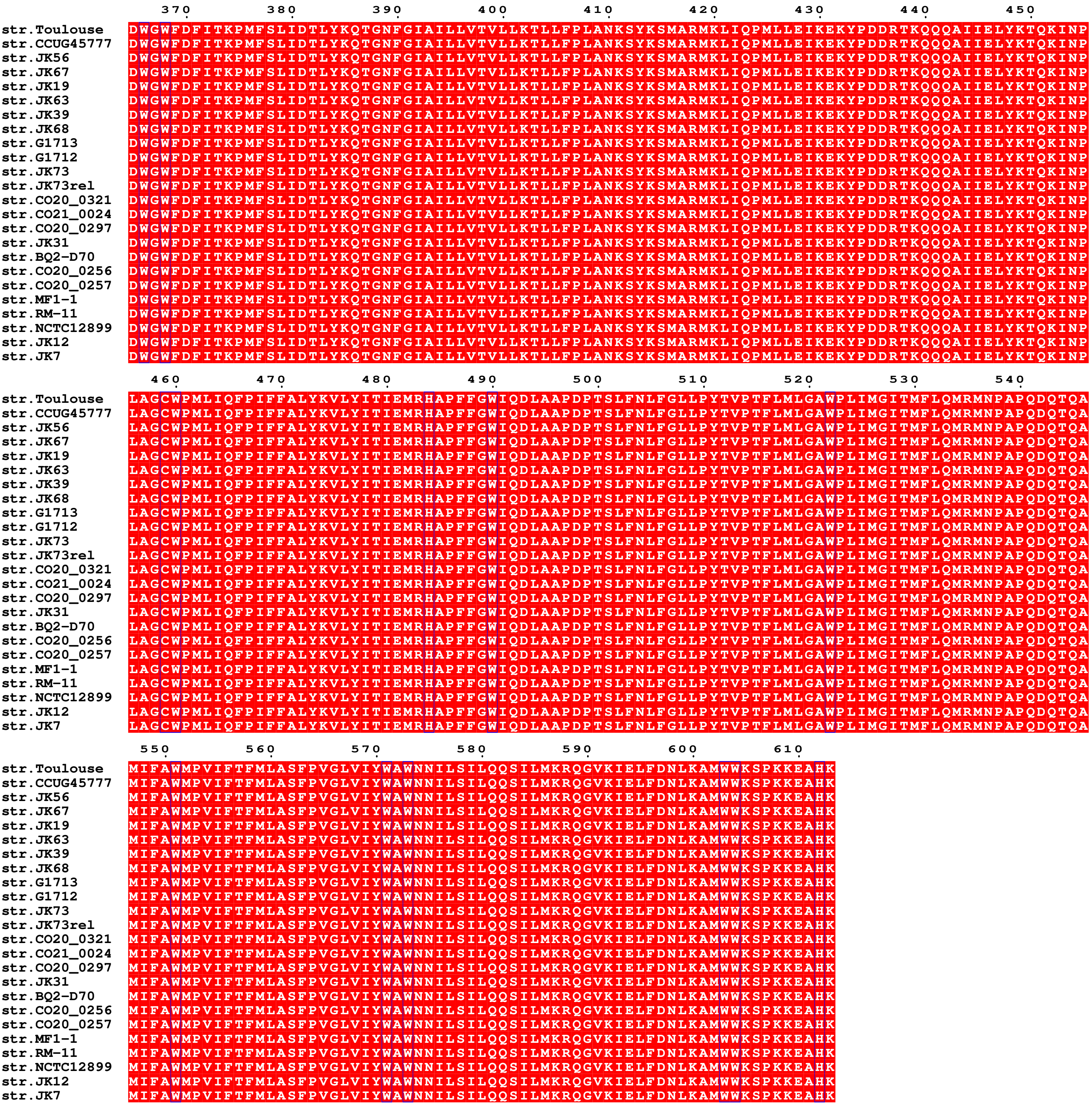
370 380 390 400 410 420 430 440 450
str.Toulouse DWGWFDFITKPMFSLIDTLYKQTGNFGIAILLVTVLLKTLLFPLANKSYKSMARMKLIQPMLLEIKEKYPDDRTKQQQAIIELYKTQKINP
str.CCUG45777 DWGWFDFITKPMFSLIDTLYKQTGNFGIAILLVTVLLKTLLFPLANKSYKSMARMKLIQPMLLEIKEKYPDDRTKQQQAIIELYKTQKINP
str.JK56 DWGWFDFITKPMFSLIDTLYKQTGNFGIAILLVTVLLKTLLFPLANKSYKSMARMKLIQPMLLEIKEKYPDDRTKQQQAIIELYKTQKINP
str.JK67 DWGWFDFITKPMFSLIDTLYKQTGNFGIAILLVTVLLKTLLFPLANKSYKSMARMKLIQPMLLEIKEKYPDDRTKQQQAIIELYKTQKINP
str.JK19 DWGWFDFITKPMFSLIDTLYKQTGNFGIAILLVTVLLKTLLFPLANKSYKSMARMKLIQPMLLEIKEKYPDDRTKQQQAIIELYKTQKINP
str.JK63 DWGWFDFITKPMFSLIDTLYKQTGNFGIAILLVTVLLKTLLFPLANKSYKSMARMKLIQPMLLEIKEKYPDDRTKQQQAIIELYKTQKINP
str.JK39 DWGWFDFITKPMFSLIDTLYKQTGNFGIAILLVTVLLKTLLFPLANKSYKSMARMKLIQPMLLEIKEKYPDDRTKQQQAIIELYKTQKINP
str.JK68 DWGWFDFITKPMFSLIDTLYKQTGNFGIAILLVTVLLKTLLFPLANKSYKSMARMKLIQPMLLEIKEKYPDDRTKQQQAIIELYKTQKINP
str.G1713 DWGWFDFITKPMFSLIDTLYKQTGNFGIAILLVTVLLKTLLFPLANKSYKSMARMKLIQPMLLEIKEKYPDDRTKQQQAIIELYKTQKINP
str.G1712 DWGWFDFITKPMFSLIDTLYKQTGNFGIAILLVTVLLKTLLFPLANKSYKSMARMKLIQPMLLEIKEKYPDDRTKQQQAIIELYKTQKINP
str.JK73 DWGWFDFITKPMFSLIDTLYKQTGNFGIAILLVTVLLKTLLFPLANKSYKSMARMKLIQPMLLEIKEKYPDDRTKQQQAIIELYKTQKINP
str.JK73rel DWGWFDFITKPMFSLIDTLYKQTGNFGIAILLVTVLLKTLLFPLANKSYKSMARMKLIQPMLLEIKEKYPDDRTKQQQAIIELYKTQKINP
str.CO20_0321 DWGWFDFITKPMFSLIDTLYKQTGNFGIAILLVTVLLKTLLFPLANKSYKSMARMKLIQPMLLEIKEKYPDDRTKQQQAIIELYKTQKINP
str.CO21_0024 DWGWFDFITKPMFSLIDTLYKQTGNFGIAILLVTVLLKTLLFPLANKSYKSMARMKLIQPMLLEIKEKYPDDRTKQQQAIIELYKTQKINP
str.CO20_0297 DWGWFDFITKPMFSLIDTLYKQTGNFGIAILLVTVLLKTLLFPLANKSYKSMARMKLIQPMLLEIKEKYPDDRTKQQQAIIELYKTQKINP
str.JK31 DWGWFDFITKPMFSLIDTLYKQTGNFGIAILLVTVLLKTLLFPLANKSYKSMARMKLIQPMLLEIKEKYPDDRTKQQQAIIELYKTQKINP
str.BQ2-D70 DWGWFDFITKPMFSLIDTLYKQTGNFGIAILLVTVLLKTLLFPLANKSYKSMARMKLIQPMLLEIKEKYPDDRTKQQQAIIELYKTQKINP
str.CO20_0256 DWGWFDFITKPMFSLIDTLYKQTGNFGIAILLVTVLLKTLLFPLANKSYKSMARMKLIQPMLLEIKEKYPDDRTKQQQAIIELYKTQKINP
str.CO20_0257 DWGWFDFITKPMFSLIDTLYKQTGNFGIAILLVTVLLKTLLFPLANKSYKSMARMKLIQPMLLEIKEKYPDDRTKQQQAIIELYKTQKINP
str.MF1-1 DWGWFDFITKPMFSLIDTLYKQTGNFGIAILLVTVLLKTLLFPLANKSYKSMARMKLIQPMLLEIKEKYPDDRTKQQQAIIELYKTQKINP
str.RM-11 DWGWFDFITKPMFSLIDTLYKQTGNFGIAILLVTVLLKTLLFPLANKSYKSMARMKLIQPMLLEIKEKYPDDRTKQQQAIIELYKTQKINP
str.NCTC12899 DWGWFDFITKPMFSLIDTLYKQTGNFGIAILLVTVLLKTLLFPLANKSYKSMARMKLIQPMLLEIKEKYPDDRTKQQQAIIELYKTQKINP
str.JK12 DWGWFDFITKPMFSLIDTLYKQTGNFGIAILLVTVLLKTLLFPLANKSYKSMARMKLIQPMLLEIKEKYPDDRTKQQQAIIELYKTQKINP
str.JK7 DWGWFDFITKPMFSLIDTLYKQTGNFGIAILLVTVLLKTLLFPLANKSYKSMARMKLIQPMLLEIKEKYPDDRTKQQQAIIELYKTQKINP
460 470 480 490 500 510 520 530 540
str.Toulouse LAGCWPMLIQFPIFFALYKVLYITIEMRHAPFFGWIQDLAAPDPTSLFNLFGLLPYTVPTFLMLGAWPLIMGITMFLQMRMNPAPQDQTQA
str.CCUG45777 LAGCWPMLIQFPIFFALYKVLYITIEMRHAPFFGWIQDLAAPDPTSLFNLFGLLPYTVPTFLMLGAWPLIMGITMFLQMRMNPAPQDQTQA
str.JK56 LAGCWPMLIQFPIFFALYKVLYITIEMRHAPFFGWIQDLAAPDPTSLFNLFGLLPYTVPTFLMLGAWPLIMGITMFLQMRMNPAPQDQTQA
str.JK67 LAGCWPMLIQFPIFFALYKVLYITIEMRHAPFFGWIQDLAAPDPTSLFNLFGLLPYTVPTFLMLGAWPLIMGITMFLQMRMNPAPQDQTQA
str.JK19 LAGCWPMLIQFPIFFALYKVLYITIEMRHAPFFGWIQDLAAPDPTSLFNLFGLLPYTVPTFLMLGAWPLIMGITMFLQMRMNPAPQDQTQA
str.JK63 LAGCWPMLIQFPIFFALYKVLYITIEMRHAPFFGWIQDLAAPDPTSLFNLFGLLPYTVPTFLMLGAWPLIMGITMFLQMRMNPAPQDQTQA
str.JK39 LAGCWPMLIQFPIFFALYKVLYITIEMRHAPFFGWIQDLAAPDPTSLFNLFGLLPYTVPTFLMLGAWPLIMGITMFLQMRMNPAPQDQTQA
str.JK68 LAGCWPMLIQFPIFFALYKVLYITIEMRHAPFFGWIQDLAAPDPTSLFNLFGLLPYTVPTFLMLGAWPLIMGITMFLQMRMNPAPQDQTQA
str.G1713 LAGCWPMLIQFPIFFALYKVLYITIEMRHAPFFGWIQDLAAPDPTSLFNLFGLLPYTVPTFLMLGAWPLIMGITMFLQMRMNPAPQDQTQA
str.G1712 LAGCWPMLIQFPIFFALYKVLYITIEMRHAPFFGWIQDLAAPDPTSLFNLFGLLPYTVPTFLMLGAWPLIMGITMFLQMRMNPAPQDQTQA
str.JK73 LAGCWPMLIQFPIFFALYKVLYITIEMRHAPFFGWIQDLAAPDPTSLFNLFGLLPYTVPTFLMLGAWPLIMGITMFLQMRMNPAPQDQTQA
str.JK73rel LAGCWPMLIQFPIFFALYKVLYITIEMRHAPFFGWIQDLAAPDPTSLFNLFGLLPYTVPTFLMLGAWPLIMGITMFLQMRMNPAPQDQTQA
str.CO20_0321 LAGCWPMLIQFPIFFALYKVLYITIEMRHAPFFGWIQDLAAPDPTSLFNLFGLLPYTVPTFLMLGAWPLIMGITMFLQMRMNPAPQDQTQA
str.CO21_0024 LAGCWPMLIQFPIFFALYKVLYITIEMRHAPFFGWIQDLAAPDPTSLFNLFGLLPYTVPTFLMLGAWPLIMGITMFLQMRMNPAPQDQTQA
str.CO20_0297 LAGCWPMLIQFPIFFALYKVLYITIEMRHAPFFGWIQDLAAPDPTSLFNLFGLLPYTVPTFLMLGAWPLIMGITMFLQMRMNPAPQDQTQA
str.JK31 LAGCWPMLIQFPIFFALYKVLYITIEMRHAPFFGWIQDLAAPDPTSLFNLFGLLPYTVPTFLMLGAWPLIMGITMFLQMRMNPAPQDQTQA
str.BQ2-D70 LAGCWPMLIQFPIFFALYKVLYITIEMRHAPFFGWIQDLAAPDPTSLFNLFGLLPYTVPTFLMLGAWPLIMGITMFLQMRMNPAPQDQTQA
str.CO20_0256 LAGCWPMLIQFPIFFALYKVLYITIEMRHAPFFGWIQDLAAPDPTSLFNLFGLLPYTVPTFLMLGAWPLIMGITMFLQMRMNPAPQDQTQA
str.CO20_0257 LAGCWPMLIQFPIFFALYKVLYITIEMRHAPFFGWIQDLAAPDPTSLFNLFGLLPYTVPTFLMLGAWPLIMGITMFLQMRMNPAPQDQTQA
str.MF1-1 LAGCWPMLIQFPIFFALYKVLYITIEMRHAPFFGWIQDLAAPDPTSLFNLFGLLPYTVPTFLMLGAWPLIMGITMFLQMRMNPAPQDQTQA
str.RM-11 LAGCWPMLIQFPIFFALYKVLYITIEMRHAPFFGWIQDLAAPDPTSLFNLFGLLPYTVPTFLMLGAWPLIMGITMFLQMRMNPAPQDQTQA
str.NCTC12899 LAGCWPMLIQFPIFFALYKVLYITIEMRHAPFFGWIQDLAAPDPTSLFNLFGLLPYTVPTFLMLGAWPLIMGITMFLQMRMNPAPQDQTQA
str.JK12 LAGCWPMLIQFPIFFALYKVLYITIEMRHAPFFGWIQDLAAPDPTSLFNLFGLLPYTVPTFLMLGAWPLIMGITMFLQMRMNPAPQDQTQA
str.JK7 LAGCWPMLIQFPIFFALYKVLYITIEMRHAPFFGWIQDLAAPDPTSLFNLFGLLPYTVPTFLMLGAWPLIMGITMFLQMRMNPAPQDQTQA
550 560 570 580 590 600 610
str.Toulouse MIFAWMPVIFTFMLASFPVGLVIYWAWNNILSILQQSILMKRQGVKIELFDNLKAMWWKSPKKEAHK
str.CCUG45777 MIFAWMPVIFTFMLASFPVGLVIYWAWNNILSILQQSILMKRQGVKIELFDNLKAMWWKSPKKEAHK
str.JK56 MIFAWMPVIFTFMLASFPVGLVIYWAWNNILSILQQSILMKRQGVKIELFDNLKAMWWKSPKKEAHK
str.JK67 MIFAWMPVIFTFMLASFPVGLVIYWAWNNILSILQQSILMKRQGVKIELFDNLKAMWWKSPKKEAHK
str.JK19 MIFAWMPVIFTFMLASFPVGLVIYWAWNNILSILQQSILMKRQGVKIELFDNLKAMWWKSPKKEAHK
str.JK63 MIFAWMPVIFTFMLASFPVGLVIYWAWNNILSILQQSILMKRQGVKIELFDNLKAMWWKSPKKEAHK
str.JK39 MIFAWMPVIFTFMLASFPVGLVIYWAWNNILSILQQSILMKRQGVKIELFDNLKAMWWKSPKKEAHK
str.JK68 MIFAWMPVIFTFMLASFPVGLVIYWAWNNILSILQQSILMKRQGVKIELFDNLKAMWWKSPKKEAHK
str.G1713 MIFAWMPVIFTFMLASFPVGLVIYWAWNNILSILQQSILMKRQGVKIELFDNLKAMWWKSPKKEAHK
str.G1712 MIFAWMPVIFTFMLASFPVGLVIYWAWNNILSILQQSILMKRQGVKIELFDNLKAMWWKSPKKEAHK
str.JK73 MIFAWMPVIFTFMLASFPVGLVIYWAWNNILSILQQSILMKRQGVKIELFDNLKAMWWKSPKKEAHK
str.JK73rel MIFAWMPVIFTFMLASFPVGLVIYWAWNNILSILQQSILMKRQGVKIELFDNLKAMWWKSPKKEAHK
str.CO20_0321 MIFAWMPVIFTFMLASFPVGLVIYWAWNNILSILQQSILMKRQGVKIELFDNLKAMWWKSPKKEAHK
str.CO21_0024 MIFAWMPVIFTFMLASFPVGLVIYWAWNNILSILQQSILMKRQGVKIELFDNLKAMWWKSPKKEAHK
str.CO20_0297 MIFAWMPVIFTFMLASFPVGLVIYWAWNNILSILQQSILMKRQGVKIELFDNLKAMWWKSPKKEAHK
str.JK31 MIFAWMPVIFTFMLASFPVGLVIYWAWNNILSILQQSILMKRQGVKIELFDNLKAMWWKSPKKEAHK
str.BQ2-D70 MIFAWMPVIFTFMLASFPVGLVIYWAWNNILSILQQSILMKRQGVKIELFDNLKAMWWKSPKKEAHK
str.CO20_0256 MIFAWMPVIFTFMLASFPVGLVIYWAWNNILSILQQSILMKRQGVKIELFDNLKAMWWKSPKKEAHK
str.CO20_0257 MIFAWMPVIFTFMLASFPVGLVIYWAWNNILSILQQSILMKRQGVKIELFDNLKAMWWKSPKKEAHK
str.MF1-1 MIFAWMPVIFTFMLASFPVGLVIYWAWNNILSILQQSILMKRQGVKIELFDNLKAMWWKSPKKEAHK
str.RM-11 MIFAWMPVIFTFMLASFPVGLVIYWAWNNILSILQQSILMKRQGVKIELFDNLKAMWWKSPKKEAHK
str.NCTC12899 MIFAWMPVIFTFMLASFPVGLVIYWAWNNILSILQQSILMKRQGVKIELFDNLKAMWWKSPKKEAHK
str.JK12 MIFAWMPVIFTFMLASFPVGLVIYWAWNNILSILQQSILMKRQGVKIELFDNLKAMWWKSPKKEAHK
str.JK7 MIFAWMPVIFTFMLASFPVGLVIYWAWNNILSILQQSILMKRQGVKIELFDNLKAMWWKSPKKEAHK

>WP_011179680.1 4-hydroxy-tetrahydrodipicolinate reductase [Bartonella quintana str. Toulouse]

```
              1        10        20        30        40        50        60        70        80        90
str.Toulouse   MRLTVVGANGKMGRELITAIQRREDVELCAVLVRKGSPFVDKDASILTGSDFLNIRITDDPENAFSNTEGILDFSQPQASILYANYAAQKS
str.JK73rel    MRLTVVGANGKMGRELITAIQRREDVELCAVLVRKGSPFVDKDASILTGSDFLNIRITDDPENAFSNTEGILDFSQPQASILYANYAAQKS
str.JK73       MRLTVVGANGKMGRELITAIQRREDVELCAVLVRKGSPFVDKDASILTGSDFLNIRITDDPENAFSNTEGILDFSQPQASILYANYAAQKS
str.CCUG45777  MRLTVVGANGKMGRELITAIQRREDVELCAVLVRKGSPFVDKDASILTGSDFLNIRITDDPENAFSNTEGILDFSQPQASILYANYAAQKS
str.JK67       MRLTVVGANGKMGRELITAIQRREDVELCAVLVRKGSPFVDKDASILTGSDFLNIRITDDPENAFSNTEGILDFSQPQASILYANYAAQKS
str.JK12       MRLTVVGANGKMGRELITAIQRREDVELCAVLVRKGSPFVDKDASILTGSDFLNIRITDDPENAFSNTEGILDFSQPQASILYANYAAQKS
str.G1712      MRLTVVGANGKMGRELITAIQRREDVELCAVLVRKGSPFVDKDASILTGSDFLNIRITDDPENAFSNTEGILDFSQPQASILYANYAAQKS
str.G1732      MRLTVVGANGKMGRELITAIQRREDVELCAVLVRKGSPFVDKDASILTGSDFLNIRITDDPENAFSNTEGILDFSQPQASILYANYAAQKS
str.CO20_0297  MRLTVVGANGKMGRELITAIQRREDVELCAVLVRKGSPFVDKDASILTGSDFLNIRITDDPENAFSNTEGILDFSQPQASILYANYAAQKS
str.CO21_0024  MRLTVVGANGKMGRELITAIQRREDVELCAVLVRKGSPFVDKDASILTGSDFLNIRITDDPENAFSNTEGILDFSQPQASILYANYAAQKS
str.CO20_0257  MRLTVVGANGKMGRELITAIQRREDVELCAVLVRKGSPFVDKDASILTGSDFLNIRITDDPENAFSNTEGILDFSQPQASILYANYAAQKS
str.CO20_0321  MRLTVVGANGKMGRELITAIQRREDVELCAVLVRKGSPFVDKDASILTGSDFLNIRITDDPENAFSNTEGILDFSQPQASILYANYAAQKS
str.CO20_0256  MRLTVVGANGKMGRELITAIQRREDVELCAVLVRKGSPFVDKDASILTGSDFLNIRITDDPENAFSNTEGILDFSQPQASILYANYAAQKS
str.JK31       MRLTVVGANGKMGRELITAIQRREDVELCAVLVRKGSPFVDKDASILTGSDFLNIRITDDPENAFSNTEGILDFSQPQASILYANYAAQKS
str.NCTC12899  MRLTVVGANGKMGRELITAIQRREDVELCAVLVRKGSPFVDKDASILTGSDFLNIRITDDPENAFSNTEGILDFSQPQASILYANYAAQKS
str.JK63       MRLTVVGANGKMGRELITAIQRREDVELCAVLVRKGSPFVDKDASILTGSDFLNIRITDDPENAFSNTEGILDFSQPQASILYANYAAQKS
str.JK68       MRLTVVGANGKMGRELITAIQRREDVELCAVLVRKGSPFVDKDASILTGSDFLNIRITDDPENAFSNTEGILDFSQPQASILYANYAAQKS
str.JK39       MRLTVVGANGKMGRELITAIQRREDVELCAVLVRKGSPFVDKDASILTGSDFLNIRITDDPENAFSNTEGILDFSQPQASILYANYAAQKS
str.JK19       MRLTVVGANGKMGRELITAIQRREDVELCAVLVRKGSPFVDKDASILTGSDFLNIRITDDPENAFSNTEGILDFSQPQASILYANYAAQKS
str.JK7        MRLTVVGANGKMGRELITAIQRREDVELCAVLVRKGSPFVDKDASILTGSDFLNIRITDDPENAFSNTEGILDFSQPQASILYANYAAQKS
str.BQ2-D70    MRLTVVGANGKMGRELITAIQRREDVELCAVLVRKGSPFVDKDASILTGSDFLNIRITDDPENAFSNTEGILDFSQPQASILYANYAAQKS
str.JK56       MRLTVVGANGKMGRELITAIQRREDVELCAVLVRKGSPFVDKDASILTGSDFLNIRITDDPENAFSNTEGILDFSQPQASILYANYAAQKS
str.RM-11      MRLTVVGANGKMGRELITAIQRREDVELCAVLVRKGSPFVDKDASLLTGSDFLNIRITDDPENAFSNTEGILDFSQPQASLLYANYAAQKS
str.MF1-1      MRLTVVGANGKMGRELITAIQRREDVELCTVLVRKGSPFVDKDASLLTGSDFLNIRITDDPENAFSNTEGILDFSQPQASLLYANYAAQKS

                     100       110       120       130       140       150       160       170       180
str.Toulouse   LVHIIGTTGFSKTEEEKIADFAKDTTIVKSGNMSLGVNLLASLVKKAAKALEVDDFDIEIYEMHHSGKVDAPSGTALLLGQAAAEGRNVML
str.JK73rel    LVHIIGTTGFSKTEEEKIADFAKDTTIVKSGNMSLGVNLLANLVKKAAKALEVDDFDIEIYEMHHSGKVDAPSGTALLLGQAAAEGRNVML
str.JK73       LVHIIGTTGFSKTEEEKIADFAKDTTIVKSGNMSLGVNLLANLVKKAAKALEVDDFDIEIYEMHHSGKVDAPSGTALLLGQAAAEGRNVML
str.CCUG45777  LVHIIGTTGFSKTEEEKIADFAKDTTIVKSGNMSLGVNLLASLVKKAAKALEVDDFDIEIYEMHHSGKVDAPSGTALLLGQAAAEGRNVML
str.JK67       LVHIIGTTGFSKTEEEKIADFAKDTTIVKSGNMSLGVNLLASLVKKAAKALEVDDFDIEIYEMHHSGKVDAPSGTALLLGQAAAEGRNVML
str.JK12       LVHIIGTTGFSKTEEEKIADFAKDTTIVKSGNMSLGVNLLASLVKKAAKALEVDDFDIEIYEMHHSGKVDAPSGTALLLGQAAAEGRNVML
str.G1712      LVHIIGTTGFSKTEEEKIADFAKDTTIVKSGNMSLGVNLLASLVKKAAKALEVDDFDIEIYEMHHSGKVDAPSGTALLLGQAAAEGRNVML
str.G1732      LVHIIGTTGFSKTEEEKIADFAKDTTIVKSGNMSLGVNLLASLVKKAAKALEVDDFDIEIYEMHHSGKVDAPSGTALLLGQAAAEGRNVML
str.CO20_0297  LVHIIGTTGFSKTEEEKIADFAKDTTIVKSGNMSLGVNLLASLVKKAAKALEVDDFDIEIYEMHHSGKVDAPSGTALLLGQAAAEGRNVML
str.CO21_0024  LVHIIGTTGFSKTEEEKIADFAKDTTIVKSGNMSLGVNLLASLVKKAAKALEVDDFDIEIYEMHHSGKVDAPSGTALLLGQAAAEGRNVML
str.CO20_0257  LVHIIGTTGFSKTEEEKIADFAKDTTIVKSGNMSLGVNLLASLVKKAAKALEVDDFDIEIYEMHHSGKVDAPSGTALLLGQAAAEGRNVML
str.CO20_0321  LVHIIGTTGFSKTEEEKIADFAKDTTIVKSGNMSLGVNLLASLVKKAAKALEVDDFDIEIYEMHHSGKVDAPSGTALLLGQAAAEGRNVML
str.CO20_0256  LVHIIGTTGFSKTEEEKIADFAKDTTIVKSGNMSLGVNLLASLVKKAAKALEVDDFDIEIYEMHHSGKVDAPSGTALLLGQAAAEGRNVML
str.JK31       LVHIIGTTGFSKTEEEKIADFAKDTTIVKSGNMSLGVNLLASLVKKAAKALEVDDFDIEIYEMHHSGKVDAPSGTALLLGQAAAEGRNVML
str.NCTC12899  LVHIIGTTGFSKTEEEKIADFAKDTTIVKSGNMSLGVNLLASLVKKAAKALEVDDFDIEIYEMHHSGKVDAPSGTALLLGQAAAEGRNVML
str.JK63       LVHIIGTTGFSKTEEEKIADFAKDTTIVKSGNMSLGVNLLASLVKKAAKALEVDDFDIEIYEMHHSGKVDAPSGTALLLGQAAAEGRNVML
str.JK68       LVHIIGTTGFSKTEEEKIADFAKDTTIVKSGNMSLGVNLLASLVKKAAKALEVDDFDIEIYEMHHSGKVDAPSGTALLLGQAAAEGRNVML
str.JK39       LVHIIGTTGFSKTEEEKIADFAKDTTIVKSGNMSLGVNLLASLVKKAAKALEVDDFDIEIYEMHHSGKVDAPSGTALLLGQAAAEGRNVML
str.JK19       LVHIIGTTGFSKTEEEKIADFAKDTTIVKSGNMSLGVNLLASLVKKAAKALEVDDFDIEIYEMHHSGKVDAPSGTALLLGQAAAEGRNVML
str.JK7        LVHIIGTTGFSKTEEEKIADFAKDTTIVKSGNMSLGVNLLASLVKKAAKALEVDDFDIEIYEMHHSGKVDAPSGTALLLGQAAAEGRNVML
str.BQ2-D70    LVHIIGTTGFSKTEEEKIADFAKDTTIVKSGNMSLGVNLLASLVKKAAKALEVDDFDIEIYEMHHSGKVDAPSGTALLLGQAAAEGRNVML
str.JK56       LVHIIGTTGFSKTEEEKIADFAKDTTIVKSGNMSLGVNLLASLVKKAAKALEVDDFDIEIYEMHHSGKVDAPSGTALLLGQAAAEGRNVML
str.RM-11      LVHIIGTTGFSKIEEEKIADFAKDTTIVKSGNMSLGVNLLASLVKKAAKALEVDDFDIEIYEMHHSGKVDAPSGTALLLGQAAAEGRNVML
str.MF1-1      LVHIIGTTGFSKIEEEKIADFAKDTTIVKSGNMSLGVNLLASLVKKAAKALEVDDFDIEIYEMHHSGKVDAPSGTALLLGQAAAEGRNVML

                    190       200       210       220       230       240       250       260       270
str.Toulouse   KNVSVNARNGYTGEREKGTIGFACSRGGTVVGDHSITFAGPNERIVLSHVAQDRSIFANGALKAALWAKNHENGLYSMLDVLGLKDEF
str.JK73rel    KNVSVNARNGYTGEREKGTIGFACSRGGTVVGDHSITFAGPNERIVLSHVAQDRSIFANGALKAALWAKNHENGLYSMLDVLGLKDEF
str.JK73       KNVSVNARNGYTGEREKGTIGFACSRGGTVVGDHSITFAGPNERIVLSHVAQDRSIFANGALKAALWAKNHENGLYSMLDVLGLKDEF
str.CCUG45777  KNVSVNARNGYTGEREKGTIGFACSRGGTVVGDHSITFAGPNERIVLSHVAQDRSIFANGALKAALWAKNHENGLYSMLDVLGLKDEF
str.JK67       KNVSVNARNGYTGEREKGTIGFACSRGGTVVGDHSITFAGPNERIVLSHVAQDRSIFANGALKAALWAKNHENGLYSMLDVLGLKDEF
str.JK12       KNVSVNARNGYTGEREKGTIGFACSRGGTVVGDHSITFAGPNERIVLSHVAQDRSIFANGALKAALWAKNHENGLYSMLDVLGLKDEF
str.G1712      KNVSVNARNGYTGEREKGTIGFACSRGGTVVGDHSITFAGPNERIVLSHVAQDRSIFANGALKAALWAKNHENGLYSMLDVLGLKDEF
str.G1732      KNVSVNARNGYTGEREKGTIGFACSRGGTVVGDHSITFAGPNERIVLSHVAQDRSIFANGALKAALWAKNHENGLYSMLDVLGLKDEF
str.CO20_0297  KNVSVNARNGYTGEREKGTIGFACSRGGTVVGDHSITFAGPNERIVLSHVAQDRSIFANGALKAALWAKNHENGLYSMLDVLGLKDEF
str.CO21_0024  KNVSVNARNGYTGEREKGTIGFACSRGGTVVGDHSITFAGPNERIVLSHVAQDRSIFANGALKAALWAKNHENGLYSMLDVLGLKDEF
str.CO20_0257  KNVSVNARNGYTGEREKGTIGFACSRGGTVVGDHSITFAGPNERIVLSHVAQDRSIFANGALKAALWAKNHENGLYSMLDVLGLKDEF
str.CO20_0321  KNVSVNARNGYTGEREKGTIGFACSRGGTVVGDHSITFAGPNERIVLSHVAQDRSIFANGALKAALWAKNHENGLYSMLDVLGLKDEF
str.CO20_0256  KNVSVNARNGYTGEREKGTIGFACSRGGTVVGDHSITFAGPNERIVLSHVAQDRSIFANGALKAALWAKNHENGLYSMLDVLGLKDEF
str.JK31       KNVSVNARNGYTGEREKGTIGFACSRGGTVVGDHSITFAGPNERIVLSHVAQDRSIFANGALKAALWAKNHENGLYSMLDVLGLKDEF
str.NCTC12899  KNVSVNARNGYTGEREKGTIGFACSRGGTVVGDHSITFAGPNERIVLSHVAQDRSIFANGALKAALWAKNHENGLYSMLDVLGLKDEF
str.JK63       KNVSVNARNGYTGEREKGTIGFACSRGGTVVGDHSITFAGPNERIVLSHVAQDRSIFANGALKAALWAKNHENGLYSMLDVLGLKDEF
str.JK68       KNVSVNARNGYTGEREKGTIGFACSRGGTVVGDHSITFAGPNERIVLSHVAQDRSIFANGALKAALWAKNHENGLYSMLDVLGLKDEF
str.JK39       KNVSVNARNGYTGEREKGTIGFACSRGGTVVGDHSITFAGPNERIVLSHVAQDRSIFANGALKAALWAKNHENGLYSMLDVLGLKDEF
str.JK19       KNVSVNARNGYTGEREKGTIGFACSRGGTVVGDHSITFAGPNERIVLSHVAQDRSIFANGALKAALWAKNHENGLYSMLDVLGLKDEF
str.JK7        KNVSVNARNGYTGEREKGTIGFACSRGGTVVGDHSITFAGPNERIVLSHVAQDRSIFANGALKAALWAKNHENGLYSMLDVLGLKDEF
str.BQ2-D70    KNVSVNARNGYTGEREKGTIGFACSRGGTVVGDHSITFAGPNERIVLSHVAQDRSIFANGALKAALWAKNHENGLYSMLDVLGLKDEF
str.JK56       KNVSVNARNGYTGEREKGTIGFACSRGGTVVGDHSITFAGPNERIVLSHVAQDRSIFANGALKAALWAKNHENGLYSMLDVLGLKDEF
str.RM-11      KNVSVNARNGYTGEREKGTIGFSCSRGGTVVGDHSVTFAGPNERIVLSHVAQDRSIFANGALKAALWAKNHENGLYSMLDVLGLKDEF
str.MF1-1      KNVSVNARNGYTGEREKGTIGFSCSRGGTVVGDHSVTFAGPNERIVLSHVAQDRSIFANGALKAALWAKNHENGLYSMLDVLGLKDEF
```

>WP_011179695.1 alanine racemase [Bartonella quintana str. Toulouse]

```
              1        10        20        30        40        50        60        70        80        90
str.Toulouse  MNKSMNNKANPLLPYTAVATIDVSAIVANYKTLAQRVAPAECSAVVKANAYGLGADKIAPENWQKLCQKKDKKFPAIIQIDTNMNRLGLDK
str.JK73      MNKSMNNKANPLLPYTAVATIDVSAIVANYKTLAQRVAPAECSAVVKANAYGLGADKIAPENWQKLCQKKDKKFPAIIQIDTNMNRLGLDK
str.CCUG45777 MNKSMNNKANPLLPYTAVATIDVSAIVANYKTLAQRVAPAECSAVVKANAYGLGADKIAPENWQKLCQKKDKKFPAIIQIDTNMNRLGLDK
str.JK73rel   MNKSMNNKANPLLPYTAVATIDVSAIVANYKTLAQRVAPAECSAVVKANAYGLGADKIAPENWQKLCQKKDKKFPAIIQIDTNMNRLGLDK
str.JK19      MNKSMNNKANPLLPYTAVATIDVSAIVANYKTLAQRVAPAECSAVVKANAYGLGADKIAPENWQKLCQKKDKKFPAIIQIDTNMNRLGLDK
str.JK63      MNKSMNNKANPLLPYTAVATIDVSAIVANYKTLAQRVAPAECSAVVKANAYGLGADKIAPENWQKLCQKKDKKFPAIIQIDTNMNRLGLDK
str.JK56      MNKSMNNKANPLLPYTAVATIDVSAIVANYKTLAQRVAPAECSAVVKANAYGLGADKIAPENWQKLCQKKDKKFPAIIQIDTNMNRLGLDK
str.JK67      MNKSMNNKANPLLPYTAVATIDVSAIVANYKTLAQRVAPAECSAVVKANAYGLGADKIAPENWQKLCQKKDKKFPAIIQIDTNMNRLGLDK
str.JK68      MNKSMNNKANPLLPYTAVATIDVSAIVANYKTLAQRVAPAECSAVVKANAYGLGADKIAPENWQKLCQKKDKKFPAIIQIDTNMNRLGLDK
str.JK39      MNKSMNNKANPLLPYTAVATIDVSAIVANYKTLAQRVAPAECSAVVKANAYGLGADKIAPENWQKLCQKKDKKFPAIIQIDTNMNRLGLDK
str.JK31      MNKSMNNKANPLLPYTAVATIDVSAIVANYKTLAQRVAPAECSAVVKANAYGLGADKIAPENWQKLCQKKDKKFPAIIQIDTNMNRLGLDK
str.G1713     MNKSMNNKANPLLPYTAVATIDVSAIVANYKTLAQRVAPAECSAVVKANAYGLGADKIAPENWQKLCQKKDKKFPAIIQIDTNMNRLGLDK
str.JK7       MNKSMNNKANPLLPYTAVATIDVSAIVANYKTLAQRVAPAECSAVVKANAYGLGADKIAPENWQKLCQKKDKKFPAIIQIDTNMNRLGLDK
str.JK12      MNKSMNNKANPLLPYTAVATIDVSAIVANYKTLAQRVAPAECSAVVKANAYGLGADKIAPENWQKLCQKKDKKFPAIIQIDTNMNRLGLDK
str.CO21_0024 MNKSMNNKANPLLPYTAVATIDVSAIVANYKTLAQRVAPAECSAVVKANAYGLGADKIAPENWQKLCQKKDKKFPAIIQIDTNMNRLGLDK
str.G1712     MNKSMNNKANPLLPYTAVATIDVSAIVANYKTLAQRVAPAECSAVVKANAYGLGADKIAPENWQKLCQKKDKKFPAIIQIDTNMNRLGLDK
str.BQ2-D7    MNKSMNNKANPLLPYTAVATIDVSAIVANYKTLAQRVAPAECSAVVKANAYGLGADKIAPENWQKLCQKKDKKFPAIIQIDTNMNRLGLDK
str.CO20_0321 MNKSMNNKANPLLPYTAVATIDVSAIVANYKTLAQRVAPAECSAVVKANAYGLGADKIAPENWQKLCQKKDKKFPAIIQIDTNMNRLGLDK
str.CO20_0297 MNKSMNNKANPLLPYTAVATIDVSAIVANYKTLAQRVAPAECSAVVKANAYGLGADKIAPENWQKLCQKKDKKFPAIIQIDTNMNRLGLDK
str.CO20_0256 MNKSMNNKANPLLPYTAVATIDVSAIVANYKTLAQRVAPAECSAVVKANAYGLGADKIAPENWQKLCQKKDKKFPAIIQIDTNMNRLGLDK
str.CO20_0257 MNKSMNNKANPLLPYTAVATIDVSAIVANYKTLAQRVAPAECSAVVKANAYGLGADKIAPENWQKLCQKKDKKFPAIIQIDTNMNRLGLDK
str.NCTC12899 MNKSMNNKANPLLPYTAVATIDVSAIVANYKTLAQRVAPAECSAVVKANAYGLGADKIAPENWQKLCQKKDKKFPAIIQIDTNMNRLGLDK
str.MF1-1     MNKSMNNKANPLLPYTAVATIDVSAIVANYKTLAQHVAPAECSAVVKANAYGLGADKIAPENWQKLCQKKDKKFPAIIQIDTNMNRLGLDK
str.RM-11     MNKSMNNKANPLLPYTAVATIDVSAIVANYKTLAQHVAPAECSAVVKANAYGLGADKIVPENWQKLCQKKDKKFPAIIQIDTNMNRLGLDK
```

```
                       100       110       120       130       140       150       160       170       180
str.Toulouse  KELQQLIKKPTIFEKAEIKYILSHLANGDDATHSSNYTQLAAFKTVLTQLPTCRASFANSGGIFLGPDFYFDLVRPGIALYGIDPQGKHPT
str.JK73      KELQQLIKKPTIFEKAEIKYILSHLANGDDATHSSNYTQLAAFKTVLTQLPTCRASFANSGGIFLGPDFYFDLVRPGIALYGIDPQGKHPT
str.CCUG45777 KELQQLIKKPTIFEKAEIKYILSHLANGDDATHSSNYTQLAAFKTVLTQLPTCRASFANSGGIFLGPDFYFDLVRPGIALYGIDPQGKHPT
str.JK73rel   KELQQLIKKPTIFEKAEIKYILSHLANGDDATHSSNYTQLAAFKTVLTQLPTCRASFANSGGIFLGPDFYFDLVRPGIALYGIDPQGKHPT
str.JK19      KELQQLIKKPTIFEKAEIKYILSHLANGDDATHSSNYTQLAAFKTVLTQLPTCRASFANSGGIFLGPDFYFDLVRPGIALYGIDPQGKHPT
str.JK63      KELQQLIKKPTIFEKAEIKYILSHLANGDDATHSSNYTQLAAFKTVLTQLPTCRASFANSGGIFLGPDFYFDLVRPGIALYGIDPQGKHPT
str.JK56      KELQQLIKKPTIFEKAEIKYILSHLANGDDATHSSNYTQLAAFKTVLTQLPTCRASFANSGGIFLGPDFYFDLVRPGIALYGIDPQGKHPT
str.JK67      KELQQLIKKPTIFEKAEIKYILSHLANGDDATHSSNYTQLAAFKTVLTQLPTCRASFANSGGIFLGPDFYFDLVRPGIALYGIDPQGKHPT
str.JK68      KELQQLIKKPTIFEKAEIKYILSHLANGDDATHSSNYTQLAAFKTVLTQLPTCRASFANSGGIFLGPDFYFDLVRPGIALYGIDPQGKHPT
str.JK39      KELQQLIKKPTIFEKAEIKYILSHLANGDDATHSSNYTQLAAFKTVLTQLPTCRASFANSGGIFLGPDFYFDLVRPGIALYGIDPQGKHPT
str.JK31      KELQQLIKKPTIFEKAEIKYILSHLANGDDATHSSNYTQLAAFKTVLTQLPTCRASFANSGGIFLGPDFYFDLVRPGIALYGIDPQGKHPT
str.G1713     KELQQLIKKPTIFEKAEIKYILSHLANGDDATHSSNYTQLAAFKTVLTQLPTCRASFANSGGIFLGPDFYFDLVRPGIALYGIDPQGKHPT
str.JK7       KELQQLIKKPTIFEKAEIKYILSHLANGDDATHSSNYTQLAAFKTVLTQLPTCRASFANSGGIFLGPDFYFDLVRPGIALYGIDPQGKHPT
str.JK12      KELQQLIKKPTIFEKAEIKYILSHLANGDDATHSSNYTQLAAFKTVLTQLPTCRASFANSGGIFLGPDFYFDLVRPGIALYGIDPQGKHPT
str.CO21_0024 KELQQLIKKPTIFEKAEIKYILSHLANGDDATHSSNYTQLAAFKTVLTQLPTCRASFANSGGIFLGPDFYFDLVRPGIALYGIDPQGKHPT
str.G1712     KELQQLIKKPTIFEKAEIKYILSHLANGDDATHSSNYTQLAAFKTVLTQLPTCRASFANSGGIFLGPDFYFDLVRPGIALYGIDPQGKHPT
str.BQ2-D7    KELQQLIKKPTIFEKAEIKYILSHLANGDDATHSSNYTQLAAFKTVLTQLPTCRASFANSGGIFLGPDFYFDLVRPGIALYGIDPQGKHPT
str.CO20_0321 KELQQLIKKPTIFEKAEIKYILSHLANGDDATHSSNYTQLAAFKTVLTQLPTCRASFANSGGIFLGPDFYFDLVRPGIALYGIDPQGKHPT
str.CO20_0297 KELQQLIKKPTIFEKAEIKYILSHLANGDDATHSSNYTQLAAFKTVLTQLPTCRASFANSGGIFLGPDFYFDLVRPGIALYGIDPQGKHPT
str.CO20_0256 KELQQLIKKPTIFEKAEIKYILSHLANGDDATHSSNYTQLAAFKTVLTQLPTCRASFANSGGIFLGPDFYFDLVRPGIALYGIDPQGKHPT
str.CO20_0257 KELQQLIKKPTIFEKAEIKYILSHLANGDDATHSSNYTQLAAFKTVLTQLPTCRASFANSGGIFLGPDFYFDLVRPGIALYGIDPQGKHPT
str.NCTC12899 KELQQLIKKPTIFEKAEIKYILSHLANGDDATHSSNYTQLAAFKTVLTQLPTCRASFANSGGIFLGPDFYFDLVRPGIALYGIDPQGKHPT
str.MF1-1     KELQQLIKKPTVFEKAEIKYILSHLANGDDATHSSNYTQLAAFKTVLTQLPTCRASFANSGGIFLGPDFYFDLVRPGIALYGIDPQGKHPT
str.RM-11     KELQQLIKKPTVFEKAEIKYILSHLANGDDATHSSNYTQLAAFKTVLTQLPTCRASFANSGGIFLGPDFYFDLVRPGIALYGIDPQGKHPT
```

```
                     190       200       210       220       230       240       250       260       270
str.Toulouse  LFKPVLKLEAQVIQSRSIEAGIPVGYEESFITRRPSTLITISIGYADGWLRILSNKGTVYFNGHKLPIVGRISMDSMTVDATNLDKKPQSG
str.JK73      LFKPVLKLEAQVIQSRSIEAGIPVGYEESFITRRPSTLITISIGYADGWLRILSNKGTVYFNGHKLPIVGRISMDSMTVDATNLDKKPQSG
str.CCUG45777 LFKPVLKLEAQVIQSRSIEAGIPVGYEESFITRRPSTLITISIGYADGWLRILSNKGTVYFNGHKLPIVGRISMDSMTVDATNLDKKPQSG
str.JK73rel   LFKPVLKLEAQVIQSRSIEAGIPVGYEESFITRRPSTLITISIGYADGWLRILSNKGTVYFNGHKLPIVGRISMDSMTVDATNLDKKPQSG
str.JK19      LFKPVLKLEAQVIQSRSIEAGIPVGYEESFITRRPSTLITISIGYADGWLRILSNKGTVYFNGHKLPIVGRISMDSMTVDATNLDKKPQSG
str.JK63      LFKPVLKLEAQVIQSRSIEAGIPVGYEESFITRRPSTLITISIGYADGWLRILSNKGTVYFNGHKLPIVGRISMDSMTVDATNLDKKPQSG
str.JK56      LFKPVLKLEAQVIQSRSIEAGIPVGYEESFITRRPSTLITISIGYADGWLRILSNKGTVYFNGHKLPIVGRISMDSMTVDATNLDKKPQSG
str.JK67      LFKPVLKLEAQVIQSRSIEAGIPVGYEESFITRRPSTLITISIGYADGWLRILSNKGTVYFNGHKLPIVGRISMDSMTVDATNLDKKPQSG
str.JK68      LFKPVLKLEAQVIQSRSIEAGIPVGYEESFITRRPSTLITISIGYADGWLRILSNKGTVYFNGHKLPIVGRISMDSMTVDATNLDKKPQSG
str.JK39      LFKPVLKLEAQVIQSRSIEAGIPVGYEESFITRRPSTLITISIGYADGWLRILSNKGTVYFNGHKLPIVGRISMDSMTVDATNLDKKPQSG
str.JK31      LFKPVLKLEAQVIQSRSIEAGIPVGYEESFITRRPSTLITISIGYADGWLRILSNKGTVYFNGHKLPIVGRISMDSMTVDATNLDKKPQSG
str.G1713     LFKPVLKLEAQVIQSRSIEAGIPVGYEESFITRRPSTLITISIGYADGWLRILSNKGTVYFNGHKLPIVGRISMDSMTVDATNLDKKPQSG
str.JK7       LFKPVLKLEAQVIQSRSIEAGIPVGYEESFITRRPSTLITISIGYADGWLRILSNKGTVYFNGHKLPIVGRISMDSMTVDATNLDKKPQSG
str.JK12      LFKPVLKLEAQVIQSRSIEAGIPVGYEESFITRRPSTLITISIGYADGWLRILSNKGTVYFNGHKLPIVGRISMDSMTVDATNLDKKPQSG
str.CO21_0024 LFKPVLKLEAQVIQSRSIEAGIPVGYEESFITRRPSTLITISIGYADGWLRILSNKGTVYFNGHKLPIVGRISMDSMTVDATNLDKKPQSG
str.G1712     LFKPVLKLEAQVIQSRSIEAGIPVGYEESFITRRPSTLITISIGYADGWLRILSNKGTVYFNGHKLPIVGRISMDSMTVDATNLDKKPQSG
str.BQ2-D7    LFKPVLKLEAQVIQSRSIEAGIPVGYEESFITRRPSTLITISIGYADGWLRILSNKGTVYFNGHKLPIVGRISMDSMTVDATNLDKKPQSG
str.CO20_0321 LFKPVLKLEAQVIQSRSIEAGIPVGYEESFITRRPSTLITISIGYADGWLRILSNKGTVYFNGHKLPIVGRISMDSMTVDATNLDKKPQSG
str.CO20_0297 LFKPVLKLEAQVIQSRSIEAGIPVGYEESFITRRPSTLITISIGYADGWLRILSNKGTVYFNGHKLPIVGRISMDSMTVDATNLDKKPQSG
str.CO20_0256 LFKPVLKLEAQVIQSRSIEAGIPVGYEESFITRRPSTLITISIGYADGWLRILSNKGTVYFNGHKLPIVGRISMDSMTVDATNLDKKPQSG
str.CO20_0257 LFKPVLKLEAQVIQSRSIEAGIPVGYEESFITRRPSTLITISIGYADGWLRILSNKGTVYFNGHKLPIVGRISMDSMTVDATNLDKKPQSG
str.NCTC12899 LFKPVLKLEAQVIQSRSIEAGIPVGYEESFITRRPSTLITISIGYADGWLRILSNKGTVYFNGHKLPIVGRISMDSMTVDATNLDKKPQSG
str.MF1-1     LFKPVLKLEAQVIQSRSIEAGIPVGYEESFITRRPSTLITISIGYADGWLRILSNKGTVYFNGHKLPIVGRISMDSMTVDATNLDKKPQSG
str.RM-11     LFKPVLKLEAQVIQSRSIEAGIPVGYEESFITRRPSTLITISIGYADGWLRILSNKGTVYFNGQKLPIVGRISMDSMTVDATNLDKKPQSG
```

```
                  280       290       300       310
str.Toulouse  DWVELIGPHQTLKKVSIDANTIPHEILTSLGSRYQRIYI
str.JK73      DWVELIGPHQTLKKVSIDANTIPHEILTSLGSRYQRIYI
str.CCUG45777 DWVELIGPHQTLKKVSIDANTIPHEILTSLGSRYQRIYI
str.JK73rel   DWVELIGPHQTLKKVSIDANTIPHEILTSLGSRYQRIYI
str.JK19      DWVELIGPHQTLKKVSIDANTIPHEILTSLGSRYQRIYI
str.JK63      DWVELIGPHQTLKKVSIDANTIPHEILTSLGSRYQRIYI
str.JK56      DWVELIGPHQTLKKVSIDANTIPHEILTSLGSRYQRIYI
str.JK67      DWVELIGPHQTLKKVSIDANTIPHEILTSLGSRYQRIYI
str.JK68      DWVELIGPHQTLKKVSIDANTIPHEILTSLGSRYQRIYI
str.JK39      DWVELIGPHQTLKKVSIDANTIPHEILTSLGSRYQRIYI
str.JK31      DWVELIGPHQTLKKVSIDANTIPHEILTSLGSRYQRIYI
str.G1713     DWVELIGPHQTLKKVSIDANTIPHEILTSLGSRYQRIYI
str.JK7       DWVELIGPHQTLKKVSIDANTIPHEILTSLGSRYQRIYI
str.JK12      DWVELIGPHQTLKKVSIDANTIPHEILTSLGSRYQRIYI
str.CO21_0024 DWVELIGPHQTLKKVSIDANTIPHEILTSLGSRYQRIYI
str.G1712     DWVELIGPHQTLKKVSIDANTIPHEILTSLGSRYQRIYI
str.BQ2-D7    DWVELIGPHQTLKKVSIDANTIPHEILTSLGSRYQRIYI
str.CO20_0321 DWVELIGPHQTLKKVSIDANTIPHEILTSLGSRYQRIYI
str.CO20_0297 DWVELIGPHQTLKKVSIDANTIPHEILTSLGSRYQRIYI
str.CO20_0256 DWVELIGPHQTLKKVSIDANTIPHEILTSLGSRYQRIYI
str.CO20_0257 DWVELIGPHQTLKKVSIDANTIPHEILTSLGSRYQRIYI
str.NCTC12899 DWVELIGPHQTLKKVSIDANTIPHEILTSLGSRYQRIYI
str.MF1-1     DWVELIGPHQTLKKVSIDANTIPHEILTSLGSRYQRIYI
str.RM-11     DWVELIGPHQTLKKVSIDANTIPHEILTSLGSRYQRIYI
```

>WP_011179784.1 UDP-N-acetylglucosamine 1-carboxyvinyltransferase [Bartonella quintana str. Toulouse]

```
               1        10        20        30        40        50        60        70        80        90
str.Toulouse   MDSIQIVGGKKLKGTIPISGAKNAALPLMIAALLTEETVTLDNIPHLADVELLIRILNNHGVGYAVDGQKSHHDCIDSRTIHFTAQKITTT
str.JK12       MDSIQIVGGKKLKGTIPISGAKNAALPLMIAALLTEETVTLDNIPHLADVELLIRILNNHGVGYAVDGQKSHHDCIDSRTIHFTAQKITTT
str.CCUG45777  MDSIQIVGGKKLKGTIPISGAKNAALPLMIAALLTEETVTLDNIPHLADVELLIRILNNHGVGYAVDGQKSHHDCIDSRTIHFTAQKITTT
str.JK56       MDSIQIVGGKKLKGTIPISGAKNAALPLMIAALLTEETVTLDNIPHLADVELLIRILNNHGVGYAVDGQKSHHDCIDSRTIHFTAQKITTT
str.JK67       MDSIQIVGGKKLKGTIPISGAKNAALPLMIAALLTEETVTLDNIPHLADVELLIRILNNHGVGYAVDGQKSHHDCIDSRTIHFTAQKITTT
str.JK19       MDSIQIVGGKKLKGTIPISGAKNAALPLMIAALLTEETVTLDNIPHLADVELLIRILNNHGVGYAVDGQKSHHDCIDSRTIHFTAQKITTT
str.JK63       MDSIQIVGGKKLKGTIPISGAKNAALPLMIAALLTEETVTLDNIPHLADVELLIRILNNHGVGYAVDGQKSHHDCIDSRTIHFTAQKITTT
str.JK39       MDSIQIVGGKKLKGTIPISGAKNAALPLMIAALLTEETVTLDNIPHLADVELLIRILNNHGVGYAVDGQKSHHDCIDSRTIHFTAQKITTT
str.JK68       MDSIQIVGGKKLKGTIPISGAKNAALPLMIAALLTEETVTLDNIPHLADVELLIRILNNHGVGYAVDGQKSHHDCIDSRTIHFTAQKITTT
str.JK31       MDSIQIVGGKKLKGTIPISGAKNAALPLMIAALLTEETVTLDNIPHLADVELLIRILNNHGVGYAVDGQKSHHDCIDSRTIHFTAQKITTT
str.G1713      MDSIQIVGGKKLKGTIPISGAKNAALPLMIAALLTEETVTLDNIPHLADVELLIRILNNHGVGYAVDGQKSHHDCIDSRTIHFTAQKITTT
str.G1712      MDSIQIVGGKKLKGTIPISGAKNAALPLMIAALLTEETVTLDNIPHLADVELLIRILNNHGVGYAVDGQKSHHDCIDSRTIHFTAQKITTT
str.JK73       MDSIQIVGGKKLKGTIPISGAKNAALPLMIAALLTEETVTLDNIPHLADVELLIRILNNHGVGYAVDGQKSHHDCIDSRTIHFTAQKITTT
str.JK7        MDSIQIVGGKKLKGTIPISGAKNAALPLMIAALLTEETVTLDNIPHLADVELLIRILNNHGVGYAVDGQKSHHDCIDSRTIHFTAQKITTT
str.JK73rel    MDSIQIVGGKKLKGTIPISGAKNAALPLMIAALLTEETVTLDNIPHLADVELLIRILNNHGVGYAVDGQKSHHDCIDSRTIHFTAQKITTT
str.CO20_0297  MDSIQIVGGKKLKGTIPISGAKNAALPLMIAALLTEETVTLDNIPHLADVELLIRILNNHGVGYAVDGQKSHHDCIDSRTIHFTAQKITTT
str.CO21_0024  MDSIQIVGGKKLKGTIPISGAKNAALPLMIAALLTEETVTLDNIPHLADVELLIRILNNHGVGYAVDGQKSHHDCIDSRTIHFTAQKITTT
str.CO20_0321  MDSIQIVGGKKLKGTIPISGAKNAALPLMIAALLTEETVTLDNIPHLADVELLIRILNNHGVGYAVDGQKSHHDCIDSRTIHFTAQKITTT
str.BQ2-D70    MDSIQIVGGKKLKGTIPISGAKNAALPLMIAALLTEETVTLDNIPHLADVELLIRILNNHGVGYAVDGQKSHHDCIDSRTIHFTAQKITTT
str.CO20_0256  MDSIQIVGGKKLKGTIPISGAKNAALPLMIAALLTEETVTLDNIPHLADVELLIRILNNHGVGYAVDGQKSHHDCIDSRTIHFTAQKITTT
str.CO20_0257  MDSIQIVGGKKLKGTIPISGAKNAALPLMIAALLTEETVTLDNIPHLADVELLIRILNNHGVGYAVDGQKSHHDCIDSRTIHFTAQKITTT
str.NCTC12899  MDSIQIVGGKKLKGTIPISGAKNAALPLMIAALLTEETVTLDNIPHLADVELLIRILNNHGVGYAVDGQKSHHDCIDSRTIHFTAQKITTT
str.MF1-1      MDSIQIVGGKKLKGTIPISGAKNAALPLMIAALLTEETVTLDNIPHLADVELLIRILNNHGVGYAVDGQKSHHDCTDSRTIHFTAQKITTT
str.RM-11      MDSIQIVGGKKLKGTIPISGAKNAALPLMIAALLTEETVTLDNIPHLADVELLIRILNNHGVGYAVDGQKPHHDCTDSRTIHFTAQKITTT
```

```
                       100       110       120       130       140       150       160       170       180
str.Toulouse   YAPYELVRKMRASFWVIGPLLARCHEAYVSLPGGCAIGTRPVNFILEGLKTLGARIAIENGYVHAKAPKGLKGAHYHFPKVTVGGTHVMLM
str.JK12       YAPYELVRKMRASFWVIGPLLARCHEAYVSLPGGCAIGTRPVNFILEGLKTLGARIAIENGYVHAKAPKGLKGAHYHFPKVTVGGTHVMLM
str.CCUG45777  YAPYELVRKMRASFWVIGPLLARCHEAYVSLPGGCAIGTRPVNFILEGLKTLGARIAIENGYVHAKAPKGLKGAHYHFPKVTVGGTHVMLM
str.JK56       YAPYELVRKMRASFWVIGPLLARCHEAYVSLPGGCAIGTRPVNFILEGLKTLGARIAIENGYVHAKAPKGLKGAHYHFPKVTVGGTHVMLM
str.JK67       YAPYELVRKMRASFWVIGPLLARCHEAYVSLPGGCAIGTRPVNFILEGLKTLGARIAIENGYVHAKAPKGLKGAHYHFPKVTVGGTHVMLM
str.JK19       YAPYELVRKMRASFWVIGPLLARCHEAYVSLPGGCAIGTRPVNFILEGLKTLGARIAIENGYVHAKAPKGLKGAHYHFPKVTVGGTHVMLM
str.JK63       YAPYELVRKMRASFWVIGPLLARCHEAYVSLPGGCAIGTRPVNFILEGLKTLGARIAIENGYVHAKAPKGLKGAHYHFPKVTVGGTHVMLM
str.JK39       YAPYELVRKMRASFWVIGPLLARCHEAYVSLPGGCAIGTRPVNFILEGLKTLGARIAIENGYVHAKAPKGLKGAHYHFPKVTVGGTHVMLM
str.JK68       YAPYELVRKMRASFWVIGPLLARCHEAYVSLPGGCAIGTRPVNFILEGLKTLGARIAIENGYVHAKAPKGLKGAHYHFPKVTVGGTHVMLM
str.JK31       YAPYELVRKMRASFWVIGPLLARCHEAYVSLPGGCAIGTRPVNFILEGLKTLGARIAIENGYVHAKAPKGLKGAHYHFPKVTVGGTHVMLM
str.G1713      YAPYELVRKMRASFWVIGPLLARCHEAYVSLPGGCAIGTRPVNFILEGLKTLGARIAIENGYVHAKAPKGLKGAHYHFPKVTVGGTHVMLM
str.G1712      YAPYELVRKMRASFWVIGPLLARCHEAYVSLPGGCAIGTRPVNFILEGLKTLGARIAIENGYVHAKAPKGLKGAHYHFPKVTVGGTHVMLM
str.JK73       YAPYELVRKMRASFWVIGPLLARCHEAYVSLPGGCAIGTRPVNFILEGLKTLGARIAIENGYVHAKAPKGLKGAHYHFPKVTVGGTHVMLM
str.JK7        YAPYELVRKMRASFWVIGPLLARCHEAYVSLPGGCAIGTRPVNFILEGLKTLGARIAIENGYVHAKAPKGLKGAHYHFPKVTVGGTHVMLM
str.JK73rel    YAPYELVRKMRASFWVIGPLLARCHEAYVSLPGGCAIGTRPVNFILEGLKTLGARIAIENGYVHAKAPKGLKGAHYHFPKVTVGGTHVMLM
str.CO20_0297  YAPYELVRKMRASFWVIGPLLARCHEAYVSLPGGCAIGTRPVNFILEGLKTLGARIAIENGYVHAKAPKGLKGAHYHFPKVTVGGTHVMLM
str.CO21_0024  YAPYELVRKMRASFWVIGPLLARCHEAYVSLPGGCAIGTRPVNFILEGLKTLGARIAIENGYVHAKAPKGLKGAHYHFPKVTVGGTHVMLM
str.CO20_0321  YAPYELVRKMRASFWVIGPLLARCHEAYVSLPGGCAIGTRPVNFILEGLKTLGARIAIENGYVHAKAPKGLKGAHYHFPKVTVGGTHVMLM
str.BQ2-D70    YAPYELVRKMRASFWVIGPLLARCHEAYVSLPGGCAIGTRPVNFILEGLKTLGARIAIENGYVHAKAPKGLKGAHYHFPKVTVGGTHVMLM
str.CO20_0256  YAPYELVRKMRASFWVIGPLLARCHEAYVSLPGGCAIGTRPVNFILEGLKTLGARIAIENGYVHAKAPKGLKGAHYHFPKVTVGGTHVMLM
str.CO20_0257  YAPYELVRKMRASFWVIGPLLARCHEAYVSLPGGCAIGTRPVNFILEGLKTLGARIAIENGYVHAKAPKGLKGAHYHFPKVTVGGTHVMLM
str.NCTC12899  YAPYELVRKMRASFWVIGPLLARCHEAYVSLPGGCAIGTRPVNFILEGLKTLGARIAIENGYVHAKAPKGLKGAHYHFPKVTVGGTHVMLM
str.MF1-1      YAPYELVRKMRASFWVIGPLLARCHEAYVSLPGGCAIGTRPVNFILEGLKTLGARIAIENGYVHAKAPKGLKGAHYHFPKVTVGGTHVMLM
str.RM-11      YAPYELVRKMRASFWVIGPLLARCHEAYVSLPGGCAIGTRPVNFILEGLKTLGARIAIENGYVHAKAPKGLKGAHYHFPKVTVGGTHVMLM
```

```
                      190       200       210       220       230       240       250       260       270
str.Toulouse   AAATANGTTLLENAACEPEVTNLIQALNAMGAKITGEGTSTLTIEGVKKLHGARISVIADRIEAGTYAMVVAMTGGNVLLKNANPHHLTQV
str.JK12       AAATANGTTLLENAACEPEVTNLIQALNAMGAKITGEGTSTLTIEGVKKLHGARISVIADRIEAGTYAMVVAMTGGNVLLKNANPHHLTQV
str.CCUG45777  AAATANGTTLLENAACEPEVTNLIQALNAMGAKITGEGTSTLTIEGVKKLHGARISVIADRIEAGTYAMVVAMTGGNVLLKNANPHHLTQV
str.JK56       AAATANGTTLLENAACEPEVTNLIQALNAMGAKITGEGTSTLTIEGVKKLHGARISVIADRIEAGTYAMVVAMTGGNVLLKNANPHHLTQV
str.JK67       AAATANGTTLLENAACEPEVTNLIQALNAMGAKITGEGTSTLTIEGVKKLHGARISVIADRIEAGTYAMVVAMTGGNVLLKNANPHHLTQV
str.JK19       AAATANGTTLLENAACEPEVTNLIQALNAMGAKITGEGTSTLTIEGVKKLHGARISVIADRIEAGTYAMVVAMTGGNVLLKNANPHHLTQV
str.JK63       AAATANGTTLLENAACEPEVTNLIQALNAMGAKITGEGTSTLTIEGVKKLHGARISVIADRIEAGTYAMVVAMTGGNVLLKNANPHHLTQV
str.JK39       AAATANGTTLLENAACEPEVTNLIQALNAMGAKITGEGTSTLTIEGVKKLHGARISVIADRIEAGTYAMVVAMTGGNVLLKNANPHHLTQV
str.JK68       AAATANGTTLLENAACEPEVTNLIQALNAMGAKITGEGTSTLTIEGVKKLHGARISVIADRIEAGTYAMVVAMTGGNVLLKNANPHHLTQV
str.JK31       AAATANGTTLLENAACEPEVTNLIQALNAMGAKITGEGTSTLTIEGVKKLHGARISVIADRIEAGTYAMVVAMTGGNVLLKNANPHHLTQV
str.G1713      AAATANGTTLLENAACEPEVTNLIQALNAMGAKITGEGTSTLTIEGVKKLHGARISVIADRIEAGTYAMVVAMTGGNVLLKNANPHHLTQV
str.G1712      AAATANGTTLLENAACEPEVTNLIQALNAMGAKITGEGTSTLTIEGVKKLHGARISVIADRIEAGTYAMVVAMTGGNVLLKNANPHHLTQV
str.JK73       AAATANGTTLLENAACEPEVTNLIQALNAMGAKITGEGTSTLTIEGVKKLHGARISVIADRIEAGTYAMVVAMTGGNVLLKNANPHHLTQV
str.JK7        AAATANGTTLLENAACEPEVTNLIQALNAMGAKITGEGTSTLTIEGVKKLHGARISVIADRIEAGTYAMVVAMTGGNVLLKNANPHHLTQV
str.JK73rel    AAATANGTTLLENAACEPEVTNLIQALNAMGAKITGEGTSTLTIEGVKKLHGARISVIADRIEAGTYAMVVAMTGGNVLLKNANPHHLTQV
str.CO20_0297  AAATANGTTLLENAACEPEVTNLIQALNAMGAKITGEGTSTLTIEGVKKLHGARISVIADRIEAGTYAMVVAMTGGNVLLKNANPHHLTQV
str.CO21_0024  AAATANGTTLLENAACEPEVTNLIQALNAMGAKITGEGTSTLTIEGVKKLHGARISVIADRIEAGTYAMVVAMTGGNVLLKNANPHHLTQV
str.CO20_0321  AAATANGTTLLENAACEPEVTNLIQALNAMGAKITGEGTSTLTIEGVKKLHGARISVIADRIEAGTYAMVVAMTGGNVLLKNANPHHLTQV
str.BQ2-D70    AAATANGTTLLENAACEPEVTNLIQALNAMGAKITGEGTSTLTIEGVKKLHGARISVIADRIEAGTYAMVVAMTGGNVLLKNANPHHLTQV
str.CO20_0256  AAATANGTTLLENAACEPEVTNLIQALNAMGAKITGEGTSTLTIEGVKKLHGARISVIADRIEAGTYAMVVAMTGGNVLLKNANPHHLTQV
str.CO20_0257  AAATANGTTLLENAACEPEVTNLIQALNAMGAKITGEGTSTLTIEGVKKLHGARISVIADRIEAGTYAMVVAMTGGNVLLKNANPHHLTQV
str.NCTC12899  AAATANGTTLLENAACEPEVTNLIQALNAMGAKITGEGTSTLTIEGVKKLHGARISVIADRIEAGTYAMVVAMTGGNVLLKNANPHHLTQV
str.MF1-1      AAATANGTTLLENAACEPEVTNLIQALNAMGAKITGEGTSTLTIEGVKKLHGARISVIADRIEAGTYAMVVAMTGGNVLLKNANPHHLTQV
str.RM-11      AAATANGTTLLENAACEPEVTNLIQALNAMGAKITGEGTSTLTIEGVKKLHGARISVIADRIEAGTYAMVVAMTGGNVLLKNANPHHLTQV
```

```
                     280       290       300       310       320       330       340       350       360
str.Toulouse   LEVLQKTGLDIEIKPQGLHLKRNPRQTKIMPVNIKTGPYPAFPTDLQAQFMALMTRAQGTACITETIFENRFMHVQELTRLGAQIKLDGQT
str.JK12       LEVLQKTGLDIEIKPQGLHLKRNPRQTKIMPVNIKTGPYPAFPTDLQAQFMALMTRAQGTACITETIFENRFMHVQELTRLGAQIKLDGQT
str.CCUG45777  LEVLQKTGLDIEIKPQGLHLKRNPRQTKIMPVNIKTGPYPAFPTDLQAQFMALMTRAQGTACITETIFENRFMHVQELTRLGAQIKLDGQT
str.JK56       LEVLQKTGLDIEIKPQGLHLKRNPRQTKIMPVNIKTGPYPAFPTDLQAQFMALMTRAQGTACITETIFENRFMHVQELTRLGAQIKLDGQT
str.JK67       LEVLQKTGLDIEIKPQGLHLKRNPRQTKIMPVNIKTGPYPAFPTDLQAQFMALMTRAQGTACITETIFENRFMHVQELTRLGAQIKLDGQT
str.JK19       LEVLQKTGLDIEIKPQGLHLKRNPRQTKIMPVNIKTGPYPAFPTDLQAQFMALMTRAQGTACITETIFENRFMHVQELTRLGAQIKLDGQT
str.JK63       LEVLQKTGLDIEIKPQGLHLKRNPRQTKIMPVNIKTGPYPAFPTDLQAQFMALMTRAQGTACITETIFENRFMHVQELTRLGAQIKLDGQT
str.JK39       LEVLQKTGLDIEIKPQGLHLKRNPRQTKIMPVNIKTGPYPAFPTDLQAQFMALMTRAQGTACITETIFENRFMHVQELTRLGAQIKLDGQT
str.JK68       LEVLQKTGLDIEIKPQGLHLKRNPRQTKIMPVNIKTGPYPAFPTDLQAQFMALMTRAQGTACITETIFENRFMHVQELTRLGAQIKLDGQT
str.JK31       LEVLQKTGLDIEIKPQGLHLKRNPRQTKIMPVNIKTGPYPAFPTDLQAQFMALMTRAQGTACITETIFENRFMHVQELTRLGAQIKLDGQT
str.G1713      LEVLQKTGLDIEIKPQGLHLKRNPRQTKIMPVNIKTGPYPAFPTDLQAQFMALMTRAQGTACITETIFENRFMHVQELTRLGAQIKLDGQT
str.G1712      LEVLQKTGLDIEIKPQGLHLKRNPRQTKIMPVNIKTGPYPAFPTDLQAQFMALMTRAQGTACITETIFENRFMHVQELTRLGAQIKLDGQT
str.JK73       LEVLQKTGLDIEIKPQGLHLKRNPRQTKIMPVNIKTGPYPAFPTDLQAQFMALMTRAQGTACITETIFENRFMHVQELTRLGAQIKLDGQT
str.JK7        LEVLQKTGLDIEIKPQGLHLKRNPRQTKIMPVNIKTGPYPAFPTDLQAQFMALMTRAQGTACITETIFENRFMHVQELTRLGAQIKLDGQT
str.JK73rel    LEVLQKTGLDIEIKPQGLHLKRNPRQTKIMPVNIKTGPYPAFPTDLQAQFMALMTRAQGTACITETIFENRFMHVQELTRLGAQIKLDGQT
str.CO20_0297  LEVLQKTGLDIEIKPQGLHLKRNPRQTKIMPVNIKTGPYPAFPTDLQAQFMALMTRAQGTACITETIFENRFMHVQELTRLGAQIKLDGQT
str.CO21_0024  LEVLQKTGLDIEIKPQGLHLKRNPRQTKIMPVNIKTGPYPAFPTDLQAQFMALMTRAQGTACITETIFENRFMHVQELTRLGAQIKLDGQT
str.CO20_0321  LEVLQKTGLDIEIKPQGLHLKRNPRQTKIMPVNIKTGPYPAFPTDLQAQFMALMTRAQGTACITETIFENRFMHVQELTRLGAQIKLDGQT
str.BQ2-D70    LEVLQKTGLDIEIKPQGLHLKRNPRQTKIMPVNIKTGPYPAFPTDLQAQFMALMTRAQGTACITETIFENRFMHVQELTRLGAQIKLDGQT
str.CO20_0256  LEVLQKTGLDIEIKPQGLHLKRNPRQTKIMPVNIKTGPYPAFPTDLQAQFMALMTRAQGTACITETIFENRFMHVQELTRLGAQIKLDGQT
str.CO20_0257  LEVLQKTGLDIEIKPQGLHLKRNPRQTKIMPVNIKTGPYPAFPTDLQAQFMALMTRAQGTACITETIFENRFMHVQELTRLGAQIKLDGQT
str.NCTC12899  LEVLQKTGLDIEIKPQGLHLKRNPRQTKIMPVNIKTGPYPAFPTDLQAQFMALMTRAQGTACITETIFENRFMHVQELTRLGAQIKLDGQT
str.MF1-1      LEVLQKTGLDIEIKPQGLHLKRNPRQTKIMPVNIKTGPYPAFPTDLQAQFMALMTRAQGTACITETIFENRFMHVQELTRLGAQIKLDGQT
str.RM-11      LEVLQKTGLDIEIKPQGLHLKRNPRQTKIMPVNIKTGPYPAFPTDLQAQFMALMTRAQGTACITETIFENRFMHVQELTRLGAQIKLDGQT
```

```
                     370       380       390       400       410       420       430
str.Toulouse     ATVYGREHLQGAPVMATDLRASVSLVIAALAAKGESVINRVYHLDRGFERLEEKLARCGAIIQRITV
str.JK12         ATVYGREHLQGAPVMATDLRASVSLVIAALAAKGESVINRVYHLDRGFERLEEKLARCGAIIQRITV
str.CCUG45777    ATVYGREHLQGAPVMATDLRASVSLVIAALAAKGESVINRVYHLDRGFERLEEKLARCGAIIQRITV
str.JK56         ATVYGREHLQGAPVMATDLRASVSLVIAALAAKGESVINRVYHLDRGFERLEEKLARCGAIIQRITV
str.JK67         ATVYGREHLQGAPVMATDLRASVSLVIAALAAKGESVINRVYHLDRGFERLEEKLARCGAIIQRITV
str.JK19         ATVYGREHLQGAPVMATDLRASVSLVIAALAAKGESVINRVYHLDRGFERLEEKLARCGAIIQRITV
str.JK63         ATVYGREHLQGAPVMATDLRASVSLVIAALAAKGESVINRVYHLDRGFERLEEKLARCGAIIQRITV
str.JK39         ATVYGREHLQGAPVMATDLRASVSLVIAALAAKGESVINRVYHLDRGFERLEEKLARCGAIIQRITV
str.JK68         ATVYGREHLQGAPVMATDLRASVSLVIAALAAKGESVINRVYHLDRGFERLEEKLARCGAIIQRITV
str.JK31         ATVYGREHLQGAPVMATDLRASVSLVIAALAAKGESVINRVYHLDRGFERLEEKLARCGAIIQRITV
str.G1713        ATVYGREHLQGAPVMATDLRASVSLVIAALAAKGESVINRVYHLDRGFERLEEKLARCGAIIQRITV
str.G1712        ATVYGREHLQGAPVMATDLRASVSLVIAALAAKGESVINRVYHLDRGFERLEEKLARCGAIIQRITV
str.JK73         ATVYGREHLQGAPVMATDLRASVSLVIAALAAKGESVINRVYHLDRGFERLEEKLARCGAIIQRITV
str.JK7          ATVYGREHLQGAPVMATDLRASVSLVIAALAAKGESVINRVYHLDRGFERLEEKLARCGAIIQRITV
str.JK73rel      ATVYGREHLQGAPVMATDLRASVSLVIAALAAKGESVINRVYHLDRGFERLEEKLARCGAIIQRITV
str.CO20_0297    ATVYGREHLQGAPVMATDLRASVSLVIAALAAKGESVINRVYHLDRGFERLEEKLARCGAIIQRITV
str.CO21_0024    ATVYGREHLQGAPVMATDLRASVSLVIAALAAKGESVINRVYHLDRGFERLEEKLARCGAIIQRITV
str.CO20_0321    ATVYGREHLQGAPVMATDLRASVSLVIAALAAKGESVINRVYHLDRGFERLEEKLARCGAIIQRITV
str.BQ2-D70      ATVYGREHLQGAPVMATDLRASVSLVIAALAAKGESVINRVYHLDRGFERLEEKLARCGAIIQRITV
str.CO20_0256    ATVYGREHLQGAPVMATDLRASVSLVIAALAAKGESVINRVYHLDRGFERLEEKLARCGAIIQRITV
str.CO20_0257    ATVYGREHLQGAPVMATDLRASVSLVIAALAAKGESVINRVYHLDRGFERLEEKLARCGAIIQRITV
str.NCTC12899    ATVYGREHLQGAPVMATDLRASVSLVIAALAAKGESVINRVYHLDRGFERLEEKLARCGAIIQRITV
str.MF1-1        ATVYGREHLQGAPVMATDLRASVSLVIAALAAKGESVINRVYHLDRGFERLEEKLARCGAIIQRITV
str.RM-11        ATVYGREHLQGAPVMATDLRASVSLVIAALAAKGESVINRVYHLDRGFERLEEKLARCGAIIQRITV
```

>WP_011179951.1 saccharopine dehydrogenase family protein [Bartonella quintana str. Toulouse]

```
               1        10        20        30        40        50        60        70        80        90
str.Toulouse   MKKNVLIIGAGGVAQVVTHKCAQNNDVLGEIHIASRTLKKCEKIVKSIKDKKAMKVEGILKSHPLNAMNVAETVQLIQKTKCEIVINVGSS
str.JK12       MKKNVLIIGAGGVAQVVTHKCAQNNDVLGEIHIASRTLKKCEKIVKSIKDKKAMKVEGILKSHPLNAMNVAETVQLIQKTKCEIVINVGSS
str.CCUG45777  MKKNVLIIGAGGVAQVVTHKCAQNNDVLGEIHIASRTLKKCEKIVKSIKDKKAMKVEGILKSHPLNAMNVAETVQLIQKTKCEIVINVGSS
str.JK67       MKKNVLIIGAGGVAQVVTHKCAQNNDVLGEIHIASRTLKKCEKIVKSIKDKKAMKVEGILKSHPLNAMNVAETVQLIQKTKCEIVINVGSS
str.JK56       MKKNVLIIGAGGVAQVVTHKCAQNNDVLGEIHIASRTLKKCEKIVKSIKDKKAMKVEGILKSHPLNAMNVAETVQLIQKTKCEIVINVGSS
str.JK19       MKKNVLIIGAGGVAQVVTHKCAQNNDVLGEIHIASRTLKKCEKIVKSIKDKKAMKVEGILKSHPLNAMNVAETVQLIQKTKCEIVINVGSS
str.JK63       MKKNVLIIGAGGVAQVVTHKCAQNNDVLGEIHIASRTLKKCEKIVKSIKDKKAMKVEGILKSHPLNAMNVAETVQLIQKTKCEIVINVGSS
str.JK39       MKKNVLIIGAGGVAQVVTHKCAQNNDVLGEIHIASRTLKKCEKIVKSIKDKKAMKVEGILKSHPLNAMNVAETVQLIQKTKCEIVINVGSS
str.JK68       MKKNVLIIGAGGVAQVVTHKCAQNNDVLGEIHIASRTLKKCEKIVKSIKDKKAMKVEGILKSHPLNAMNVAETVQLIQKTKCEIVINVGSS
str.JK73       MKKNVLIIGAGGVAQVVTHKCAQNNDVLGEIHIASRTLKKCEKIVKSIKDKKAMKVEGILKSHPLNAMNVAETVQLIQKTKCEIVINVGSS
str.G1712      MKKNVLIIGAGGVAQVVTHKCAQNNDVLGEIHIASRTLKKCEKIVKSIKDKKAMKVEGILKSHPLNAMNVAETVQLIQKTKCEIVINVGSS
str.G1713      MKKNVLIIGAGGVAQVVTHKCAQNNDVLGEIHIASRTLKKCEKIVKSIKDKKAMKVEGILKSHPLNAMNVAETVQLIQKTKCEIVINVGSS
str.JK31       MKKNVLIIGAGGVAQVVTHKCAQNNDVLGEIHIASRTLKKCEKIVKSIKDKKAMKVEGILKSHPLNAMNVAETVQLIQKTKCEIVINVGSS
str.JK7        MKKNVLIIGAGGVAQVVTHKCAQNNDVLGEIHIASRTLKKCEKIVKSIKDKKAMKVEGILKSHPLNAMNVAETVQLIQKTKCEIVINVGSS
str.CO20_0321  MKKNVLIIGAGGVAQVVTHKCAQNNDVLGEIHIASRTLKKCEKIVKSIKDKKAMKVEGILKSHPLNAMNVAETVQLIQKTKCEIVINVGSS
str.BQ2-D70    MKKNVLIIGAGGVAQVVTHKCAQNNDVLGEIHIASRTLKKCEKIVKSIKDKKAMKVEGILKSHPLNAMNVAETVQLIQKTKCEIVINVGSS
str.JK73rel    MKKNVLIIGAGGVAQVVTHKCAQNNDVLGEIHIASRTLKKCEKIVKSIKDKKAMKVEGILKSHPLNAMNVAETVQLIQKTKCEIVINVGSS
str.CO21_0024  MKKNVLIIGAGGVAQVVTHKCAQNNDVLGEIHIASRTLKKCEKIVKSIKDKKAMKVEGILKSHPLNAMNVAETVQLIQKTKCEIVINVGSS
str.CO20_0297  MKKNVLIIGAGGVAQVVTHKCAQNNDVLGEIHIASRTLKKCEKIVKSIKDKKAMKVEGILKSHPLNAMNVAETVQLIQKTKCEIVINVGSS
str.CO20_0256  MKKNVLIIGAGGVAQVVTHKCAQNNDVLGEIHIASRTLKKCEKIVKSIKDKKAMKVEGILKSHPLNAMNVAETVQLIQKTKCEIVINVGSS
str.CO20_0257  MKKNVLIIGAGGVAQVVTHKCAQNNDVLGEIHIASRTLKKCEKIVKSIKDKKAMKVEGILKSHPLNAMNVAETVQLIQKTKCEIVINVGSS
str.NCTC12899  MKKNVLIIGAGGVAQVVTHKCAQNNDVLGEIHIASRTLKKCEKIVKSIKDKKAMKVEGILKSHPLNAMNVAETVQLIQKTKCEIVINVGSS
str.MF1-1      MKKNVLIIGAGGVAQVVTHKCAQNNDVLGEIHIASRTLKKCETIVKSIKDKKAMKVEGILKSHPLNAMNVAETVQLIQKTKCEIVINVGSS
str.RM-11      MKKNVLIIGAGGVAQVVTHKCAQNNDVLGEIHIASRTLKKCETIVKSIKDKKAMKVEGILKSHPLNAMNVAETVQLIQKTKCEIVINVGSS

                    100       110       120       130       140       150       160       170       180
str.Toulouse   FLNMSVLSACIETKCAYIDTAIHEDALKICETPPWYGNYEWPRRQECRNAGITAILGAGFDPGVVNAYAALARDNYFDKITDIDIIDINAG
str.JK12       FLNMSVLSACIETKCAYIDTAIHEDALKICETPPWYGNYEWPRRQECRNAGITAILGAGFDPGVVNAYAALARDNYFDKITDIDIIDINAG
str.CCUG45777  FLNMSVLSACIETKCAYIDTAIHEDALKICETPPWYGNYEWPRRQECRNAGITAILGAGFDPGVVNAYAALARDNYFDKITDIDIIDINAG
str.JK67       FLNMSVLSACIETKCAYIDTAIHEDALKICETPPWYGNYEWPRRQECRNAGITAILGAGFDPGVVNAYAALARDNYFDKITDIDIIDINAG
str.JK56       FLNMSVLSACIETKCAYIDTAIHEDALKICETPPWYGNYEWPRRQECRNAGITAILGAGFDPGVVNAYAALARDNYFDKITDIDIIDINAG
str.JK19       FLNMSVLSACIETKCAYIDTAIHEDALKICETPPWYGNYEWPRRQECRNAGITAILGAGFDPGVVNAYAALARDNYFDKITDIDIIDINAG
str.JK63       FLNMSVLSACIETKCAYIDTAIHEDALKICETPPWYGNYEWPRRQECRNAGITAILGAGFDPGVVNAYAALARDNYFDKITDIDIIDINAG
str.JK39       FLNMSVLSACIETKCAYIDTAIHEDALKICETPPWYGNYEWPRRQECRNAGITAILGAGFDPGVVNAYAALARDNYFDKITDIDIIDINAG
str.JK68       FLNMSVLSACIETKCAYIDTAIHEDALKICETPPWYGNYEWPRRQECRNAGITAILGAGFDPGVVNAYAALARDNYFDKITDIDIIDINAG
str.JK73       FLNMSVLSACIETKCAYIDTAIHEDALKICETPPWYGNYEWPRRQECRNAGITAILGAGFDPGVVNAYAALARDNYFDKITDIDIIDINAG
str.G1712      FLNMSVLSACIETKCAYIDTAIHEDALKICETPPWYGNYEWPRRQECRNAGITAILGAGFDPGVVNAYAALARDNYFDKITDIDIIDINAG
str.G1713      FLNMSVLSACIETKCAYIDTAIHEDALKICETPPWYGNYEWPRRQECRNAGITAILGAGFDPGVVNAYAALARDNYFDKITDIDIIDINAG
str.JK31       FLNMSVLSACIETKCAYIDTAIHEDALKICETPPWYGNYEWPRRQECRNAGITAILGAGFDPGVVNAYAALARDNYFDKITDIDIIDINAG
str.JK7        FLNMSVLSACIETKCAYIDTAIHEDALKICETPPWYGNYEWPRRQECRNAGITAILGAGFDPGVVNAYAALARDNYFDKITDIDIIDINAG
str.CO20_0321  FLNMSVLSACIETKCAYIDTAIHEDALKICETPPWYGNYEWPRRQECRNAGITAILGAGFDPGVVNAYAALARDNYFDKITDIDIIDINAG
str.BQ2-D70    FLNMSVLSACIETKCAYIDTAIHEDALKICETPPWYGNYEWPRRQECRNAGITAILGAGFDPGVVNAYAALARDNYFDKITDIDIIDINAG
str.JK73rel    FLNMSVLSACIETKCAYIDTAIHEDALKICETPPWYGNYEWPRRQECRNAGITAILGAGFDPGVVNAYAALARDNYFDKITDIDIIDINAG
str.CO21_0024  FLNMSVLSACIETKCAYIDTAIHEDALKICETPPWYGNYEWPRRQECRNAGITAILGAGFDPGVVNAYAALARDNYFDKITDIDIIDINAG
str.CO20_0297  FLNMSVLSACIETKCAYIDTAIHEDALKICETPPWYGNYEWPRRQECRNAGITAILGAGFDPGVVNAYAALARDNYFDKITDIDIIDINAG
str.CO20_0256  FLNMSVLSACIETKCAYIDTAIHEDALKICETPPWYGNYEWPRRQECRNAGITAILGAGFDPGVVNAYAALARDNYFDKITDIDIIDINAG
str.CO20_0257  FLNMSVLSACIETKCAYIDTAIHEDALKICETPPWYGNYEWPRRQECRNAGITAILGAGFDPGVVNAYAALARDNYFDKITDIDIIDINAG
str.NCTC12899  FLNMSVLSACIETKCAYIDTAIHEDALKICETPPWYGNYEWPRRQECRNAGITAILGAGFDPGVVNAYAALARDNYFDKITDIDIIDINAG
str.MF1-1      FLNMSVLSACIETKCAYIDTAIHEDALKICETPPWYGNYEWPRRQECRNAGITAILGAGFDPGVVNAYAALARDDYFDKITDIDIIDINAG
str.RM-11      FLNMSVLSACIETKCAYIDTAIHEDALKICETPPWYGNYEWPRRQECRNAGITAILGAGFDPGVVNAYAALARDDYFDKITDIDIIDINAG

                      190       200       210       220       230       240       250       260       270
str.Toulouse   NHGRWFATNFDPEINFREFTGQVWSWQNKKWTSNQMFEVSREWDLPVVGKQKTYMTGHDEIHSLSKNLDVPNIRFWMGFSERYITVFTVLK
str.JK12       NHGRWFATNFDPEINFREFTGQVWSWQNKKWTSNQMFEVSREWDLPVVGKQKTYMTGHDEIHSLSKNLDVPNIRFWMGFSERYITVFTVLK
str.CCUG45777  NHGRWFATNFDPEINFREFTGQVWSWQNKKWTSNQMFEVSREWDLPVVGKQKTYMTGHDEIHSLSKNLDVPNIRFWMGFSERYITVFTVLK
str.JK67       NHGRWFATNFDPEINFREFTGQVWSWQNKKWTSNQMFEVSREWDLPVVGKQKTYMTGHDEIHSLSKNLDVPNIRFWMGFSERYITVFTVLK
str.JK56       NHGRWFATNFDPEINFREFTGQVWSWQNKKWTSNQMFEVSREWDLPVVGKQKTYMTGHDEIHSLSKNLDVPNIRFWMGFSERYITVFTVLK
str.JK19       NHGRWFATNFDPEINFREFTGQVWSWQNKKWTSNQMFEVSREWDLPVVGKQKTYMTGHDEIHSLSKNLDVPNIRFWMGFSERYITVFTVLK
str.JK63       NHGRWFATNFDPEINFREFTGQVWSWQNKKWTSNQMFEVSREWDLPVVGKQKTYMTGHDEIHSLSKNLDVPNIRFWMGFSERYITVFTVLK
str.JK39       NHGRWFATNFDPEINFREFTGQVWSWQNKKWTSNQMFEVSREWDLPVVGKQKTYMTGHDEIHSLSKNLDVPNIRFWMGFSERYITVFTVLK
str.JK68       NHGRWFATNFDPEINFREFTGQVWSWQNKKWTSNQMFEVSREWDLPVVGKQKTYMTGHDEIHSLSKNLDVPNIRFWMGFSERYITVFTVLK
str.JK73       NHGRWFATNFDPEINFREFTGQVWSWQNKKWTSNQMFEVSREWDLPVVGKQKTYMTGHDEIHSLSKNLDVPNIRFWMGFSERYITVFTVLK
str.G1712      NHGRWFATNFDPEINFREFTGQVWSWQNKKWTSNQMFEVSREWDLPVVGKQKTYMTGHDEIHSLSKNLDVPNIRFWMGFSERYITVFTVLK
str.G1713      NHGRWFATNFDPEINFREFTGQVWSWQNKKWTSNQMFEVSREWDLPVVGKQKTYMTGHDEIHSLSKNLDVPNIRFWMGFSERYITVFTVLK
str.JK31       NHGRWFATNFDPEINFREFTGQVWSWQNKKWTSNQMFEVSREWDLPVVGKQKTYMTGHDEIHSLSKNLDVPNIRFWMGFSERYITVFTVLK
str.JK7        NHGRWFATNFDPEINFREFTGQVWSWQNKKWTSNQMFEVSREWDLPVVGKQKTYMTGHDEIHSLSKNLDVPNIRFWMGFSERYITVFTVLK
str.CO20_0321  NHGRWFATNFDPEINFREFTGQVWSWQNKKWTSNQMFEVSREWDLPVVGKQKTYMTGHDEIHSLSKNLDVPNIRFWMGFSERYITVFTVLK
str.BQ2-D70    NHGRWFATNFDPEINFREFTGQVWSWQNKKWTSNQMFEVSREWDLPVVGKQKTYMTGHDEIHSLSKNLDVPNIRFWMGFSERYITVFTVLK
str.JK73rel    NHGRWFATNFDPEINFREFTGQVWSWQNKKWTSNQMFEVSREWDLPVVGKQKTYMTGHDEIHSLSKNLDVPNIRFWMGFSERYITVFTVLK
str.CO21_0024  NHGRWFATNFDPEINFREFTGQVWSWQNKKWTSNQMFEVSREWDLPVVGKQKTYMTGHDEIHSLSKNLDVPNIRFWMGFSERYITVFTVLK
str.CO20_0297  NHGRWFATNFDPEINFREFTGQVWSWQNKKWTSNQMFEVSREWDLPVVGKQKTYMTGHDEIHSLSKNLDVPNIRFWMGFSERYITVFTVLK
str.CO20_0256  NHGRWFATNFDPEINFREFTGQVWSWQNKKWTSNQMFEVSREWDLPVVGKQKTYMTGHDEIHSLSKNLDVPNIRFWMGFSERYITVFTVLK
str.CO20_0257  NHGRWFATNFDPEINFREFTGQVWSWQNKKWTSNQMFEVSREWDLPVVGKQKTYMTGHDEIHSLSKNLDVPNIRFWMGFSERYITVFTVLK
str.NCTC12899  NHGRWFATNFDPEINFREFTGQVWSWQNKKWTSNQMFEVSREWDLPVVGKQKTYMTGHDEIHSLSKNLDVPNIRFWMGFSERYITVFTVLK
str.MF1-1      NHGRWFATNFDPEINFREFTGQVWSWQNKKWTSNQMFEVSREWDLPVVGKQKTYMTGHDEIHSLSKNLDVPNIRFWMGFSERYITVFTVLK
str.RM-11      NHGRWFATNFDPEINFREFTGQVWSWQNKKWTSNQMFEVSREWDLPVVGKQKTYMTGHDEIHSLSKNLDVPNIRFWMGFSERYITVFTVLK

                    280       290       300       310       320       330       340       350       360
str.Toulouse   NLGLLSEQPIKTAEGQEVVPLKVVKAVLPNPASLAPDYIGKTCIGDLIKGEKGGKPREVFIYNITDHKQAFNETGAQGISYTAGVPAAAAA
str.JK12       NLGLLSEQPIKTAEGQEVVPLKVVKAVLPNPASLAPDYIGKTCIGDLIKGEKGGKPREVFIYNITDHKQAFNETGAQGISYTAGVPAAAAA
str.CCUG45777  NLGLLSEQPIKTAEGQEVVPLKVVKAVLPNPASLAPDYIGKTCIGDLIKGEKGGKPREVFIYNITDHKQAFNETGAQGISYTAGVPAAAAA
str.JK67       NLGLLSEQPIKTAEGQEVVPLKVVKAVLPNPASLAPDYIGKTCIGDLIKGEKGGKPREVFIYNITDHKQAFNETGAQGISYTAGVPAAAAA
str.JK56       NLGLLSEQPIKTAEGQEVVPLKVVKAVLPNPASLAPDYIGKTCIGDLIKGEKGGKPREVFIYNITDHKQAFNETGAQGISYTAGVPAAAAA
str.JK19       NLGLLSEQPIKTAEGQEVVPLKVVKAVLPNPASLAPDYIGKTCIGDLIKGEKGGKPREVFIYNITDHKQAFNETGAQGISYTAGVPAAAAA
str.JK63       NLGLLSEQPIKTAEGQEVVPLKVVKAVLPNPASLAPDYIGKTCIGDLIKGEKGGKPREVFIYNITDHKQAFNETGAQGISYTAGVPAAAAA
str.JK39       NLGLLSEQPIKTAEGQEVVPLKVVKAVLPNPASLAPDYIGKTCIGDLIKGEKGGKPREVFIYNITDHKQAFNETGAQGISYTAGVPAAAAA
str.JK68       NLGLLSEQPIKTAEGQEVVPLKVVKAVLPNPASLAPDYIGKTCIGDLIKGEKGGKPREVFIYNITDHKQAFNETGAQGISYTAGVPAAAAA
str.JK73       NLGLLSEQPIKTAEGQEVVPLKVVKAVLPNPASLAPDYIGKTCIGDLIKGEKGGKPREVFIYNITDHKQAFNETGAQGISYTAGVPAAAAA
str.G1712      NLGLLSEQPIKTAEGQEVVPLKVVKAVLPNPASLAPDYIGKTCIGDLIKGEKGGKPREVFIYNITDHKQAFNETGAQGISYTAGVPAAAAA
str.G1713      NLGLLSEQPIKTAEGQEVVPLKVVKAVLPNPASLAPDYIGKTCIGDLIKGEKGGKPREVFIYNITDHKQAFNETGAQGISYTAGVPAAAAA
str.JK31       NLGLLSEQPIKTAEGQEVVPLKVVKAVLPNPASLAPDYIGKTCIGDLIKGEKGGKPREVFIYNITDHKQAFNETGAQGISYTAGVPAAAAA
str.JK7        NLGLLSEQPIKTAEGQEVVPLKVVKAVLPNPASLAPDYIGKTCIGDLIKGEKGGKPREVFIYNITDHKQAFNETGAQGISYTAGVPAAAAA
str.CO20_0321  NLGLLSEQPIKTAEGQEVVPLKVVKAVLPNPASLAPDYIGKTCIGDLIKGEKGGKPREVFIYNITDHKQAFNETGAQGISYTAGVPAAAAA
str.BQ2-D70    NLGLLSEQPIKTAEGQEVVPLKVVKAVLPNPASLAPDYIGKTCIGDLIKGEKGGKPREVFIYNITDHKQAFNETGAQGISYTAGVPAAAAA
str.JK73rel    NLGLLSEQPIKTAEGQEVVPLKVVKAVLPNPASLAPDYIGKTCIGDLIKGEKGGKPREVFIYNITDHKQAFNETGAQGISYTAGVPAAAAA
str.CO21_0024  NLGLLSEQPIKTAEGQEVVPLKVVKAVLPNPASLAPDYIGKTCIGDLIKGEKGGKPREVFIYNITDHKQAFNETGAQGISYTAGVPAAAAA
str.CO20_0297  NLGLLSEQPIKTAEGQEVVPLKVVKAVLPNPASLAPDYIGKTCIGDLIKGEKGGKPREVFIYNITDHKQAFNETGAQGISYTAGVPAAAAA
str.CO20_0256  NLGLLSEQPIKTAEGQEVVPLKVVKAVLPNPASLAPDYIGKTCIGDLIKGEKGGKPREVFIYNITDHKQAFNETGAQGISYTAGVPAAAAA
str.CO20_0257  NLGLLSEQPIKTAEGQEVVPLKVVKAVLPNPASLAPDYIGKTCIGDLIKGEKGGKPREVFIYNITDHKQAFNETGAQGISYTAGVPAAAAA
str.NCTC12899  NLGLLSEQPIKTAEGQEVVPLKVVKAVLPNPASLAPDYIGKTCIGDLIKGEKGGKPREVFIYNITDHKQAFNETGAQGISYTAGVPAAAAA
str.MF1-1      NLGLLSEQPIKTAEGQEVVPLKVVKAVLPNPASLAPDYTGKTCIGDLIKGEKGGKPREVFIYNITDHKQAFNETGAQGISYTAGVPAAAAA
str.RM-11      NLGLLSEQPIKTAEGQEVVPLKVVKAVLPNPASLAPDYTGKTCIGDLIKGEKGGKPREVFIYNITDHKQAFNETGAQGISYTAGVPAAAAA
```

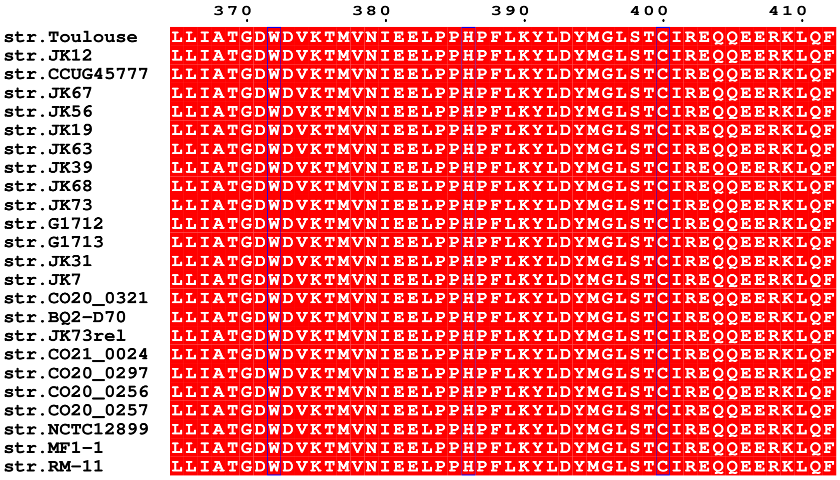
370 380 390 400 410
str.Toulouse LLIATGDWDVKTMVNIEELPPHPFLKYLDYMGLSTCIREQQEERKLQF
str.JK12 LLIATGDWDVKTMVNIEELPPHPFLKYLDYMGLSTCIREQQEERKLQF
str.CCUG45777 LLIATGDWDVKTMVNIEELPPHPFLKYLDYMGLSTCIREQQEERKLQF
str.JK67 LLIATGDWDVKTMVNIEELPPHPFLKYLDYMGLSTCIREQQEERKLQF
str.JK56 LLIATGDWDVKTMVNIEELPPHPFLKYLDYMGLSTCIREQQEERKLQF
str.JK19 LLIATGDWDVKTMVNIEELPPHPFLKYLDYMGLSTCIREQQEERKLQF
str.JK63 LLIATGDWDVKTMVNIEELPPHPFLKYLDYMGLSTCIREQQEERKLQF
str.JK39 LLIATGDWDVKTMVNIEELPPHPFLKYLDYMGLSTCIREQQEERKLQF
str.JK68 LLIATGDWDVKTMVNIEELPPHPFLKYLDYMGLSTCIREQQEERKLQF
str.JK73 LLIATGDWDVKTMVNIEELPPHPFLKYLDYMGLSTCIREQQEERKLQF
str.G1712 LLIATGDWDVKTMVNIEELPPHPFLKYLDYMGLSTCIREQQEERKLQF
str.G1713 LLIATGDWDVKTMVNIEELPPHPFLKYLDYMGLSTCIREQQEERKLQF
str.JK31 LLIATGDWDVKTMVNIEELPPHPFLKYLDYMGLSTCIREQQEERKLQF
str.JK7 LLIATGDWDVKTMVNIEELPPHPFLKYLDYMGLSTCIREQQEERKLQF
str.CO20_0321 LLIATGDWDVKTMVNIEELPPHPFLKYLDYMGLSTCIREQQEERKLQF
str.BQ2-D70 LLIATGDWDVKTMVNIEELPPHPFLKYLDYMGLSTCIREQQEERKLQF
str.JK73rel LLIATGDWDVKTMVNIEELPPHPFLKYLDYMGLSTCIREQQEERKLQF
str.CO21_0024 LLIATGDWDVKTMVNIEELPPHPFLKYLDYMGLSTCIREQQEERKLQF
str.CO20_0297 LLIATGDWDVKTMVNIEELPPHPFLKYLDYMGLSTCIREQQEERKLQF
str.CO20_0256 LLIATGDWDVKTMVNIEELPPHPFLKYLDYMGLSTCIREQQEERKLQF
str.CO20_0257 LLIATGDWDVKTMVNIEELPPHPFLKYLDYMGLSTCIREQQEERKLQF
str.NCTC12899 LLIATGDWDVKTMVNIEELPPHPFLKYLDYMGLSTCIREQQEERKLQF
str.MF1-1 LLIATGDWDVKTMVNIEELPPHPFLKYLDYMGLSTCIREQQEERKLQF
str.RM-11 LLIATGDWDVKTMVNIEELPPHPFLKYLDYMGLSTCIREQQEERKLQF

>WP_011179960.1 diaminopimelate epimerase [Bartonella quintana str. Toulouse]

```
              1         10        20        30        40        50        60        70        80        90
str.Toulouse  MKTPFRKMDGLGNQIIVADMRESTHALTPQAILALAADPQTHFDQIMTIHSSTQKEADFRIEIWNADGSMAKACGNGTRCVIAWLTDHNLG
str.NCTC12899 MKTPFRKMDGLGNQIIVADMRESTHALTPQAILALAADPQTHFDQIMTIHSSTQKEADFRIEIWNADGSMAKACGNGTRCVIAWLTDHNLG
str.CO20_0257 MKTPFRKMDGLGNQIIVADMRESTHALTPQAILALAADPQTHFDQIMTIHSSTQKEADFRIEIWNADGSMAKACGNGTRCVIAWLTDHNLG
str.CO20_0256 MKTPFRKMDGLGNQIIVADMRESTHALTPQAILALAADPQTHFDQIMTIHSSTQKEADFRIEIWNADGSMAKACGNGTRCVIAWLTDHNLG
str.CO20_0297 MKTPFRKMDGLGNQIIVADMRESTHALTPQAILALAADPQTHFDQIMTIHSSTQKEADFRIEIWNADGSMAKACGNGTRCVIAWLTDHNLG
str.CO21_0024 MKTPFRKMDGLGNQIIVADMRESTHALTPQAILALAADPQTHFDQIMTIHSSTQKEADFRIEIWNADGSMAKACGNGTRCVIAWLTDHNLG
str.CO20_0321 MKTPFRKMDGLGNQIIVADMRESTHALTPQAILALAADPQTHFDQIMTIHSSTQKEADFRIEIWNADGSMAKACGNGTRCVIAWLTDHNLG
str.BQ2-D70   MKTPFRKMDGLGNQIIVADMRESTHALTPQAILALAADPQTHFDQIMTIHSSTQKEADFRIEIWNADGSMAKACGNGTRCVIAWLTDHNLG
str.JK73rel   MKTPFRKMDGLGNQIIVADMRESTHALTPQAILALAADPQTHFDQIMTIHSSTQKEADFRIEIWNADGSMAKACGNGTRCVIAWLTDHNLG
str.JK7       MKTPFRKMDGLGNQIIVADMRESTHALTPQAILALAADPQTHFDQIMTIHSSTQKEADFRIEIWNADGSMAKACGNGTRCVIAWLTDHNLG
str.JK73      MKTPFRKMDGLGNQIIVADMRESTHALTPQAILALAADPQTHFDQIMTIHSSTQKEADFRIEIWNADGSMAKACGNGTRCVIAWLTDHNLG
str.G1712     MKTPFRKMDGLGNQIIVADMRESTHALTPQAILALAADPQTHFDQIMTIHSSTQKEADFRIEIWNADGSMAKACGNGTRCVIAWLTDHNLG
str.G1713     MKTPFRKMDGLGNQIIVADMRESTHALTPQAILALAADPQTHFDQIMTIHSSTQKEADFRIEIWNADGSMAKACGNGTRCVIAWLTDHNLG
str.JK31      MKTPFRKMDGLGNQIIVADMRESTHALTPQAILALAADPQTHFDQIMTIHSSTQKEADFRIEIWNADGSMAKACGNGTRCVIAWLTDHNLG
str.JK68      MKTPFRKMDGLGNQIIVADMRESTHALTPQAILALAADPQTHFDQIMTIHSSTQKEADFRIEIWNADGSMAKACGNGTRCVIAWLTDHNLG
str.JK39      MKTPFRKMDGLGNQIIVADMRESTHALTPQAILALAADPQTHFDQIMTIHSSTQKEADFRIEIWNADGSMAKACGNGTRCVIAWLTDHNLG
str.JK63      MKTPFRKMDGLGNQIIVADMRESTHALTPQAILALAADPQTHFDQIMTIHSSTQKEADFRIEIWNADGSMAKACGNGTRCVIAWLTDHNLG
str.JK19      MKTPFRKMDGLGNQIIVADMRESTHALTPQAILALAADPQTHFDQIMTIHSSTQKEADFRIEIWNADGSMAKACGNGTRCVIAWLTDHNLG
str.JK67      MKTPFRKMDGLGNQIIVADMRESTHALTPQAILALAADPQTHFDQIMTIHSSTQKEADFRIEIWNADGSMAKACGNGTRCVIAWLTDHNLG
str.JK56      MKTPFRKMDGLGNQIIVADMRESTHALTPQAILALAADPQTHFDQIMTIHSSTQKEADFRIEIWNADGSMAKACGNGTRCVIAWLTDHNLG
str.JK12      MKTPFRKMDGLGNQIIVADMRESTHALTPQAILALAADPQTHFDQIMTIHSSTQKEADFRIEIWNADGSMAKACGNGTRCVIAWLTDHNLG
str.CCUG45777 MKTPFRKMDGLGNQIIVADMRESTHALTPQAILALAADPQTHFDQIMTIHSSTQKEADFRIEIWNADGSMAKACGNGTRCVIAWLTDHNLG
str.RM-11     MKTPFRKMDGLGNQIIVADMRESTHALTPQAILALAADPQTHFDQIMTIHSSTQKEADFRIEIWNADGSMAKACGNGTRCVIAWLTDHNLG
str.MF1-1     MKTPFRKMDGLGNQIIVADMRESTHALTPQAILALAADPQTHFDQIMTIHSSTQKEADFRIEIWNADGSMAKACGNGTRCVIAWLTDHNLG
```

```
                  100       110       120       130       140       150       160       170       180
str.Toulouse  ESFRLETPAGIIEGKRQTDNLISVDMGCPNFNAKEMPVSREIADTNYVKITAGPLKDACLVSIGNLHAIFFVEDNIQQIPLEKYGPKLEHD
str.NCTC12899 ESFRLETPAGIIEGKRQTDNLISVDMGCPNFNAKEMPVSREIADTNYVKITAGPLKDACLVSIGNLHAIFFVEDNIQQIPLEKYGPKLEHD
str.CO20_0257 ESFRLETPAGIIEGKRQTDNLISVDMGCPNFNAKEMPVSREIADTNYVKITAGPLKDACLVSIGNLHAIFFVEDNIQQIPLEKYGPKLEHD
str.CO20_0256 ESFRLETPAGIIEGKRQTDNLISVDMGCPNFNAKEMPVSREIADTNYVKITAGPLKDACLVSIGNLHAIFFVEDNIQQIPLEKYGPKLEHD
str.CO20_0297 ESFRLETPAGIIEGKRQTDNLISVDMGCPNFNAKEMPVSREIADTNYVKITAGPLKDACLVSIGNLHAIFFVEDNIQQIPLEKYGPKLEHD
str.CO21_0024 ESFRLETPAGIIEGKRQTDNLISVDMGCPNFNAKEMPVSREIADTNYVKITAGPLKDACLVSIGNLHAIFFVEDNIQQIPLEKYGPKLEHD
str.CO20_0321 ESFRLETPAGIIEGKRQTDNLISVDMGCPNFNAKEMPVSREIADTNYVKITAGPLKDACLVSIGNLHAIFFVEDNIQQIPLEKYGPKLEHD
str.BQ2-D70   ESFRLETPAGIIEGKRQTDNLISVDMGCPNFNAKEMPVSREIADTNYVKITAGPLKDACLVSIGNLHAIFFVEDNIQQIPLEKYGPKLEHD
str.JK73rel   ESFRLETPAGIIEGKRQTDNLISVDMGCPNFNAKEMPVSREIADTNYVKITAGPLKDACLVSIGNLHAIFFVEDNIQQIPLEKYGPKLEHD
str.JK7       ESFRLETPAGIIEGKRQTDNLISVDMGCPNFNAKEMPVSREIADTNYVKITAGPLKDACLVSIGNLHAIFFVEDNIQQIPLEKYGPKLEHD
str.JK73      ESFRLETPAGIIEGKRQTDNLISVDMGCPNFNAKEMPVSREIADTNYVKITAGPLKDACLVSIGNLHAIFFVEDNIQQIPLEKYGPKLEHD
str.G1712     ESFRLETPAGIIEGKRQTDNLISVDMGCPNFNAKEMPVSREIADTNYVKITAGPLKDACLVSIGNLHAIFFVEDNIQQIPLEKYGPKLEHD
str.G1713     ESFRLETPAGIIEGKRQTDNLISVDMGCPNFNAKEMPVSREIADTNYVKITAGPLKDACLVSIGNLHAIFFVEDNIQQIPLEKYGPKLEHD
str.JK31      ESFRLETPAGIIEGKRQTDNLISVDMGCPNFNAKEMPVSREIADTNYVKITAGPLKDACLVSIGNLHAIFFVEDNIQQIPLEKYGPKLEHD
str.JK68      ESFRLETPAGIIEGKRQTDNLISVDMGCPNFNAKEMPVSREIADTNYVKITAGPLKDACLVSIGNLHAIFFVEDNIQQIPLEKYGPKLEHD
str.JK39      ESFRLETPAGIIEGKRQTDNLISVDMGCPNFNAKEMPVSREIADTNYVKITAGPLKDACLVSIGNLHAIFFVEDNIQQIPLEKYGPKLEHD
str.JK63      ESFRLETPAGIIEGKRQTDNLISVDMGCPNFNAKEMPVSREIADTNYVKITAGPLKDACLVSIGNLHAIFFVEDNIQQIPLEKYGPKLEHD
str.JK19      ESFRLETPAGIIEGKRQTDNLISVDMGCPNFNAKEMPVSREIADTNYVKITAGPLKDACLVSIGNLHAIFFVEDNIQQIPLEKYGPKLEHD
str.JK67      ESFRLETPAGIIEGKRQTDNLISVDMGCPNFNAKEMPVSREIADTNYVKITAGPLKDACLVSIGNLHAIFFVEDNIQQIPLEKYGPKLEHD
str.JK56      ESFRLETPAGIIEGKRQTDNLISVDMGCPNFNAKEMPVSREIADTNYVKITAGPLKDACLVSIGNLHAIFFVEDNIQQIPLEKYGPKLEHD
str.JK12      ESFRLETPAGIIEGKRQTDNLISVDMGCPNFNAKEMPVSREIADTNYVKITAGPLKDACLVSIGNLHAIFFVEDNIQQIPLEKYGPKLEHD
str.CCUG45777 ESFRLETPAGIIEGKRQTDNLISVDMGCPNFNAKEMPVSREIADTNYVKITAGPLKDACLVSIGNLHAIFFVEDNIQQIPLEKYGPKLEHD
str.RM-11     ESFRLETPAGIIEGKRQTDNLISVDMGCPNFNAKEMPVSREIADTNYVKITAGPLKDACLVSIGNLHAIFFVEDNIQQIPLEKYGPKLEHD
str.MF1-1     ESFRLETPAGIIEGKRQTDNLISVDMGCPNFNAKEMPVSREIADTNYVKITAGPLKDACLVSIGNLHAIFFVEDNIQQIPLEKYGPKLEHD
```

```
                 190       200       210       220       230       240       250       260       270
str.Toulouse  PLFPERCNISIASVTSHKSLNLRTWERGAGLTKACGSAACASAVAAYRRGLTQRHIDVNLPGGTLNIVYREDNHIVMTGPTKYEFGGFLNP
str.NCTC12899 PLFPERCNISIASVTSHKSLNLRTWERGAGLTKACGSAACASAVAAYRRGLTQRHIDVNLPGGTLNIVYREDNHIVMTGPTKYEFGGFLNP
str.CO20_0257 PLFPERCNISIASVTSHKSLNLRTWERGAGLTKACGSAACASAVAAYRRGLTQRHIDVNLPGGTLNIVYREDNHIVMTGPTKYEFGGFLNP
str.CO20_0256 PLFPERCNISIASVTSHKSLNLRTWERGAGLTKACGSAACASAVAAYRRGLTQRHIDVNLPGGTLNIVYREDNHIVMTGPTKYEFGGFLNP
str.CO20_0297 PLFPERCNISIASVTSHKSLNLRTWERGAGLTKACGSAACASAVAAYRRGLTQRHIDVNLPGGTLNIVYREDNHIVMTGPTKYEFGGFLNP
str.CO21_0024 PLFPERCNISIASVTSHKSLNLRTWERGAGLTKACGSAACASAVAAYRRGLTQRHIDVNLPGGTLNIVYREDNHIVMTGPTKYEFGGFLNP
str.CO20_0321 PLFPERCNISIASVTSHKSLNLRTWERGAGLTKACGSAACASAVAAYRRGLTQRHIDVNLPGGTLNIVYREDNHIVMTGPTKYEFGGFLNP
str.BQ2-D70   PLFPERCNISIASVTSHKSLNLRTWERGAGLTKACGSAACASAVAAYRRGLTQRHIDVNLPGGTLNIVYREDNHIVMTGPTKYEFGGFLNP
str.JK73rel   PLFPERCNISIASVTSHKSLNLRTWERGAGLTKACGSAACASAVAAYRRGLTQRHIDVNLPGGTLNIVYREDNHIVMTGPTKYEFGGFLNP
str.JK7       PLFPERCNISIASVTSHKSLNLRTWERGAGLTKACGSAACASAVAAYRRGLTQRHIDVNLPGGTLNIVYREDNHIVMTGPTKYEFGGFLNP
str.JK73      PLFPERCNISIASVTSHKSLNLRTWERGAGLTKACGSAACASAVAAYRRGLTQRHIDVNLPGGTLNIVYREDNHIVMTGPTKYEFGGFLNP
str.G1712     PLFPERCNISIASVTSHKSLNLRTWERGAGLTKACGSAACASAVAAYRRGLTQRHIDVNLPGGTLNIVYREDNHIVMTGPTKYEFGGFLNP
str.G1713     PLFPERCNISIASVTSHKSLNLRTWERGAGLTKACGSAACASAVAAYRRGLTQRHIDVNLPGGTLNIVYREDNHIVMTGPTKYEFGGFLNP
str.JK31      PLFPERCNISIASVTSHKSLNLRTWERGAGLTKACGSAACASAVAAYRRGLTQRHIDVNLPGGTLNIVYREDNHIVMTGPTKYEFGGFLNP
str.JK68      PLFPERCNISIASVTSHKSLNLRTWERGAGLTKACGSAACASAVAAYRRGLTQRHIDVNLPGGTLNIVYREDNHIVMTGPTKYEFGGFLNP
str.JK39      PLFPERCNISIASVTSHKSLNLRTWERGAGLTKACGSAACASAVAAYRRGLTQRHIDVNLPGGTLNIVYREDNHIVMTGPTKYEFGGFLNP
str.JK63      PLFPERCNISIASVTSHKSLNLRTWERGAGLTKACGSAACASAVAAYRRGLTQRHIDVNLPGGTLNIVYREDNHIVMTGPTKYEFGGFLNP
str.JK19      PLFPERCNISIASVTSHKSLNLRTWERGAGLTKACGSAACASAVAAYRRGLTQRHIDVNLPGGTLNIVYREDNHIVMTGPTKYEFGGFLNP
str.JK67      PLFPERCNISIASVTSHKSLNLRTWERGAGLTKACGSAACASAVAAYRRGLTQRHIDVNLPGGTLNIVYREDNHIVMTGPTKYEFGGFLNP
str.JK56      PLFPERCNISIASVTSHKSLNLRTWERGAGLTKACGSAACASAVAAYRRGLTQRHIDVNLPGGTLNIVYREDNHIVMTGPTKYEFGGFLNP
str.JK12      PLFPERCNISIASVTSHKSLNLRTWERGAGLTKACGSAACASAVAAYRRGLTQRHIDVNLPGGTLNIVYREDNHIVMTGPTKYEFGGFLNP
str.CCUG45777 PLFPERCNISIASVTSHKSLNLRTWERGAGLTKACGSAACASAVAAYRRGLTQRHIDVNLPGGTLNIVYREDNHIVMTGPTKYEFGGFLNP
str.RM-11     PLFPERCNISIASVTSHKSLNLRTWERGAGLTQACGSAACASAVAAYRRGLTQRHIDVNLPGGTLNIVYREDNHIVMTGPTKYEFGGFLNP
str.MF1-1     PLFPERCNISIASVTSHKSLNLRTWERGAGLTQACGSAACASAVAAYRRGLTQRHIDVNLPGGTLNIFYREDNHIVMTGPTKYEFGGFLNP
```

```
                280
str.Toulouse  LTGTYKKDHF
str.NCTC12899 LTGTYKKDHF
str.CO20_0257 LTGTYKKDHF
str.CO20_0256 LTGTYKKDHF
str.CO20_0297 LTGTYKKDHF
str.CO21_0024 LTGTYKKDHF
str.CO20_0321 LTGTYKKDHF
str.BQ2-D70   LTGTYKKDHF
str.JK73rel   LTGTYKKDHF
str.JK7       LTGTYKKDHF
str.JK73      LTGTYKKDHF
str.G1712     LTGTYKKDHF
str.G1713     LTGTYKKDHF
str.JK31      LTGTYKKDHF
str.JK68      LTGTYKKDHF
str.JK39      LTGTYKKDHF
str.JK63      LTGTYKKDHF
str.JK19      LTGTYKKDHF
str.JK67      LTGTYKKDHF
str.JK56      LTGTYKKDHF
str.JK12      LTGTYKKDHF
str.CCUG45777 LTGTYKKDHF
str.RM-11     LTGTYKKDHF
str.MF1-1     LTGTYKKDHF
```

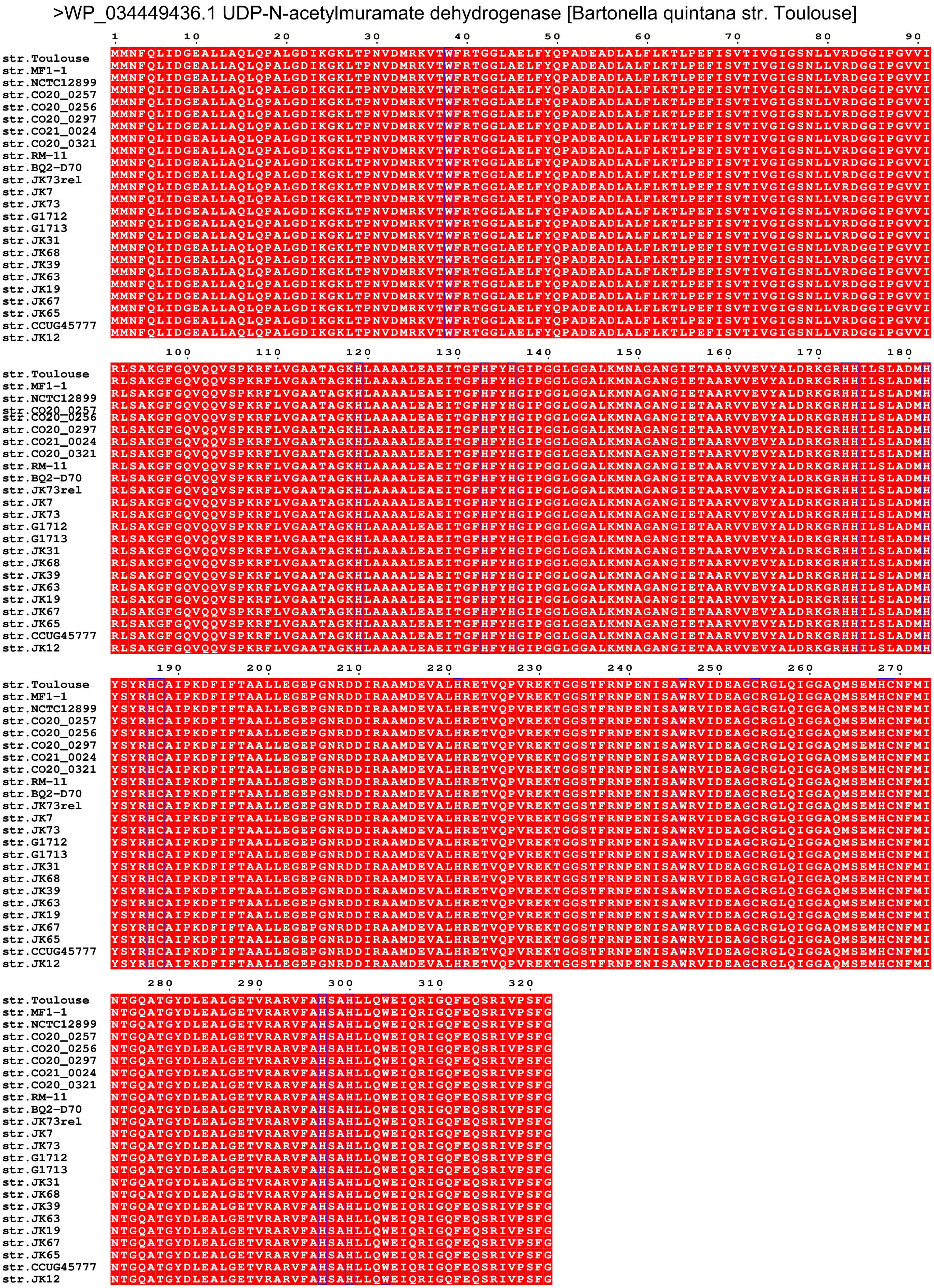

Fig. 1: multiple sequence alignment of the 28 target sequences of *Bartonella quintana* str. Toulouse across the 25 *B. quintana* strains using CLUSTAL W. At the start of each sequence, the word (str.) represents the strain, followed by the strain name. Shaded residues are conserved across all the strains, while unshaded residues are not conserved. It’s clear from the alignments that the targets share a high identity among all strains.

1) >WP_011179066.1 lipid IV(A) 3-deoxy-D-manno-octulosonic acid transferase (*Bartonella quintana* str. Toulouse)

MVELKAHAALLIYRMIGFCLRPLVPFYLFFRAIRGKEEWNRKKERLGKSHQVRPQSPLIWLHAASVGETLALFPLINYILSLKINVLLTTGTVTSSYLVRKHFDDRLIHQYAPLDLDLAVRRFISHWKPDLALTCESEIWPLRIKELAKMRIPQILVNAHMSERSFKAWQKRRILARHIFKHIDLAIAQNERDVAYYRALGIKSVALSGNLKADVFWAEDQALLAYYRAAIGNRPVWAAVSTHEGEEEIAFEVHKILKNYFPDLLTIIVPRHPERSEDLIKKCDNKSLRFIRRSNNAIPARDTDVLLGDTIGEMGLFLRLSKVSFIGKSLCGDGGHNPLELALLGSAILTGPHISNFQEMFEQFLTCDAACMVQDTKQLAIQVYRLLTNEALRQEMVDKAYEVATDMAGALERTLRALDPFLQPLVIQTVLSQHRGRYAY.

2) >WP_011179434.1 lipid-A-disaccharide synthase (*Bartonella quintana*)

MNNCFLKIAVVAGEESGDSLGADLISCLSQQTGCNIHLIGVGGRHLKTLGLKSIFNFHDIALIGLGAVLKKLPLLLIHIHNLSKLIAQEQPDCLIIIDSPDFTHRVAKKVRSLAPSIPIIKYVAPTVWAWRPERARAMRKFVDHVLAVFPFEEKIMTDLEGPPTTYVGHRLLTYPPLLTVQSEKKHSFGKQASFLTLIVLPGSRNLEIRYLMPIFGEAVEILAQRIPNLRIILPTLPHLVDEIRCFVQKWKSKVEIVVGEEAKWRAFADANVALAALGTVSLELALARIPMVLCYKLDRFSKFFIFPKIMLWSAALPNILSDKPIVPEYFNEFLRPGMLARQIEQLLHNPLLRQAQLDAFELMEQKMKTEVPPGIIAAQTIITLLKEKLGHLKFS.

3) >WP_011179606.1 phospho-N-acetylmuramoyl-pentapeptide-transferase (*Bartonella quintana*)

MMLFFSSLSDWFPGVSVFRYITFRTVAAMLTSGLIVFLFGPSIIASLKLRQGKGQPIRADGPQTHFKKAGTPTMGGLMILTGIVVSAFLWCNLSNIYFWVSLFVMLSFGMIGFYDDYLKVTKQTEKGFSGKARLSLEFLIAIIAAFVLLQVGSSGLALPFVKDYFINLSWFFLPFSAFVIVGTGNAVNLTDGLDGLAIVPVMVAALSFALIAYLSGNINFADYLQIHYVSGTGELAVLLGAVVGAGLGFLWFNAPPAAIFMGDTGSLALGGLLGIVAVATKHEIVLALIGGLFVLEGFSVVIQVGYFKLKKKRVFLMAPIHHHFEKKGWTESQVVIRFWIISIVLALVGLSTLKLR.

4) >WP_011179609.1 penicillin-binding protein 2 (*Bartonella quintana*)

MKSLFLFSQKKKRLNNQLDFHNFSIRRSYSARPRLLFSLLCFLILYGVIGACLISYGLEGG
QIEEAKGPGVLQLTARPDIIDRNGRLLATDIKTYSLFAEPRRVIDVDETIELLSTVLSDL
NWHETYKRLKRKSGFSWIQRGLTPTQKAQIMALGIPGIGFRPEIRRFYPGGSVASHILG
MVNVDNQGIAGMEKYIDDAGLSALRSAGLATEEALKPIQLSIDVRIQAIVHDELIKAMKR
YKAIAAGAVILNIHTNEVLAMVSVPDFDPGNPVDALKSDRLNRITAGAFEMGSIMKSFTT
AMALDSDMFHLNSLIDASKPIQASSGYIIHDFHGKNRPLTLWEVFIYSSNIGSAKEA
LAIGIEKHRAFLKKLGLLDRLTTELPEVTHPIVPRHWKDIHSMTISFGHGMATTPLQTAV
GAAALMNGGWLIAPTFLKRTKEQALKQAKQVLQAKTSQNMRYLYKLNSDIGSG
RNAKVEGYRVGGKTGTAEKVENGKYSKTKNFNSFLAAFPIEDPAYVVLTIIDEPKP
EDGKYAATAGLNAGPMLSNIVRRSASFLGIKPDFKKEYDSILSTKNSSRLVKQR.

5) >WP_011179659.1 ABC transporter permease (*Bartonella quintana*)

MAIMNHPKPQSLFSLSRWLNESVPRSIIQAKLQKIYHSVIKFSRNFSAVFGLIIIFVMILCAV
FAPWIATHDFVSNDLAHRLQPPSMLHYLGTDELGRDIFSRLVFGTRITLYIIFLTTIIV
GPIGLIIGTVSGYIGGWVDTLLMRIVDIFLAFPGLILALAFAAALGPGIENASIAISIAAWPPI
ARLARAETLTIRSSDYVSAVRLQGASAWRIILFHVAPMCIPSVIVRLTLDMSGIILTAAGLG
FLGLGAQLPSPEWGAMLSTGREFMMTCWWVAAMPGCAILCASLAFNLLGDGLRDIL
DPRNG.

6) >WP_011179677.1 membrane protein insertase YidC (*Bartonella quintana*)

MEYNRNFFIAIGLSFGVLIAWHFFYVAPKQAQLQQKRLIAQQLSKQQSTLSTSTPYFSDN
ASITHESASIITHPITPEIRNEALAKTNRIAIKTDELEGSINLVGAQFDDLLLKKYRLTVD
KKSPEIALLNPKGFTTTYLAEFGFTSSSLSAKALPQSDTQWQIEGNNTTLTPSTPVT
LIYNNGQGQIFRRTLSVDNHYMFTIEDSIKNESDKPIYLSSYARVARAAPPEHTNATYL
LHEGMIGIASDSLKTEKYKTLAELNPNPDNSQKSITFSKNIGGWIGITDKYWAVAVIPP
QDKEYTSRFIYFDRLNTHYQSDLLGSLLTVAPNETKIVTNRLFAGAKQVEIINHYQNDLKI
KKFALLIDWGWFDFITKPMFSLIDTLYKQTGNFGIAILLVTVLLKTLLFPLANKSYKSMAR
MKLIQPMLLEIKEKYPDDRTKQQQAIIELYKTQKINPLAGCWPMLIQFPIFFALYKVL
YITIEMRHAPFFGWIQDLAAPDPTSLFNLFGLLPYTVPTFLMLGAWPLIMGITMFLQMR
MNPAPQDQTQAMIFAWMPVIFTFMLASFPVGLVIYWAWNNILSILQQSILMKRQGVKI
ELFDNLKAMWWKSPKKEAHK.

Fig. 2: linear B cell epitope prediction analyses were performed with algorithms (ABCPred, BCPred, and BepiPred) showing consensus sequences in all evaluated proteins. The red bold underlined with a black background shows the antigenic epitopes predicted by at least two algorithms. (1) lipid IV(A) 3-deoxy-D-manno-octulosonic acid transferase; (2) lipid-A-disaccharide synthase; (3) phospho-N-acetylmuramoyl-pentapeptide transferase; (4) penicillin-binding protein; (5) ABC transporter permease; and (6) membrane protein insertase YidC.
